# Piperazine‐Derived Bisphosphonate‐Based Ionizable Lipid Nanoparticles Enhance mRNA Delivery to the Bone Microenvironment

**DOI:** 10.1002/anie.202415389

**Published:** 2024-12-13

**Authors:** Il‐Chul Yoon, Lulu Xue, Qinyuan Chen, Jingyi Liu, Junchao Xu, Zain Siddiqui, Dongyoon Kim, Bingling Chen, Qiangqiang Shi, Emily Laura Han, Mia Cherry Ruiz, Kyle H. Vining, Michael J. Mitchell

**Affiliations:** ^1^ Department of Bioengineering School of Engineering and Applied Science University of Pennsylvania Philadelphia PA 19104 United States; ^2^ Department of Materials Science and Engineering School of Engineering and Applied Science University of Pennsylvania Philadelphia PA 19104 United States; ^3^ Preventive and Restorative Sciences School of Dental Medicine University of Pennsylvania Philadelphia PA 19104 United States; ^4^ Abramson Cancer Center Perelman School of Medicine University of Pennsylvania Philadelphia PA 19104 United States; ^5^ Center for Cellular Immunotherapies Perelman School of Medicine University of Pennsylvania Philadelphia PA 19104 United States; ^6^ Penn Institute for RNA Innovation Perelman School of Medicine University of Pennsylvania Philadelphia PA 19104 United States; ^7^ Institute for Immunology Perelman School of Medicine University of Pennsylvania Philadelphia PA 19104 United States; ^8^ Cardiovascular Institute Perelman School of Medicine University of Pennsylvania Philadelphia PA 19104 United States; ^9^ Institute for Regenerative Medicine Perelman School of Medicine University of Pennsylvania Philadelphia PA 19104 United States

**Keywords:** Bisphosphonate, Lipid nanoparticles, mRNA delivery, Piperazine-based ionizable lipids, Targeting bone microenvironment

## Abstract

Nucleic acid delivery with mRNA lipid nanoparticles are being developed for targeting a wide array of tissues and cell types. However, targeted delivery to the bone microenvironment remains a significant challenge in the field, due in part to low local blood flow and poor interactions between drug carriers and bone material. Here we report bone‐targeting ionizable lipids incorporating a piperazine backbone and bisphosphate moieties, which bind tightly with hydroxyapatite ([Ca_5_(PO_4_)_3_OH]), a key component of mineralized tissues. These lipids demonstrate biocompatibility and low toxicity in both vitro and in vivo studies. LNP formulated with these lipids facilitated efficient cellular transfection and improved binding to hydroxyapatite in vitro, and targeted delivery to the bone microenvironment in vivo following systemic administration. Overall, our findings demonstrate the critical role of the piperazine backbone in a novel ionizable lipid, which incorporates a bisphosphonate group to enable efficient bone‐targeted delivery, highlighting the potential of rational design of ionizable lipids for next‐generation bone‐targeting delivery systems.

## Graphical abstract

Piperazine‐based bisphosphonate‐linked ionizable lipids for enhanced lipid nanoparticle (LNP)‐mediated messenger RNA (mRNA) delivery to the bone microenvironment through systemic administration.

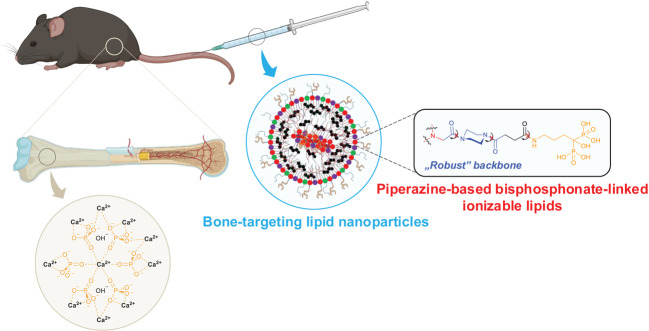

## Introduction

Bones, as a type of connective tissue, are predominantly composed of collagen, which is mineralized with calcium‐phosphate complexes in a lattice‐like structure, increasing hardness and strength.[^1^, ^3^] Additionally, blood vessels located in the bone marrow supply nutrients, while the nervous system transmits signals.^[6]^ Bone tissue consists of osteoblasts, which secrete calcium, phosphate, and collagen. Osteoclasts can resorb aged bone tissue to maintain serum calcium levels, while osteocytes, the most abundant cells in bone tissue, are responsible for maintaining bone structure and mineral metabolism through signaling within the bone matrix.[^2^, ^9^, ^13^] Dysregulation of the signaling pathways involving these cells can lead to diseases such as osteoporosis, osteogenesis imperfecta, osteonecrosis, and osteosarcoma.[^2^, ^16^] These diseases are influenced by genetic factors and disease complexity. Current treatments provide only symptomatic relief and are associated with clinical challenges and potential side effects.^[30]^ The bone microenvironment remains a challenge for targeted delivery due to low blood flow and a dense cortical structure,^[32]^ which may reduce bioavailability of drugs in the bone tissue. Local delivery of therapeutics to bone tissue would open the door for new targeted therapies for mineralized tissue diseases.^[34]^

Bisphosphonates (BPs) with structures resembling phosphates are commonly prescribed as drugs (e.g., alendronate) for diseases, such as osteoporosis.^[38]^ BPs inhibit bone resorption, promote bone formation, and increase bone strength and density by enhancing calcium deposition on bone trabecular surfaces and inhibiting the activity of osteoclasts. Recently, lipid nanoparticles (LNPs) have gained attention as a carrier for nucleic acid therapeutics, notably the COVID‐19 mRNA vaccines.^[43]^ LNPs offer several advantages as a clinically‐approved non‐viral delivery system, such as their high biocompatibility. For the treatment of bone‐related diseases, LNPs can be designed with BP compounds to target mineralized tissues.[^49^, ^53^] We propose that BP LNPs may facilitate targeted nucleic acid delivery to the bone microenvironments.

**Figure 1:**
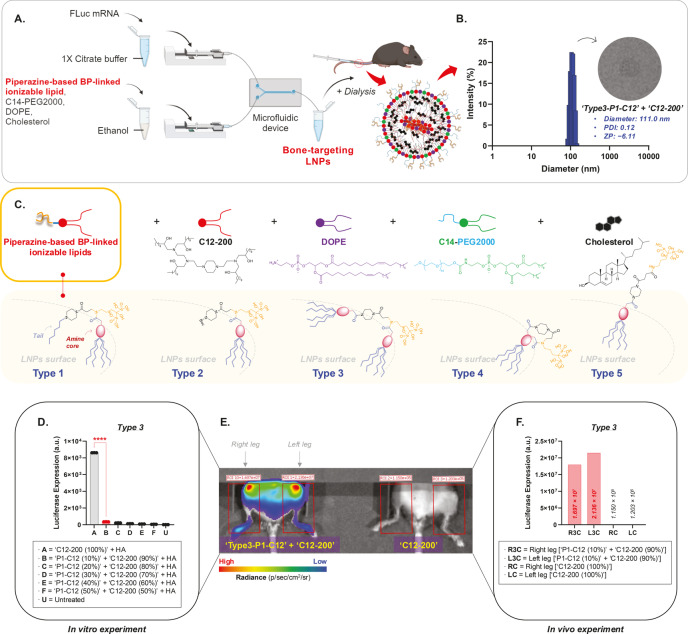
Efficient delivery of mRNA in the bone microenvironment using bisphosphonate‐based LNPs linked to a robust piperazine scaffold. (a) Illustration of systemic delivery of piperazine‐based bisphosphonate‐linked LNPs (PIP‐BP LNPs) to bone, and (b) the hydrodynamic diameter and TEM image of top‐performing PIP‐BP‐LNPs (′**Type** **3**‐**P1**‐**C12** (10 %)′+′**C12**‐**200** (90 %)′) (Figure S17). (c) Components of the LNPs with 5 different types of PIP‐BP ionizable lipids. (d) Luciferase expression in the BJ cell line, 5.000 cells of the BJ cell line, and 10 ng of FLuc mRNA were treated in each well. (e) Bioluminescence of in vivo transfection results, and (f) quantification of in vivo transfection results. ****p<0.0001. Ordinary one‐way ANOVA with Tukey's multiple comparisons test, *n*=3.

In our previous work, LNPs were manufactured with alendronate‐modified ionizable lipids to facilitate mRNA delivery to various cell types in bones.^[58]^ However, long‐term or excessive use of BPs typically leads to adverse effects such as gastrointestinal issues, decreased blood supply to bones resulting in increased fracture risks, and renal problems. There remains a need for rationally designed BP‐modified ionizable lipids that improve stability and mitigate potential side effects.^[59]^ Herein, to address these needs, a novel library of piperazine‐based BP ionizable lipids was successfully developed using a robust piperazine ring structure (PIP)[^63^, ^64^, ^65^, ^66^, ^67^, ^68^] conjugated to a BP group (Figure 1).^[53]^ PIP‐BP ionizable lipids were categorized into five types, resulting in a library comprising a total of 140 lipids. PIP‐BP based LNPs exhibited excellent cellular uptake, luciferase transfection through lysosomal‐endosomal escape,[^43^, ^44^, ^63^, ^74^] and binding affinity with hydroxyapatite. Furthermore, incorporating only 10 % of our ′**PIP**‐**based BP**‐**linked’** ionizable lipid with 90 % of a gold‐standard ′**C12**‐**200**′ ionizable lipid enabled efficient in vivo mRNA delivery to bone tissues.^[75]^ The results support our hypothesis that the rigidity of PIP‐based ionizable lipids benefits the overall structure of LNPs for bone targeting applications. These new lipid‐like materials have the potential to advance mRNA‐LNP therapies in regenerative medicine, protein replacement, and gene editing applications.

Here, we control the targeted delivery of mRNA into the bone microenvironment using the binding of a PIP‐BP group to hydroxyapatite via modification of the ionizable lipid of LNPs. The ionizable lipid is used for targeting instead of the typical PEG‐phospholipids, because PEG‐phospholipid constitutes only about 2 % of LNPs′ composition, and more importantly, modifying the terminal group of PEG‐phospholipids with BP presents challenges in controlling the orientation of these BP on the LNP surface. Specifically, due to the flexible ether group‘s chemical structure, some BP‐linked PEG‐phospholipids could be present on the surface of LNPs, while others could be located inside the LNPs.

These PIP‐BP ionizable lipids are distinct from previous BP ionizable lipids in two main aspects: PIP‐based linkages and incorporation of alkyl chains attached to the PIP terminal group. First, to achieve strong binding to bones, it is crucial for the terminal group of PIP‐BP ionizable lipids, as depicted in Figure 1, to maintain a chemical skeleton on the surface of LNPs. To accomplish this, we introduced a chemically structured PIP with a chair‐form configuration to immobilize the movement of BP molecules.[^53^, ^65^] Inspired by the robust structure that can be formed when this PIP ring undergoes amide bond formation with acyl groups via coupling reactions, we synthesized novel ionizable lipids. Additionally, these amides play a vital role in enhancing luciferase expression by assisting in the late stage of endosomal escape of mRNA. Secondly, in addition to the fixation of BP on the surface of LNPs,[^53^, ^65^] diverse synthesis designs of ionizable lipids were developed chemically to facilitate lysosomal‐endosomal escape (**Type** **1** to **Type** **5**).[^43^, ^44^, ^63^, ^74^] We aimed to investigate the impact on lysosomal/endosomal escape by adjusting the ratio of hydrophilicity to hydrophobicity within the PIP‐BP ionizable lipids, demonstrated through protein expression (Figures S24–S28). Under acidic conditions, the formation of ionized forms through the resonance of the non‐bonding electron pairs on nitrogen is minimal. However, with PIP‐BP ionizable lipids, endosomal escape in the cytosol occurs via a ′flip‐flop′ mechanism, facilitated by the robust PIP backbone linkage following the shared bonding between BP and polyamines.^[76]^ This hypothesis underpinned the design of bone‐targeting ionizable lipids, as evidenced by the experimental results, wherein ′**Type1**‐**P1**‐**C12**′ and ′**Type3**‐**P1**‐**C12**′, characterized by polyalkyl chains with stronger hydrophobicity compared to the BP functional group within the ionizable lipid, exhibited notably high transfection efficiency. Notably, the transfection efficiency of most PIP‐BP LNPs is significantly higher than that of the previous bone‐targeting LNPs. A comparison between **Type** **1** and **Type** **2** elucidates how alkyl chains attached to the PIP terminal group disrupt the LNP membrane to induce mRNA release. In other words, long alkyl chains with hydrophobicity form loose associations with adjacent lipids and PEG units compared to the Boc protecting group, thereby enhancing mRNA escape capability in the late stage of endosomal escape. In contrast to **Type** **1**, **Type** **2**, **Type** **3**, and **Type** **4** primarily focus on maintaining the skeleton on the surface of LNPs. However, the structural drawback of **Type** **4** lies in the ring structure formed through cyclization, which may result in the random orientation of the bisphosphonate group on the surface of LNPs, either outward or inward. Finally, **Type** **5** was designed and synthesized to clearly demonstrate the unique rigidity of the acyl PIP backbone, bearing a similar skeletal structure to the ionizable lipids used in previously developed LNPs. Although lysosomal/endosomal escape studies were not performed here, additional experiments with AFM, SEM, and SAX can be used to study mechanisms of lysosomal/endosomal escape.^[81]^

These PIP‐BP ionizable lipids were initially formulated into LNPs using the commonly utilized molar ratios in lipid formulation, including 35 % piperazine‐based bisphosphonate‐linked ionizable lipid, 16 % DOPE phospholipid, 46.5 % cholesterol, and 2.5 % C14‐PEG2000. PIP‐BP ionizable lipids were utilized at a concentration of 35 % in the LNP formulation, whereas PEG units with longer polyether chains were employed at 2.5 %. This approach allows for more efficient bone‐targeting LNPs through the extensive use of PIP‐BP ionizable lipids. Typically, most lipids with epoxide‐based alkyl chains exhibit strong expression in the liver. Therefore, after the formulation of these LNPs, an initial screening was performed using the Hep‐G2 cell line to prioritize selecting lipids that facilitated the highest transfection. Through these experiments, an understanding of the mechanism of action of PIP‐BP ionizable lipids and LNPs within cells was achieved, and in‐depth studies on these fundamental principles are ongoing as an extension of this work. The PIP‐BP ionizable lipids utilized in this study have relatively large molecular weights compared to other ionizable lipids, and thus to produce stable LNPs with a uniform size distribution, were formulated in combination with the ′**C12**‐**200**′ ionizable lipid, which is based on a PIP backbone structure. The most effective PIP‐BP LNPs in in vivo experiments, ′**Type3**‐**P1**‐**C12**′+′**C12**‐**200**′, maintained physicochemical properties of 111 nm in size, 0.12 polydispersity index (PDI), and a δ value of −6.11 at the fixed molar ratio.

The ′**Type3**‐**P1**‐**C12**′ PIP‐BP lipid showed higher in vitro transfection than the previously published bisphosphonate lipid **‘490BP**‐**C14** ^[43]^’ (Figure S32). The BP‐containing LNPs incorporate PIP with a robust 6‐membered ring structure to maintain a rigid conformation due to the unique properties of acyl PIP. This allows them to orient towards the outer surface of LNPs, thereby forming strong bonds with bone components like hydroxyapatite, resulting in enhanced binding affinity to bone compared to conventional particles.

## Results and Discussion

### The Rationale Design, Synthesis, and Initial In Vitro Screening of Bone‐Targeting PIP‐BP Ionizable Lipids

**Figure 2:**
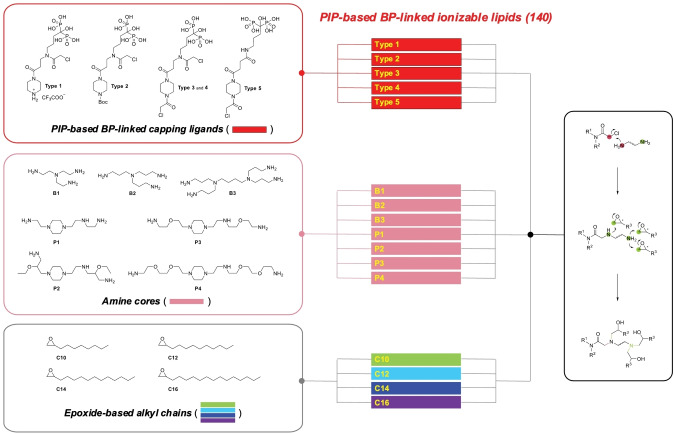
Schematic illustration of bone‐targeting ionizable lipid synthesis. ‘PIP‐BP capping ligands (5)’×‘Amine cores (7)’×‘Epoxide‐based alkyl chains (4)’=PIP‐BP ionizable lipids (140).

As depicted in Figure 2, PIP‐based BP‐linked ionizable lipids are constructed with three main components. The first part involves BP‐linked capping ligands based on PIP. These ligands are chemically designed to enable the generation of new PIP‐based BP‐linked ionizable lipids through chemical bonding with various forms of amines. Precursors used for the synthesis of PIP‐based BP‐linked ionizable lipids from **Type** **1** to **Type** **5** were synthesized on a multi‐gram scale via this route. The other components, amine cores, and epoxide‐based tails, are commercially available, enabling the design of a synthetic pathway for the synthesis of novel forms of bone‐targeting ionizable lipids through chemical bonding with BP‐linked capping ligands (Scheme S1). Notably, in the chemical synthesis, commercially available BPs such as alendronate, which are only soluble in water, were successfully synthesized using co‐solvent systems, allowing for the successful production of target molecules. Furthermore, most of the synthesized compounds were purified without using column‐based physical purification methods. Instead, compound separation was achieved through extraction and recrystallization methods utilizing differences in solubility, leveraging the physicochemical properties of the materials. The overall yield of the final products following this synthetic route and purification procedure is at a minimum of 60 %, with a purity of at least 90 %. Therefore, the designed synthetic pathways allow for immediate industrial application of the chemical processes. Currently, a total of 140 new PIP‐BP ionizable lipids have been synthesized using this synthetic route.

**Figure 3:**
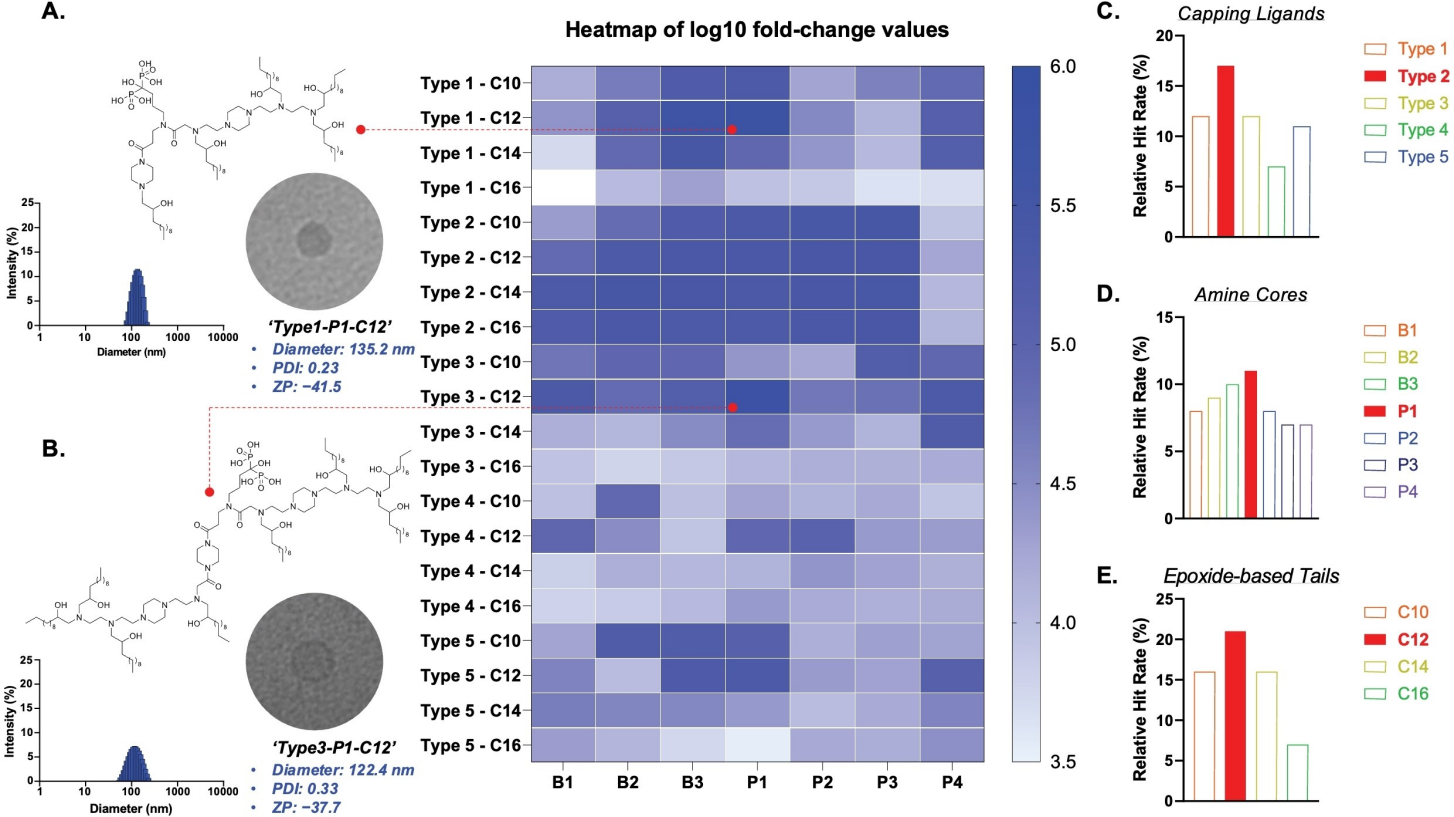
In vitro screening results of piperazine‐based bisphosphonate‐linked (PIP‐BP) LNPs in the HepG2 cell line, utilizing LNPs containing 100 % of PIP‐BP ionizable lipids alongside DOPE, cholesterol, and C14‐PEG2000 lipids. The hydrodynamic diameters and TEM images of (a) top‐performing PIP‐BP LNPs in the HepG2 cell line and (b) top‐performing PIP‐BP LNPs in the BJ cell line. (c) Analysis of the relationship between the chemical structure of PIP‐BP ionizable lipids and the protein expression efficiency of BP‐LNPs in vitro. 5.000 cells of the HepG2 cell line and 10 ng of FLuc mRNA were treated in each well.

The PIP‐BP LNPs were prepared using newly synthesized ionizable lipids at specific lipid formulation parameters (ionizable lipid/DOPE/cholesterol/C14‐PEG2000=35/16/46.5/2.5 %, molar ratios). The encapsulation efficiency of mRNA in these LNPs and the concentration of mRNA were measured using the RiboGreen assay. Screening both in vitro and in vivo utilized firefly luciferase (FLuc) mRNA substituted with 5‐methoxy‐U. To evaluate the structure–activity relationship of the PIP‐BP lipid library for mRNA delivery in vitro, LNPs containing FLuc mRNA were formulated and used to transfect HepG2 cells. The results of the initial screening of these PIP‐BP LNPs in the HepG2 cell line are presented in Figure 3 and Figures S24–S28 as luciferase expression. A heatmap was generated to evaluate the influence of various PIP‐BP structural parameters on in vitro mRNA delivery by calculating a relative hit rate. Among the PIP‐BP LNPs generated with the fixed molar ratios, LNPs based on ′**Type1**‐**P1**‐**C12**′ and ′**Type3**‐**P1**‐**C12**′ ionizable lipids exhibited the highest efficiencies (Figures 3a and 3b). A brief structure–activity reveals that overall, LNPs with **Type** **2** capping ligands bearing P1 amine cores and a 12‐carbon epoxide‐based alkyl chain showed superior transfection in the HepG2 cells (Figures 3c, 3d, and 3e). This could be attributed to the simple chemical structure of capping ligands similar to **Type** **2**, excluding the 1‐acyl‐4‐boc piperazine structure, which allows direct binding of alendronate to the amine cores, providing favorable conditions for interactions among adjacent lipids and lysosomal‐endosomal escape in the HepG2 cell line. From the perspective of amine cores, the analysis suggests that the packing among adjacent lipids facilitated by amines contributes to favorable transfection, analogous to the analysis of the relationship between chemical structure and luciferase expression through capping ligands.^[83]^ Comparing amine cores between the B series and P1, it is evident that transfection occurs more efficiently when a rigid backbone like PIP is present. Additionally, comparing amine cores within the P series, the simpler structure of the PIP‐based core P1 appears to favor transfection. Lastly, considering the relationship between tail length and transfection, it can be inferred that shorter tails are advantageous for transfection when the molecular size of the capping ligand and amine core is large, facilitating interactions among adjacent lipids. However, C12 is preferred over C10 because transfection seems to be determined by the shape, number, and size of the amine core. Furthermore, except for **Type** **3** capping ligands, which bind to one amine, the molecular size of the ionizable lipid center is relatively small in other Types, indicating that epoxide‐based alkyl chains with slightly longer lengths, such as 12 carbons, are generally advantageous for transfection.

**Figure 4:**
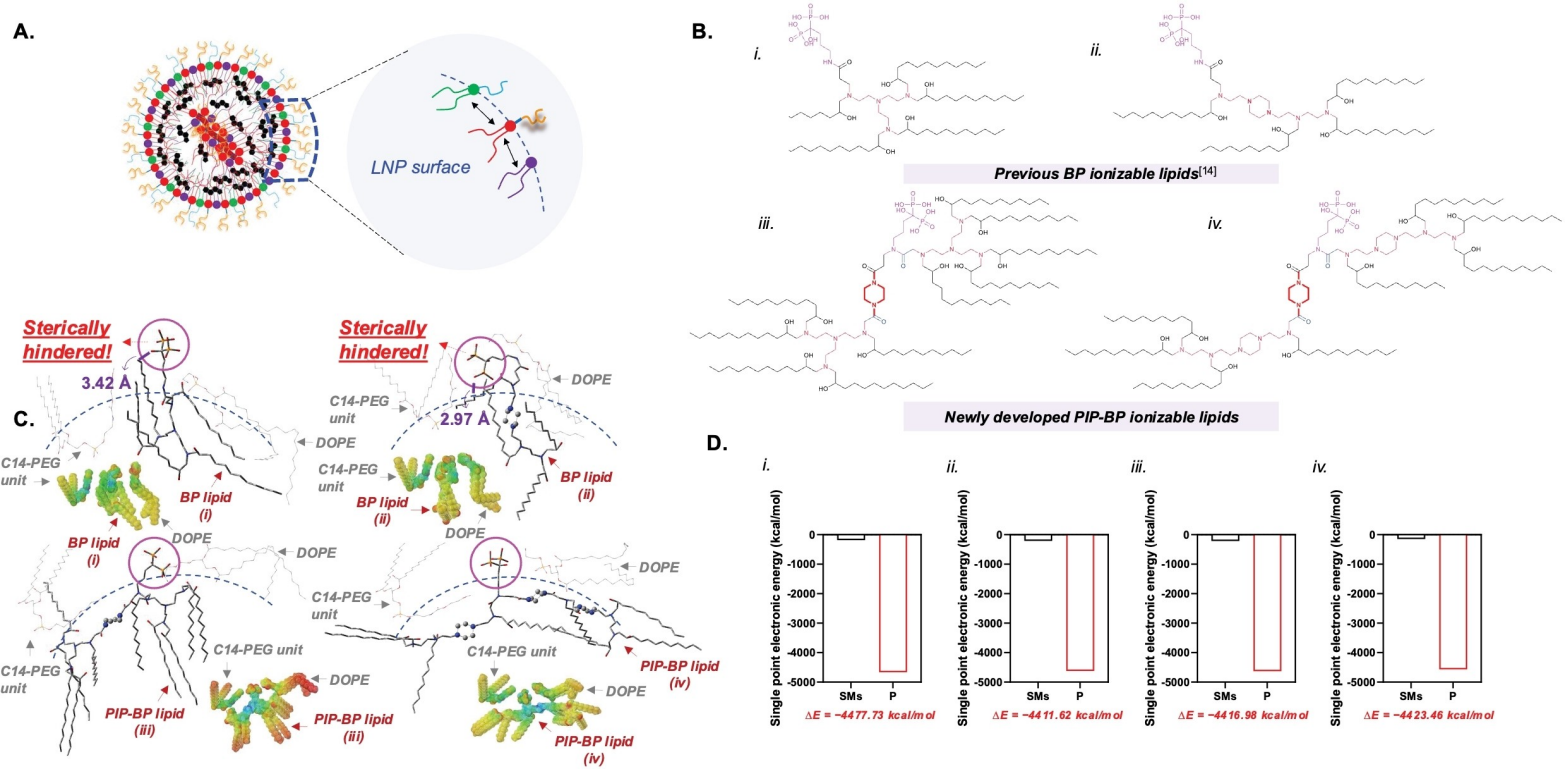
Simulation results on the correlation among bone‐targeting ionizable, C14‐PEG, and DOPE lipids on the surface of LNPs, based on structural conformation, energy, and electrostatic potential. (a) Pictorial representation of the correlation. (b and c) Chemical structures corresponding to the computed chemical structures on the LNP surface and (d) single‐point electronic energy values.

To gain a fundamental understanding of the relationship between chemical structure and luciferase expression, theoretical interpretations of interactions among adjacent lipids leading to packing and morphological analyses are essential. Both low toxicity in cells and high luciferase expression were observed (Figures S24–S31). Molecular dynamic simulations were conducted to further analyze the predicted chemical structures to compare PIP‐BP LNPs with conventional LNPs (Figure 4). The electrostatic potential map revealed that the amine groups exhibited electron‐deficient regions (blue‐colored region), facilitating efficient binding with mRNA. Conversely, the negatively charged BP groups (red‐colored circles) posed challenges for mRNA binding (Figure 4c). Through chemical structural interpretation based on the computed molecular structures, we could discern that the PIP‐based BP‐linked ionizable lipids were exposed on the LNPs′ surface. This suggests that they could interact with mineralized substrates (Figures 4b.iii and iv). In contrast, the chemical structure of conventional bone‐targeting ionizable lipids exhibited lower rigidity within LNPs (Figures 4b.i and ii), making them more susceptible to shielding by surrounding lipids or experiencing interference. Increasing the rigidity of the overall chemical structure of targeting lipids could facilitate terminal group functions. The calculated molecular structures showed that the thermodynamic energy was more stable (approximately −4,400 kcal/mol) when lipids interacted with each other rather than existing individually (Figure 4d). This indicates a tendency for the lipids to aggregate. Experimentally, it was also observed that the surface charges of these bone‐targeting LNPs are all around −30 mV, indicating that the bisphosphonate group, alendronate, remains exposed while maintaining the rigidity of the ionizable lipid on the surface of LNPs (Figures S24–S28, and Figure S38).

### Efficient Formulation of PIP‐BP LNPs

In vitro experiments were conducted on the BJ cell line, a fibroblast cell type, for the 16 top‐performing PIP‐BP LNPs selected from the initial screening (Figure S29). The hydroxyl groups generated by ring‐opening reactions of polyamines exhibit a high affinity for plasma proteins such as apolipoprotein E in the liver. Therefore, an initial screening was conducted using the HepG2 cell line, followed by further evaluation in the BJ fibroblast cell line to assess their efficacy with mesenchymal cells that reside in the bone microenvironment. Among the different types of PIP‐BP ionizable lipids, **Type** **1** with alkyl chains at the piperazine terminal group designed to disrupt the LNP membrane, and **Type** **3** with multiple amine groups, were found to be most advantageous for facilitating luciferase expression in BJ cells. Furthermore, this approach provided insights into mRNA transfection based on differences in cell morphology and LNP morphology. The interaction of LNPs with cells might be influenced by cell morphology.[^26^, ^27^, ^28^, ^29^, ^30^] While the polygonal‐shaped Hep‐G2 cell line exhibited the highest transfection efficiency with ′**Type** **1**‐**P1**‐**C12** (M.W. 1566.2309 g/mol)′, the spindle‐shaped fibroblast BJ cell line showed higher luciferase expression with the relatively larger ′**Type** **3**‐**P1**‐**C12** (M. W. 2374.5460 g/mol)′.

**Figure 5:**
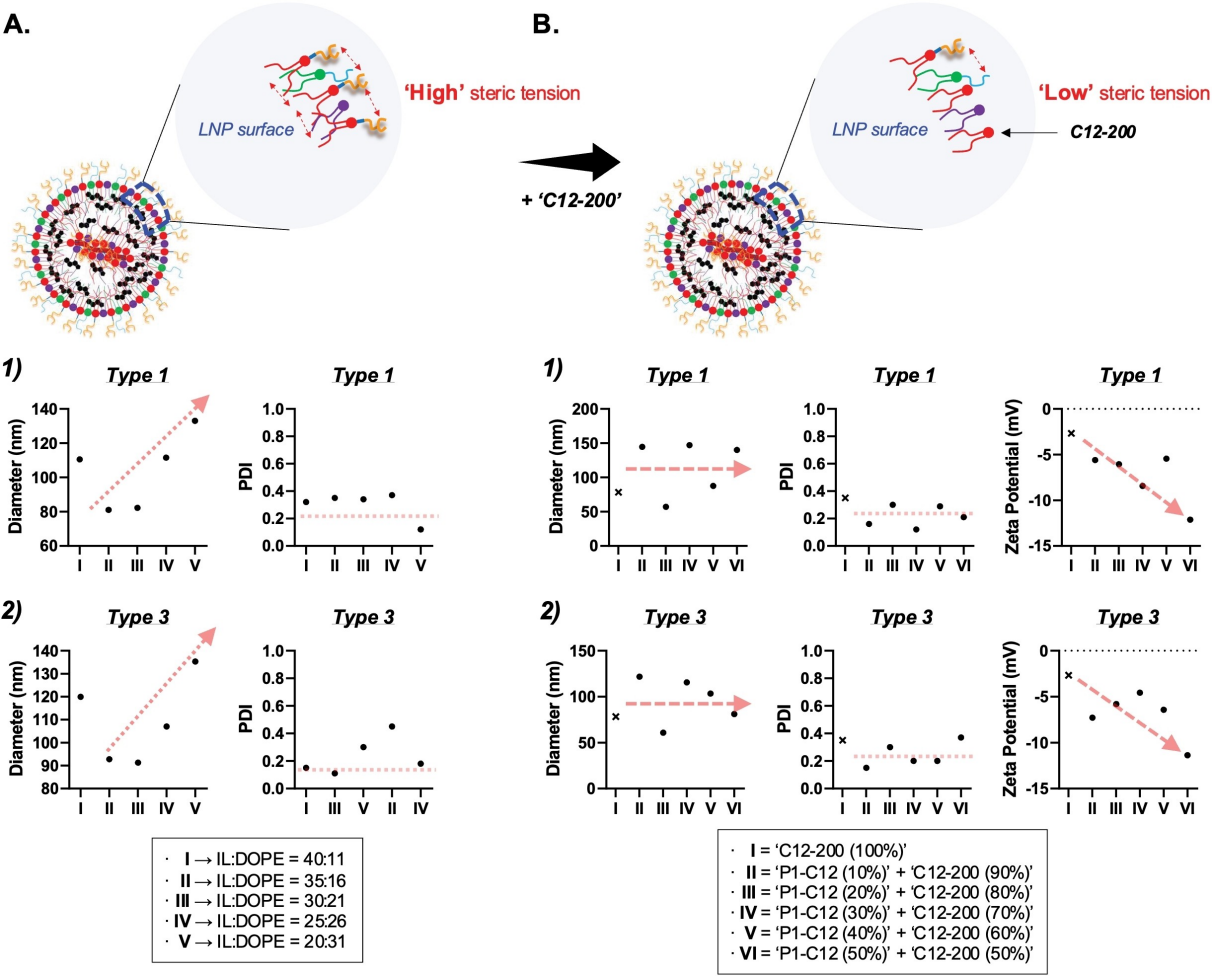
Changes in physicochemical properties of piperazine‐based bisphosphonate‐linked (PIP‐BP) LNPs according to variations in the composition ratio of PIP‐BP ionizable lipids. Refer to supplementary Figures S20 and S21 for the results of luciferase expression in in vitro experiments corresponding to these changes. (a) LNPs with 100 % PIP‐BP ionizable lipids, along with cholesterol, DOPE, and C14‐PEG2000 lipids. (b) LNPs with PIP‐BP ionizable lipids mixed with C12‐200, cholesterol, DOPE, and C14‐PEG2000 lipids. (a1 and b1) ‘**Type** **1**‐**P1**‐**C12**’+‘**C12**‐**200**’, and (a2 and b2) ‘**Type** **3**‐**P1**‐**C12**’+‘**C12**‐**200**’ were utilized. The PIP‐BP LNPs were formulated with the following ratios: Ionizable lipid(s)/DOPE/Cholesterol/C14‐PEG2000=35/16/46.5/2.5 %.

Solubility of bisphosphonates in solvents or solutions is a major challenge in the manufacturing of these PIP‐BP LNPs, as briefly mentioned earlier. Bisphosphonates typically dissolve only in water. Furthermore, when combining bisphosphonates with hydrophobic organic compounds, they exhibit a solubility profile very different from conventional ones. Therefore, the newly synthesized PIP‐based BP‐linked ionizable lipids did not dissolve in organic solvents but only dissolved in very small quantities in highly polar solvents such as ethanol (Figure S18). In order to address this solubility issue, a small amount of diluted 1 N hydrochloric acid (HCl) was added to protonate the hydroxyl group in the bisphosphonate, thereby increasing its solubility in ethanol solution. This step facilitates the protonation of hydroxyl groups in alendronate, allowing the PIP‐based BP‐linked ionizable lipids to transition from a solid to a liquid phase and enhancing their solubility in ethanol. This led to an average improvement of approximately 20 % in encapsulation efficiency, maintaining an overall average encapsulation efficiency of around 60 %. Another solution to this solubility issue is to use the newly developed PIP‐based BP‐linked ionizable lipids in small quantities. Subsequently, a majority of ionizable lipids with relatively smaller molecular weights, such as ′**C12**‐**200**′ ionizable lipid, can be used with more than half of the bone‐targeting ionizable lipids to manufacture stable bone‐targeting LNPs. The latter approach can reduce the overall size and/or PDI of PIP‐BP LNPs, resulting in stable bone‐targeting LNPs (Figure 5). In this study, both methods, using a small amount of 1 N HCl and adding ′**C12**‐**200**′ ionizable lipid as a fifth lipid component, were appropriately utilized to manufacture PIP‐BP LNPs via lipid formulation.

Next, PIP‐BP LNPs were formulated using ′**C12**‐**200**′ ionizable lipid to reduce their size and improve their stability. The encapsulation efficiency of PIP‐BP LNPs composed solely of PIP‐based BP‐linked ionizable lipids was approximately 60 %, with some PDI deviation observed (Figure S20). In contrast, when ′**C12**‐**200**′ ionizable lipid was used in conjunction with PIP‐based BP‐linked ionizable lipids, the encapsulation efficiency exceeded 85 %, with lower PDI deviation (Figure S21). This indicates that in BP‐LNPs composed solely of PIP‐based BP‐linked ionizable lipids, the high steric tension among the lipids led to decreased stability of the LNPs, resulting in reduced mRNA encapsulation.

Interestingly, higher luciferase transfection was observed with LNPs formulated with a lower fraction of PIP‐BP lipids, consistent with the influence of steric tension among lipids on LNP stability (Figure 5a). Additionally, when the relatively larger molecule ′**Type** **3**‐**P1**‐**C12**′ was used in lower quantities, mRNA encapsulation decreased (Figure 5b), likely because the ionizable lipid forms relatively more well‐packed LNPs through low steric interactions with surrounding lipids. This influence of lipids′ packing on the overall structure of LNPs was also evident from the hydrodynamic size distribution of these PIP‐BP LNPs. The hydrodynamic size of PIP‐BP LNPs mixed with ′**C12**‐**200**′ ionizable lipid, such as ′**Type** **1**‐**P1**‐**C12**′ or ′**Type** **3**‐**P1**‐**C12**′, became smaller or exhibited a more uniform polydisperse index distribution, indicating an understanding of the impact of steric interactions among lipids on LNPs morphology (Figure S22). This concept, correlated with simulations in Figure 4 and Figure S38 and experiments in Figure S23, explains why PIP‐BP ionizable lipids mixed with ′**C12**‐**200**′ ionizable lipids were adopted for these in vivo experiments. The acyl PIP scaffold possesses a 6‐membered ring, which strongly binds and maintains the structure of BP on the exterior of LNPs. To demonstrate this, we conducted experiments using AFM to measure the binding to hydroxyapatite, a component of bone.

### Binding Study of PIP‐BP LNPs to Hydroxyapatite

**Figure 6:**
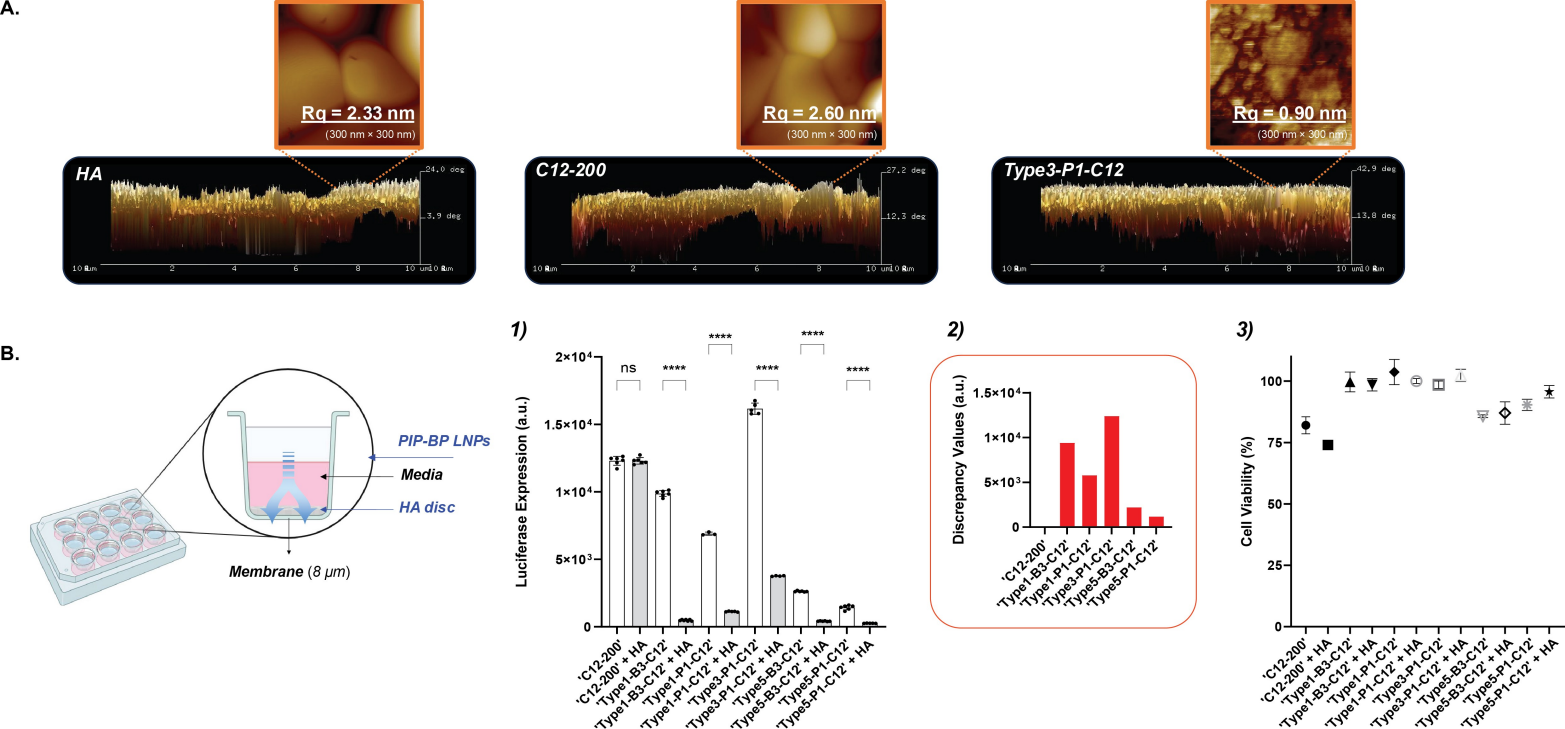
Binding study on hydroxyapatite (HA) using the PIP‐BP LNPs. (a) Analytical results of AFM measurements with PIP‐BP LNPs composed of 100 % bone‐targeting ionizable lipids, DOPE, cholesterol, and C14‐PEG2000 lipids on the HA surface. (b) Binding study of PIP‐BP LNPs to the HA surface in the BJ cell line. Luciferase expression in the BJ cells. 5.000 cells per well were treated with 10 ng of FLuc mRNA. The BJ cells were seeded on the bottom surface of the well plate and the HA surface for 24 hours. The PIP‐BP LNPs were dosed into the HA surface in the chamber, and luciferase expression was measured in BJ cells on the bottom surface of the well plate. The luciferase expression in BJ cells on the bottom surface of the well plate was measured, after incubating the PIP‐BP LNPs on HA discs for 24 hours. The number of replicates for this binding study was more than five, and each experiment was repeated at least three times. The discrepancy values represent the differences in luciferase expression between ′LNPs′ and ′LNPs+HA′. The PIP‐BP LNPs were formulated with the following ratios: Ionizable lipid(s)/DOPE/Cholesterol/C14‐PEG2000=35/16/46.5/2.5 %. ****p<0.0001, and ns=not significant. Ordinary one‐way ANOVA with Tukey's multiple comparisons test, *n*=6. Error bars represent SEM.

**Figure 7:**
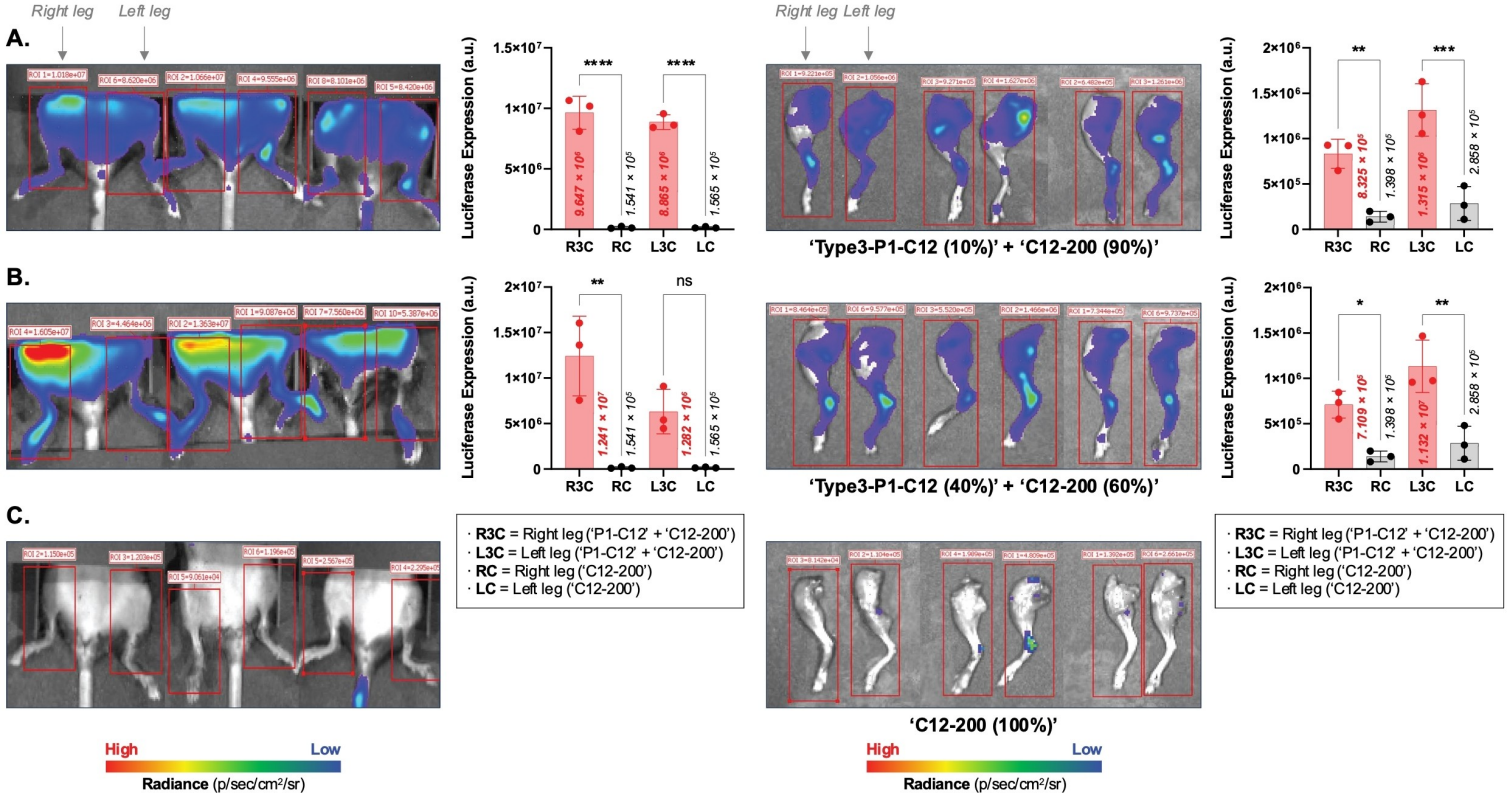
Bioluminescence results in vivo and ex vivo. PIP‐BP LNPs are composed of (a) 10 % PIP‐BP and 90 % ‘**C12**‐**200**’ ionizable lipids or (b) 40 % PIP‐BP and 60 % ‘**C12**‐**200**’ ionizable lipids. (c) LNPs are composed of 100 % ‘**C12**‐**200**’ ionizable lipids. Each mouse received an intravenous injection of 0.5 mg mRNA/kg, and bioluminescence intensity was measured after 6 hours. The number of replicates was three, with the experiment repeated three times. The PIP‐BP LNPs were formulated with the following ratios: Ionizable lipid (s)/DOPE/Cholesterol/C14‐PEG2000=35/16/46.5/2.5 %. ****p<0.0001, ***p<0.001, **p<0.01, *p<0.05, and ns=not significant. Ordinary one‐way ANOVA with Tukey's multiple comparisons test, *n*=3 per group. Error bars represent SEM.

As evident from Figure 4 and Figure S38, the BP group maintains a rigid structure on the surface of PIP‐BP LNPs through PIP. Next, we aimed to experimentally demonstrate the interaction between LNPs and HA visually through confocal microscopy and AFM to confirm whether bone‐targeting LNPs effectively bind to the components of bone materials, such as HA (Figures 6, 7, and S23). Commercially available HA discs were activated by washing them with deionized water and sonication at least three times. HA surfaces were treated with either negative control LNPs based on **′C12**‐**200′** ionizable lipid or PIP‐BP LNPs based on **′Type** **3‐P1**‐**C12′** ionizable lipid. After 24 hours, the surfaces were thoroughly rinsed with deionized water approximately five times to remove any unbound LNPs. The HA surfaces were then imaged using a laser‐scanning confocal microscope. The BP group exhibited absorbance at 488 nm in the photodiode array (PDA) HPLC detector, as shown in Figure S19. Similarly, absorption occurred at 488 nm in the laser scanning confocal microscope images (Figure S23a). In contrast, the HA surfaces coated with LNPs based on **′C12**‐**200′** ionizable lipid, the negative control group, showed no absorbance after thorough washing with deionized water.

As depicted in Figure 6a, the surface of the HA disc was analyzed using AFM. The HA surface coated with LNPs based on **′C12**‐**200′** ionizable lipid exhibited a surface roughness similar to that of the HA surface without any LNP deposition. However, the HA surface coated with the PIP‐BP LNPs, **′Type** **3**‐**P1**‐**C12′** ionizable lipid, exhibited smoother surface roughness. The roughness values for the HA surface without any PIP‐BP LNP deposition and the HA surface coated with LNPs based on **′C12**‐**200′** ionizable lipid were 2.33 nm and 2.60 nm, respectively. In contrast, the HA surface coated with **′Type** **3**‐**P1**‐**C12′** ionizable lipid exhibited a roughness value of 0.90 nm, indicating that the HA disc surface with rough features was coated PIP‐BP LNP structures.

PIP‐BP LNPs were labeled with fluorescent dyes to further investigate their adsorption onto HA surfaces. After manufacturing the LNPs, fluorescent dyes such as DiO or DiD were mixed to allow the dyes to integrate into the LNPs through hydrophobic interactions, which maintained consistent lipid molar ratios. DiO or DiD mixed with LNPs of five types (**′Type** **1**‐**P1**‐**C12′**, **′Type** **3**‐**P1**‐**C12′**, **′Type** **1**‐**P1**‐**C12′**+**′C12**‐**200′**, **′Type** **3**‐**P1**‐**C12′**+**′C12**‐**200′**, and **′C12**‐**200′** ionizable lipid‐based LNPs) were coated onto the HA surface. After 24 hours, the HA surface was thoroughly rinsed approximately five times with deionized water and analyzed using laser scanning confocal microscopy. The results showed consistent findings as mentioned earlier (Figures S23.b2 and S23.b3): LNPs with PIP‐BP ionizable lipids adsorbed onto the HA surface, whereas conventional LNPs based on **′C12**‐**200′** ionizable lipid adsorbed less onto the HA surface. As mentioned in Figure 5, **′PIP**‐**BP LNPs′** with a loose structure exhibited better interaction with the relatively small hindrance factor DiO dye. Conversely, **′PIP**‐**BP LNPs with C12**‐**200′**, which had a relatively compact structure, showed better interaction with the larger‐sized and hook‐shaped DiD dye. These experimental results further indirectly indicated that **′PIP**‐**BP LNPs with C12**‐**200′** tended to have a more compact LNP morphology.

Next, we investigated whether PIP‐BP LNPs binding to HA impacts in vitro transfection (Figure 6b). Six types of PIP‐BP LNPs were used, including five PIP‐BP LNPs that showed top performance in the BJ cell line and one negative control group based on ′**C12**‐**200**′ ionizable lipid‐based LNPs. Chambers containing HA discs and 12‐well plates with BJ cells were incubated for approximately 24 hours. Subsequently, the six types of PIP‐BP LNPs were added to the chambers and incubated for another 24 hours. After 24 hours, the chambers containing the HA discs were removed, and the luciferase expression of the bone‐targeting LNPs within the BJ cells attached to the bottom of the 12‐well plates was measured using a microplate reader. Most of the ′**C12**‐**200**′ ionizable lipid‐based LNPs were able to transport across the HA disc and transwell membrane, resulting in increased cellular uptake by the attached BJ cells on the bottom and higher luciferase expression. However, PIP‐BP LNPs treated in wells containing HA discs resulted in low luciferase expression. Reduced luminescence indicated the PIP‐BP LNPs remained bound to HA and the fraction of freely soluble PIP‐BP LNPs was significantly lower compared to control ‘**C12**‐**200**’ LNPs. Therefore, the binding of PIP‐BP LNPs to HA likely controls the release and delivery of mRNA to cells. This result also suggests that control of local delivery may prolong transfection in vivo and prevent rapid clearance of LNPs. As mentioned in Figure 1, PIP‐BP LNPs of different types have different shapes, so the degree of adhesion to the HA surface varies. However, all PIP‐BP LNPs used in the experiment interacted strongly with the HA surface. Furthermore, as previously mentioned, compared to ′**C12**‐**200**′ ionizable lipid‐based LNPs, all PIP‐BP LNPs showed high cell viability, indicating no cell toxicity issues.

### PIP‐BP LNP Mediated In vivo mRNA Delivery to Bone

After demonstrating the in vitro efficacy of PIP‐BP LNPs for bone targeting, we further evaluated whether PIP‐BP LNPs enhance mRNA delivery in vivo using ′**Type** **1**‐**P1**‐**C12**′ and ′**Type** **3**‐**P1**‐**C12**′ bone‐targeting ionizable lipids, which showed the highest transfection in the Hep‐G2 and BJ cell lines. Prior to performing in vivo experiments, cell toxicity analysis and dose‐dependent transfection studies were conducted for these two different PIP‐BP ionizable lipids in vitro to investigate whether these two lipids could safely and effectively deliver mRNA in in vivo environments. The results of these experiments, as shown in Figures S30 and S31, revealed no significant cell toxicity, and successful cell transfection was achieved even with increased mRNA dosage for the PIP‐BP LNPs. Based on this series of research findings, we proceeded with the in vivo experiments. Each C57BJ/6J mouse was intravenously injected with an mRNA dosage of 0.5 mg/kg.

It was necessary to enhance the encapsulation efficiency and stability of PIP‐BP LNPs to effectively deliver mRNA to bone cells. Therefore, as mentioned earlier, we aimed to add ′**C12**‐**200**′ ionizable lipid as a fifth lipid component to reduce the size and achieve uniformity in LNPs. Preliminary studies determined the optimal ratio of ′**C12**‐**200**′ ionizable lipid to one of the PIP‐BP ionizable lipids. However, rather than increasing the proportion of relatively large PIP‐BP ionizable lipids compared to ′**C12**‐**200**′ ionizable lipid, we limited the usage of a PIP‐BP ionizable lipid to a maximum of 50 % relative to ′**C12**‐**200**′ ionizable lipid to create relatively stable bone‐targeting LNPs. It was observed that bone‐targeting LNPs generated by a harmonized ratio of 10 % ′**Type** **3**‐**P1**‐**C12**′ PIP‐BP lipid and 90 % ′**C12**‐**200**′ ionizable lipid strongly bound to the surrounding cells near the bone. These in vivo experimental results (Figures 7a and 7b) closely resembled the findings of the in vitro experiments (Figures 1d and Figure S29). LNPs based on ′**Type** **3**‐**P1**‐**C12**′ mixed with ′**C12**‐**200**′ ionizable lipid exhibited high luciferase expression in vivo. This could be attributed to the chemical structure of ′**Type** **3**′, which facilitates binding with mRNA due to multiple amine cores and tails, as well as advantages in endocytosis based on electrostatic interactions.

Bone‐targeting LNPs based on ′**Type** **3**‐**P1**‐**C12**′ PIP‐BP lipid exhibited higher transfection compared to LNPs based on ′**Type** **1**‐**P1**‐**C12**′ PIP‐BP lipid (Figure S33). Furthermore, it was observed that bone‐targeting LNPs generated by a harmonized ratio of 10 % ′**Type** **3**‐**P1**‐**C12**′ PIP‐BP lipid and 90 % ′**C12**‐**200**′ ionizable lipid demonstrated the most efficient transfection. In order to examine protein expression in cells when excess PIP‐BP ionizable lipid was used, we increased the proportion of ′**Type** **3**‐**P1**‐**C12**′ PIP‐BP lipid relative to ′**C12**‐**200**′ ionizable lipid up to 50 % and observed its effect on transfection. The results showed that PIP‐BP LNPs generated by a harmonized ratio of 40 % ′**Type** **3**‐**P1**‐**C12**′ PIP‐BP lipid and 60 % ′**C12**‐**200**′ ionizable lipid exhibited the strongest luciferase expression (Figure S34). To the best of our knowledge, this phenomenon suggests that although it may take some time for PIP‐BP LNPs to enter the cells around the bone, they gradually accumulate in the adjacent bone cells.

In Figure 1 and Figures S33–S34, it was confirmed that PIP‐BP LNPs bind to the bones of mice. An in vivo imaging system (IVIS) was used for mice whole‐body imaging and imaging of the leg portions after sacrificing the mice, as shown in Figure 7. The PIP‐BP LNPs generated by the ratio of 10 % ′**Type** **3**‐**P1**‐**C12**′ PIP‐BP lipid and 90 % ′**C12**‐**200**′ ionizable lipid, as well as the ratio of 40 % ′**Type** **3**‐**P1**‐**C12**′ PIP‐BP lipid and 60 % ′**C12**‐**200**′ ionizable lipid, which exhibited the highest luciferase expression, were used to encapsulate FLuc mRNA for systemic administration. Through dissection of the leg portions of the mice, particularly in the femur, patella, and medial fabella, strong luciferase expression was observed. Thus, it was evident from the in vivo experiments that the newly developed PIP‐BP LNPs were delivered to bone tissues.

**Figure 8:**
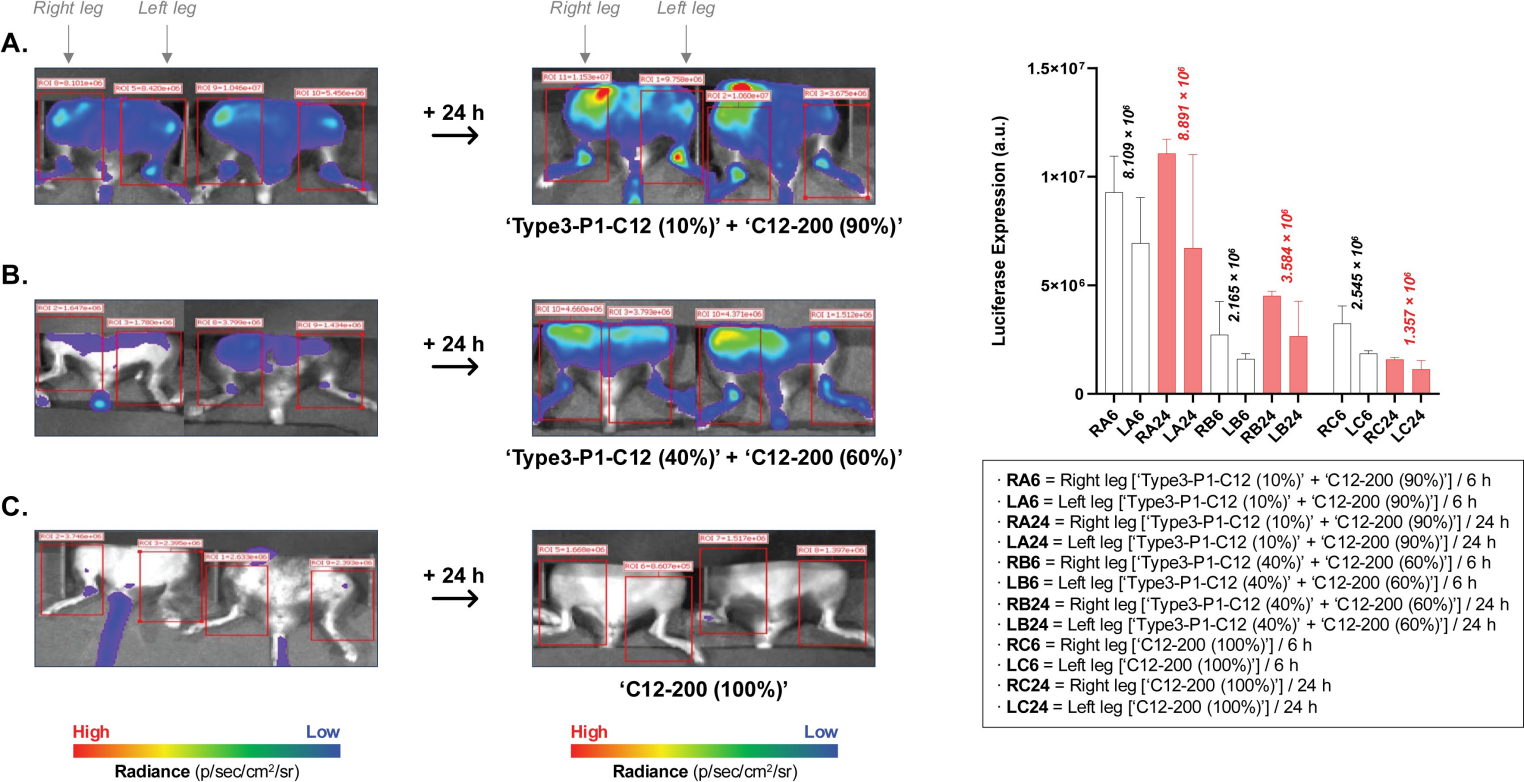
Time‐dependent bioluminescence expression of PIP‐BP LNPs are composed of (a) 10 % PIP‐BP and 90 % ‘**C12**‐**200**’ ionizable lipids or (b) 40 % PIP‐BP and 60 % ‘**C12**‐**200**’ ionizable lipids. (c) LNPs are composed of 100 % ‘**C12**‐**200**’ ionizable lipids. The bone‐targeting LNPs were formulated with the following ratios: Ionizable lipid(s)/DOPE/Cholesterol/C14‐PEG2000=35/16/46.5/2.5 %. Error bars represent SEM.

To investigate the accumulation of PIP‐BP LNPs in bones over time, we injected the mRNA PIP‐BP LNPs generated by the ratio of 10 % ′**Type** **3**‐**P1**‐**C12**′ PIP‐BP lipid and 90 % ′**C12**‐**200**′ ionizable lipid, as well as the ratio of 40 % ′**Type** **3**‐**P1**‐**C12**′ PIP‐BP lipid and 60 % ′**C12**‐**200**′ ionizable lipid into mice and observed changes in luciferase expression over time (Figure 8). Initially, although weak luminescence intensity was observed, over time, strong luciferase expression was evident in the major bone regions, indicating the accumulation of bone‐targeting LNPs in these areas. In contrast, ′**C12**‐**200**′ ionizable lipid‐based LNPs showed a decrease or little change in luminescence intensity over time. Together these results suggest PIP‐BP LNPs facilitate targeting to the bone microenvironment as well as retention and transfection of cells in vivo over time.

## Conclusions

Bone‐targeting ionizable lipids (PIP‐BP) were synthesized with a rigid backbone of piperazine and amides. LNP formulations composed of various types of PIP‐BP ionizable lipids enhanced our understanding of the chemical structures of bone‐targeting LNPs and their strong interaction with hydroxyapatite surfaces. The synthesis of the various forms of PIP‐BP ionizable lipids were derived from economically viable starting materials such as 1‐boc‐piperazine. All 140 types of PIP‐BP LNPs exhibited consistent negative surface charges and anticipated morphologies. Further, these PIP‐BP LNPs can be immobilized on a hydroxyapatite surface, which enables three‐dimensional shape analysis using analytical equipment such as AFM, QCM−D, and cryo‐TEM. Altering the molar ratios among the lipids in this LNP formulation enabled the manufacturing of LNPs with tunable physicochemical properties. The PIP‐BP lipid ′**Type** **3**‐**P1**‐**C12**′ exhibited very high luciferase expression in both in vitro and in vivo experiments, with significantly lower cytotoxicity compared to LNPs based on ′**C12**‐**200**′ ionizable lipid. The synthesized capping ligands are capable of immediate chemical bonding with numerous forms of amines. This study paves the way for the development of efficient ionizable lipids based on piperazine‐linkages for new nucleic acid and gene therapies.

## Contributions

I.‐C. Y., L. X., K. H. V., and M. J. M. contributed to the experimental design and analyzed the data. I.‐C. Y. designed, synthesized, and characterized the chemical structures of ionizable lipids and bone‐targeting LNPs. I.‐C. Y., L. X., Q. C., J. L., J. X., Z. S., D. K., B. C., Q. S., E. L. H., and M. C. R. performed the biology and validation experiments. I.‐C. Y. wrote and edited the manuscript and Supporting Information. All the authors discussed the experimental results.

## Conflict of Interests

ICY, LX, KV, MJM have filed a patent on the lipid nanoparticle technology described in this work. The other authors declare no competing interests.

1

## Supporting information

As a service to our authors and readers, this journal provides supporting information supplied by the authors. Such materials are peer reviewed and may be re‐organized for online delivery, but are not copy‐edited or typeset. Technical support issues arising from supporting information (other than missing files) should be addressed to the authors.

Supporting Information

## Data Availability

The data that support the findings of this study are available in the supplementary material of this article.
