# Supplementary material for: Piperazine‐Derived Bisphosphonate‐Based Ionizable Lipid Nanoparticles Enhance mRNA Delivery to the Bone Microenvironment
Source: Angew Chem Int Ed Engl. 2024 Dec 13;64(3):e202415389. doi: 10.1002/anie.202415389 (PMC11735871; doi:10.1002/anie.202415389)
Supplement: Supplementary file 1 — Supporting Information [file ANIE-64-e202415389-s001.pdf]

Angewandte Chemie
Eine Zeitschrift der Gesellschaft Deutscher Chemiker

Supporting Information

Piperazine-Derived Bisphosphonate-Based Ionizable Lipid Nanoparticles Enhance mRNA Delivery to the Bone Microenvironment

I.-C. Yoon, L. Xue, Q. Chen, J. Liu, J. Xu, Z. Siddiqui, D. Kim, B. Chen, Q. Shi, E. Laura Han, M. Cherry Ruiz, K. H. Vining, M. J. Mitchell**

Supporting Information

Piperazine-Derived Bisphosphonate-Based Ionizable Lipid Nanoparticles Enhance mRNA Delivery to the Bone Microenvironment

Il-Chul Yoon[1,2,3], Lulu Xue[1], Qinyuan Chen[3], Jingyi Liu[1,3], Junchao Xu[1], Zain Siddiqui[1,3], Dongyoon Kim[1], Bingling Chen[3], Qiangqiang Shi[1], Emily Laura Han[1], Mia Cherry Ruiz[3], Kyle H. Vining[2,3*], and Michael J. Mitchell[1,4,5,6,7,8,9*]

[1]Department of Bioengineering, School of Engineering and Applied Science, University of Pennsylvania, Philadelphia, PA 19104, United States

[2]Department of Materials Science and Engineering, School of Engineering and Applied Science, University of Pennsylvania, Philadelphia, PA 19104, United States

[3]Preventive and Restorative Sciences, School of Dental Medicine, University of Pennsylvania, Philadelphia, PA 19104, United States

[4]Abramson Cancer Center, Perelman School of Medicine, University of Pennsylvania, Philadelphia, PA 19104, United States

[5]Center for Cellular Immunotherapies, Perelman School of Medicine, University of Pennsylvania, Philadelphia, PA 19104, United States

[6]Penn Institute for RNA Innovation, Perelman School of Medicine, University of Pennsylvania, Philadelphia, PA 19104, United States

[7]Institute for Immunology, Perelman School of Medicine, University of Pennsylvania, Philadelphia, PA 19104, United States

[8]Cardiovascular Institute, Perelman School of Medicine, University of Pennsylvania, Philadelphia, PA 19104, United States

[9]Institute for Regenerative Medicine, Perelman School of Medicine, University of Pennsylvania, Philadelphia, PA 19104, United States

* Corresponding authors: viningk@seas.upenn.edu; mjmitch@seas.upenn.edu

Table of contents

1. Materials S1

1.1. Chemical reagents S1

1.2. Biological assays S1

1.3. Cell culture S2

1.4. Animal studies S2

2. Instruments and methods S2

3. Synthesis S3

3.1. Overall synthetic pathway S3

3.2. Product 1 (*tert*-Butyl 4-acryloylpiperazine-1-carboxylate) S4

3.3. Product 2 (4-((3-(4-(*tert*-Butoxycarbonyl)piperazin-1-yl)-3-oxopropyl)amino)-1-hydroxybutane-1,1-diyl)bis(phosphonic acid) S4

3.4. Product 3 (4-(*N*-(3-(4-(*tert*-Butoxycarbonyl)piperazin-1-yl)-3-oxopropyl)-2-chloroacetamido)-1-hydroxybutane-1,1-diyl)bis(phosphonic acid) S5

3.5. Product 4 (4-(2-Chloro-*N*-(3-(4-(2-chloroacetyl)piperazin-1-yl)-3-oxopropyl)acetamido)-1-hydroxybutane-1,1-diyl)bis(phosphonic acid) S7

3.6. Product 5 (*tert*-Butyl 4-(4-((2,5-dioxopyrrolidin-1-yl)oxy)-4-oxobutanoyl)piperazine-1-carboxylate) S8

3.7. Product 6 (4-(4-(4-(*tert*-Butoxycarbonyl)piperazin-1-yl)-4-oxobutanamido)-1-hydroxybutane-1,1-diyl)bis(phosphonic acid) S9

3.8. Product 7 (4-(4-(4-(2-Chloroacetyl)piperazin-1-yl)-4-oxobutanamido)-1-hydroxybutane-1,1-diyl)bis(phosphonic acid) S10

3.9. Synthetic protocol for the bone-targeting ionizable lipids 'Type 1' S12

3.10. Synthetic protocol for the bone-targeting ionizable lipids 'Type 2' S14

3.11. Synthetic protocol for the bone-targeting ionizable lipids 'Type 3' S17

3.12. Synthetic protocol for the bone-targeting ionizable lipids 'Type 4' S19

3.13. Synthetic protocol for the bone-targeting ionizable lipids 'Type 5' S22

3.14. Structural characterization of 'Type1-P1-C12' S24

3.15. Structural characterization of 'Type3-P1-C12' S26

4. Biological assays S28
4.1. LNP formulation S28
4.2. FLuc mRNA *in vitro* delivery screening S29
4.3. Characterization of bone-targeted LNPs-HA interaction *in vitro* S30
4.4. FLuc mRNA *in vivo* delivery screening S30

5. Supplementary figures S30
5.1. Cryo-TEM analysis S30
5.2. Solubility testing of compounds linked with alendronate S31
5.3. LC-UV diode-array detection for alendronate S32
5.4. Effects of LNP formulation changes on *in vitro* experimental results S32
5.5. Impact of 'C12-200' on the morphology of bone-targeted LNPs S33
5.6. Bone-targeted LNPs adsorption on hydroxyapatite experiments S34
5.7. Hep-G2 cell line *in vitro* screening results S36
5.8. BJ cell line *in vitro* screening results S40
5.9. mRNA dose-dependent transfection experiments S41
5.10. Comparative study between previous and current bone-targeting LNPs S41
5.11. *In vivo* bioluminescence imaging S42
5.12. *Ex vivo* bioluminescence imaging S43
5.13. H&E-stained histological images of mouse leg bone tissue S44
5.14. H&E-stained histological images of mouse organ tissue S44

6. Computed structure coordinates S45
6.1. Overall computational results S45
6.2. Previous branched amine core based bone-targeting ionizable lipids S45
6.3. Previous piperazine amine core based bone-targeting ionizable lipids S62
6.4. Branched amine core based bone-targeting ionizable lipids 'Type 1' S78
6.5. Piperazine amine core based bone-targeting ionizable lipids 'Type 1' S96
6.6. Branched amine core based bone-targeting ionizable lipids 'Type 2' S114
6.7. Piperazine amine core based bone-targeting ionizable lipids 'Type 2' S132
6.8. Branched amine core based bone-targeting ionizable lipids 'Type 3' S149

6.9. Piperazine amine core based bone-targeting ionizable lipids 'Type 3' S173
6.10. Branched amine core based bone-targeting ionizable lipids 'Type 4' S196
6.11. Piperazine amine core based bone-targeting ionizable lipids 'Type 4' S212
6.12. Branched amine core based bone-targeting ionizable lipids 'Type 5' S228
6.13. Piperazine amine core based bone-targeting ionizable lipids 'Type 5' S245

7. References S262

1. Materials

1.1. Chemical reagents

1-Boc-piperazine (98%) was purchased from Oakwood Chemical. Acryloyl chloride (98%), chloroacetyl chloride (97%), succinyl chloride (95%), *N*-hydroxysuccinimide (98%), triethylamine (≥ 99%), dichloromethane (≥ 99.8%), ethyl acetate (ACS reagent, ≥ 99.5%), *n*-hexane (≥ 95%), and cholesterol (≥ 99%) were purchased from Sigma-Aldrich. Alendronate sodium trihydrate (98%), 1,2-epoxyhexadecane (C16, 98%), and *N*1-(2-(4-(2-aminoethyl)piperazin-1-yl)ethyl)ethane-1,2-diamine were purchased from AmBeed Chemical. Potassium carbonate (anhydrous, 99%), sodium hydroxide (white pellets), Benzoxazolium, 3-octadecyl-2-[3-(3-octadecyl-2(3*H*)-benzoxazolylidene)-1-propenyl]-, perchlorate (DiO, 97% at 490 nm), and 1,1'-Dioctadecyl-3,3,3',3'-tetramethylindodicarbocyanine (DiD, 99% at 640 nm) were purchased from ThermoFisher Scientific. *N*,*N*-Diisopropylethylamine (≥ 99.5%) was purchased from Acros Organics Chemicals. Trifluoroacetic acid (≥ 99.0%), acetonitrile (≥ 99.8%), and tris(2-aminoethyl)amine (B1, 97%) were purchased from Alfa Aesar Chemicals. Ethyl alcohol (200 proof, 100%) was purchased from Decon Labs. 1,2-Epoxydecane (C10, ≥ 97.0%), 1,2-epoxydodecane (C12, ≥ 95.0%), 1,2-epoxytetradecane (C14, ≥ 95.0%), and tris(3-aminopropyl)amine (B3, 97%) were purchased from TCI Chemicals. Bis(3-aminopropyl)({4-[bis(3-aminopropyl)amino]butyl})amine (B2, 95%), 2-(4-{2-[(2-aminoethyl)amino]ethyl}piperazin-1-yl)ethan-1-amine (P1, 95%), 3-(4-{2-[(3-amino-2-ethoxypropyl)amino]ethyl}piperazin-1-yl)-2-ethoxypropan-1-amine (P2, 95%), 2-{2-[4-(2-{[2-(2-aminoethoxy)ethyl]amino}ethyl)piperazin-1-yl]ethoxy}ethan-1-amine (P3, 95%), and {2-[2-(2-aminoethoxy)ethoxy]ethyl}[2-(4-{2-[2-(2-aminoethoxy)ethoxy]ethyl}piperazin-1-yl)ethyl]amine (P4, 95%) were purchased from Enamine. 1,2-dioleoyl-sn-glycero-3-phosphoethanolamine (DOPE, > 99%) and 1,2-dimyristoyl-sn-glycero-3-phosphoethanolamine-N-[methoxy(polyethyleneglycol)-2000] (C12-PEG2000, > 99%) were purchased from Avanti Polar Lipids. Hydroxyapatite disc was purchased from Clarkson Chromatography Products. Deuterium oxide (D_2O, 99.8%), dimethyl sulfoxide-*d6* (DMSO-*d6*, 99.8%), and chloroform-*d* ($CDCl_3$) were purchased from Acros Organics Chemicals. All the chemicals were used without further purification.

1.2. Biological assays

Firefly Luciferase (FLuc) mRNA (5-methoxyuridine) (Ref. L-7202) was purchased from TriLink Biotechnologies. Quant-it™ RiboGreen™ RNA reagent and kit were purchased from ThermoFisher Scientific. Luciferase 1000 assay system (Ref. E4550) and CellTiter-Glo luminescent cell viability (Ref. G7572) were purchased from Promega Corporation.

1.3. Cell culture

Dulbecco's Modified Eagle Medium (DMEM) with high glucose, *L*-glutamine, phenol red, and without sodium pyruvate and HEPES was purchased from Gibco Scientific. Trypsin-EDTA (0.25%) and penicillin-streptomycin (P/S) were purchased from Gibco Scientific. Fetal bovine serum (FBS) was purchased from Sigma-Aldrich. Hep-G2 cells were cultured in DMEM supplemented with 10% FBS and 1% P/S. BJ cells were cultured in DMEM supplemented with 10% FBS.

1.4. Animal studies

C57BL/6J mice were purchased from Jackson Laboratory. All experimental procedures were approved by the Institutional Animal Care & Use Committee (IACUC) of the University of Pennsylvania (Protocol number: 806540) and complied with relevant local, state, and federal regulations.

2. Instruments and methods

FT-IR spectra were recorded with a Thermo Scientific™ Nicolet™ iS™ 5 FT-IR spectrometer, equipped with an iD7 ATR diamond. ^{1}H and ^{13}C NMR spectra were acquired using a Bruker NEO NMR spectrometer at 400 MHz and 101 MHz (or 600 MHz and 125 MHz), respectively. ^{31}P NMR spectra were also acquired using the same instrument operating at 162 MHz (or 243 MHz). All the NMR measurements were conducted at room temperature in D_2O, DMSO-*d6*, or $CDCl_3$. NMR data were processed using MNova 14 software. Chemical shifts (δ) are reported in parts per million (ppm). The resonance patterns in the 1H NMR spectra are denoted as "s" (singlet), "d" (doublet), "t" (triplet), "q" (quartet), "m" (multiplet), and "br" (broad resonance). The residual protic solvent of D_2O (^{1}H, δ 4.79 ppm), DMSO-*d6* (^{1}H, δ 2.50 ppm; ^{13}C, δ 39.52 ppm), $CDCl_3$ (^{1}H, δ 7.26 ppm; ^{13}C, δ 77.16 ppm) and tetramethylsilane (TMS, δ 0 ppm) served as the internal reference for ^{1}H or ^{13}C NMR spectra. The structural frameworks of all compounds were conclusively determined by two-dimensional nuclear magnetic resonance spectroscopy (2D NMR), including ^{1}H-^{1}H COSY[1,2], ^{1}H-^{13}C HSQC[1,2], and ^{1}H-^{13}C HMBC[1,2]. LC-MS analyses were conducted on a Waters™ Chromatography Column equipped with a UV-Vis detector. The analysis was carried out using a solution consisting of a mixture of water (50%) and acetonitrile (50%) at room temperature. Compound detection was performed through UV absorbance at λ = 254 nm. A PerSeptive Biosystem-Voyager-DE MALDI-ToF spectrometer (Framingham, MA) equipped with a nitrogen laser (337 nm) and operating in linear mode was utilized for characterizing the ionizable lipids. Angiotensin II and Bombesin served as reference standards for calibration. In order to prepare the sample solution, each compound was initially dissolved in THF at a concentration of 5−10 mg/mL. Subsequently, a matrix solution of 2,5-dihydroxybenzoic acid was prepared in THF at a concentration of 10 mg/mL, and the two solutions were combined at a ratio of 1/5 (*v/v*, compound solution to matrix solution). LNPs were formulated

using a Pump33DS syringe pump (Harvard Apparatus, Holliston, MA). Particle size and zeta potential were measured by Dynamic Light Scattering (DLS) using a DynaPro™ Plate Reader III or Malvern Zetasizer Nano ZS. Particle morphology was assessed using Cryo-TEM. The AFM images were acquired using a Bruker Dimension Icon AFM operating in Tapping Mode, employing MikroMasch 325 kHz cantilevers. The surface of the HA discs was examined using a confocal laser scanning microscope (Zeiss LSM800) equipped with a 20× water-dipping objective (numerical aperture = 1.0). Sequential scanning was conducted using diode lasers (488 nm for DiO and 640 nm for DiD), and the emitted signal was collected using optimized emission wavelength filter sets. A 405-nm laser in reflection mode was used to visualize the HA surface. Z-stack datasets (0.260-μm pixel size and 0.850 μm z step) were acquired and processed (maximum intensity projection) using ZEN lite software (Zeiss). The DiO fluorescence dye is excited at 490 nm and emits at 506 nm. The DiD fluorescence dye is excited at 649 nm and emits at 668 nm. All computed conformers were generated using the Gaussian 16 software package[3]. Initial chemical structures were optimized using the Universal Force Field (UFF) molecular mechanics method[4], followed by single-point energy calculations[5]. The conformers were analyzed and visualized using the GaussView 6[6] and Jmol[7] software programs.

3. Synthesis

3.1. Overall synthetic pathway

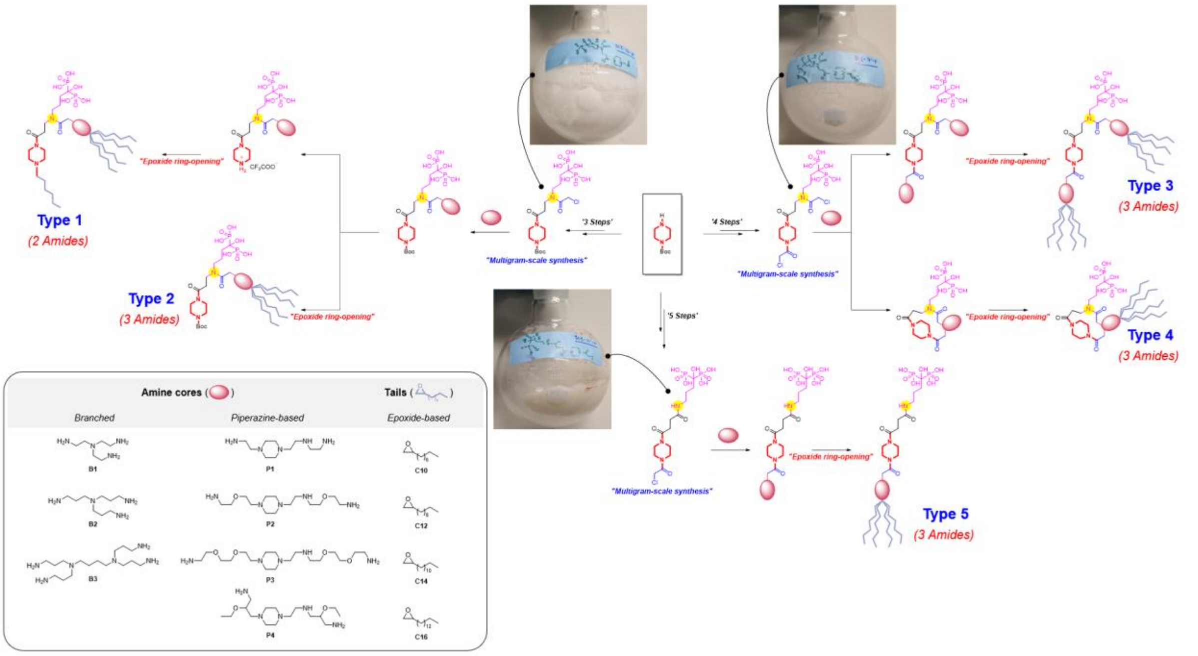

Scheme S1. Synthetic route for the synthesis of bone-targeting ionizable lipids.

3.2. Product 1 (*tert*-Butyl 4-acryloylpiperazine-1-carboxylate)[8]

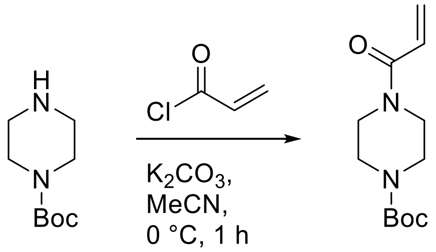

After dissolving 1-boc-piperazine (5 g, 26.84 mmol, 1 eq.) in acetonitrile (50 mL, 0.5 M to '1-boc-piperazine') containing potassium carbonate (11.13 g, 80.53 mmol, 3 eq.), the mixture was stirred rapidly at room temperature for 15 minutes. Subsequently, the reaction mixture was cooled to 0 °C using an ice bath, and acryloyl chloride (2.62 mL, 32.21 mmol, 1.2 eq.) was added dropwise while maintaining the temperature at 0 °C. The reaction mixture was stirred at 0 °C for 1 hour. After filtration to remove potassium carbonate, the solutions of supernatant were completely removed under reduced pressure to give the product (6.19 g, 96%). White solid.

FT-IR (ATR mode): 1682 and 1633 [v(R_2N-($\underline{C=O}$)-)], 1618 [v(-C=C-)], 1251 and 1167 [v(-C-O-)] cm^{-1}.

^{1}H NMR (400 MHz, $CDCl_3$): δ 6.50 (m, 1H, R_2N-(C=O)-C$\underline{H}$=CH_2), 6.23 and 5.66 (dd, 2H, R_2N-(C=O)-CH=C$\underline{H}_2$, *J* = 16.8, 1.9 Hz), 3.70−3.30 (m, 8H, -RN-(CH_2CH_2)$_2$-NR-), 1.41 (s, 9H, -(C=O)-O-C(CH_3)$_3$) ppm.

^{13}C{H} NMR (101 MHz, $CDCl_3$): δ 165.6 (R_2N-($\underline{C}$=O)-CH=CH_2), 154.5 (-($\underline{C}$=O)-O-(CH_3)$_3$), 128.3 (R_2N-(C=O)-$\underline{C}$H=CH_2), 127.3 (R_2N-(C=O)-CH=$\underline{C}H_2$), 80.3 (-(C=O)-O-$\underline{C}$(CH_3)$_3$), 45.5, 43.5, and 41.8 (-RN-(CH_2CH_2)$_2$-NR-), 28.3 (-(C=O)-O-C($\underline{C}H_3$)$_3$) ppm.

MS (ES +ve) *m/z* (abundance %) for $C_{12}H_{20}N_2O_3$: calculated $[M + H]^+$ 241.3068, found $[M + H]^+$ 241.2650 (100).

3.3. Product 2 (4-((3-(4-(*tert*-Butoxycarbonyl)piperazin-1-yl)-3-oxopropyl)amino)-1-hydroxybutane-1,1-diyl)bis(phosphonic acid)[9]

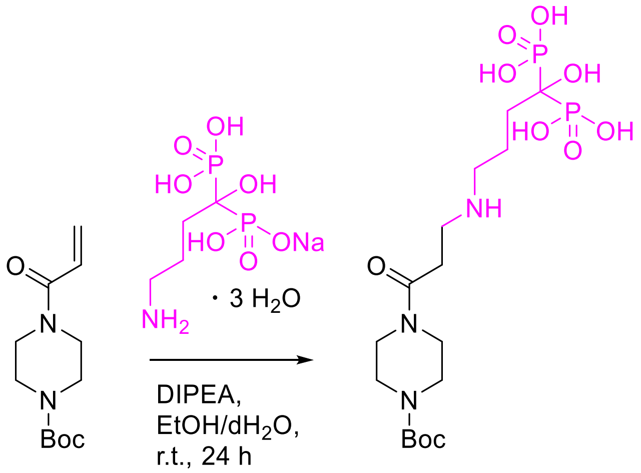

'**Product 1**' (5.32 g, 22.14 mmol, 1.2 eq.) was dissolved in ethanol (184.5 mL, 0.12 M to '**Product 1**') and *N*,*N*-diisopropylethylamine (DIPEA) (9.64 mL, 55.35 mmol, 3 eq.) was added at room temperature. In another vial, alendronate (6 g, 18.45 mmol, 1 eq.) was dissolved in deionized water (dH_2O) (123.0 mL, 0.15 M to alendronate) at room temperature and the resulting solution was added dropwise to the round-bottom flask containing '**Product 1**'. The reaction mixture was stirred at room temperature for 24 hours. The reaction solution was then completely removed under reduced pressure. Subsequently, acetone was added to induce precipitation of '**Product 2**' (8.13 g, 90%), and no additional separations were conducted for the next reactions. White hygroscopic solid. Due to the hydrophobic alkyl chains and bisphosphonate group, the compound exhibits very low solubility in both aqueous and organic solvents.

FT-IR (ATR mode): 2974 [ν(-NH-)], 1692 and 1637 [ν(R_2N-(C=O)-)], 1234 and 1162 [ν(-C-O-)], 1053 [ν(-(P=O)-)], 535 and 453 [ν(-(P-O)-)] cm^{-1}.

^{1}H NMR (400 MHz, D_2O): δ 3.69−3.63 (m, 2H, R_2N-(C=O)-CH_2-CH_2-NHR), 3.61−3.45 (m, 8H, -RN-(CH_2CH_2)$_2$-NR-), 3.40−3.26 (m, 2H, R_2N-(C=O)-CH_2-CH_2-NHR), 3.11−3.03 (m, 2H, -NH-CH_2-CH_2-CH_2-), 2.16−1.93 (m, 4H, -NH-CH_2-CH_2-CH_2-), 1.48 (s, 9H, -(C=O)-O-C(CH_3)$_3$) ppm.

^{13}C{H} NMR (101 MHz, D_2O): δ 170.4 (R_2N-(C=O)-CH_2-CH_2-NHR), 156.3 (-(C=O)-O-C(CH_3)$_3$), 82.2 (-(C=O)-O-C(CH_3)$_3$), 73.5 (-C(OH)-(HPO_3)$_2$), 54.4 (R_2N-(C=O)-CH_2-CH_2-NHR), 45.6, 42.5, and 41.6 (-RN-(CH_2CH_2)$_2$-NR-), 44.9 (R_2N-(C=O)-CH_2-CH_2-NHR), 40.0 (-NH-CH_2-CH_2-CH_2-), 30.7 (-NH-CH_2-CH_2-CH_2-), 27.6 (-(C=O)-O-C(CH_3)$_3$), 22.3 (-NH-CH_2-CH_2-CH_2-) ppm.

^{31}P{H} NMR (162 MHz, D_2O): δ 17.9 and 17.8 ppm.

MS (ES +ve) *m/z* (abundance %) for $C_{16}H_{39}N_3O_{13}P_2$: calculated $[M + H_2O + H]^+$ 562.4640, found $[M + H_2O + H]^+$ 562.7176 (50).

3.4. Product 3 (4-(*N*-(3-(4-(*tert*-Butoxycarbonyl)piperazin-1-yl)-3-oxopropyl)-2-chloroacetamido)-1-hydroxybutane-1,1-diyl)bis(phosphonic acid)

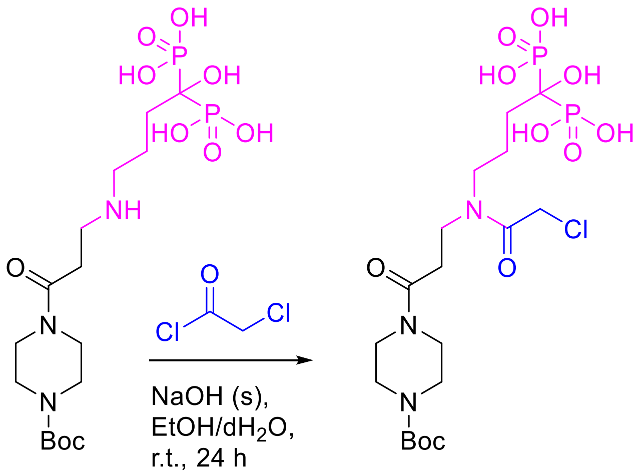

‘**Product 2**’ (4 g, 8.17 mmol, 1 eq.) was dissolved in a solution containing 33% dH_2O and 67% ethyl acetate (EA) (21.5 mL, 0.38 M to ‘**Product 2**’). Subsequently, a finely ground powder of sodium hydroxide (3 eq.) was added, and the mixture was stirred at room temperature for 15 minutes. Chloroacetyl chloride (0.78 mL, 9.80 mmol, 1.2 eq.) was then added dropwise to the reaction solution, which was stirred at room temperature for 24 hours. The reaction solution was completely removed under reduced pressure to give the product (4.3 g, 93%), and no additional separations were conducted for the next reactions. White solid. Due to the hydrophobic alkyl chains and bisphosphonate group, the compound exhibits very low solubility in both aqueous and organic solvents.

FT-IR (ATR mode): 1693 and 1626 [$\nu(R_2N\text{-}(\underline{C{=}O})\text{-})$], 1560 [$\nu(R_2N\text{-}(\underline{C{=}O})\text{-}CH_2\text{-}Cl)$], 1240 and 1166 [$\nu(\text{-}C\text{-}O\text{-})$], 1054 [$\nu(\text{-}(P{=}O)\text{-})$], 647 [$\nu(\text{-}CH_2\text{-}Cl)$], 542 and 463 [$\nu(\text{-}(P\text{-}O)\text{-})$] cm^{-1}.

^1H NMR (400 MHz, D_2O): δ 4.49 (s, 2H, $R_2N\text{-}(C{=}O)\text{-}C\underline{H}_2\text{-}Cl$), 3.68−3.44 (m, 10H, $R_2N\text{-}(C{=}O)\text{-}C\underline{H}_2\text{-}CH_2\text{-}NHR$ and $\text{-}RN\text{-}(CH_2CH_2)_2\text{-}NR\text{-}$), 3.41−3.20 (m, 2H, $R_2N\text{-}(C{=}O)\text{-}CH_2\text{-}C\underline{H}_2\text{-}NHR$), 3.14−3.02 (m, 2H, $\text{-}NH\text{-}C\underline{H}_2\text{-}CH_2\text{-}CH_2\text{-}$), 2.19−1.97 (m, 4H, $\text{-}NH\text{-}CH_2\text{-}C\underline{H}_2\text{-}C\underline{H}_2\text{-}$), 1.50 (s, 9H, $\text{-}(C{=}O)\text{-}O\text{-}C(CH_3)_3$) ppm.

$^{13}C\{H\}$ NMR (101 MHz, D_2O): δ 175.0 ($R_2N\text{-}(\underline{C}{=}O)\text{-}CH_2\text{-}CH_2\text{-}NHR$), 170.5 ($R_2N\text{-}(\underline{C}{=}O)\text{-}CH_2\text{-}Cl$), 156.4 ($\text{-}(\underline{C}{=}O)\text{-}O\text{-}C(CH_3)_3$), 82.3 ($\text{-}(C{=}O)\text{-}O\text{-}\underline{C}(CH_3)_3$), 73.7 ($\text{-}C(OH)\text{-}(HPO_3)_2$), 63.3 ($R_2N\text{-}(C{=}O)\text{-}\underline{C}H_2\text{-}Cl$), 53.2 ($R_2N\text{-}(C{=}O)\text{-}\underline{C}H_2\text{-}CH_2\text{-}NHR$), 49.5, 45.0, and 41.7 ($\text{-}RN\text{-}(CH_2CH_2)_2\text{-}NR\text{-}$), 43.9 ($R_2N\text{-}(C{=}O)\text{-}CH_2\text{-}\underline{C}H_2\text{-}NHR$), 38.1 ($\text{-}NH\text{-}\underline{C}H_2\text{-}CH_2\text{-}CH_2\text{-}$), 30.3 ($\text{-}NH\text{-}CH_2\text{-}\underline{C}H_2\text{-}CH_2\text{-}$), 27.7 ($\text{-}(C{=}O)\text{-}O\text{-}C(\underline{C}H_3)_3$), 20.3 ($\text{-}NH\text{-}CH_2\text{-}CH_2\text{-}\underline{C}H_2\text{-}$) ppm.

$^{31}P\{H\}$ NMR (162 MHz, D_2O): δ 18.4 and 18.0 ppm.

MS (ES +ve) *m/z* (abundance %) for $C_{18}H_{40}ClN_3O_{14}P_2$: calculated $[M + 2Na − H]^+$ 619.9222, found $[M + 2Na − H]^+$ 665.0854 (15).

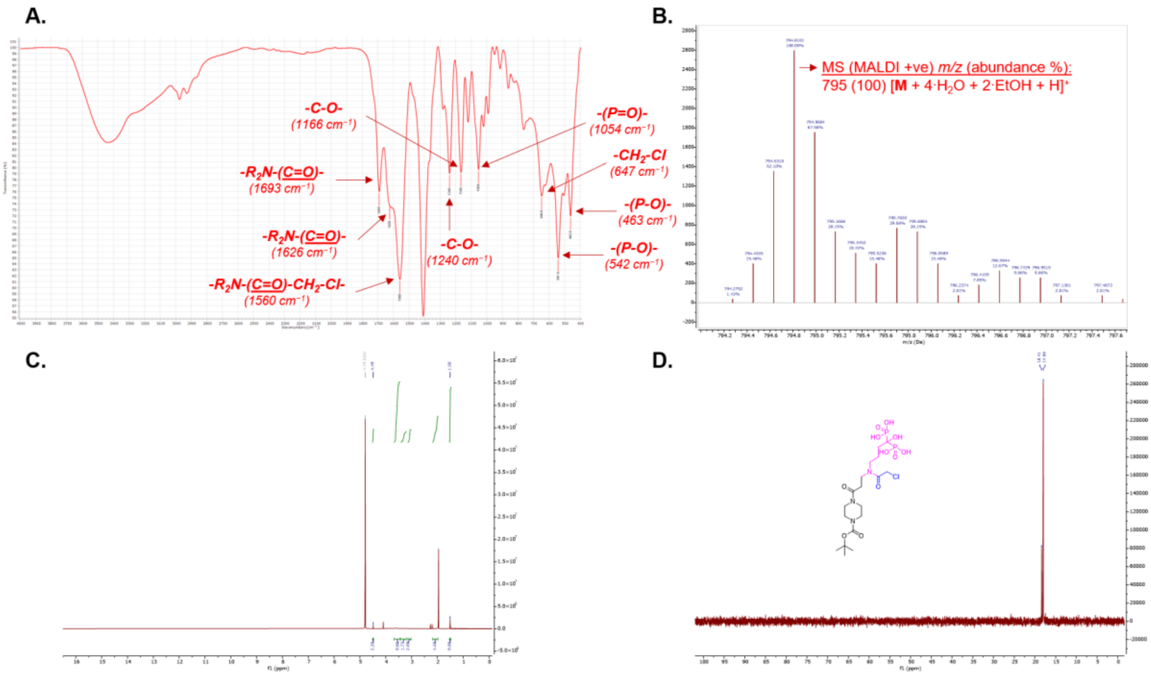

Figure S1. Structural characterization of '**Product 3**': (a) FT-IR, (b) MALDI-ToF, (c) ^{1}H NMR, and (d) ^{31}P NMR spectra.

3.5. Product 4 (4-(2-Chloro-*N*-(3-(4-(2-chloroacetyl)piperazin-1-yl)-3-oxopropyl)acetamido)-1-hydroxybutane-1,1-diyl)bis(phosphonic acid)

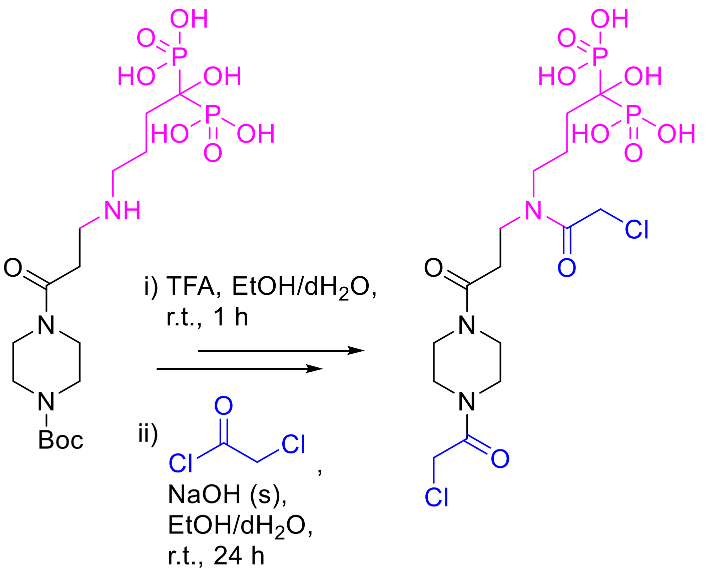

'**Product 2**' (4 g, 8.17 mmol, 1 eq.) was dissolved in a solution containing 60% dH_2O and 40% ethanol (21.5 mL, 0.38 M to '**Product 2**'). Trifluoroacetic acid (TFA) was then added dropwise at 30% of the volume of '**Product 2**' at 0 °C using an ice bath, and the mixture was stirred at room temperature for 1 hour. After completion of the reaction, the reaction solution was removed under reduced pressure. Subsequently, any remaining TFA in the reaction vessel was completely removed using a rotary evaporator with two cycles of ethanol and dichloromethane (DCM) injections. The resulting residue was dissolved in a solution containing 33% dH_2O and 67% EA (1 M to the 'resulting residue'). A finely ground powder of sodium hydroxide (0.98 g, 24.5 mmol, 3 eq.) was then added, and the mixture was stirred at room temperature for 15 minutes. Following this, chloroacetyl chloride (1.56 mL, 19.61 mmol, 2.4 eq.) was added dropwise to the reaction solution, which was stirred at room temperature for 24 hours. The reaction solution was completely removed under reduced pressure to give the product (4.16 g, 94%), and no additional separations were conducted for the next reactions. White solid. Due to the hydrophobic alkyl chains and bisphosphonate group, the compound exhibits very low solubility in both aqueous and organic solvents.

FT-IR (ATR mode): 1678 [ν(R_2N-($\underline{C=O}$)-)], 1634 [ν(R_2N-($\underline{C=O}$)-CH_2-Cl)], 1183 and 1132 [ν(-(P=O)-)], 719 [ν(-CH_2-Cl)], 521 and 446 [ν(-(P-O)-)] cm^{-1}.

^{1}H NMR (400 MHz, D_2O): δ 4.35 (s, 4H, 2 × R_2N-(C=O)-C$\underline{H}_2$-Cl), 4.04−3.59 (m, 8H, -RN-(CH_2CH_2)$_2$-NR-), 3.55−3.28 (m, 2H, R_2N-(C=O)-CH_2-C$\underline{H}_2$-NHR), 3.25−3.13 (m, 2H, -NH-C$\underline{H}_2$-CH_2-CH_2-), 2.31−2.00 (m, 6H, R_2N-(C=O)-C$\underline{H}_2$-CH_2-NHR and -NH-CH_2-C$\underline{H}_2$-C$\underline{H}_2$-) ppm.

^{13}C{H} NMR (101 MHz, D_2O): δ 168.2 (R_2N-($\underline{C}$=O)-CH_2-CH_2-NHR), 163.2 and 162.9 (2 × R_2N-($\underline{C}$=O)-CH_2-Cl), 77.2 (-C(OH)-$(HPO_3)_2$), 50.0, 45.1, and 41.5 (-RN-$(CH_2CH_2)_2$-NR-), 42.2, 42.1 (2 × R_2N-(C=O)-$\underline{C}H_2$-Cl and R_2N-(C=O)-$\underline{C}H_2$-CH_2-NHR), 41.2 (R_2N-(C=O)-CH_2-$\underline{C}H_2$-NHR), 40.1 (-NH-$\underline{C}H_2$-CH_2-CH_2-), 30.6 (-NH-CH_2-$\underline{C}H_2$-CH_2-), 20.7 (-NH-CH_2-CH_2-$\underline{C}H_2$-) ppm.

^{31}P{H} NMR (162 MHz, D_2O): δ 18.8 and 18.2 ppm.

MS (ES +ve) *m/z* (abundance %) for $C_{15}H_{27}Cl_2N_3O_{10}P_2$: calculated $[M + 2MeCN + H]^+$ 625.3539, found $[M + 2MeCN + H]^+$ 625.5791 (20).

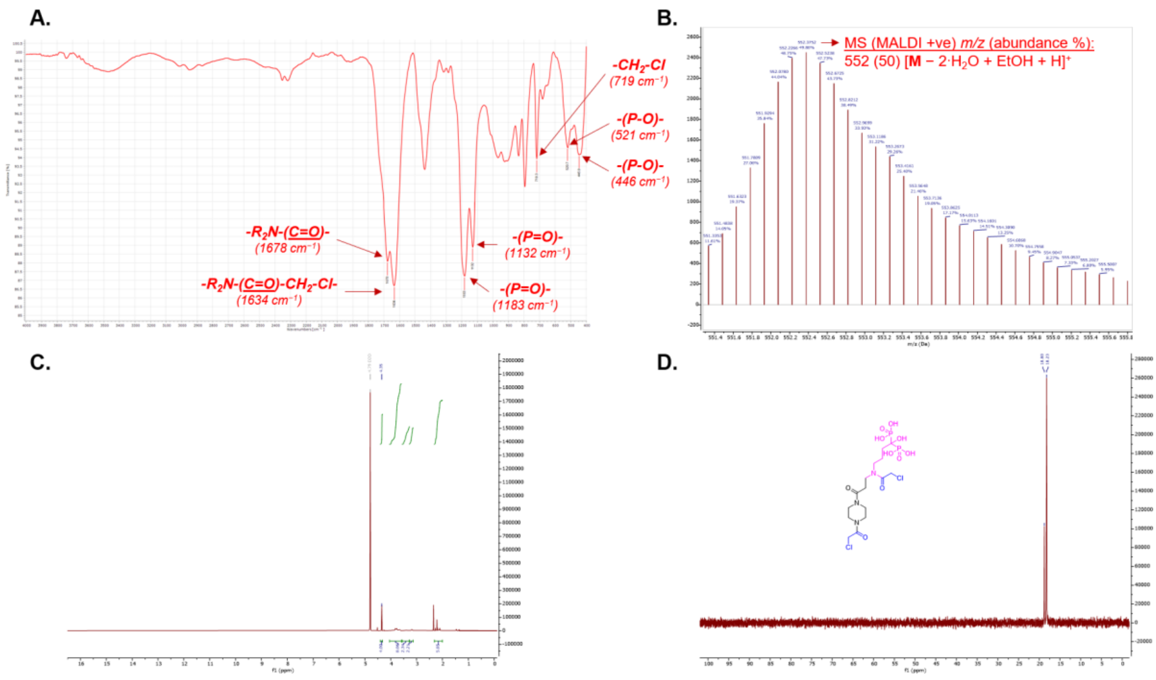

Figure S2. Structural characterization of '**Product 4**': (a) FT-IR, (b) MALDI-ToF, (c) ^{1}H NMR, and (d) ^{31}P NMR spectra.

3.6. Product 5 (*tert*-Butyl 4-(4-((2,5-dioxopyrrolidin-1-yl)oxy)-4-oxobutanoyl)piperazine-1-carboxylate)

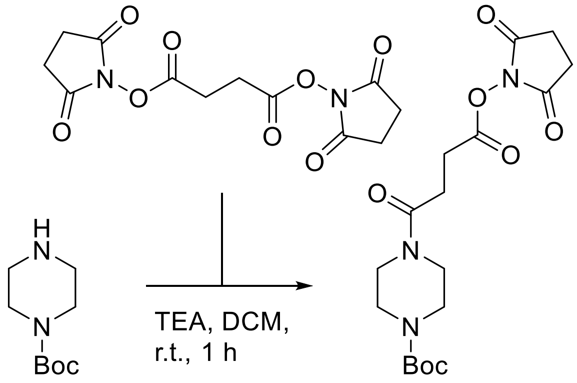

1-Boc-piperazine (2.14 g, 11.53 mmol, 1.2 eq.) was fully dissolved in anhydrous DCM (160.2 mL, 0.06 M to 'bis(2,5-dioxopyrrolidin-1-yl) succinate[10]'), followed by the gradual addition of triethylamine (4.02 mL, 28.83 mmol, 3 eq.). Bis(2,5-dioxopyrrolidin-1-yl) succinate[10] (3 g, 9.61 mmol, 1 eq.) was then added dropwise at room temperature, and the mixture was stirred for 1 hour. After completion of the reaction, DCM was completely removed under reduced pressure to afford the product. No additional separations were conducted for the subsequent reactions, which were carried out continuously due to the product's rapid chemical reactivity in the presence of moisture. Brown solid.

FT-IR (ATR mode): 1706 [ν(-(C=O)-)], 1475 and 1395 [ν(-O-NR_2)], 1212 and 1173 [ν(-C-O-)] cm^{-1}.

3.7. Product 6 (4-(4-(4-(*tert*-Butoxycarbonyl)piperazin-1-yl)-4-oxobutanamido)-1-hydroxybutane-1,1-diyl)bis(phosphonic acid)[8,11]

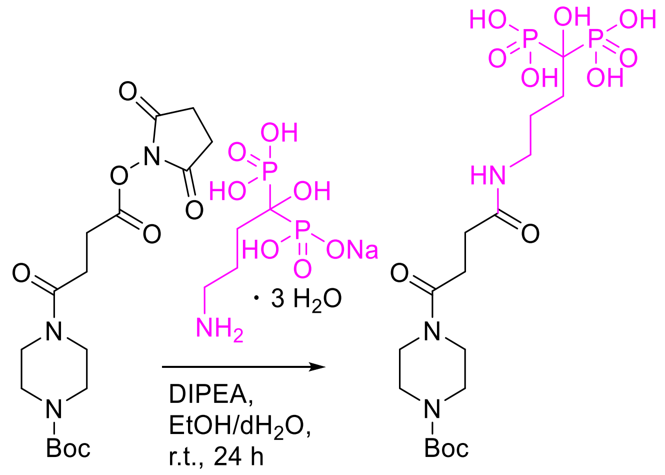

'**Product 5**' (2.83 g, 7.38 mmol, 1.2 eq.) was dissolved in ethanol (68.3 mL, 0.09 M to alendronate) at room temperature, followed by the addition of DIPEA (3 eq.). In another vial, alendronate (2 g, 6.15 mmol, 1 eq.) was dissolved in dH_2O (61.5 mL, 0.1 M to alendronate) at room temperature, and the resulting reaction mixture was added dropwise to the round-bottom flask containing '**Product 5**', followed by stirring at room temperature for 24 hours. The reaction solution was then completely removed under reduced pressure. Subsequently, acetone was added to induce precipitation of '**Product 6**' (3.44 g, 90%), and no additional separations were conducted for the next reactions. Pale-beige solid. Due to the hydrophobic alkyl chains and bisphosphonate group, the compound exhibits very low solubility in both aqueous and organic solvents.

FT-IR (ATR mode): 2978 [ν(-NH-)], 1735 and 1670 [ν(-NR_2-(C=O)-)], 1211 [ν(-(P=O)-)], 1201 and 1157 [ν(-C-O-)], 541 and 441 [ν(-(P-O)-)] cm^{-1}.

^{1}H NMR (400 MHz, D_2O): δ 3.90−3.18 (m, 12H, R_2N-(C=O)-CH_2-CH_2-(C=O)-NR_2 and -RN-(CH_2CH_2)$_2$-NR-), 3.13−3.00 (m, 2H, -NH-CH_2-CH_2-CH_2-), 2.12−1.91 (m, 4H, -NH-CH_2-CH_2-CH_2-), 1.49 (s, 9H, -(C=O)-O-C(CH_3)$_3$) ppm.

^{13}C{H} NMR (101 MHz, D_2O): δ 180.8 and 179.6 (R_2N-(C=O)-CH_2-CH_2-(C=O)-NR_2), 155.8 (-(C=O)-O-C(CH_3)$_3$), 82.7 (-(C=O)-O-C(CH_3)$_3$), 73.6 (-C(OH)-(HPO_3)$_2$), 54.4 (R_2N-(C=O)-CH_2-CH_2-(C=O)-NR_2), 52.2, 43.0, and 42.6 (-RN-(CH_2CH_2)$_2$-NR-), 40.0 (-NH-CH_2-CH_2-CH_2-), 30.2 (-NH-CH_2-CH_2-CH_2-), 27.5 (-(C=O)-O-C(CH_3)$_3$), 22.4 (-NH-CH_2-CH_2-CH_2-) ppm.

^{31}P{H} NMR (162 MHz, D_2O): δ 17.9 ppm.

MS (ES +ve) *m/z* (abundance %) for $C_{17}H_{33}N_3O_{11}P_2$: calculated $[M + Na]^+$ 540.3948, found $[M + Na]^+$ 540.6430 (30).

3.8. Product 7 (4-(4-(4-(2-Chloroacetyl)piperazin-1-yl)-4-oxobutanamido)-1-hydroxybutane-1,1-diyl)bis(phosphonic acid)

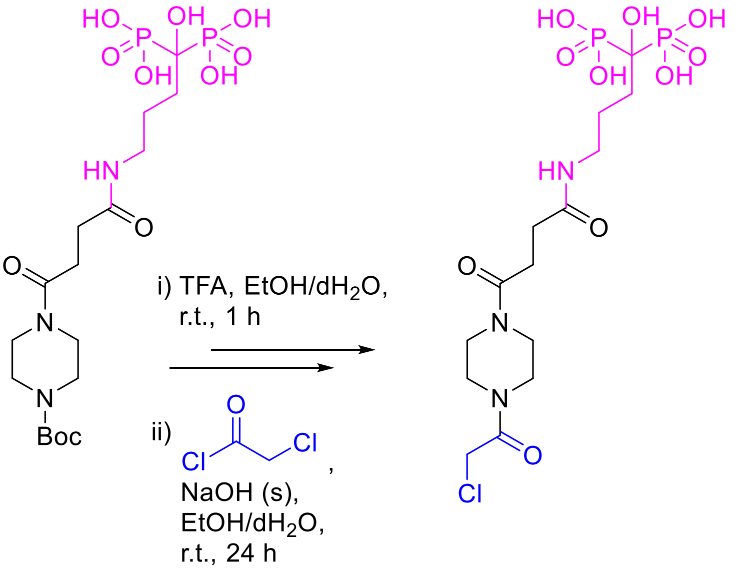

'**Product 6**' (3 g, 5.80 mmol, 1 eq.) was dissolved in a solution of 60% dH_2O and 40% ethanol (15.3 mL, 0.38 M to '**Product 6**'). Subsequently, TFA was added dropwise to the solution at 30% of the volume of '**Product 6**' at 0 °C using an ice bath, followed by stirring at room temperature for 1 hour. After the reaction was complete, the reaction solution was completely removed under reduced pressure. Ethanol or DCM was then added to the reaction vessel twice each, and the remaining TFA in the reaction vessel was completely removed using a rotavapor. The resulting residue was dissolved in a solution of 33% dH_2O and 67% EA (1 M to the 'resulting residue'). A finely ground powder of sodium hydroxide (0.70 g, 17.4 mmol, 3 eq.) was then added, and the mixture was stirred at room temperature for 15 minutes. Subsequently, chloroacetyl chloride (0.94 mL, 11.6 mmol, 1.2 eq.) was added dropwise to the reaction mixture, and this was stirred at room temperature for 24 hours. The reaction solution was then completely removed under reduced pressure to give the product (2.63 g, 92%), and no additional separations were conducted for the next reactions. White solid. Due to the hydrophobic alkyl chains and bisphosphonate group, the compound exhibits very low solubility in both aqueous and organic solvents.

FT-IR (ATR mode): 1680 and 1631 [ν(R$_2$N-(<u>C=O</u>)-)], 1561 [ν(R$_2$N-(<u>C=O</u>)-CH$_2$-Cl)], 1155 and 1101 [ν(-(P=O)-)], 890 [ν(-CH$_2$-Cl)], 542 and 447 [ν(-(P-O)-)] cm^{-1}.

^{1}H NMR (600 MHz, D$_2$O): δ 3.97 (s, 2H, R$_2$N-(C=O)-C<u>H</u>$_2$-Cl), 4.05−3.03 (m, 14H, R$_2$N-(C=O)-CH$_2$-CH$_2$-(C=O)-NR$_2$, -RN-(CH$_2$CH$_2$)$_2$-NR-, and -NH-C<u>H</u>$_2$-CH$_2$-CH$_2$-), 2.20−2.02 (m, 4H, -NH-CH$_2$-C<u>H</u>$_2$-C<u>H</u>$_2$-) ppm.

^{13}C{H} NMR (101 MHz, D$_2$O): δ 175.1 and 174.2 (R$_2$N-(<u>C</u>=O)-CH$_2$-CH$_2$-(<u>C</u>=O)-NR$_2$), 162.8 (R$_2$N-(<u>C</u>=O)-CH$_2$-Cl), 73.6 (-C(OH)-(HPO$_3$)$_2$), 52.2, 44.0, and 40.2 (-RN-(CH$_2$CH$_2$)$_2$-NR-), 40.1 (-NH-<u>C</u>H$_2$-CH$_2$-CH$_2$-), 30.9 (-NH-CH$_2$-<u>C</u>H$_2$-CH$_2$-), 22.3 (-NH-CH$_2$-CH$_2$-<u>C</u>H$_2$-) ppm.

^{31}P{H} NMR (162 MHz, D$_2$O): δ 18.5 and 18.2 ppm.

MS (ES +ve) *m/z* (abundance %) for $C_{14}H_{26}ClN_3O_{10}P_2$: calculated [M + MeCN + H]$^+$ 535.8306, found [M + MeCN + H]$^+$ 536.1517 (25).

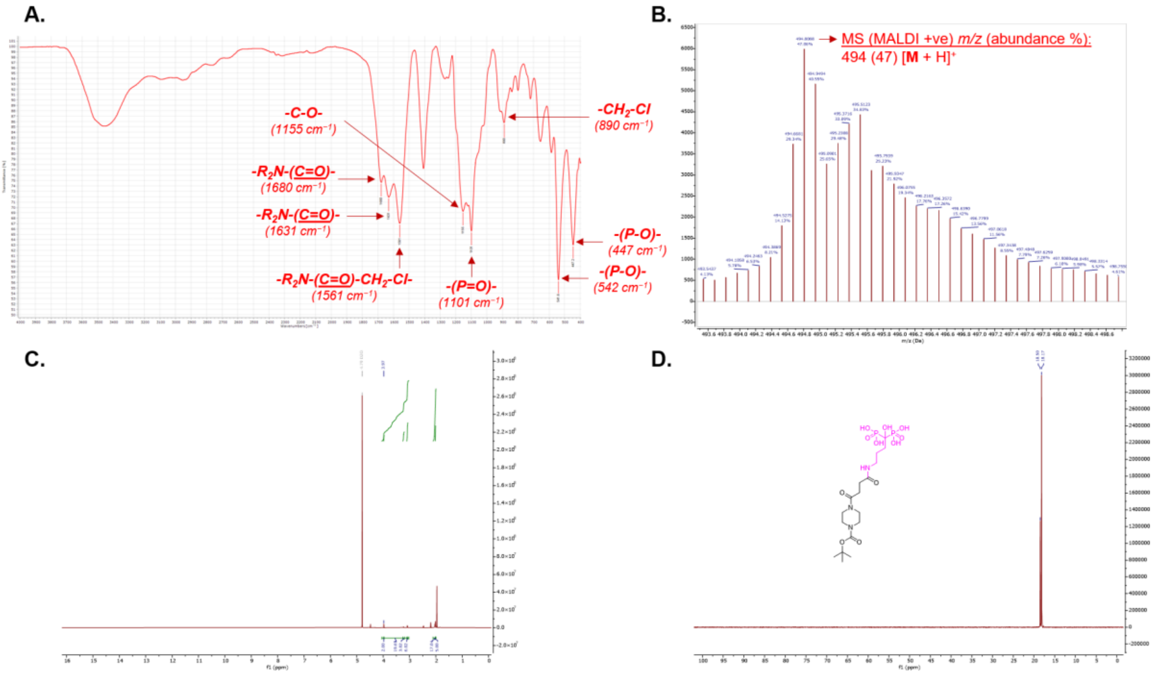

Figure S3. Structural characterization of '**Product 7**': (a) FT-IR, (b) MALDI-ToF, (c) ^{1}H NMR, and (d) ^{31}P NMR spectra.

→ A total of 140 different bone-targeting piperazine-based bisphosphonate-linked ionizable lipids were synthesized using capping ligands 'Products 7, 11, and 16' on a multi-gram scale, based on the synthetic method described below.[12] The resulting bone-targeting ionizable lipids were analyzed by FT-IR, MS (ES and MALDI-ToF), and 2D NMR spectroscopy before undergoing *in vitro* and *in vivo* screening.

3.9. Synthetic protocol for the bone-targeting ionizable lipids ‘Type 1’

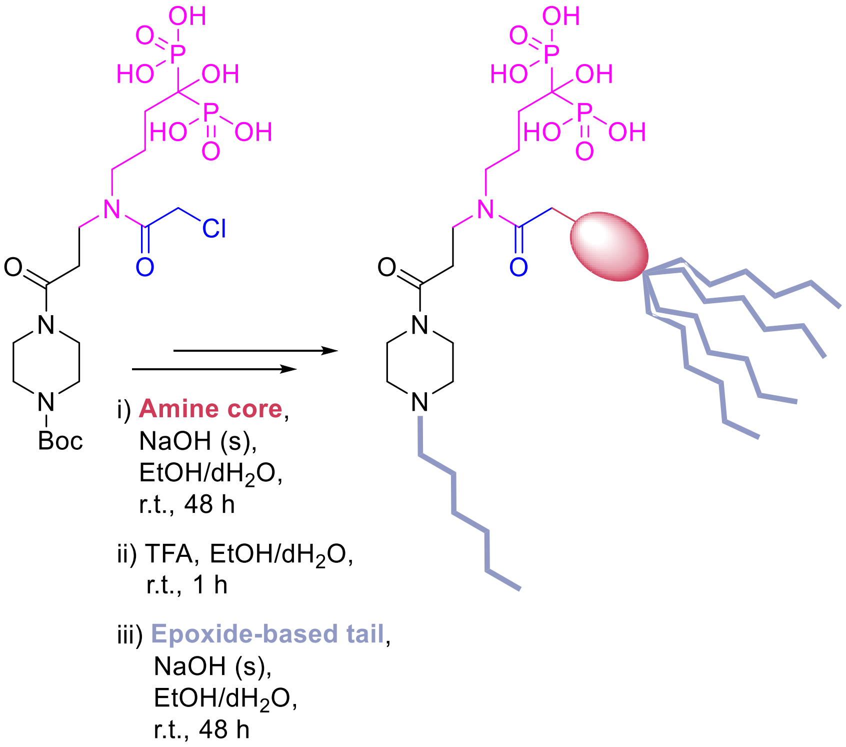

A finely ground powder of sodium hydroxide (3 eq.) was added to a solution containing 60% dH_2O and 40% ethanol (0.12 M to ‘**Product 3**’). After adding the amine (1.1 eq.) to the solution, vigorous stirring was carried out at room temperature for 30 minutes. '**Product 3**' (1 eq.) was then added dropwise to the reaction mixture at room temperature, and stirring was continued for 48 hours. Upon completion of the reaction, the reaction solution was completely removed, and acetone was added to wash out any unreacted amine through a filtration method. The obtained product was dissolved in a solution consisting of 60% dH_2O and 40% ethanol (0.12 M to ‘**Product 3**’). Subsequently, the solution was cooled to 0 °C on an ice bath, and TFA was added dropwise until the pH reached 2−3. This was stirred for an additional hour at room temperature. After the reaction was complete, ethanol or DCM was added to the reaction vessel twice each, and the remaining TFA in the reaction vessel was completely removed using a rotavapor. The precipitated residue was dissolved in a solution containing 60% dH_2O and 40% ethanol (0.12 M to ‘**Product 3**’), and a finely ground powder of sodium hydroxide was added and stirred at room temperature for 30 minutes to raise the pH of the reaction mixture to above 12. The 'epoxide-based tail' (2.4 equivalents of an epoxide were added for a primary amine and 1.2 equivalents for a secondary amine) was then added dropwise to the reaction mixture at room temperature, and the reaction was allowed to proceed at 80 °C for 2 days. After completion of the reaction, the reaction solution was completely removed under reduced pressure. Subsequently, acetone was added to induce precipitation of the product, and the solution was decanted to remove the liquid. *n*-Hexane was then added to the remaining mixture to remove unreacted epoxide-based tails. Afterward, acetone was added again to induce precipitation of the product, and the product was filtered to obtain pure product.

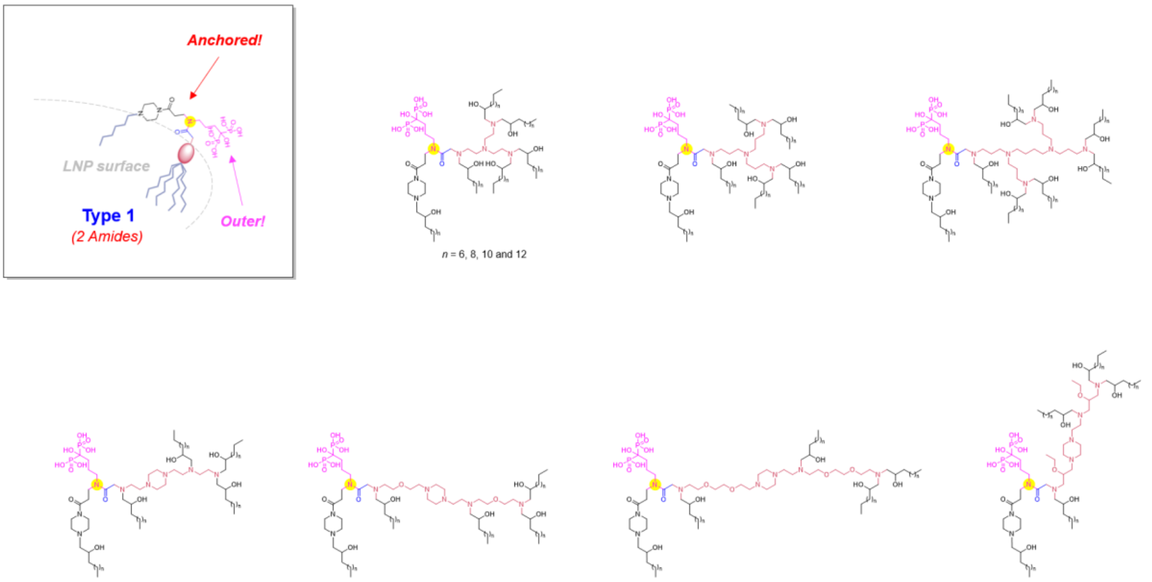

Figure S4. Targeting molecules for the '**Type 1**' bone-targeting ionizable lipids.

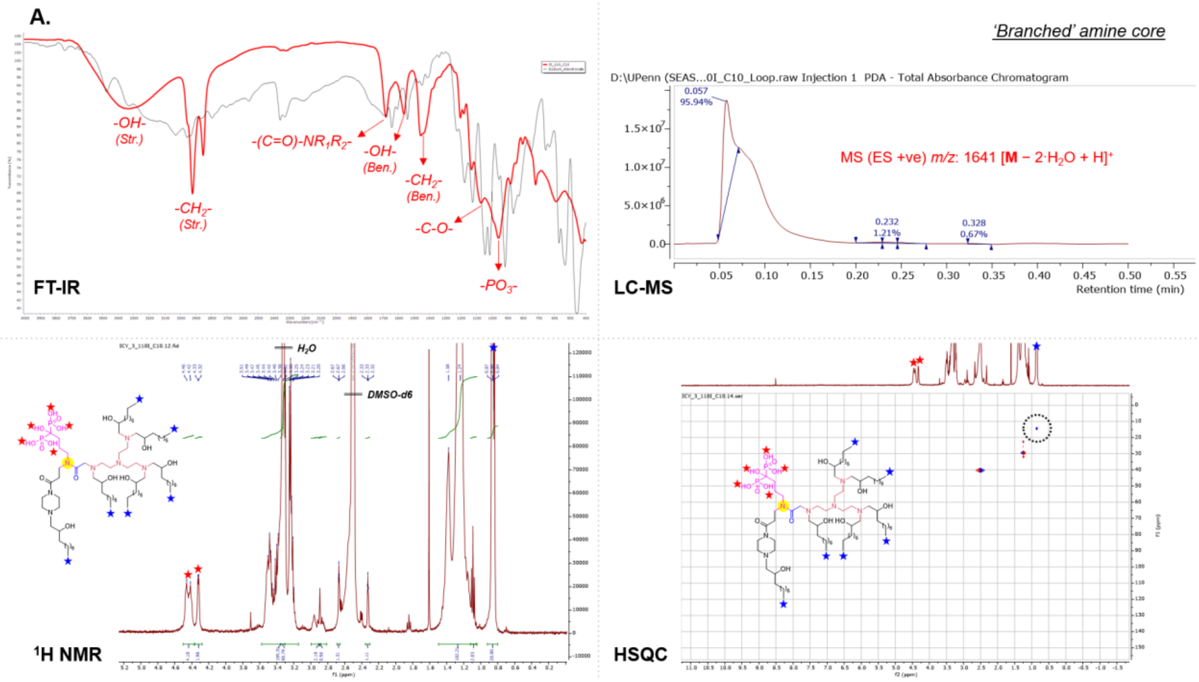

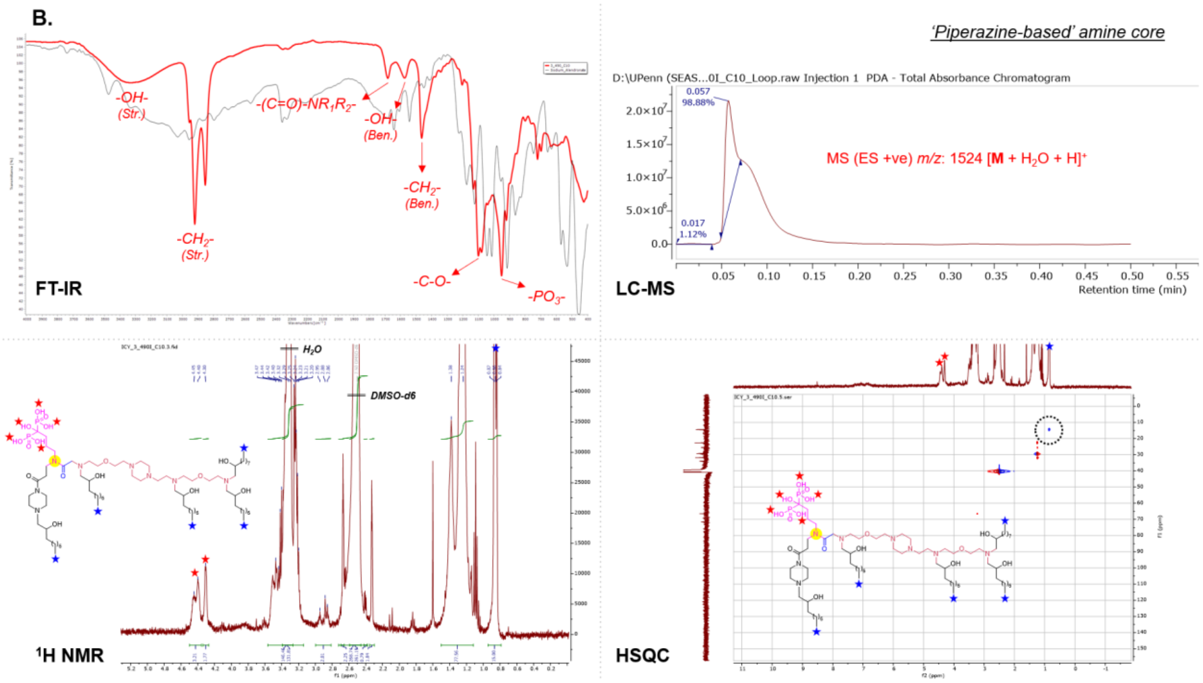

Figure S5. Structural characterization of the '**Type 1**' bone-targeting ionizable lipids based on (a) 'Branched' and (b) 'Piperazine' amine cores.

3.10. Synthetic protocol for the bone-targeting ionizable lipids 'Type 2'

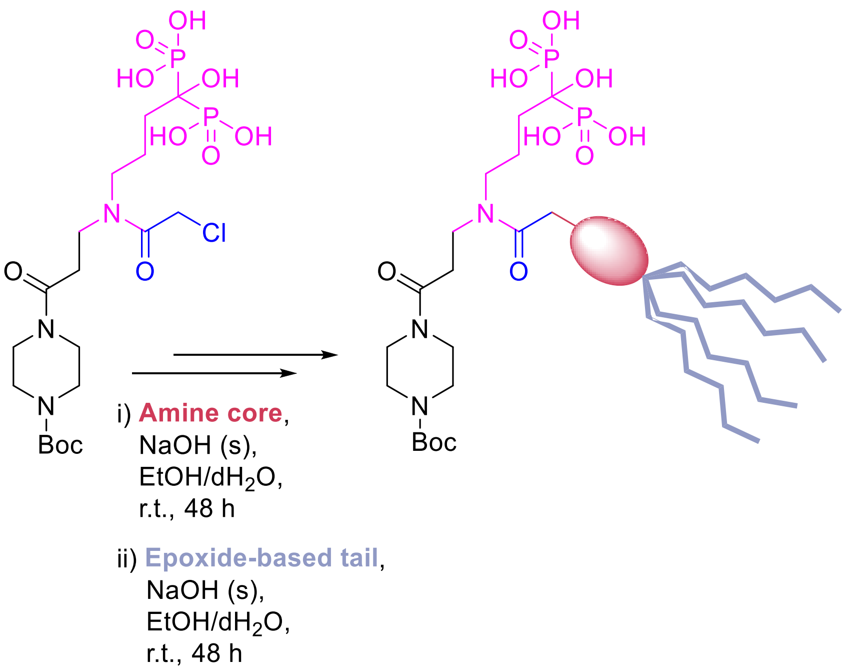

A finely ground powder of sodium hydroxide (3 eq.) was added to a solution containing 60% dH_2O and 40% ethanol (0.12 M to '**Product 3**'). After adding the amine (1.1 eq.) to the solution, vigorous stirring was carried out at room temperature for 30 minutes. '**Product 3**' (1 eq.) was then added dropwise to the reaction mixture at room temperature, and stirring was continued for 48 hours. Upon completion of the reaction, the reaction solution was completely removed, and acetone was added to wash out

any unreacted amine through a filtration method. Subsequently, the precipitated residue was dissolved in a solution containing 60% dH_2O and 40% ethanol (0.12 M to '**Product 3**'), and a finely ground powder of sodium hydroxide was added and stirred at room temperature for 30 minutes to raise the pH of the reaction mixture to 12. The 'epoxide-based tail' (2.4 equivalents of an epoxide were added for a primary amine and 1.2 equivalents for a secondary amine) was then added dropwise to the reaction mixture at room temperature, and the reaction was allowed to proceed at 80 °C for 2 days. After completion of the reaction, the reaction solution was completely removed under reduced pressure. Subsequently, acetone was added to induce precipitation of the product, and the solution was decanted to remove the liquid. *n*-Hexane was then added to the remaining mixture to remove unreacted epoxide-based tails. Afterward, acetone was added again to induce precipitation of the product, and the product was filtered to obtain pure product.

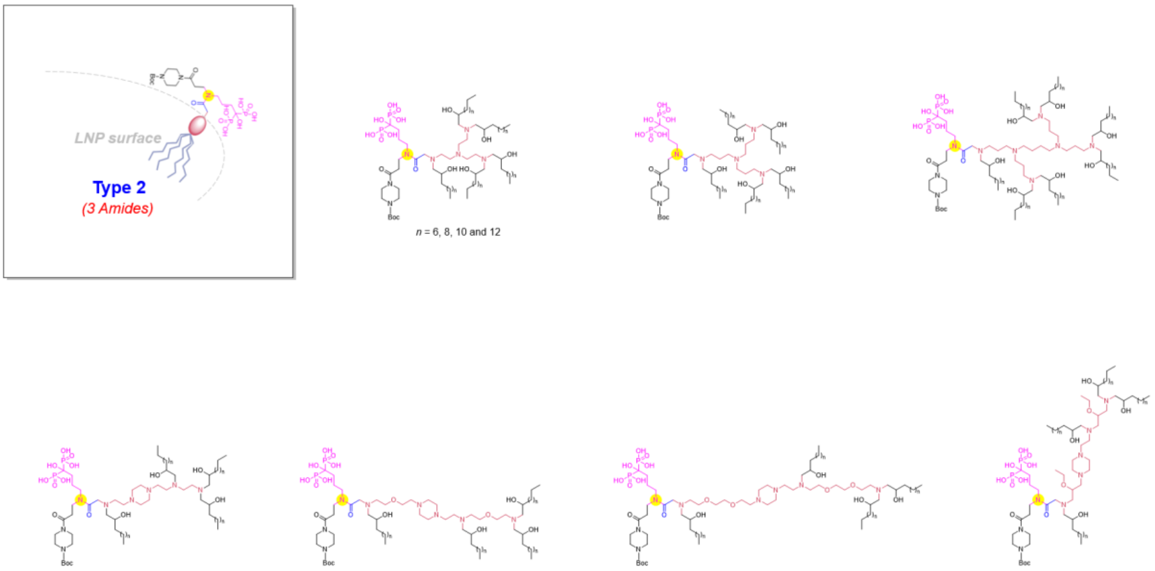

Figure S6. Targeting molecules for the '**Type 2**' bone-targeting ionizable lipids.

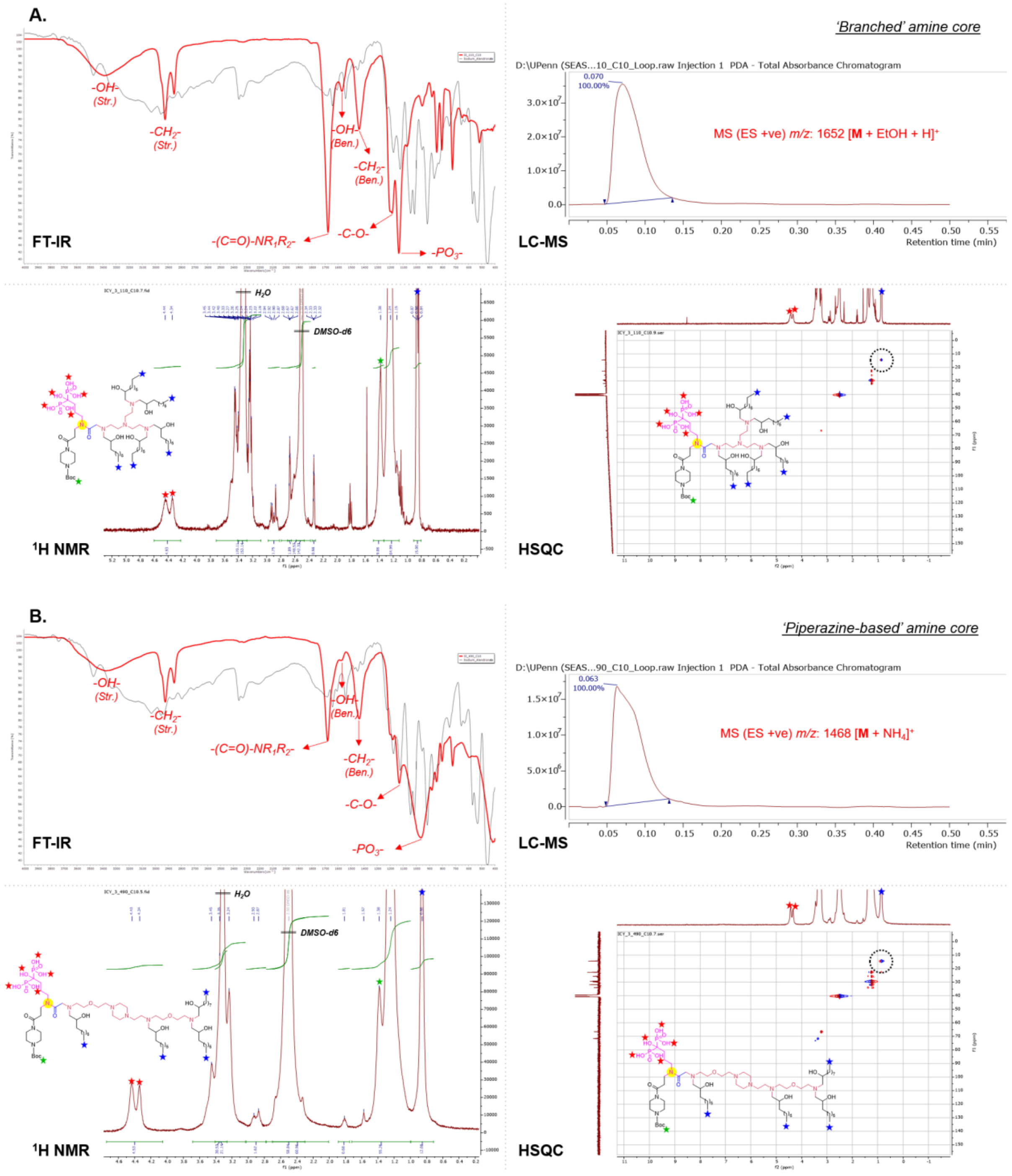

Figure S7. Structural characterization of the '**Type 2**' bone-targeting ionizable lipids based on (a) 'Branched' and (b) 'Piperazine' amine cores.

3.11. Synthetic protocol for the bone-targeting ionizable lipids 'Type 3'

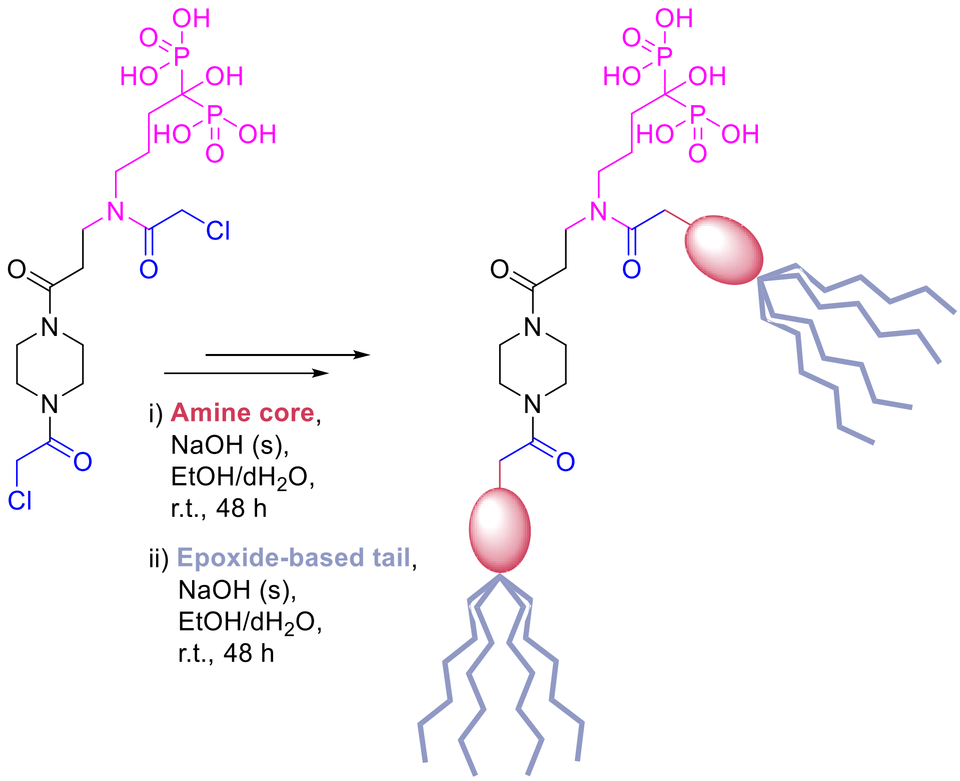

A finely ground powder of sodium hydroxide (3 eq.) was added to a solution containing 60% dH_2O and 40% ethanol (0.12 M to '**Product 4**'). After adding the amine (1.1 eq.) to the solution, vigorous stirring was carried out at room temperature for 30 minutes. '**Product 4**' (1 eq.) was then added dropwise to the reaction mixture at room temperature, and stirring was continued for 48 hours. The solution was then vigorously stirred at room temperature for 30 minutes after adding amine. It was stirred at room temperature for an additional 48 hours. Upon completion of the reaction, the reaction solution was completely removed, and acetone was added to wash out any unreacted amine through a filtration method. The precipitated residue was dissolved in a solution containing 60% dH_2O and 40% ethanol (0.12 M to the 'precipitated residue'), and a finely ground powder of sodium hydroxide was added and stirred at room temperature for 30 minutes to adjust the pH of the reaction mixture to above 12. Subsequently, 'epoxide-based tail' (2.4 equivalents of an epoxide were added for a primary amine and 1.2 equivalents for a secondary amine) was added dropwise at room temperature, and the reaction was allowed to proceed at 80 °C for 2 days. After completion of the reaction, the reaction solution was completely removed under reduced pressure. Subsequently, acetone was added to induce precipitation of the product, and the solution was decanted to remove the liquid. *n*-Hexane was then added to the remaining mixture to remove unreacted epoxide-based tails. Afterward, acetone was added again to induce precipitation of the product, and the product was filtered to obtain pure product.

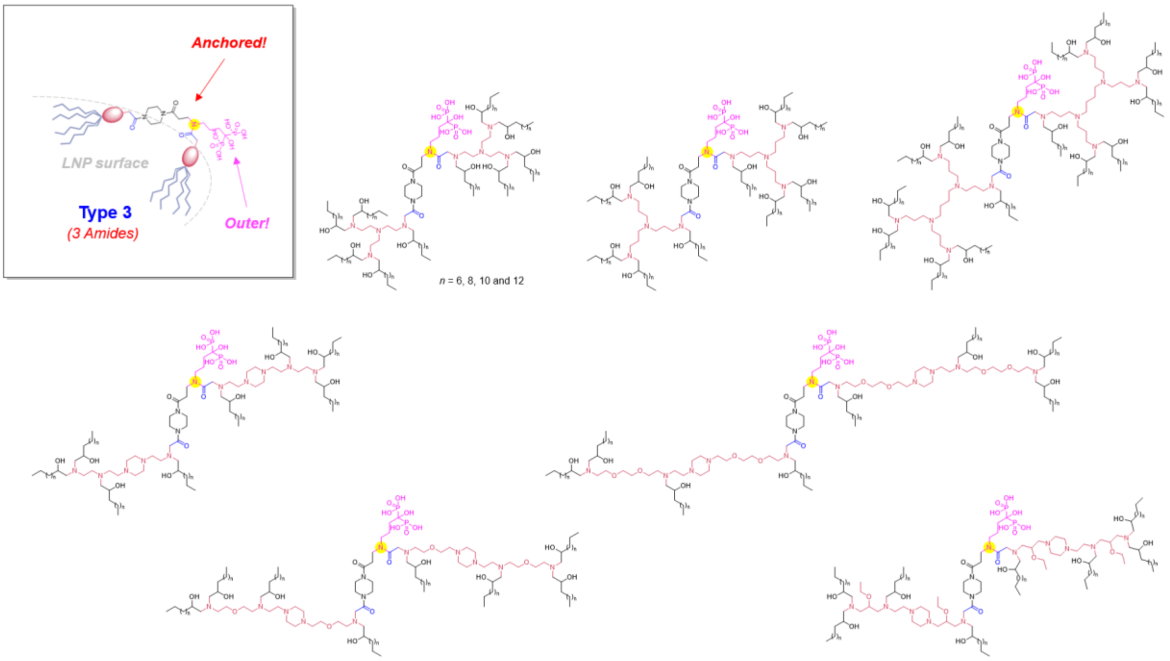

Figure S8. Targeting molecules for the '**Type 3**' bone-targeting ionizable lipids.

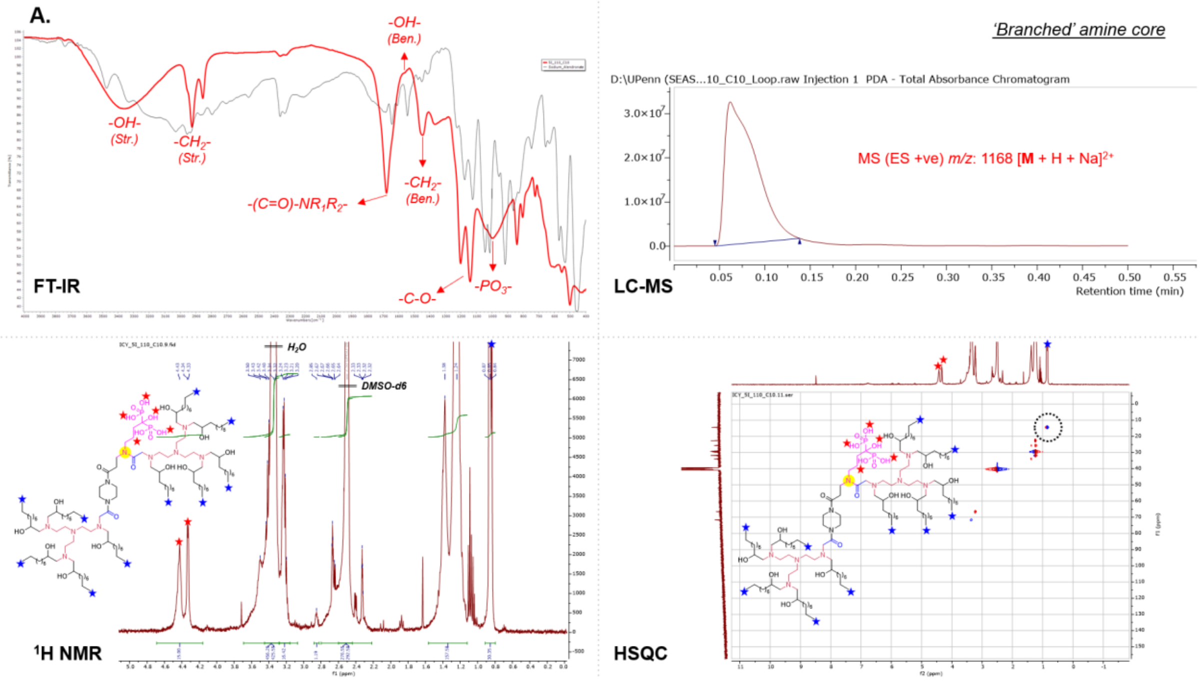

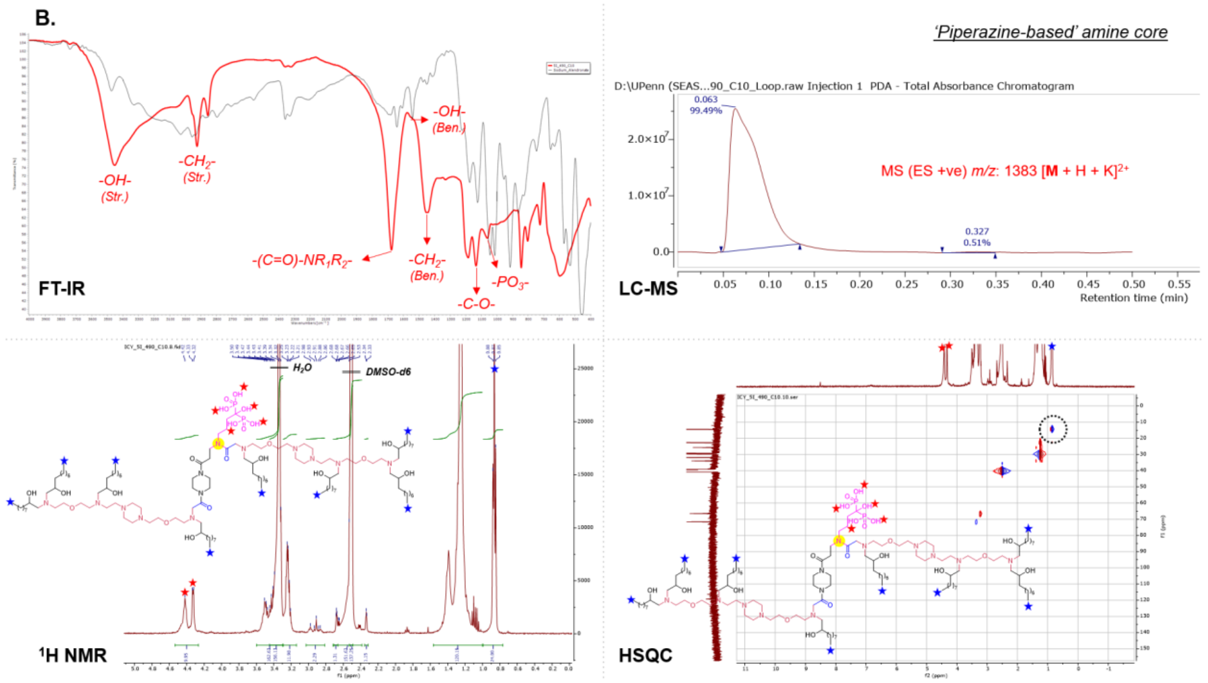

Figure S9. Structural characterization of the '**Type 3**' bone-targeting ionizable lipids based on (a) 'Branched' and (b) 'Piperazine' amine cores.

3.12. Synthetic protocol for the bone-targeting ionizable lipids 'Type 4'

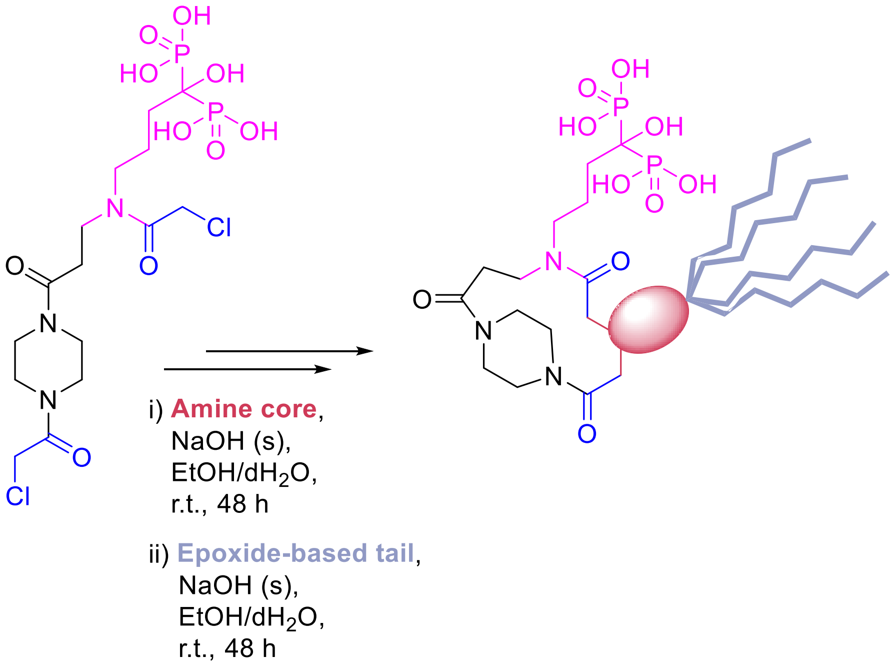

A finely ground powder of sodium hydroxide (3 eq.) was added to a solution containing 60% dH_2O and 40% ethanol (0.12 M to '**Product 4**'). After adding the amine (1.1 eq.) to the solution, vigorous stirring was carried out at room temperature for 30 minutes. '**Product 4**' (1 eq.) was then added dropwise to the reaction mixture at room temperature, and stirring was continued for 48 hours. The solution was then vigorously stirred at room temperature for 30 minutes after adding amine. It was stirred at room

temperature for an additional 48 hours. Upon completion of the reaction, the reaction solution was completely removed, and acetone was added to wash out any unreacted amine through a filtration method. The precipitated residue was dissolved in a solution containing 60% dH_2O and 40% ethanol (0.12 M to the ‘precipitated residue’), and a finely ground powder of sodium hydroxide was added and stirred at room temperature for 30 minutes to adjust the pH of the reaction mixture to above 12. Subsequently, 'epoxide-based tail' (2.4 equivalents of an epoxide were added for a primary amine and 1.2 equivalents for a secondary amine) was added dropwise at room temperature, and the reaction was allowed to proceed at 80 °C for 2 days. After completion of the reaction, the reaction solution was completely removed under reduced pressure. Subsequently, acetone was added to induce precipitation of the product, and the solution was decanted to remove the liquid. *n*-Hexane was then added to the remaining mixture to remove unreacted epoxide-based tails. Afterward, acetone was added again to induce precipitation of the product, and the product was filtered to obtain pure product.

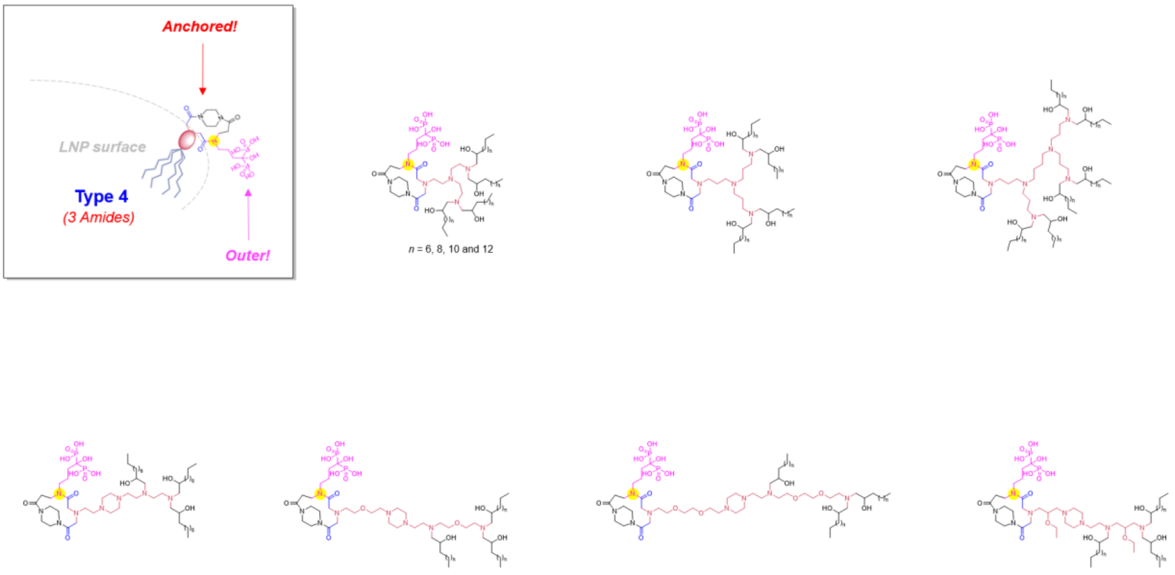

Figure S10. Targeting molecules for the '**Type 4**' bone-targeting ionizable lipids.

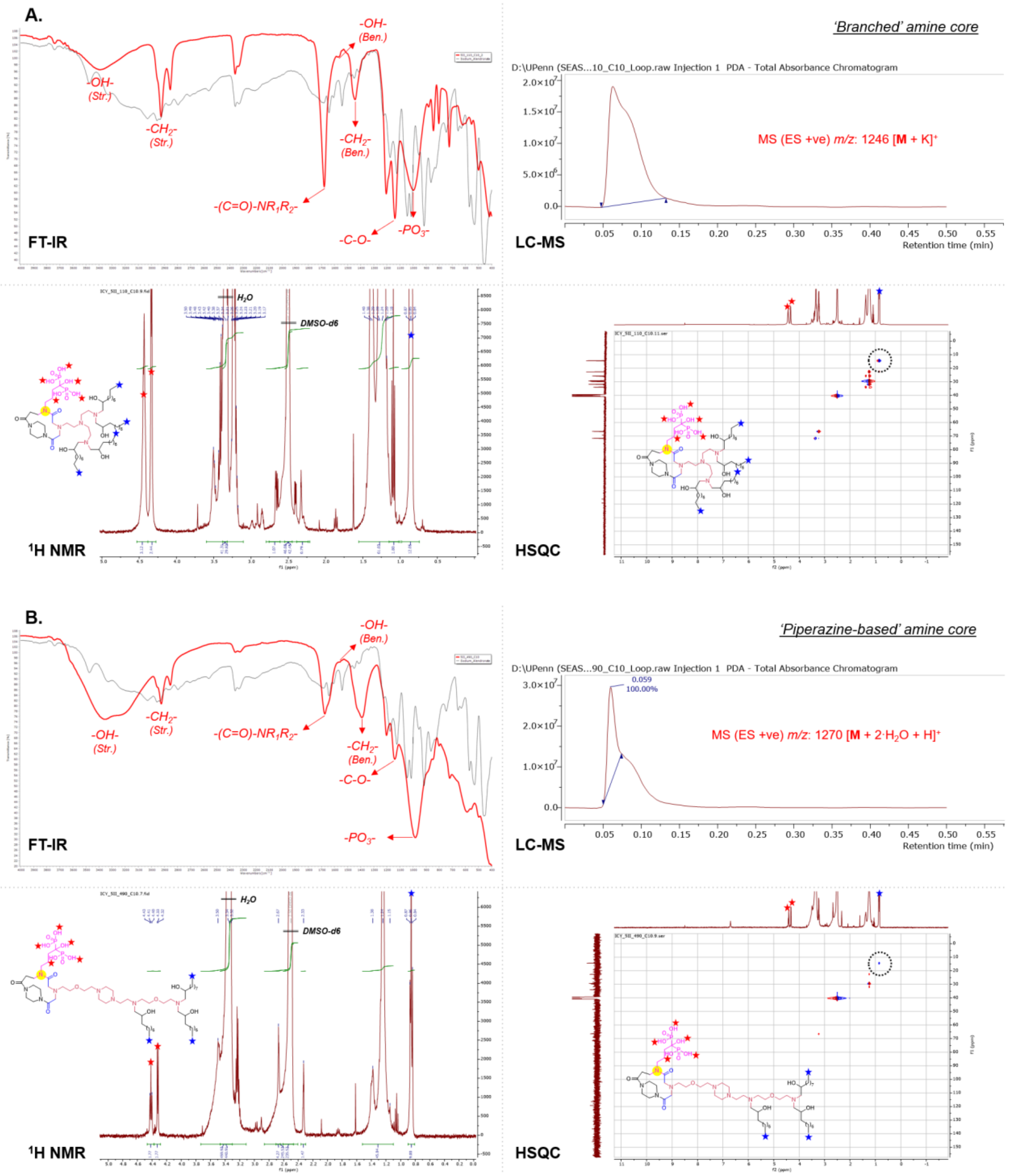

Figure S11. Structural characterization of the '**Type 4**' bone-targeting ionizable lipids based on (a) 'Branched' and (b) 'Piperazine' amine cores.

3.13. Synthetic protocol for the bone-targeting ionizable lipids 'Type 5'

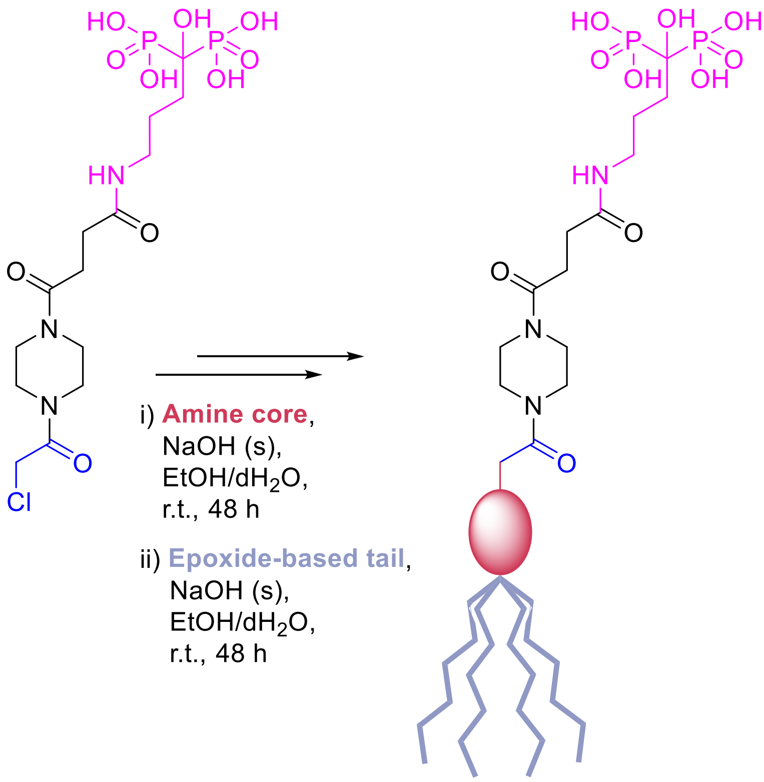

A finely ground powder of sodium hydroxide (3 eq.) was added to a solution containing 60% dH_2O and 40% ethanol (0.12 M to '**Product 7**'). After adding the amine (1.1 eq.) to the solution, vigorous stirring was carried out at room temperature for 30 minutes. '**Product 7**' (1 eq.) was then added dropwise to the reaction mixture at room temperature, and stirring was continued for 48 hours. Upon completion of the reaction, the reaction solution was completely removed, and acetone was used to wash out any unreacted amine through a filtration method. The precipitated residue was dissolved in a solution of 60% dH_2O and 40% ethanol (0.12 M to the 'precipitated residue'), followed by the addition of a finely ground sodium hydroxide powder and stirring at room temperature for 30 minutes to adjust the pH of the reaction mixture to above 12. Subsequently, the 'epoxide-based tail' (2.4 equivalents of an epoxide were added for a primary amine and 1.2 equivalents for a secondary amine) was added dropwise at room temperature, and the reaction was allowed to proceed at 80 °C for 2 days. After completion of the reaction, the reaction solution was completely removed under reduced pressure. Subsequently, acetone was added to induce precipitation of the product, and the solution was decanted to remove the liquid. *n*-Hexane was then added to the remaining mixture to remove unreacted epoxide-based tails. Afterward, acetone was added again to induce precipitation of the product, and the product was filtered to obtain pure product.

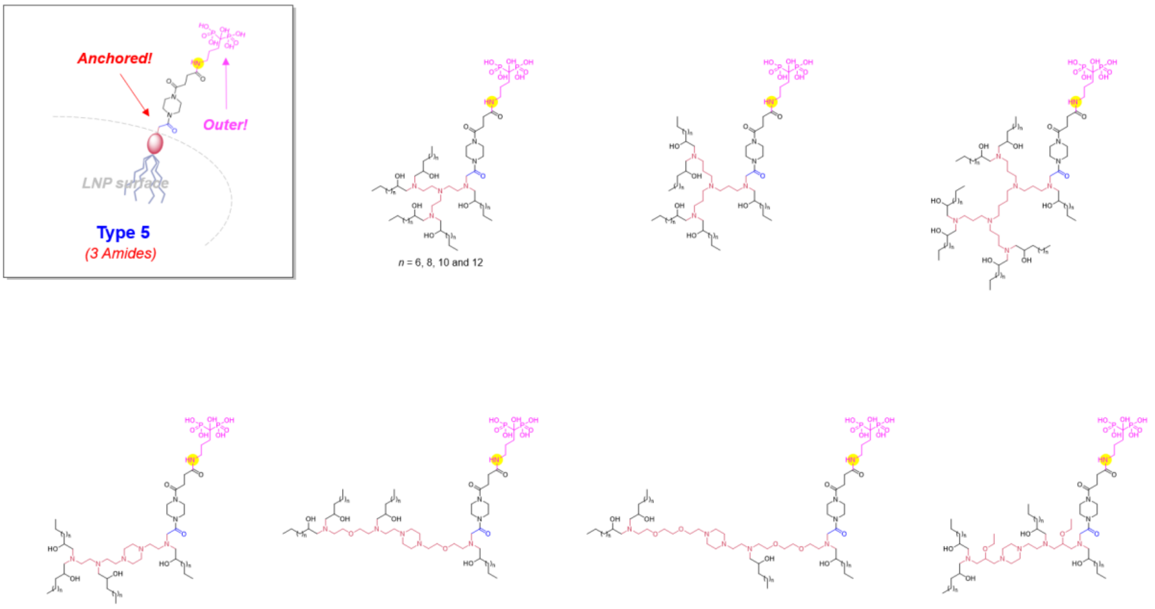

Figure S12. Targeting molecules for the '**Type 5**' bone-targeting ionizable lipids.

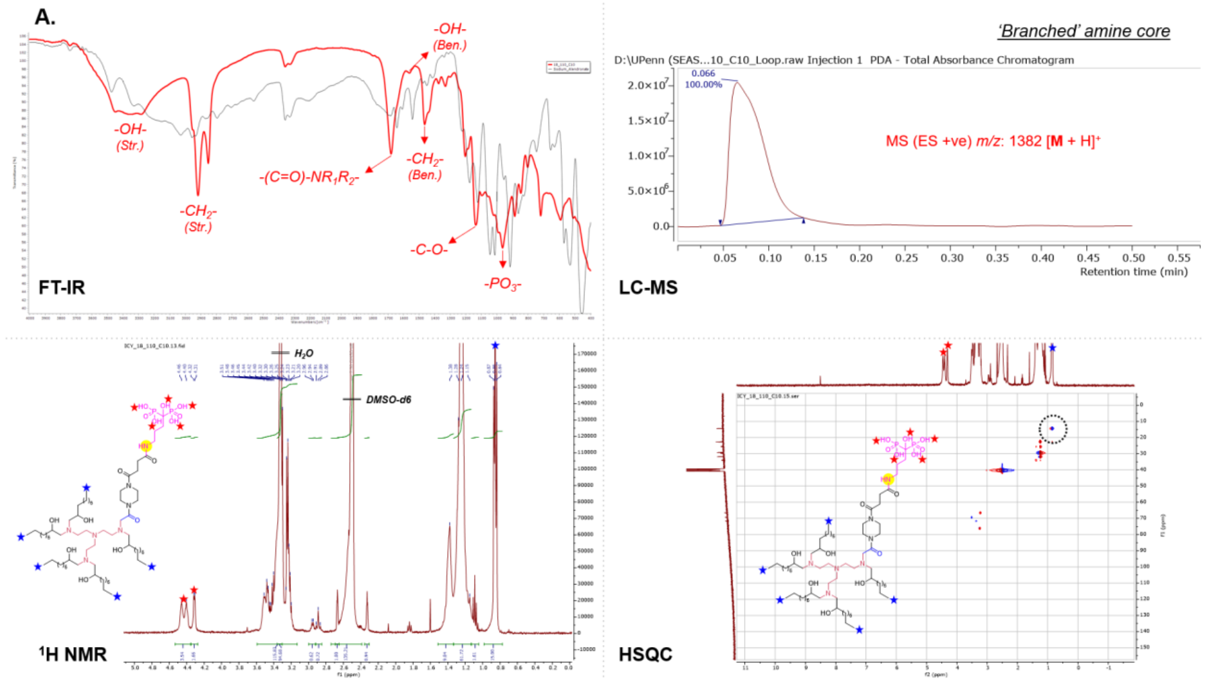

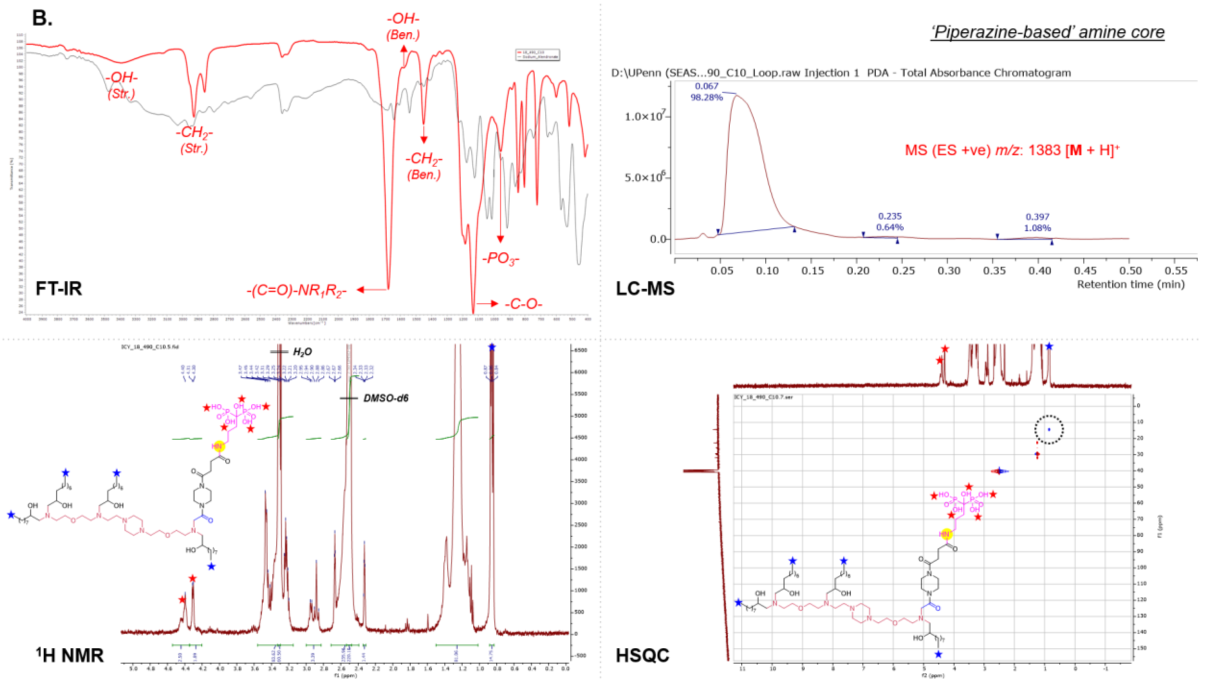

Figure S13. Structural characterization of the '**Type 5**' bone-targeting ionizable lipids based on (a) 'Branched' and (b) 'Piperazine' amine cores.

3.14. Structural characterization of 'Type1-P1-C12'

White hygroscopic solid. The purity and yield of the compound exceeded 95%. Due to its very low solubility in both aqueous and organic solvents, the structure of the compound was elucidated using two different NMR solvents D_2O and DMSO-*d6*.

FT-IR (ATR mode): 3410 and 3316 [ν(-OH)], 2954, 2915, and 2848 [ν(-CH_2- and -CH_3)], 1676 [ν(R_2N-(C=O)-)], 1465 [ν(-CH_2- and CH_3)], 1335 [ν(-OH)], 1144 [ν(R_2HC-O-)], 1097 and 1070 [ν(-(P=O)-)], 592 and 519 [ν(-(P-O)-)] cm^{-1}.

^{1}H NMR (600 MHz, DMSO-*d6*): δ 4.62−4.34 (m, 10H, -OH), 1.43−1.33 and 1.29−1.16 (m, 90H, -CH_2- (epoxide-based alkyl chains)), 0.85 (t, 24H, -CH_3, *J* = 6.9 Hz) ppm.

^{1}H NMR (600 MHz, D_2O): δ 4.00−2.50 (m, 51H, -CH_2- (main structural framework)), 1.98 (m, 4H, -NH-CH_2-CH_2-CH_2-), 1.76−0.94 (m, 90H, -CH_2- (epoxide-based alkyl chains)), 0.86−0.69 (m, 15H, -CH_3) ppm.

^{13}C{H} NMR (151 MHz, DMSO-*d6*): δ 175.5, 166.8, and 158.1 (-(C=O)-), 75.0 (-C(OH)-(HPO_3)$_2$), 71.2, 68.9, 66.1, and 65.8 (R_2C(OH)-), 56.2, 51.7, and 46.2 (-CH_2-(C=O)-N($CH_2$$CH_2$)$_2$N-(C=O)-, -($CH_2$)$_2NCH_2$$CH_2$N($CH_2$-)$CH_2$$CH_2$-N($CH_2$$CH_2$)$_2NCH_2$$CH_2$N($CH_2$)$_2$-, and -$CH_2$N(-(C=O)-)$CH_2$$CH_2$$CH_2$$CH_2$C(OH)-($HPO_3$)$_2$), 39.5 (-NH-$CH_2$-$CH_2$-$CH_2$-), 33.9, 33.5, 31.4, 29.4, 29.3, 29.2, 29.1, 29.0, 28.9, 28.8, 25.3, 25.2, and 25.1 (-CH_2- (epoxide-based alkyl chains)), 31.4 (-NH-CH_2-CH_2-CH_2-), 22.2 (-NH-CH_2-CH_2-CH_2-), 15.2 (-CH_2-C(OH)-(HPO_3)$_2$), 14.1 (-CH_3) ppm.

MS (MALDI +ve) *m/z* (abundance %) for $C_{83}H_{170}N_8O_{14}P_2$: calculated [M − H_2PO_3 + H_2O + H]$^+$ 1504.2663, found [M − H_2PO_3 + H_2O + H]$^+$ 1505.5477 (100).

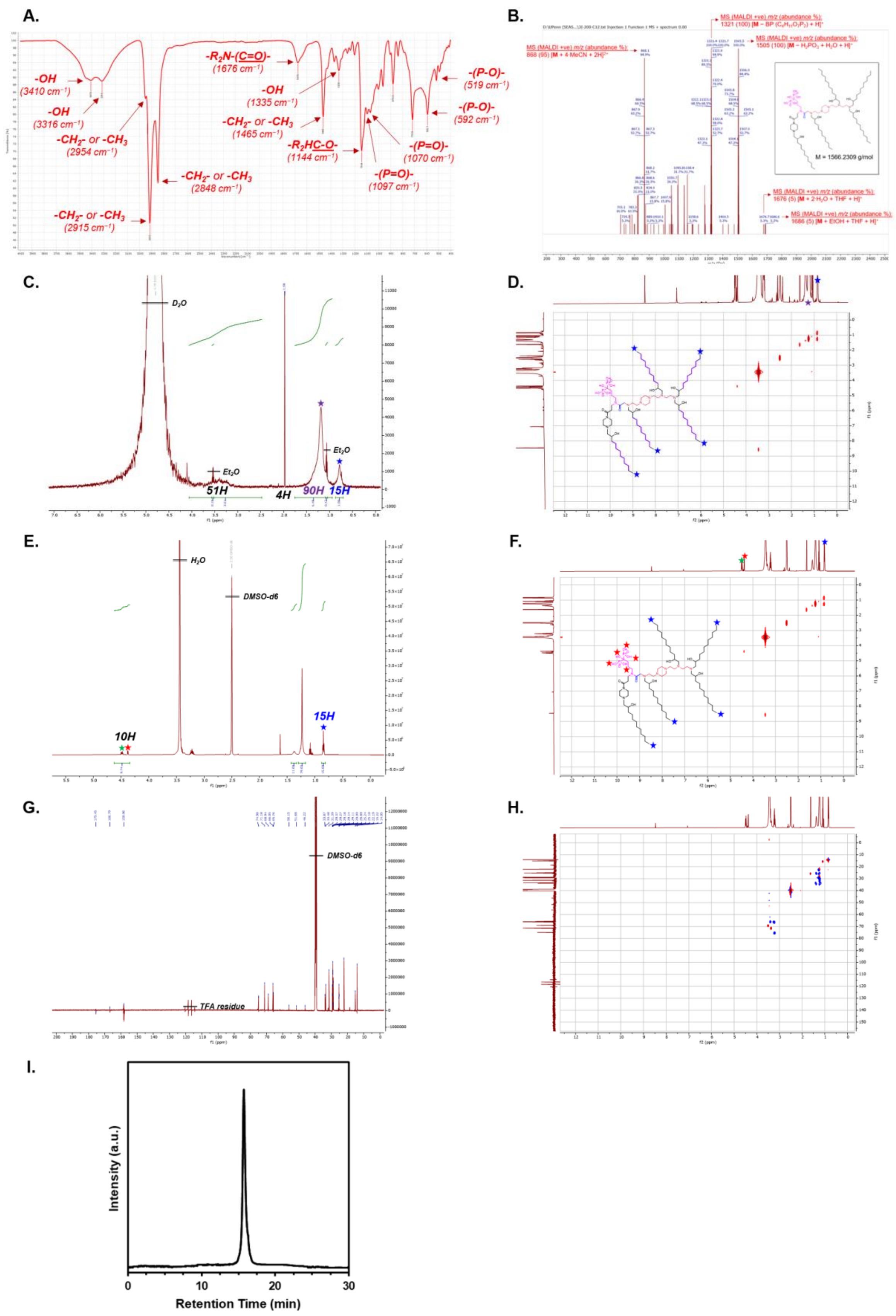

Figure S14. Structural characterization of the '**Type1-P1-C12**' bone-targeting ionizable lipids: (a) FT-IR, (b) MALDI-ToF, (c) ^1H (D_2O), (d) ^1H-^1H COSY (D_2O), (e) ^1H

(DMSO-*d6*), (f) ^{1}H-^{1}H COSY (DMSO-*d6*), (g) ^{13}C (DMSO-*d6*), and (h) ^{1}H-^{13}C HSQC (DMSO-*d6*) NMR spectra. (i) HPLC trace.

3.15. Structural characterization of 'Type3-P1-C12'

White hygroscopic solid. The purity and yield of the compound exceeded 95%. Due to its very low solubility in both aqueous and organic solvents, the structure of the compound was elucidated using two different NMR solvents D_2O and DMSO-*d6*.

FT-IR (ATR mode): 3407 and 3319 [v(-OH)], 2954, 2915, and 2848 [v(-CH_2- and -CH_3)], 1679 [v(R_2N-($\underline{C=O}$)-)], 1464 [v(-CH_2- and CH_3)], 1336 [v(-OH)], 1143 [v($R_2H\underline{C}$-$\underline{O}$-)], 1099 and 1070 [v(-(P=O)-)], 592 and 517 [v(-(P-O)-)] cm^{-1}.

^{1}H NMR (600 MHz, DMSO-*d6*): δ 4.50−4.28 (m, 13H, -OH), 1.44−1.14 (m, 144H, -CH_2- (epoxide-based alkyl chains)), 0.85 (t, 24H, -CH_3, J = 7.0 Hz) ppm.

^{1}H NMR (600 MHz, D_2O): δ 4.00−2.50 (m, 82H, -CH_2- (main structural framework)), 2.03 (m, 4H, -NH-CH_2-$C\underline{H}_2$-$C\underline{H}_2$-), 1.62−1.06 (m, 144H, -CH_2- (epoxide-based alkyl chains)), 0.90−0.76 (m, 24H, -CH_3) ppm.

$^{13}C\{H\}$ NMR (151 MHz, DMSO-*d6*): δ 175.9, 166.8, and 158.2 (-(C=O)-), 75.0 (-C(OH)-(HPO_3)$_2$), 71.1, 68.9, 66.0, and 65.7 (R_2C(OH)-), 56.1, 51.6, and 46.1 (-$\underline{C}H_2$-(C=O)-N($\underline{C}H_2\underline{C}H_2$)$_2$N-(C=O)-, -($CH_2$)$_2NCH_2CH_2$N($CH_2$-)$CH_2CH_2$-N($CH_2CH_2$)$_2NCH_2CH_2$N($CH_2$)$_2$-, and -$\underline{C}H_2$N(-(C=O)-)$\underline{C}H_2CH_2CH_2$C(OH)-($HPO_3$)$_2$), 39.5 (-NH-$\underline{C}H_2$-$CH_2$-$CH_2$-), 33.8, 33.7, 33.4, 31.9, 31.3, 29.3, 29.2, 29.1, 29.0, 28.9, 28.8, 25.6, 25.2, and 25.1 (-CH_2- (epoxide-based alkyl chains)), 31.3 (-NH-CH_2-$\underline{C}H_2$-CH_2-), 22.1 (-NH-CH_2-CH_2-$\underline{C}H_2$-), 15.2 (-$\underline{C}H_2$-C(OH)-(HPO_3)$_2$), 14.0 (-CH_3) ppm.

MS (MALDI +ve) *m/z* (abundance %) for $C_{131}H_{267}N_{13}O_{18}P_2$: calculated $[M + H_2O + 3 \cdot Na]^+$ 820.5102, found $[M + H_2O + 3 \cdot Na]^+$ 820.8733 (100).

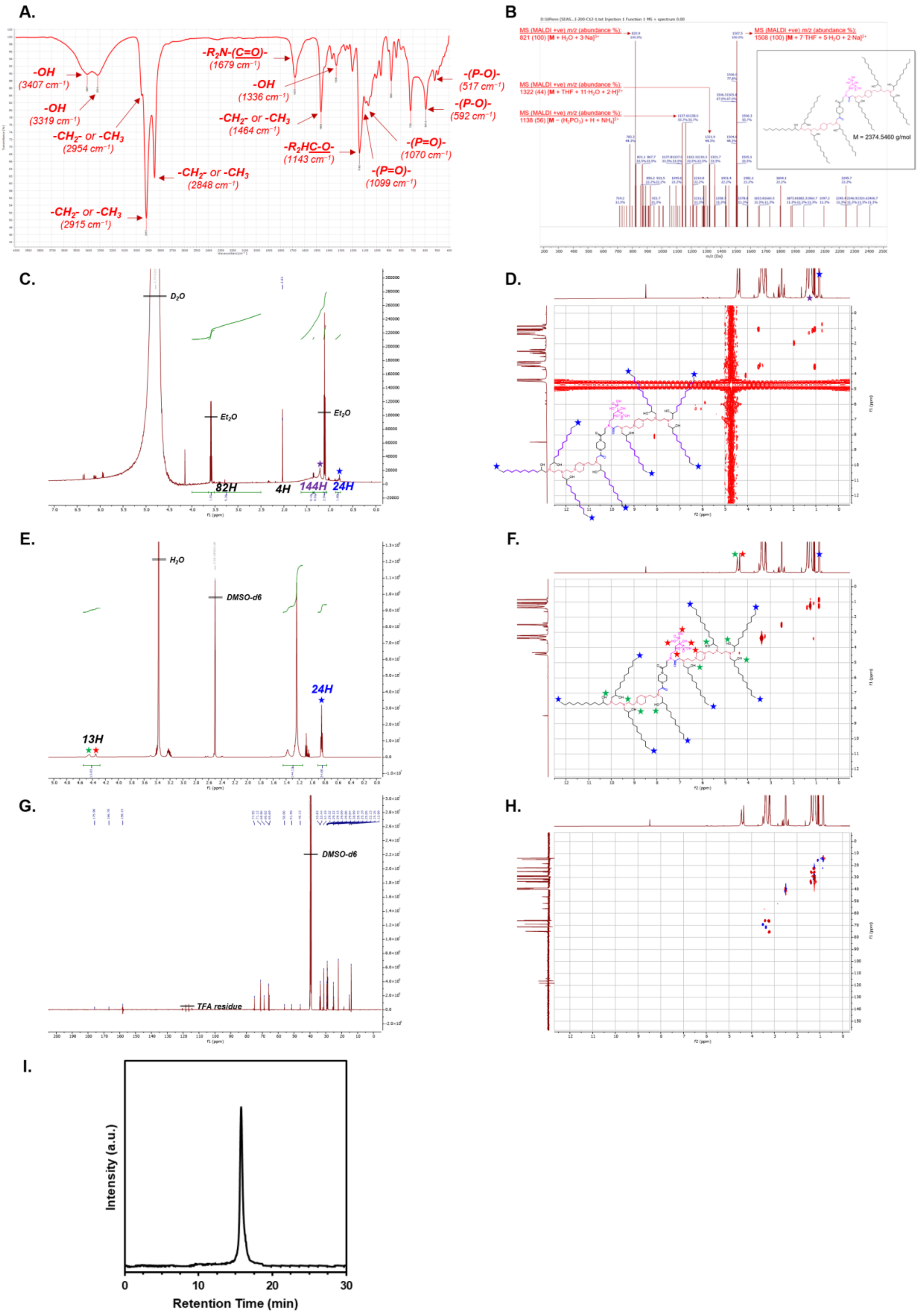

Figure S15. Structural characterization of the '**Type3-P1-C12**' bone-targeting ionizable lipids: (a) FT-IR, (b) MALDI-ToF, (c) ^1H (D_2O), (d) ^1H-^1H COSY (D_2O), (e) ^1H

(DMSO-*d6*), (f) ^1H-^1H COSY (DMSO-*d6*), (g) ^{13}C (DMSO-*d6*), and (h) ^1H-^{13}C HSQC (DMSO-*d6*) NMR spectra. (i) HPLC trace.

4. Biological assays

4.1. LNP formulation

The LNPs utilized in this investigation were synthesized through the following procedure: An ethanol phase comprising all lipid constituents and an aqueous phase containing FLuc mRNA were combined using a microfluidic device to fabricate LNPs. The ethanol phase consisted of either PIP-BP ionizable lipid or standard ionizable lipid, along with DOPE, cholesterol, and C14-PEG2000 in fixed molar ratios of 35% (2.20 × e^{-07} mol), 16% (1.01 × e^{-07} mol), 46.5% (2.92 × e^{-07} mol), and 2.5% (1.57 × e^{-08} mol), respectively. The fifth component LNP formulation used an ethanol phase containing C12-200 ionizable lipid, PIP-BP ionizable lipid, DOPE, cholesterol, and C14-PEG2000 with the fixed molar ratio of 35% for the ionizable lipids. The molar amount for the ionizable lipid is allocated according to the specified percentages of the components in the formulation. The aqueous phase contained mRNA dissolved in a 10 mM citrate buffer. Subsequently, the ethanol and aqueous phases were blended at flow rates of 1.8 ml/min and 0.6 ml/min (in a 3:1 ratio) using Pump33DS syringe pumps. The resulting LNPs underwent dialysis in 1x PBS using a microdialysis cassette (MWCO = 20,000, ThermoFisher Scientific, Waltham, MA) for 2 hours followed by filtration through a 0.22 µm filter. The *Z*-average diameters, polydispersity index (PDI), and Zeta potential were assessed using a DynaPro™ Plate Reader III or Malvern Zetasizer Nano ZS instrument. Additionally, the mRNA concentration and encapsulation efficiency in each LNP formulation were determined via a modified Quant-iT RiboGreen assay (ThermoFisher Scientific) conducted on a plate reader.

→ There are three main limitations to consider when modifying the terminal group of PEG units for specific targeting purposes. First, from a chemical synthesis and analytical perspective: Both PEG2000 and bisphosphonates exhibit high solubility in water. Furthermore, alendronate typically exists in three hydrate forms and dissolves exclusively in water. However, achieving a chemical bond between the terminal group of PEG2000 and alendronate requires initial chemical bonding in an organic solvent, posing a significant challenge. Our synthetic approach involves adjusting the pH of alendronate, followed by maintaining a ratio of dH_2O/Ethanol (*v/v* = 1/0.9) during chemical synthesis, while avoiding precipitation, which can occur in certain solvent conditions. Second, modifying the terminal group of the PEG unit presents limitations. The linear alkyl chain of the PEG unit maintains skeletal structure up to C5 through lipid stacking, but beyond this point, it tends to fold. We rationally designed PIP-BP ionizable lipids to achieve a more stable presentation on the exterior of the LNP, as evidenced by simulation results shown in Figure S16.

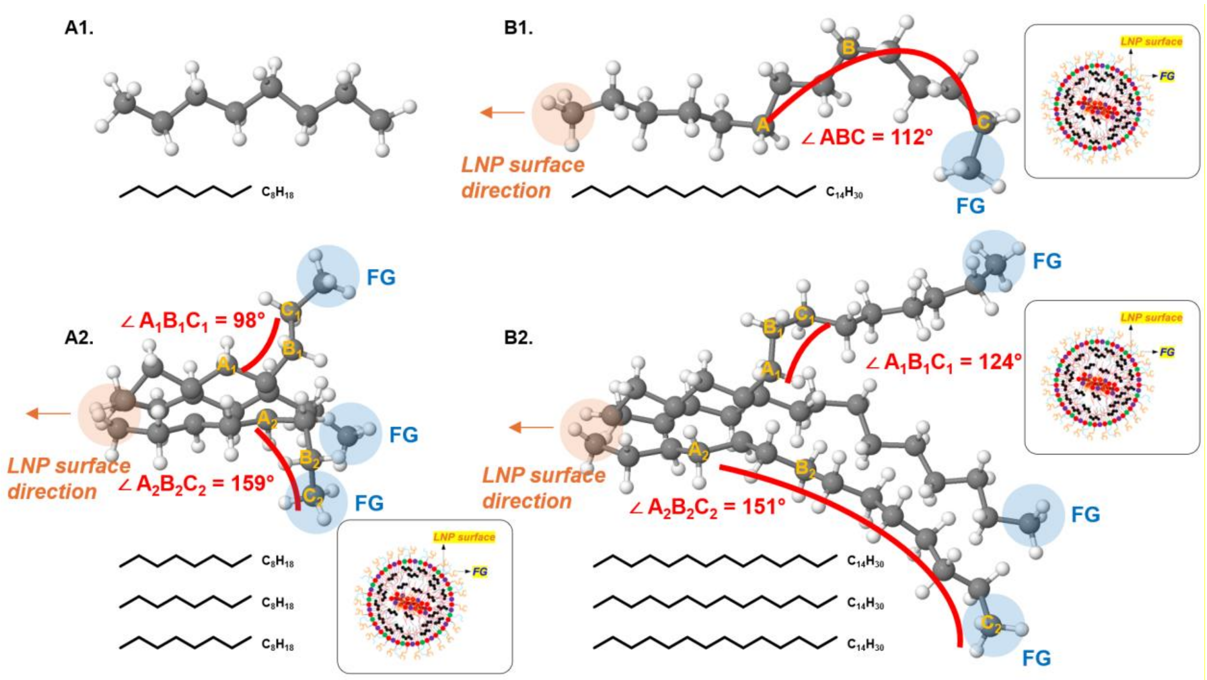

Figure S16. Computed structures of (a) C8 and (b) C14 linear alkyl chains. (a1) C_8H_{18}, (a2) 3 × C_8H_{18}, (b1) $C_{14}H_{30}$, and (b2) 3 × $C_{14}H_{30}$ alkyl chains on LNP surface. Grey balls: carbon atoms, white balls: hydrogen atoms. FG: Functional group. All the chemical structures were calculated based on an empirical force field model (MM2).

Third, characterizing PEG units after modifying the terminal group presents challenges, since it has a molecular weight distribution. Modification of ionizable lipids is more facile for physicochemical analysis of chemical bonds compared to modification of DSPE-PEG2000. MALDI-ToF shows the trends in polymer distribution, which makes it more difficult to identify specific chemical changes.

4.2. FLuc mRNA *in vitro* delivery screening

Hep-G2 or BJ cells were seeded at a density of 5,000 cells per well in 96-well plates with transparent bottoms and white walls. Each well contained 100 µL of growth medium, consisting of DMEM supplemented with 10% FBS and 1% P/S for Hep-G2 cells, and DMEM supplemented with 10% FBS for BJ cells. The plates were then incubated at 37 °C in a 5% CO_2 atmosphere. Following this, the growth medium was replaced with fresh medium, and LNPs were administered at a dosage of 5 ng FLuc mRNA per well. After 24 hours of post-LNP transfection, firefly luciferase expression was quantified using the Luciferase Assay System (Promega) as per the manufacturer's instructions. The luminescent signal was normalized against cells treated with growth medium alone. Cell viability was assessed using the CellTiter-Glo Luminescent Cell Viability Assay (Promega), following the manufacturer's protocol.

4.3. Characterization of bone-targeted LNPs-HA interaction *in vitro*

Cells were seeded at a density of 20,000 cells per well in a 12-well plate, with each well containing 400 μL of growth medium consisting of 10% FBS in DMEM. The plate was then incubated at 37 °C in a 5% CO_2 atmosphere. Similarly, each chamber was filled with 200 μL of growth medium consisting of 10% FBS in DMEM and placed into a separate 12-well plate. These chambers in the plate were also incubated at 37 °C with a 5% CO_2 concentration. Subsequently, the growth medium in the 12-well plate was replaced with fresh medium, while the medium in the chambers was replaced with fresh medium containing LNPs at a concentration of 40 ng FLuc mRNA. After 24 hours of post-LNP transfection, the chambers were removed, and firefly luciferase expression in the adherent cells at the bottom of the 12-well plate was quantified using the Luciferase Assay System (Promega) according to the manufacturer's instructions. The luminescent signal was normalized against cells treated with growth medium alone. Cell viability was evaluated using the CellTiter-Glo Luminescent Cell Viability Assay (Promega), following the manufacturer's protocol.

4.4. FLuc mRNA *in vivo* delivery screening

All experimental procedures involving female C57BL/6J mice aged 6-8 weeks were conducted under the guidelines approved by the Institutional Animal Care & Use Committee (IACUC) of the University of Pennsylvania. Mice received a single intravenous injection of FLuc mRNA LNPs at a dosage of 0.5 mg/kg via the tail vein. The expression of luciferase was assessed using an IVIS Spectrum imaging system (Caliper Life Sciences) 12 hours post-injection. Subsequently, mice were injected with *D*-luciferin (PerkinElmer) at a dose of 150 mg/kg via intraperitoneal injection (IP). After a 10-minute incubation under anesthesia, bioluminescence intensity was quantified by measuring photon flux in the region of interest using Living IMAGE Software provided by Caliper. *Ex vivo* imaging was conducted on the heart, liver, spleen, lung, kidney, forelimbs, and legs after resection.

5. Supplementary figures

5.1. Cryo-TEM analysis

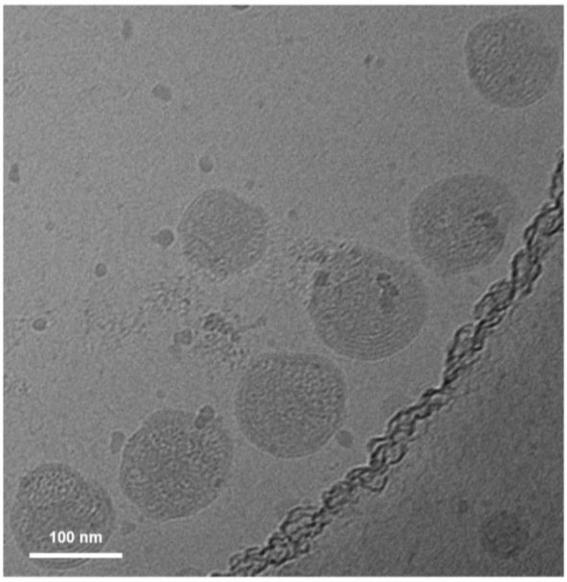

Figure S17. TEM image of bone-targeted LNPs composed of the '**Type3-P1-C12** (10%)' and '**C12-200** (90%)' ionizable lipids.

5.2. Solubility testing of compounds linked with alendronate

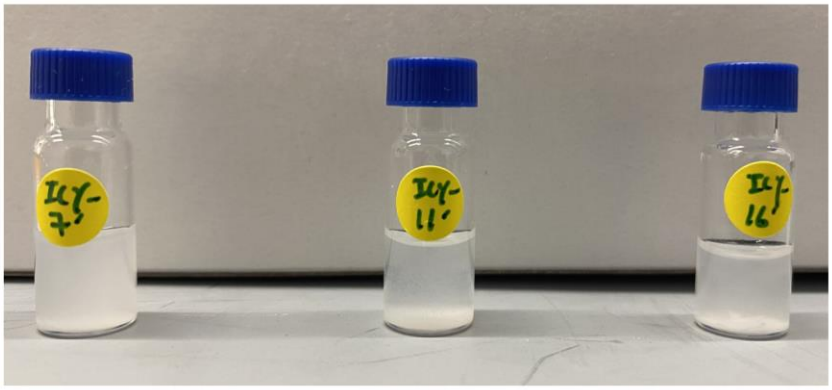

Figure S18. Solubility of the '**Product 4**', '**Product 7**', and '**Product 3**' (Left to right) in ethanol.

5.3. LC-UV diode-array detection for alendronate

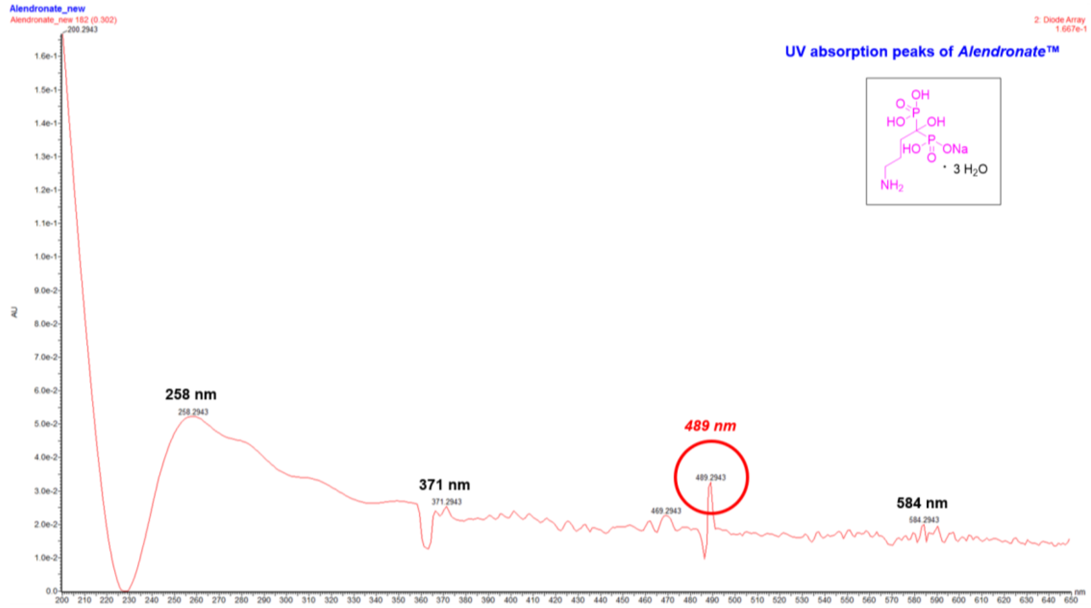

Figure S19. UV absorption peaks of alendronate.

5.4. Effects of LNP formulation changes on *in vitro* experimental results

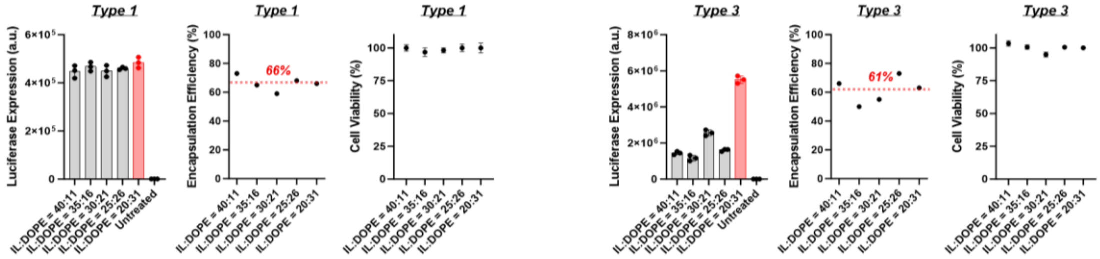

Figure S20. Changes in the physicochemical properties of bone-targeted LNPs, consisting of 100% bone-targeting ionizable lipid ('**Type1-P1-C12**' or '**Type3-P1-C12**'), C14-PEG2000, and DOPE lipids, and their influence on encapsulation efficiency, luciferase expression, and cell viability in the Hep-G2 cell line. Cholesterol:C14-PEG2000 = 46.5:2.5%, and 5,000 cells of the BJ cell line and 10 ng of FLuc mRNA were treated in each well. Error bars represent SEM.

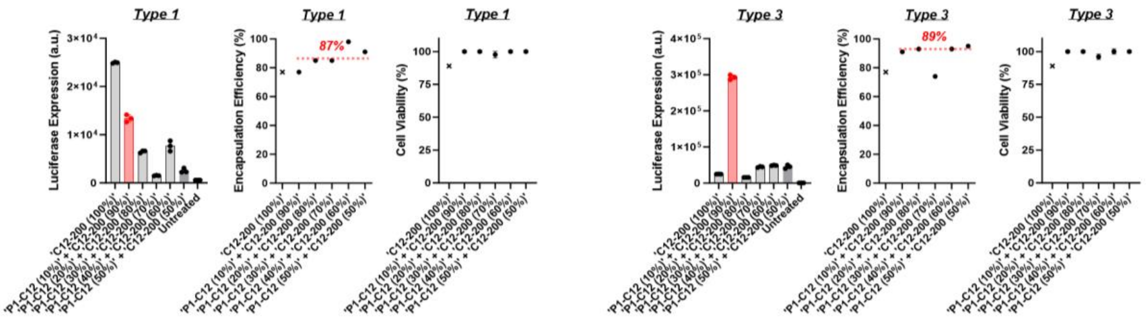

Figure S21. Changes in the physicochemical properties of bone-targeted LNPs, consisting of bone-targeting ionizable lipid ('**Type1-P1-C12**' or '**Type3-P1-C12**') mixed with '**C12-200**' lipid, C14-PEG2000, and DOPE lipids, and their influence on encapsulation efficiency, luciferase expression, and cell viability in the Hep-G2 cell line. Cholesterol:C14-PEG2000 = 46.5:2.5%, and 5,000 cells of the BJ cell line and 10 ng of FLuc mRNA were treated in each well. Error bars represent SEM.

5.5. Impact of 'C12-200' on the morphology of bone-targeted LNPs

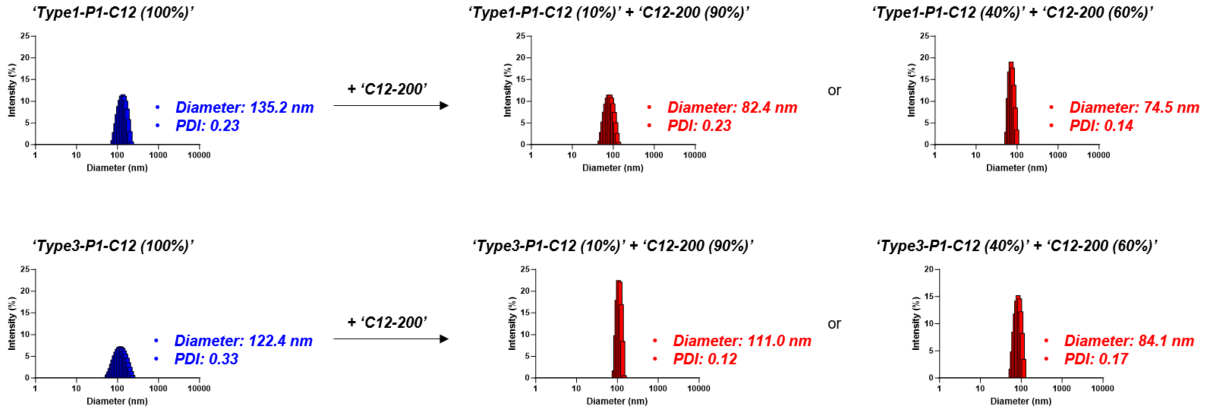

Figure S22. Change in hydrodynamic diameters based on the composition of ionizable lipids, and the bone-targeted LNPs were formulated with the following ratios: Ionizable lipid(s)/DOPE/Cholesterol/C14-PEG2000 = 35/16/46.5/2.5%.

5.6. Bone-targeted LNPs adsorption on hydroxyapatite experiments

A.

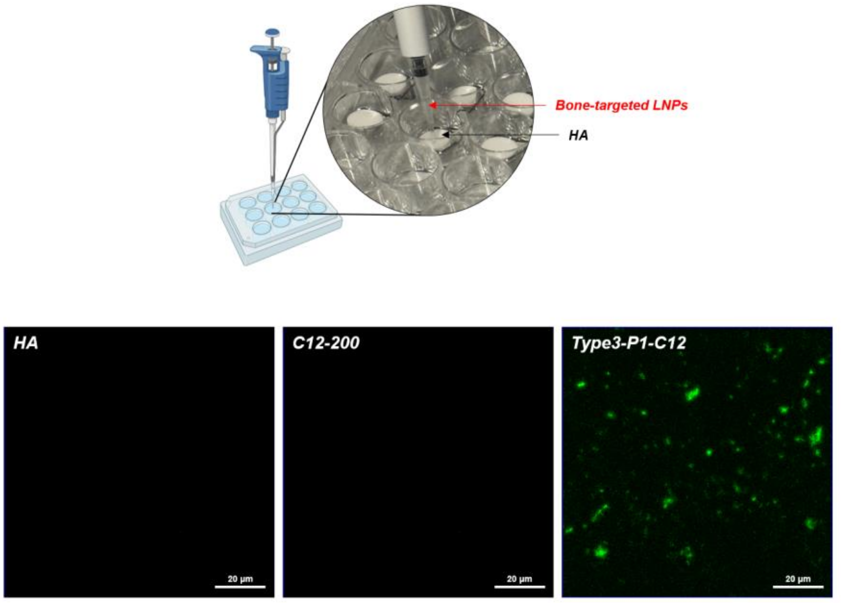

B1.

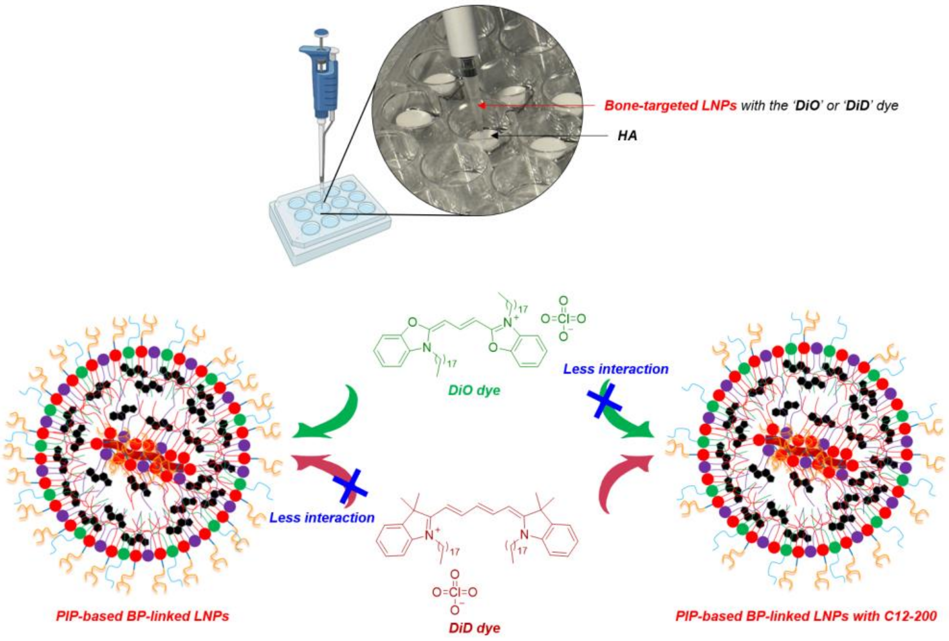

B2.

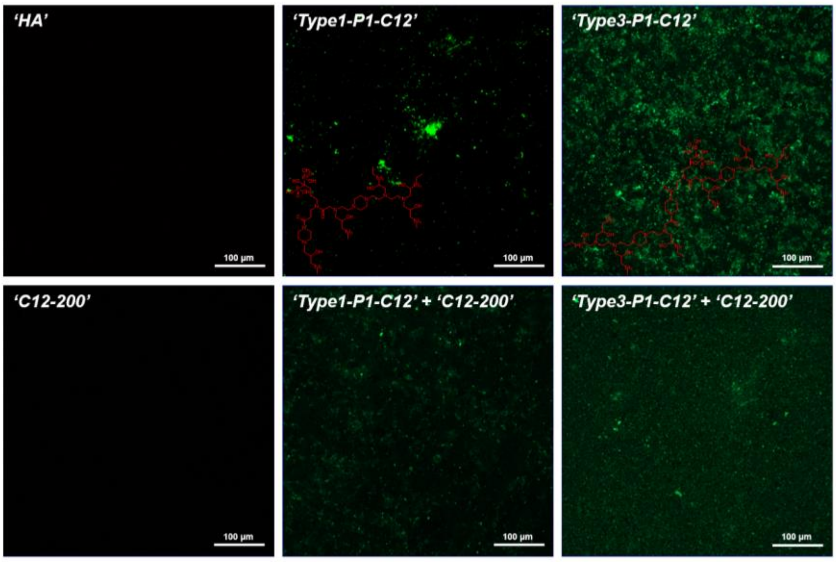

B3.

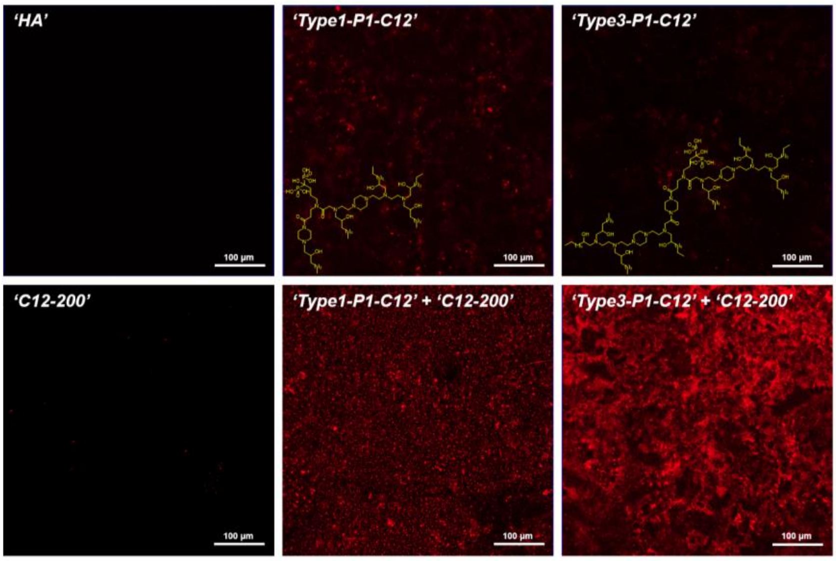

Figure S23. Binding study on hydroxyapatite using the bone-targeted LNPs. (a1) Confocal microscopy images. (b1) Pictorial representation for the Interaction between the fluorescence dyes (DiO or DiD) and the surface of bone-targeted LNPs. Images from confocal microscopy of bone-targeted LNPs stained with (b2) DiO and (b3) DiD dyes. The bone-targeted LNPs were formulated with the following ratios: Ionizable lipid(s)/DOPE/Cholesterol/C14-PEG2000 = 35/16/46.5/2.5%.

5.7. Hep-G2 cell line *in vitro* screening results

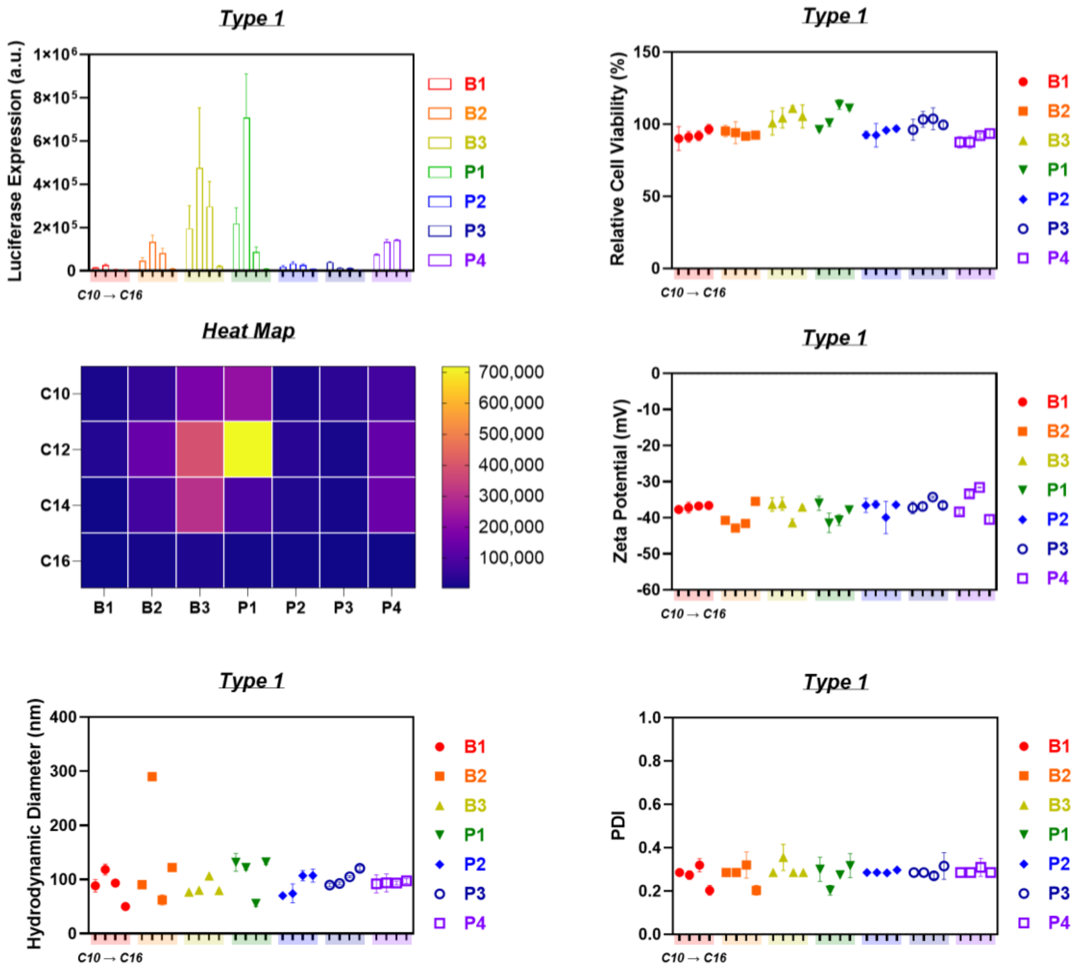

Figure S24. *In vitro* screening results in Hep-G2 cell line and the physicochemical properties of bone-targeted LNPs with '**Type 1**' ionizable lipids. 5,000 cells of the Hep-G2 cell line and 10 ng of FLuc mRNA were treated in each well. Error bars represent SEM.

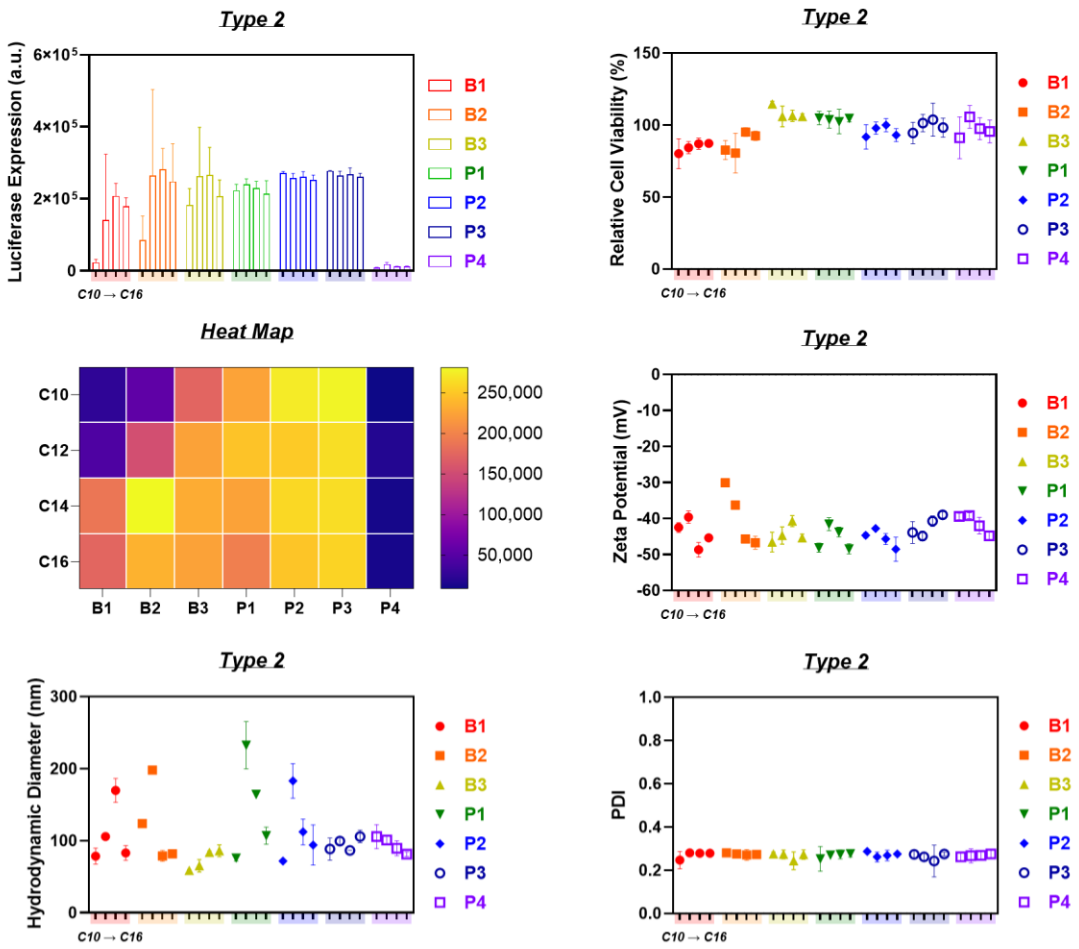

Figure S25. *In vitro* screening results in Hep-G2 cell line and the physicochemical properties of bone-targeted LNPs with '**Type 2**' ionizable lipids. 5,000 cells of the Hep-G2 cell line and 10 ng of FLuc mRNA were treated in each well. Error bars represent SEM.

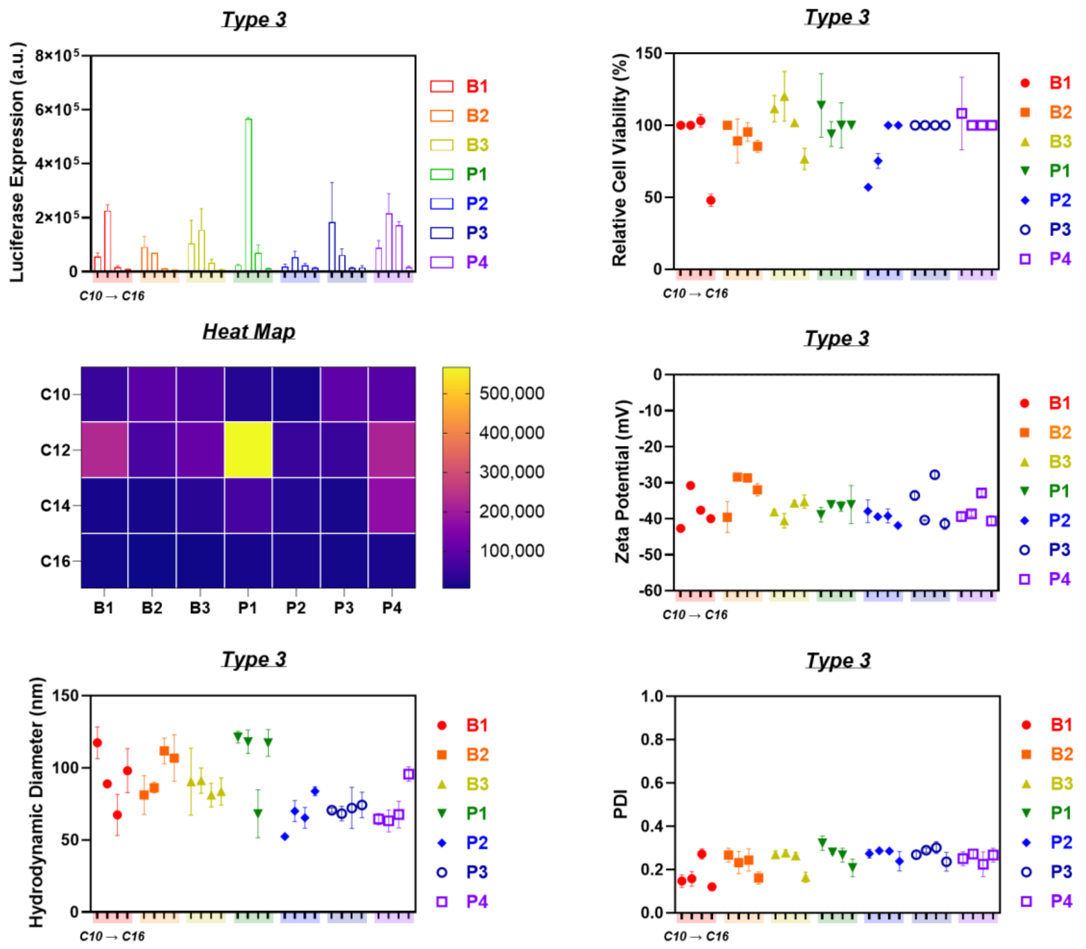

Figure S26. *In vitro* screening results in Hep-G2 cell line and the physicochemical properties of bone-targeted LNPs with '**Type 3**' ionizable lipids. 5,000 cells of the Hep-G2 cell line and 10 ng of FLuc mRNA were treated in each well. Error bars represent SEM.

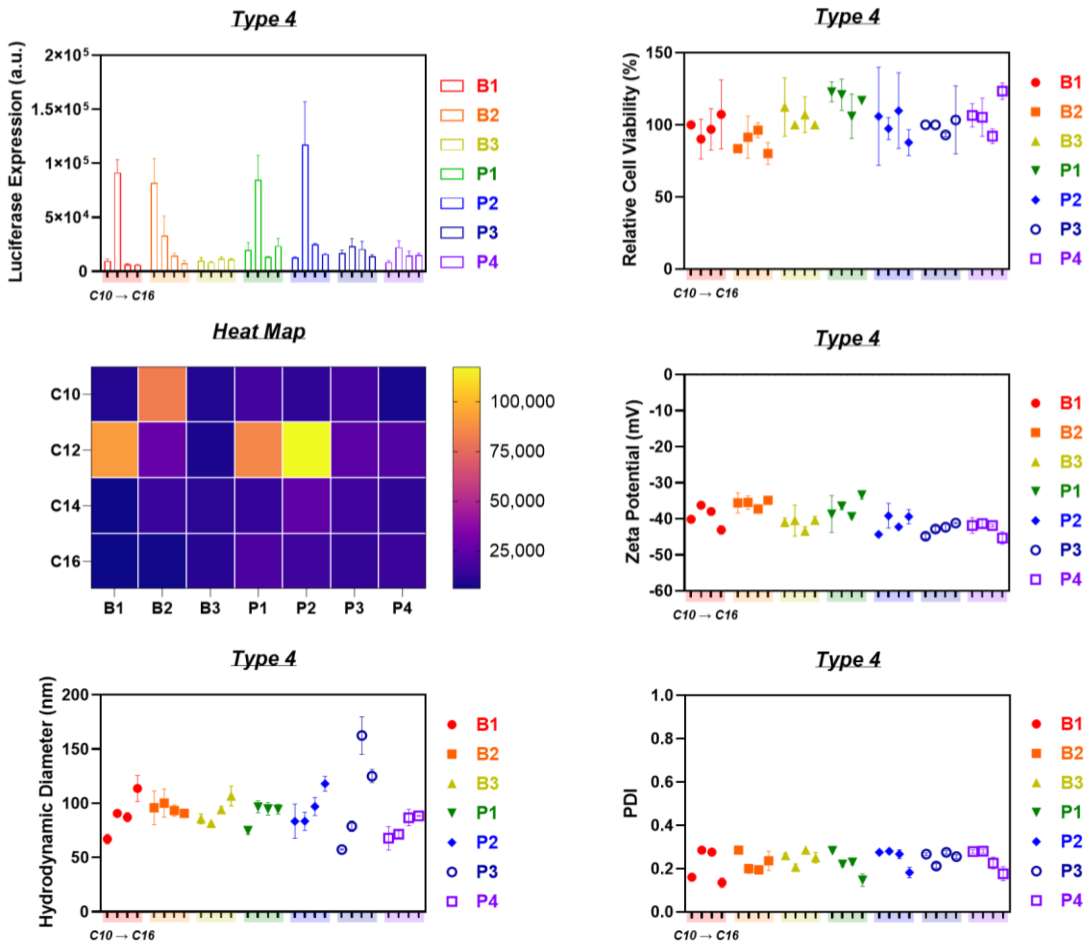

Figure S27. *In vitro* screening results in Hep-G2 cell line and the physicochemical properties of bone-targeted LNPs with '**Type 4**' ionizable lipids. 5,000 cells of the Hep-G2 cell line and 10 ng of FLuc mRNA were treated in each well. Error bars represent SEM.

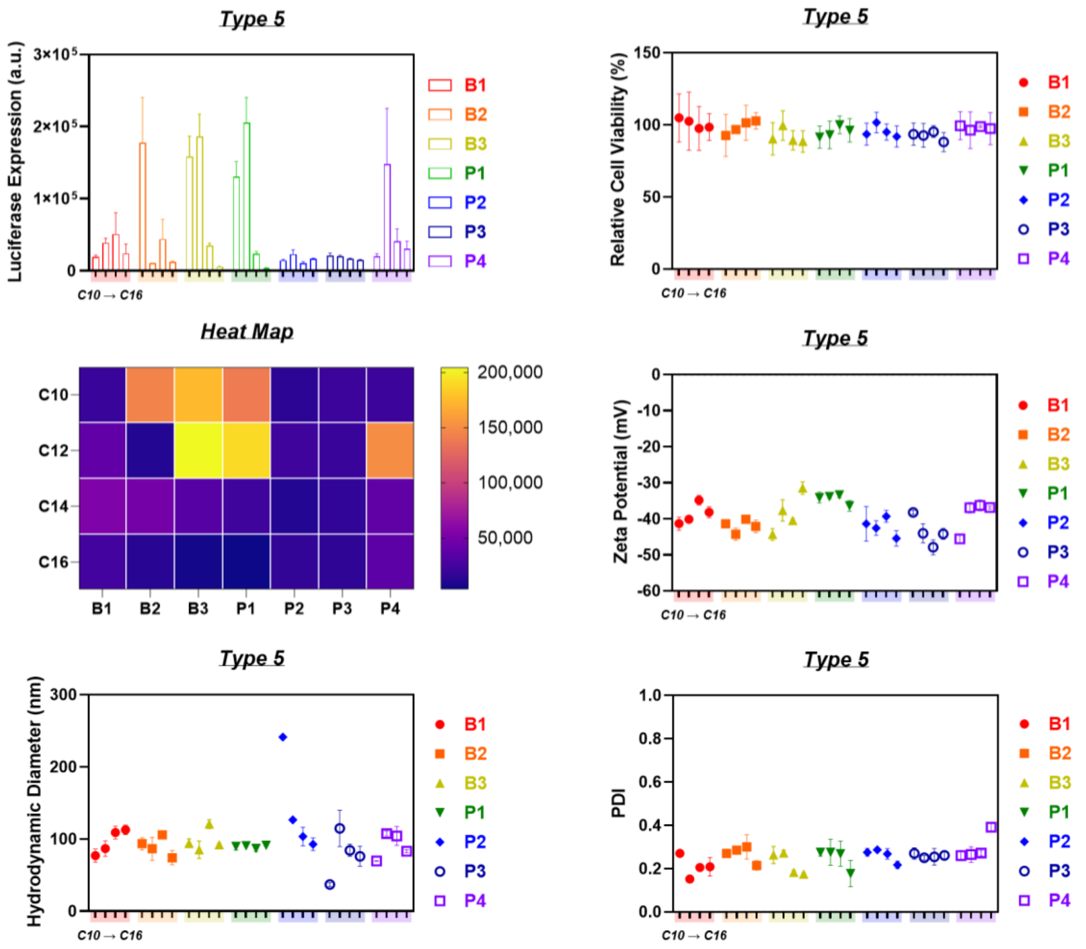

Figure S28. *In vitro* screening results in Hep-G2 cell line and the physicochemical properties of bone-targeted LNPs with '**Type 5**' ionizable lipids. 5,000 cells of the Hep-G2 cell line and 10 ng of FLuc mRNA were treated in each well. Error bars represent SEM.

5.8. BJ cell line *in vitro* screening results

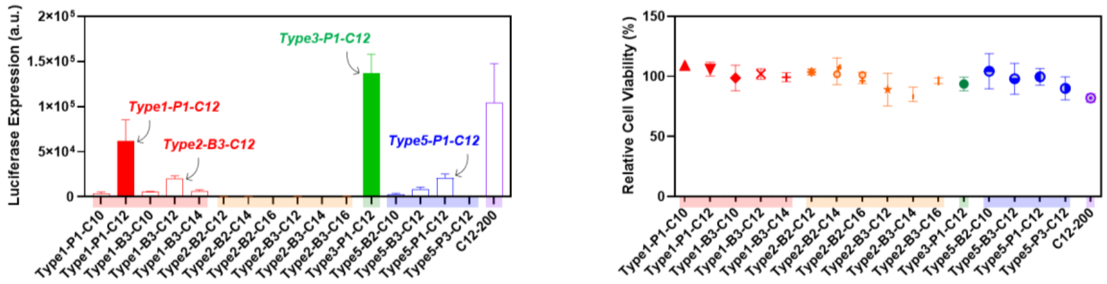

Figure S29. *In vitro* screening results and cell viability of the top-performing 16 bone-targeting ionizable lipids in the Hep-G2 cell line. 5,000 cells of the BJ cell line and 10 ng of FLuc mRNA were treated in each well. Error bars represent SEM.

5.9. mRNA dose-dependent transfection experiments

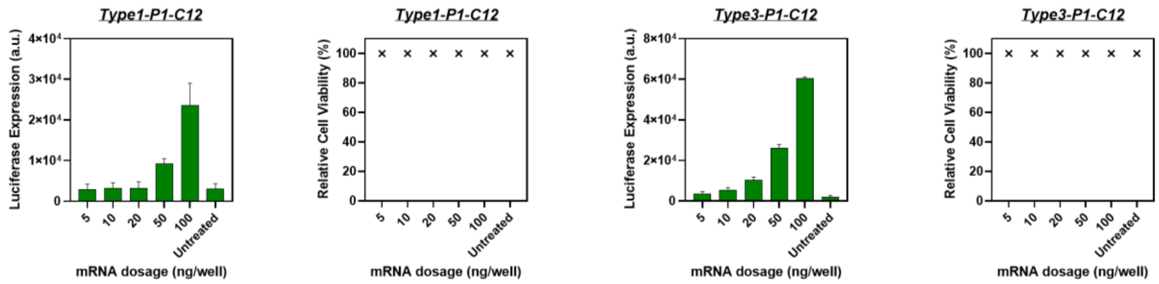

Figure S30. *In vitro* experiments for the bone-targeted LNPs based on the '**Type1-P1-C12** (100%)' and '**Type3-P1-C12** (100%)' bone-targeting ionizable lipids in the Hep-G2 cell line. 5,000 cells of the Hep-G2 cell line were treated in each well. Error bars represent SEM.

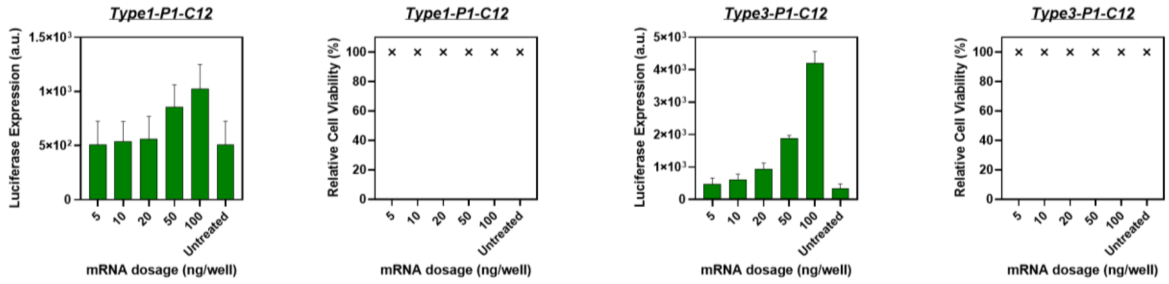

Figure S31. *In vitro* experiments for the bone-targeted LNPs based on the '**Type1-P1-C12** (100%)' and '**Type3-P1-C12** (100%)' bone-targeting ionizable lipids in the BJ cell line. 5,000 cells of the BJ cell line were treated in each well. Error bars represent SEM.

5.10. Comparative study between previous and current bone-targeting LNPs

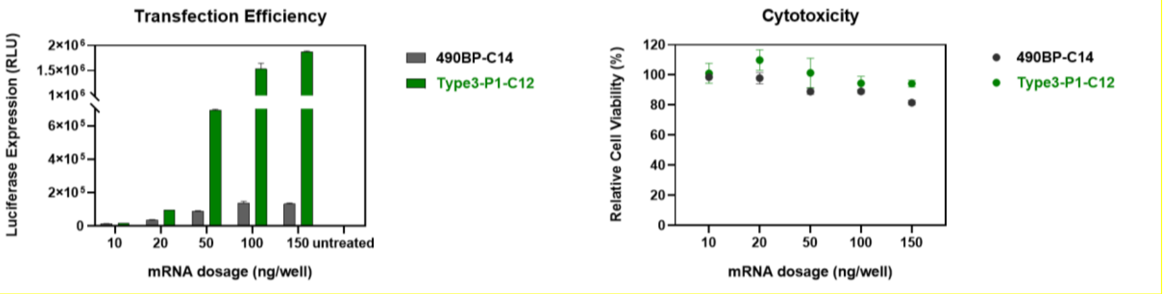

Figure S32. Comparison of the previous ('**490BP-C14**[11]') and current ('**Type3-P1-C12**') bone-targeting LNPs *in vitro* experiments. 5,000 cells of the BJ cell line were treated in each well. Error bars represent SEM.

5.11. *In vivo* bioluminescence imaging

A.

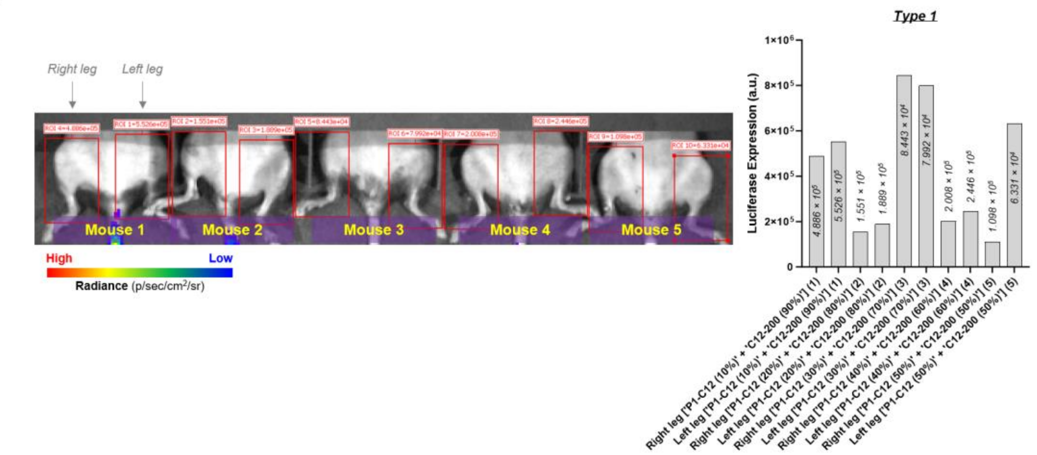

B.

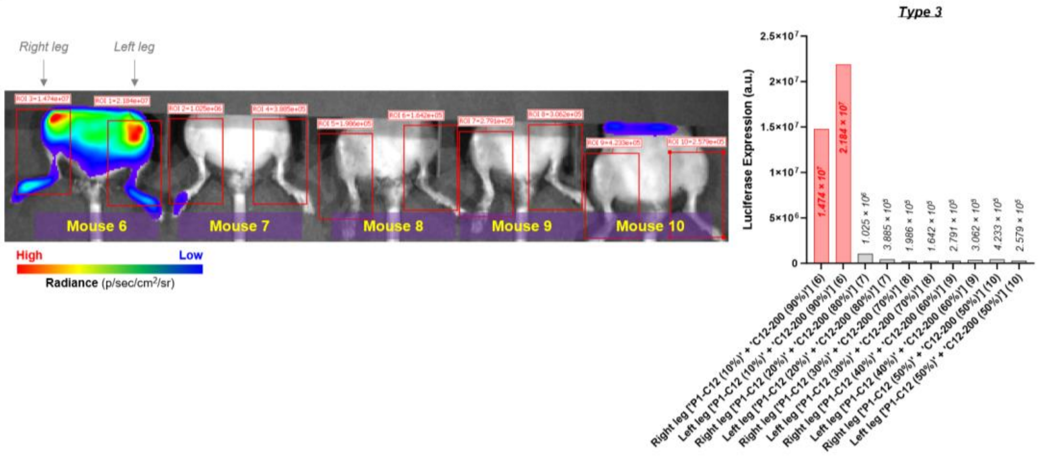

Figure S33. Luciferase expression changes with formulation variations of the bone-targeted LNPs composed of bone-targeting ionizable lipid ((a) '**Type1-P1-C12**' or (b) '**Type3-P1-C12**') mixed with '**C12-200**' lipid. Cholesterol:C14-PEG2000 = 46.5:2.5%.

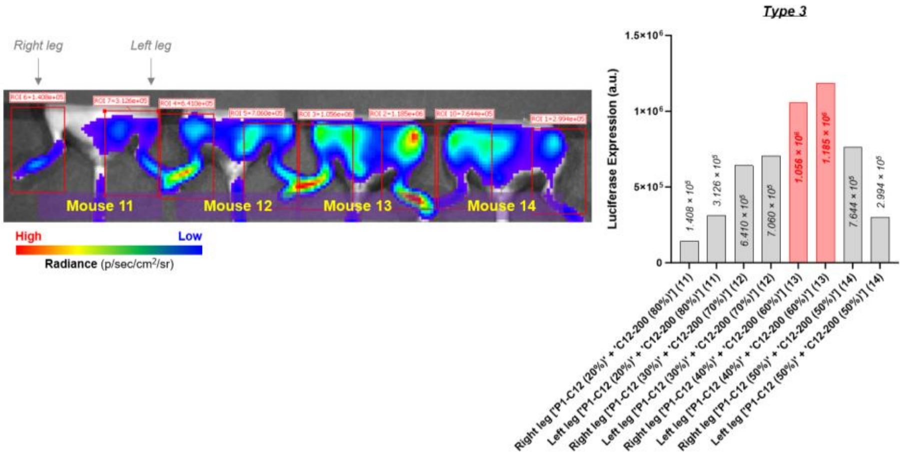

Figure S34. Luciferase expression of bone-targeted LNPs excluding those composed of 10% bone-targeting ionizable lipid ('**Type3-P1-C12**') mixed with '**C12-200**' lipid, with up to 50% bone-targeting ionizable lipid.

5.12. *Ex vivo* bioluminescence imaging

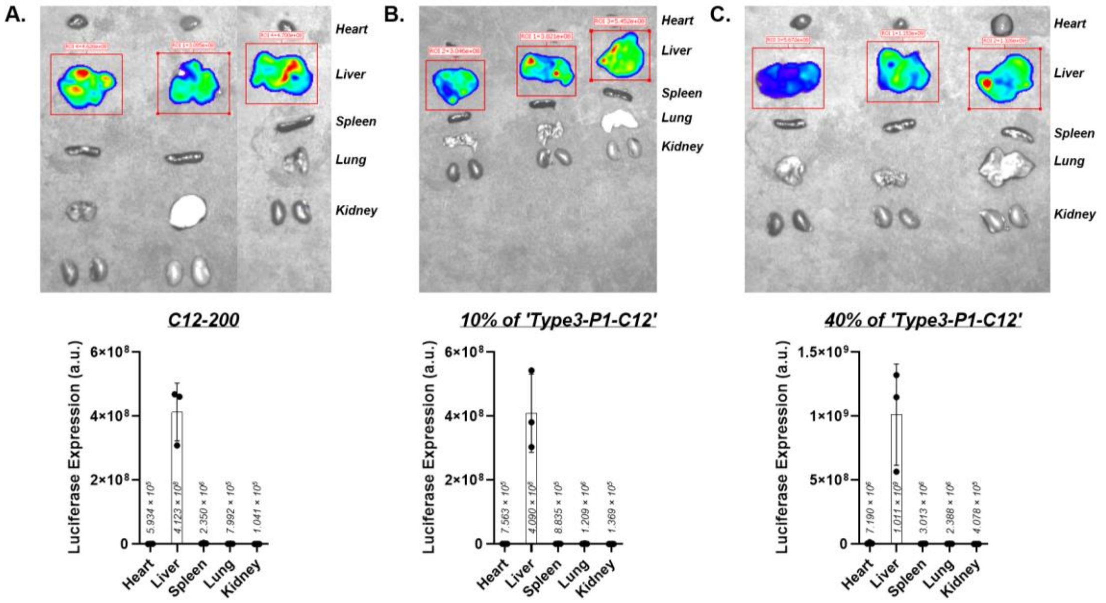

Figure S35. Luciferase expression in 5 different mouse organs (heart, liver, spleen, lung, and kidney) treated with bone-targeted LNPs based on (a) '**C12-200** (100%)', (b) '**Type3-P1-C12** (10%)' + '**C12-200** (90%)', and (c) '**Type3-P1-C12** (40%)' + '**C12-200** (60%)' ionizable lipids. Error bars represent SEM.

5.13. H&E-stained histological images of mouse leg bone tissue

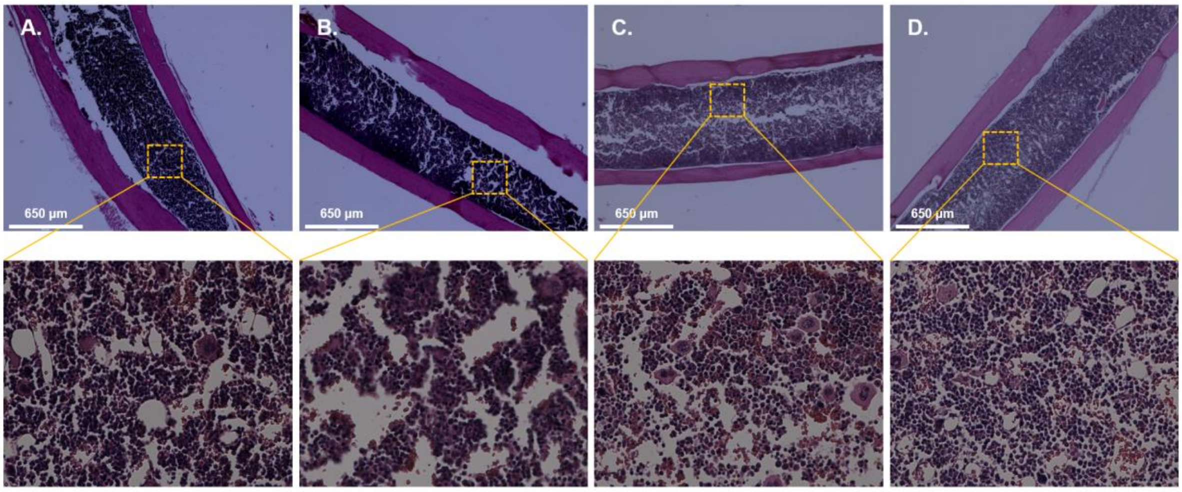

Figure S36. Representative H&E staining of leg tissue from mice treated with (a) PBS, LNPs composed of (b) '**C12-200** (100%)', (c) '**Type3-P1-C12** (10%)' + '**C12-200** (90%)', and (d) '**Type3-P1-C12** (40%)' + '**C12-200** (60%)' lipids.

5.14. H&E-stained histological images of mouse organ tissue

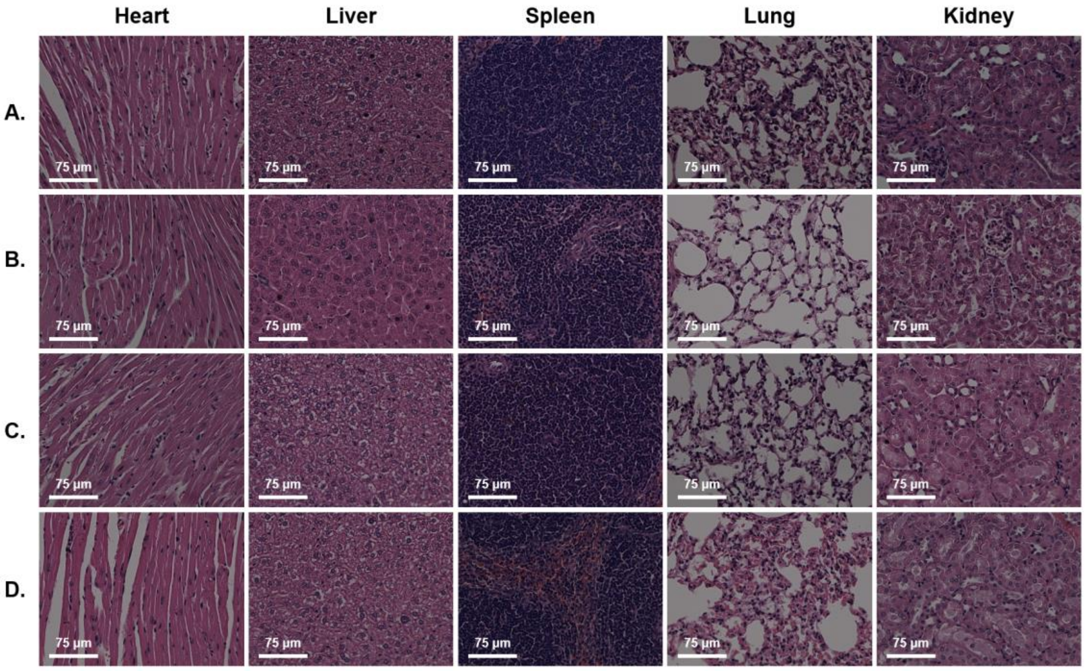

Figure S37. Representative H&E staining of 5 different organ tissues (Heart, liver, spleen, lung, and kidney) from mice treated with (a) PBS, LNPs composed of (b) '**C12-200** (100%)', (c) '**Type3-P1-C12** (10%)' + '**C12-200** (90%)', and (d) '**Type3-P1-C12** (40%)' + '**C12-200** (60%)' lipids.

6. Computed structure coordinates

6.1. Overall computational results

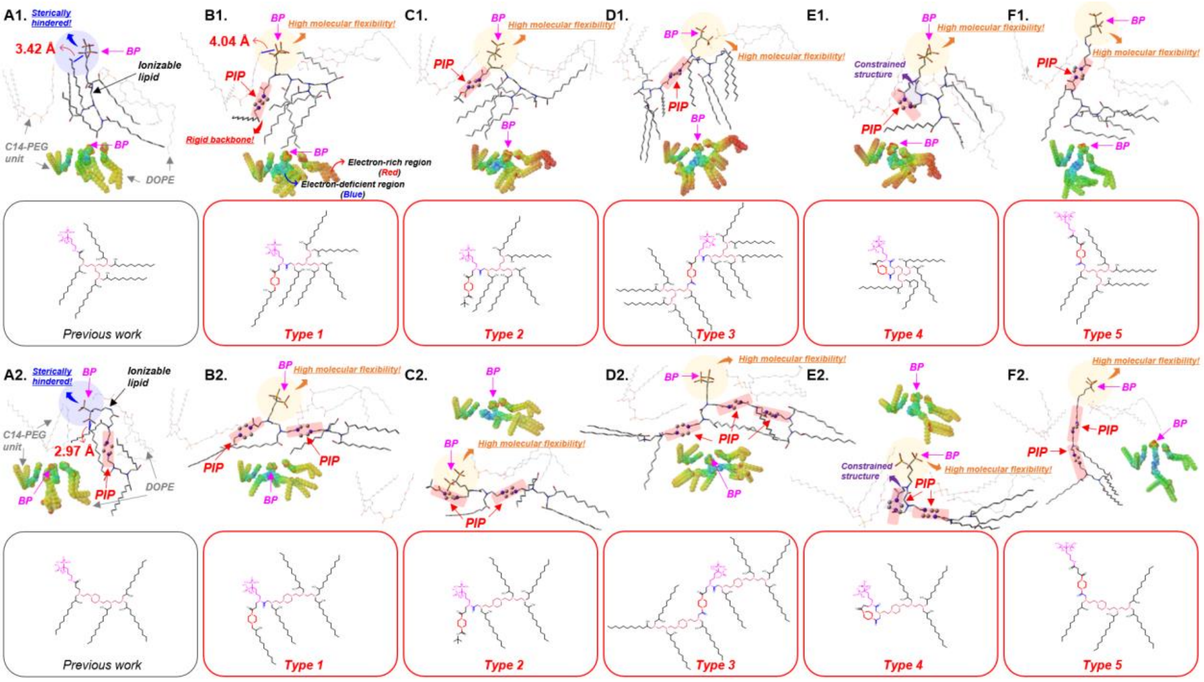

Figure S38. Computed relations among 3 different component lipids (‘C14-PEG unit’, ‘bone-targeting ionizable lipid’, and ‘DOPE’). (a1, b1, c1, d1, e1, and f1) ‘Branched’ and (a2, b2, c2, d2, e2, and f2) ‘Piperazine’ amine core based bone-targeting ionizable lipids between ‘C14-PEG unit’ and ‘DOPE’ lipids. (a) Previous bone-targeting ionizable lipids. Newly developed (b) ‘**Type 1**’, (c) ‘**Type 2**’, (d) ‘**Type 3**’, (e) ‘**Type 4**’, and (f) ‘**Type 5**’ bone-targeting ionizable lipids. 'BP' denotes bisphosphonate, and 'PIP' denotes piperazine.

6.2. Previous branched amine core based bone-targeting ionizable lipids

<u>Previous-B1-C12</u>^{REF}

P	-13.5626150000	-3.8235200000	-0.7091240000
P	9.6764520000	10.8253530000	0.8123220000
P	1.0240800000	9.2130490000	1.0936960000
P	-0.1032080000	9.7159740000	-1.6183320000
O	-11.2954500000	6.3269020000	1.9519720000
O	-11.3739130000	1.4010250000	2.7870590000
O	-14.1810730000	-2.9866830000	-2.1016770000
O	-12.8401500000	-5.0081740000	-1.2464060000
O	-12.6992070000	-2.8901270000	0.2725890000

1	O	-15.0305890000	-4.2399070000	-0.1223400000
2	O	-17.6020590000	-4.9635210000	-0.1562920000
3	O	-16.9665190000	-6.1585530000	-1.9942340000
4	O	-16.3781250000	-5.8753740000	3.0846540000
5	O	-15.8153250000	-6.2884710000	5.2264150000
6	O	-11.3785930000	2.8405930000	1.0514610000
7	O	10.0086060000	4.9826370000	3.5465010000
8	O	10.3740980000	5.1828220000	1.3060170000
9	O	9.1146180000	4.5583170000	-2.5184620000
10	O	9.2877660000	6.2441570000	-1.0449200000
11	O	8.0738180000	11.3458800000	0.3920010000
12	O	10.1099160000	9.3101710000	0.6552400000
13	O	10.4410700000	12.0154230000	0.0006400000
14	O	9.6494960000	11.4012550000	2.1834610000
15	O	-1.5492080000	8.8978210000	0.4015790000
16	O	2.3810100000	8.7161220000	0.6479350000
17	O	1.0600790000	10.9031250000	1.1902560000
18	O	0.6859930000	8.5844110000	2.6295830000
19	O	1.3086640000	9.6383940000	-2.1570790000
20	O	-1.1922870000	9.1804430000	-2.8005450000
21	O	-0.4669930000	11.3237090000	-1.2356440000
22	O	-0.2835250000	1.5808440000	1.9297440000
23	O	-4.3503950000	-6.1205560000	-2.8441570000
24	O	-2.8541270000	-3.3178050000	-7.9273310000
25	O	0.9951970000	-9.1840690000	-0.6532640000
26	O	3.4408130000	-4.7799240000	1.7891330000
27	O	2.6051380000	3.6302770000	-4.7206840000
28	N	-11.6416560000	0.5844730000	0.7000900000
29	N	8.9964170000	13.2870190000	1.5030850000
30	N	-0.2473710000	3.7770400000	1.1692680000

1	N	-1.7828620000	-3.8728920000	-4.3909830000
2	N	2.5695910000	-5.8233630000	-0.7865680000
3	N	1.1185910000	-2.6221010000	-2.2530060000
4	N	1.0733470000	1.1633250000	-2.4353550000
5	C	-11.2823490000	7.5498900000	1.2470530000
6	C	-11.3564320000	5.2171430000	1.0894700000
7	C	-11.3583430000	3.9428990000	1.9210750000
8	C	-21.0997680000	4.9887010000	16.3072510000
9	C	-20.4370730000	3.6655490000	15.9016590000
10	C	-20.4203450000	3.4780330000	14.3762030000
11	C	-19.7558800000	2.1534940000	13.9670740000
12	C	-20.8034670000	1.9336600000	-16.0992100000
13	C	-20.1971880000	0.7697170000	-15.3039940000
14	C	-20.2894620000	1.0030210000	-13.7875570000
15	C	-19.6845600000	-0.1629720000	-12.9888030000
16	C	-19.7796760000	0.0660680000	-11.4713930000
17	C	-19.7393170000	1.9634360000	12.4414060000
18	C	-12.6254600000	-1.5138830000	0.0029380000
19	C	-11.7667660000	-0.8282910000	1.0644420000
20	C	-15.2845190000	-5.5473680000	0.3222170000
21	C	-16.7575390000	-5.6455160000	0.7347490000
22	C	-16.9926210000	-5.0551990000	2.1267620000
23	C	-17.4678350000	-5.1755100000	-1.4970900000
24	C	-17.9807510000	-3.9706680000	-2.2701040000
25	C	-16.3378120000	-5.5556760000	4.4121940000
26	C	-16.9861650000	-4.2304370000	4.8021630000
27	C	-17.0005770000	-3.9797360000	6.3210330000
28	C	-17.6748620000	-2.6467350000	6.6872880000
29	C	-17.6916670000	-2.4197130000	8.2079480000
30	C	-18.3657070000	-1.0924600000	8.5918570000

1	C	-18.3832570000	-0.8830330000	10.1151090000
2	C	-19.0539620000	0.4422210000	10.5115610000
3	C	-19.0715520000	0.6391720000	12.0364310000
4	C	-17.8276100000	-4.1138450000	-3.7924760000
5	C	-18.3740400000	-2.8933830000	-4.5513990000
6	C	-18.2407650000	-3.0669870000	-6.0733350000
7	C	-18.8108320000	-1.8686610000	-6.8495330000
8	C	-18.6954220000	-2.0688610000	-8.3697350000
9	C	-19.2832940000	-0.8870980000	-9.1577700000
10	C	-19.1804950000	-1.1054190000	-10.6763460000
11	C	-11.4512410000	1.5936190000	1.5964420000
12	C	16.4929490000	-6.6681210000	3.9402480000
13	C	17.4505500000	-5.7900470000	3.1253030000
14	C	16.8626160000	-5.4033710000	1.7587060000
15	C	17.8457430000	-4.5460440000	0.9469660000
16	C	17.2802970000	-4.1281090000	-0.4193300000
17	C	18.3226460000	-3.3610780000	-1.2488270000
18	C	17.7496360000	-2.8541550000	-2.5813250000
19	C	18.7730610000	-2.0086840000	-3.3537340000
20	C	18.2198990000	-1.5836230000	-4.6901120000
21	C	17.5674780000	-0.4429120000	-4.9658950000
22	C	17.2293050000	0.6752210000	-4.0092790000
23	C	15.7053550000	0.8416420000	-3.8930190000
24	C	15.3006430000	2.0766400000	-3.0699610000
25	C	13.7730630000	2.2525580000	-3.0375650000
26	C	13.3327350000	3.5485830000	-2.3363770000
27	C	11.8084940000	3.7427760000	-2.4207070000
28	C	21.6260970000	-8.7489570000	5.7095000000
29	C	21.1598510000	-7.2911160000	5.8160850000
30	C	21.3967000000	-6.5186950000	4.5087610000

1	C	20.9296450000	-5.0580950000	4.6127260000
2	C	21.1237410000	-4.2979100000	3.2907730000
3	C	20.5678560000	-2.8670150000	3.3644720000
4	C	20.7411010000	-2.1112580000	2.0370210000
5	C	20.1039640000	-0.7142460000	2.0884970000
6	C	20.1930610000	-0.0296470000	0.7471890000
7	C	19.1693770000	0.2662370000	-0.0702460000
8	C	17.7089000000	-0.0235670000	0.1750080000
9	C	17.0012430000	1.2175360000	0.7332190000
10	C	15.5008680000	0.9714740000	0.9584320000
11	C	14.8013670000	2.2404330000	1.4679750000
12	C	13.2924630000	2.0355620000	1.6763320000
13	C	12.6100230000	3.3471800000	2.0933980000
14	C	11.0976220000	3.1844730000	2.3110480000
15	C	10.4245620000	4.5340070000	2.5025490000
16	C	11.3364790000	5.0482180000	-1.7541180000
17	C	9.8232220000	5.2438860000	-1.8099220000
18	C	9.8394780000	6.4769490000	1.2718720000
19	C	9.9966640000	7.0453560000	-0.1406380000
20	C	9.4104380000	8.4589320000	-0.2169510000
21	C	8.9275660000	13.8350320000	0.0824540000
22	C	9.8039050000	12.9447730000	-0.8247620000
23	C	-0.1167480000	4.7479210000	0.0813720000
24	C	-0.2622840000	6.1784120000	0.6084290000
25	C	-0.0791450000	7.1961270000	-0.5307520000
26	C	-0.2609380000	8.6802290000	-0.1177130000
27	C	0.1512500000	1.6289360000	-1.3673630000
28	C	0.9500550000	1.8731020000	-0.0859320000
29	C	0.0711830000	2.3832050000	1.0247960000
30	C	-4.5820330000	7.5082490000	0.1135270000

1	C	-4.1229760000	6.9807890000	-1.2428290000
2	C	-3.9721300000	5.4569020000	-1.2164770000
3	C	-3.4668070000	4.9201700000	-2.5618110000
4	C	-3.4148310000	3.3891220000	-2.5439550000
5	C	-2.9106610000	2.8182290000	-3.8758010000
6	C	-2.9759990000	1.2877400000	-3.8526200000
7	C	-3.8951870000	5.0885470000	4.0207980000
8	C	-3.9441940000	4.5203820000	2.6064180000
9	C	-3.8871420000	2.9891750000	2.6229070000
10	C	-3.9082180000	2.4228770000	1.2003460000
11	C	-3.9056890000	0.8893920000	1.2147230000
12	C	-4.0595360000	0.3322880000	-0.2022790000
13	C	-3.9704630000	-1.1998440000	-0.2259810000
14	C	10.4354600000	-6.9394360000	-1.0718620000
15	C	9.1648890000	-6.5336580000	-1.8129930000
16	C	8.7125820000	-5.1275060000	-1.4039940000
17	C	7.5218180000	-4.6625910000	-2.2488750000
18	C	7.0528340000	-3.2679620000	-1.8162340000
19	C	6.0228080000	-2.7029790000	-2.8004060000
20	C	5.5665260000	-1.3004800000	-2.3790810000
21	C	-4.7114130000	1.3030400000	6.0896600000
22	C	-4.9009040000	0.0763770000	5.2012900000
23	C	-3.5512340000	-0.5153260000	4.7810790000
24	C	-3.7399260000	-1.7764020000	3.9286150000
25	C	-2.3870390000	-2.3381280000	3.4789520000
26	C	-2.5627830000	-3.5799560000	2.5965020000
27	C	-1.2032970000	-4.2010010000	2.2598680000
28	C	14.5439590000	-12.8396200000	2.5991190000
29	C	13.7887870000	-11.5902060000	3.0430110000
30	C	12.4675510000	-11.4414220000	2.2801510000

1	C	11.7129310000	-10.1840330000	2.7273530000
2	C	10.3925160000	-10.0336740000	1.9630710000
3	C	9.6420460000	-8.7718030000	2.4037530000
4	C	8.3241900000	-8.6198030000	1.6347530000
5	C	4.7477240000	-0.6359570000	-3.4907780000
6	C	-4.3345460000	-1.7318920000	-1.6155880000
7	C	-2.4717680000	0.6615180000	-5.1585870000
8	C	-1.3405970000	-5.3342620000	1.2360410000
9	C	-4.0760790000	-3.2389570000	-1.7568820000
10	C	-4.4869660000	-3.7136170000	-3.1652000000
11	C	-3.8729970000	-5.0557050000	-3.6284740000
12	C	-2.7507800000	-0.8468760000	-5.1597520000
13	C	-2.2182560000	-1.5356540000	-6.4225570000
14	C	-2.7298130000	-2.9937310000	-6.5642220000
15	C	-0.0255340000	-6.1130770000	1.1173520000
16	C	-0.0518710000	-7.0940730000	-0.0638090000
17	C	1.2718570000	-7.8891390000	-0.1832170000
18	C	7.6269820000	-7.3012170000	1.9895870000
19	C	6.2946560000	-7.1654180000	1.2424840000
20	C	5.5909680000	-5.8434510000	1.5937330000
21	C	4.1183870000	-5.8227690000	1.1353000000
22	C	-2.3269760000	-5.0710970000	-3.6922070000
23	C	-1.8353200000	-4.0477050000	-5.8732150000
24	C	3.9818070000	-5.6144720000	-0.3813840000
25	C	2.2878740000	-7.2420010000	-1.1461760000
26	C	4.2636860000	0.7565120000	-3.0699740000
27	C	3.5726710000	1.4788750000	-4.2395730000
28	C	2.6169020000	2.5994270000	-3.7661060000
29	C	-0.3860630000	-3.5888630000	-3.9717210000
30	C	1.1723770000	2.0956770000	-3.5886250000

1	C	2.1442390000	-4.8856920000	-1.8527650000
2	C	-0.2730740000	-3.0823160000	-2.5190060000
3	C	1.8119630000	-3.4965420000	-1.2706270000
4	C	0.7856760000	-0.2234360000	-2.8661440000
5	C	1.1249500000	-1.2293710000	-1.7507930000
6	H	-12.1735470000	7.6332000000	0.6607720000
7	H	-10.4279380000	7.5816180000	0.6037220000
8	H	-11.2356330000	8.3618460000	1.9423560000
9	H	-12.3015110000	5.2635050000	0.5898950000
10	H	-10.4501300000	5.2155600000	0.5206870000
11	H	-12.2788530000	3.9273370000	2.4663450000
12	H	-10.4257630000	3.9104030000	2.4446570000
13	H	-15.1280500000	-3.1301820000	-2.1668110000
14	H	-22.1105000000	5.0036390000	15.9564000000
15	H	-20.5600300000	5.8051770000	15.8748730000
16	H	-21.0908950000	5.0799710000	17.3733140000
17	H	-21.0583160000	2.8869900000	16.2925570000
18	H	-19.4137940000	3.7371490000	16.2060850000
19	H	-21.4437280000	3.4059390000	14.0722470000
20	H	-19.7998660000	4.2573560000	13.9856120000
21	H	-20.3759600000	1.3743950000	14.3587430000
22	H	-18.7323740000	2.2261780000	14.2704760000
23	H	-21.6102980000	1.5719370000	-16.7017720000
24	H	-20.0532710000	2.3643990000	-16.7289490000
25	H	-21.1695850000	2.6762520000	-15.4214090000
26	H	-20.8055860000	-0.0852500000	-15.5132420000
27	H	-19.1541100000	0.7552830000	-15.5420670000
28	H	-21.3326560000	1.0191800000	-13.5501030000
29	H	-19.6798890000	1.8571870000	-13.5784620000
30	H	-20.2923280000	-1.0177040000	-13.2008210000

1	H	-18.6408690000	-0.1769680000	-13.2242040000
2	H	-20.8233120000	0.0851470000	-11.2361040000
3	H	-19.1674710000	0.9172620000	-11.2579120000
4	H	-20.7625430000	1.8882500000	12.1376670000
5	H	-19.1206730000	2.7432930000	12.0489750000
6	H	-12.1073660000	-1.4093410000	-0.9274120000
7	H	-13.6166100000	-1.1252110000	0.1099660000
8	H	-10.7883500000	-1.2570100000	1.0027680000
9	H	-12.3116680000	-0.8689450000	1.9844030000
10	H	-10.8131530000	0.6091970000	0.1406510000
11	H	-15.1521610000	-6.1946220000	-0.5194740000
12	H	-14.7018230000	-5.6949120000	1.2074270000
13	H	-16.9876490000	-6.6904570000	0.7279270000
14	H	-16.4855140000	-4.1133860000	2.1538150000
15	H	-18.0459620000	-5.1010960000	2.3091500000
16	H	-19.0334480000	-3.9279360000	-2.0832770000
17	H	-17.3535090000	-3.1512110000	-1.9873360000
18	H	-18.0119110000	-4.3074460000	4.5075190000
19	H	-16.3654410000	-3.4656460000	4.3841960000
20	H	-17.6122300000	-4.7490280000	6.7440840000
21	H	-15.9766140000	-3.8868300000	6.6172910000
22	H	-18.6975500000	-2.7340470000	6.3849870000
23	H	-17.0590840000	-1.8749810000	6.2748410000
24	H	-18.3045560000	-3.1948850000	8.6182810000
25	H	-16.6682230000	-2.3318410000	8.5075180000
26	H	-19.3883700000	-1.1762670000	8.2884790000
27	H	-17.7501070000	-0.3155100000	8.1890110000
28	H	-18.9990220000	-1.6607800000	10.5161610000
29	H	-17.3599410000	-0.8015580000	10.4169130000
30	H	-20.0768050000	0.3635430000	10.2074200000

1	H	-18.4364670000	1.2211880000	10.1155690000
2	H	-19.6896240000	-0.1399860000	12.4311460000
3	H	-18.0482450000	0.7156940000	12.3395610000
4	H	-18.4427390000	-4.9417370000	-4.0772740000
5	H	-16.7753750000	-4.1379280000	-3.9851450000
6	H	-19.4230140000	-2.8551130000	-4.3438190000
7	H	-17.7458380000	-2.0686560000	-4.2866580000
8	H	-18.8509230000	-3.9070530000	-6.3319770000
9	H	-17.1911520000	-3.0841260000	-6.2805010000
10	H	-19.8574680000	-1.8385800000	-6.6291970000
11	H	-18.1891560000	-1.0317210000	-6.6088010000
12	H	-19.3035000000	-2.9169920000	-8.6059860000
13	H	-17.6484690000	-2.0831340000	-8.5901540000
14	H	-20.3281160000	-0.8636580000	-8.9282090000
15	H	-18.6672620000	-0.0413240000	-8.9340030000
16	H	-19.7879370000	-1.9581060000	-10.8973440000
17	H	-18.1355470000	-1.1189210000	-10.9061330000
18	H	15.5793200000	-6.1375480000	4.1095840000
19	H	16.2875980000	-7.5685780000	3.3999630000
20	H	16.9438360000	-6.9108450000	4.8797620000
21	H	17.5577500000	-4.8772140000	3.6731570000
22	H	18.3089920000	-6.3950510000	2.9204910000
23	H	16.0155680000	-4.7814060000	1.9601230000
24	H	16.7386720000	-6.3163910000	1.2147090000
25	H	17.9783020000	-3.6410830000	1.5022810000
26	H	18.6864330000	-5.1748000000	0.7400710000
27	H	16.4876450000	-3.4378640000	-0.2189160000
28	H	17.0789390000	-5.0340570000	-0.9518830000
29	H	18.5723640000	-2.4892450000	-0.6809820000
30	H	19.0826200000	-4.0704900000	-1.5019590000

1	H	16.9440100000	-2.1959950000	-2.3309440000
2	H	17.5651660000	-3.7224480000	-3.1787680000
3	H	18.9170000000	-1.1131250000	-2.7861460000
4	H	19.6037340000	-2.6509660000	-3.5595430000
5	H	18.3621830000	-2.2625400000	-5.5048070000
6	H	17.2510400000	-0.3112280000	-5.9795160000
7	H	17.6017990000	1.5764570000	-4.4496450000
8	H	17.5769050000	0.3674110000	-3.0452630000
9	H	15.3560030000	-0.0075290000	-3.3436900000
10	H	15.3503860000	1.0189770000	-4.8867240000
11	H	15.6030280000	1.8800450000	-2.0625810000
12	H	15.6911650000	2.9265810000	-3.5895690000
13	H	13.3959400000	1.4499680000	-2.4387940000
14	H	13.4651850000	2.3493860000	-4.0577300000
15	H	13.5637940000	3.4236390000	-1.2991210000
16	H	13.7702470000	4.3538090000	-2.8887310000
17	H	11.3764750000	2.9464420000	-1.8514000000
18	H	11.5796260000	3.8419140000	-3.4612320000
19	H	20.7959000000	-9.4022620000	5.8793940000
20	H	22.3828790000	-8.9358810000	6.4424660000
21	H	22.0246690000	-8.9254120000	4.7323080000
22	H	20.0996530000	-7.3248580000	5.9565920000
23	H	21.7828990000	-6.8301860000	6.5538210000
24	H	20.7741450000	-6.9804140000	3.7711020000
25	H	22.4569650000	-6.4848430000	4.3687860000
26	H	19.8743490000	-5.0927690000	4.7860680000
27	H	21.5738060000	-4.5877660000	5.3259910000
28	H	20.5334550000	-4.8112280000	2.5607270000
29	H	22.1804740000	-4.2018850000	3.1529520000
30	H	19.5133500000	-2.9635120000	3.5181120000

1	H	21.1681460000	-2.3497660000	4.0835030000
2	H	20.1926630000	-2.6641970000	1.3032810000
3	H	21.7928350000	-1.9569370000	1.9147830000
4	H	19.0625930000	-0.8631560000	2.2841270000
5	H	20.6978200000	-0.1350710000	2.7643600000
6	H	21.1708690000	0.2483520000	0.4132590000
7	H	19.4142640000	0.7573870000	-0.9887790000
8	H	17.6704460000	-0.7677270000	0.9428930000
9	H	17.2719520000	-0.2191710000	-0.7819230000
10	H	17.4241050000	1.3935880000	1.7002210000
11	H	17.0707360000	1.9684440000	-0.0258640000
12	H	15.4274940000	0.2493030000	1.7445520000
13	H	15.0813900000	0.7602280000	-0.0029800000
14	H	15.2112830000	2.4374740000	2.4365020000
15	H	14.8980090000	2.9667850000	0.6882490000
16	H	13.1893030000	1.3642670000	2.5031460000
17	H	12.8875990000	1.7739530000	0.7210600000
18	H	13.0208810000	3.6050890000	3.0471160000
19	H	12.7202750000	4.0153620000	1.2649790000
20	H	10.9797620000	2.6538930000	3.2327270000
21	H	10.7035220000	2.7755970000	1.4041820000
22	H	11.5825950000	4.9542970000	-0.7170520000
23	H	11.7571370000	5.8430360000	-2.3339560000
24	H	8.7892080000	6.3839080000	1.4540130000
25	H	10.4432020000	7.0794340000	1.9179590000
26	H	11.0393540000	7.0671700000	-0.3798510000
27	H	8.4078550000	8.3968040000	0.1516490000
28	H	9.5946560000	8.8155390000	-1.2088150000
29	H	9.4022220000	13.9738900000	2.1060200000
30	H	9.5582550000	12.4598490000	1.5144110000

1	H	7.9165870000	13.7204570000	-0.2487460000
2	H	9.3784680000	14.8052480000	0.0988650000
3	H	9.1444090000	12.3994270000	-1.4670740000
4	H	10.5638520000	13.5700860000	-1.2447200000
5	H	7.9397460000	11.2380890000	-0.5524600000
6	H	-0.8180800000	4.0434030000	2.0034330000
7	H	-0.9074680000	4.5575250000	-0.6726220000
8	H	0.8767260000	4.6470610000	-0.4028440000
9	H	-1.2710840000	6.2889700000	1.0574600000
10	H	0.5041920000	6.3420210000	1.3934540000
11	H	-0.8154940000	6.9518030000	-1.3257560000
12	H	0.9331050000	7.0612690000	-0.9719430000
13	H	-1.5853310000	8.5251470000	1.3188370000
14	H	1.7271920000	11.1024340000	1.8945640000
15	H	0.0868190000	9.2499440000	3.0523520000
16	H	-0.7064720000	8.4788420000	-3.3033190000
17	H	-0.4626070000	11.7975930000	-2.1052520000
18	H	-0.3884620000	2.5537260000	-1.6423740000
19	H	-0.6514830000	0.8882610000	-1.1535380000
20	H	1.7832110000	2.5812140000	-0.2752470000
21	H	1.4157380000	0.9157780000	0.2328930000
22	H	-5.5724480000	7.0806460000	0.3783910000
23	H	-3.8470360000	7.2402640000	0.9018120000
24	H	-4.6706080000	8.6142030000	0.0719300000
25	H	-4.8701310000	7.2695800000	-2.0136480000
26	H	-3.1528260000	7.4529980000	-1.5035520000
27	H	-4.9580530000	4.9988960000	-0.9801150000
28	H	-3.2544160000	5.1721680000	-0.4184250000
29	H	-4.1497410000	5.2540480000	-3.3736820000
30	H	-2.4501620000	5.3242340000	-2.7631520000

1	H	-4.4342210000	2.9944840000	-2.3359110000
2	H	-2.7399010000	3.0560060000	-1.7283710000
3	H	-3.5443670000	3.1994570000	-4.7063910000
4	H	-1.8630300000	3.1459370000	-4.0483960000
5	H	-4.0310810000	0.9796280000	-3.6789450000
6	H	-2.3575710000	0.9072620000	-3.0136940000
7	H	-4.7587020000	4.7201370000	4.6147290000
8	H	-2.9502160000	4.7902780000	4.5230660000
9	H	-3.9397360000	6.1972160000	3.9768390000
10	H	-4.8831870000	4.8560420000	2.1145400000
11	H	-3.0854720000	4.9230340000	2.0307790000
12	H	-4.7646060000	2.6009780000	3.1843310000
13	H	-2.9581410000	2.6567200000	3.1365120000
14	H	-4.8187400000	2.7868870000	0.6747970000
15	H	-3.0131480000	2.7848440000	0.6503400000
16	H	-4.7521720000	0.5266700000	1.8364760000
17	H	-2.9522700000	0.5262490000	1.6564170000
18	H	-5.0408780000	0.6569950000	-0.6140110000
19	H	-3.2544150000	0.7474900000	-0.8430390000
20	H	-4.6704360000	-1.6329860000	0.5211640000
21	H	-2.9316660000	-1.5025800000	0.0319310000
22	H	10.2898860000	-6.8507010000	0.0239250000
23	H	11.2818630000	-6.2882160000	-1.3772010000
24	H	10.6899440000	-7.9926070000	-1.3148380000
25	H	8.3593140000	-7.2643870000	-1.5851300000
26	H	9.3663760000	-6.5647450000	-2.9060130000
27	H	8.4257690000	-5.1292660000	-0.3303810000
28	H	9.5563710000	-4.4156160000	-1.5418580000
29	H	6.6813670000	-5.3823050000	-2.1414820000
30	H	7.8289220000	-4.6370290000	-3.3178480000

1	H	6.6064380000	-3.3267370000	-0.7994720000
2	H	7.9272940000	-2.5812120000	-1.7772730000
3	H	5.1401470000	-3.3780040000	-2.8487870000
4	H	6.4834350000	-2.6531760000	-3.8119540000
5	H	4.9527910000	-1.3776300000	-1.4544110000
6	H	6.4553560000	-0.6677240000	-2.1617850000
7	H	-5.7023780000	1.7325890000	6.3470300000
8	H	-4.1915590000	1.0199940000	7.0296070000
9	H	-4.1144830000	2.0774190000	5.5633260000
10	H	-5.4788660000	0.3697110000	4.2984200000
11	H	-5.4871840000	-0.6844980000	5.7611580000
12	H	-2.9840530000	0.2443540000	4.1999490000
13	H	-2.9631980000	-0.7732750000	5.6894630000
14	H	-4.3546140000	-1.5354250000	3.0357200000
15	H	-4.2774820000	-2.5458050000	4.5254270000
16	H	-1.8355070000	-1.5601900000	2.9056190000
17	H	-1.7880940000	-2.6048080000	4.3777820000
18	H	-3.0873850000	-3.2973170000	1.6595820000
19	H	-3.1830330000	-4.3331890000	3.1304950000
20	H	-0.5275060000	-3.4216430000	1.8420800000
21	H	-0.7484300000	-4.5937280000	3.1963440000
22	H	13.9349530000	-13.7481100000	2.7936250000
23	H	14.7818230000	-12.7821260000	1.5155030000
24	H	15.4944530000	-12.9209440000	3.1673800000
25	H	13.5859910000	-11.6613380000	4.1339690000
26	H	14.4292740000	-10.6996440000	2.8611490000
27	H	11.8332820000	-12.3365410000	2.4647930000
28	H	12.6780330000	-11.3758720000	1.1896700000
29	H	11.5015270000	-10.2496180000	3.8175660000
30	H	12.3481830000	-9.2894210000	2.5433420000

1	H	9.7542930000	-10.9254630000	2.1499100000
2	H	10.6049300000	-9.9724010000	0.8726230000
3	H	9.4248580000	-8.8315120000	3.4930800000
4	H	10.2844240000	-7.8823850000	2.2240140000
5	H	7.6554950000	-9.4722240000	1.8869990000
6	H	8.5246640000	-8.6460740000	0.5419370000
7	H	3.8681720000	-1.2736580000	-3.7304060000
8	H	5.3771580000	-0.5436520000	-4.4035170000
9	H	-5.4075040000	-1.5198720000	-1.8189570000
10	H	-3.7243170000	-1.1948530000	-2.3703030000
11	H	-2.9931680000	1.1301730000	-6.0219540000
12	H	-1.3807060000	0.8430670000	-5.2617040000
13	H	-1.6162040000	-4.8980150000	0.2508360000
14	H	-2.1469480000	-6.0338670000	1.5485060000
15	H	-4.6618640000	-3.7941520000	-0.9925120000
16	H	-3.0020850000	-3.4299040000	-1.5723530000
17	H	-5.5950230000	-3.8002180000	-3.1989190000
18	H	-4.2169320000	-2.9412980000	-3.9062500000
19	H	-4.0000750000	-6.0005010000	-1.9233320000
20	H	-4.2410850000	-5.2293850000	-4.6626690000
21	H	-3.8517140000	-0.9885020000	-5.1023360000
22	H	-2.2812430000	-1.3071320000	-4.2682880000
23	H	-1.9534040000	-3.2363990000	-8.3388370000
24	H	-2.5703840000	-0.9531370000	-7.3028180000
25	H	-1.1070360000	-1.5080140000	-6.4329630000
26	H	-3.7501550000	-3.0718910000	-6.1367100000
27	H	0.8156110000	-5.4042120000	1.0016760000
28	H	0.1405000000	-6.6788450000	2.0606410000
29	H	0.6375550000	-9.7019030000	0.1152140000
30	H	-0.2717020000	-6.5559970000	-1.0106260000

1	H	-0.8928950000	-7.8023220000	0.1058960000
2	H	1.7526520000	-7.9723040000	0.8186510000
3	H	7.4387420000	-7.2728420000	3.0855830000
4	H	8.2885460000	-6.4481650000	1.7251310000
5	H	5.6430590000	-8.0228240000	1.5205770000
6	H	6.4825320000	-7.2073470000	0.1486850000
7	H	3.2121930000	-5.1068680000	2.6985000000
8	H	5.6101460000	-5.7152820000	2.6986600000
9	H	6.1456260000	-4.9887300000	1.1483740000
10	H	3.6484550000	-6.7947300000	1.4067740000
11	H	-1.9820760000	-6.0109280000	-4.1797540000
12	H	-1.9577310000	-5.1575760000	-2.6568600000
13	H	-2.2376210000	-5.0460380000	-6.1586710000
14	H	-0.8240760000	-4.0151590000	-6.3412540000
15	H	4.3290190000	-4.5847430000	-0.6112850000
16	H	4.6668710000	-6.2807200000	-0.9472040000
17	H	1.9114470000	-7.3466060000	-2.1891690000
18	H	3.2050280000	-7.8718000000	-1.1164200000
19	H	3.5795520000	0.6537660000	-2.2053200000
20	H	5.1280370000	1.3706140000	-2.7337420000
21	H	3.4309990000	4.1638120000	-4.5803410000
22	H	3.0251000000	0.7604560000	-4.8873670000
23	H	4.3718510000	1.9161080000	-4.8782890000
24	H	2.9628270000	3.0148390000	-2.7891770000
25	H	0.2485820000	-4.4854970000	-4.1316820000
26	H	0.0310980000	-2.7794670000	-4.6120970000
27	H	0.5314950000	2.9943530000	-3.4657010000
28	H	0.8225270000	1.6276950000	-4.5364410000
29	H	1.2289690000	-5.2750520000	-2.3331150000
30	H	2.9237450000	-4.7978980000	-2.6424280000

H	-1.0231270000	-2.2740310000	-2.3800910000
H	-0.5688020000	-3.8625530000	-1.7921110000
H	1.1983320000	-3.5984030000	-0.3495300000
H	2.7728430000	-3.0315900000	-0.9643020000
H	-0.2743120000	-0.3119550000	-3.1790210000
H	1.4098900000	-0.4884730000	-3.7457950000
H	0.4214150000	-1.1279460000	-0.8943980000
H	2.1358230000	-0.9629340000	-1.3743750000

6.3. Previous piperazine amine core based bone-targeting ionizable lipids

Previous-P1-C12^{REF}

P	-6.1400240000	-11.7744050000	0.3880280000
P	11.8813380000	2.3276190000	-1.9610430000
P	2.1294290000	-7.0635610000	-0.6264210000
P	1.9340020000	-9.8023570000	0.5751170000
O	-12.0562720000	-10.4448020000	-1.8376330000
O	-11.3774070000	-9.5220530000	0.1324200000
O	-11.6446050000	-9.3462930000	4.1945190000
O	-10.2584570000	-10.1484530000	2.6201960000
O	-6.0093490000	-13.2625870000	1.2736300000
O	-7.4291370000	-10.8624840000	0.5102120000
O	-4.6646020000	-11.2186790000	0.8052000000
O	-5.8409830000	-12.3799840000	-0.9372120000
O	9.6993420000	-7.5141830000	1.7308910000
O	9.5855060000	-2.5246790000	1.9751920000
O	12.6211630000	1.3555460000	-3.1978640000
O	11.1682980000	3.4150270000	-2.6843340000
O	10.9743360000	1.4860050000	-0.9365960000
O	13.2913090000	2.8611940000	-1.3286520000
O	15.8382350000	3.6656650000	-1.2718960000

1	O	15.3057880000	4.6139610000	-3.2787040000
2	O	14.3588630000	4.9076020000	1.7451010000
3	O	13.6315850000	5.5488950000	3.7784090000
4	O	9.7543320000	-4.1567120000	0.4283740000
5	O	0.0513830000	-8.6522650000	-0.8996310000
6	O	2.5213940000	-5.9220220000	0.2366190000
7	O	3.3799490000	-7.6270870000	-1.4758240000
8	O	1.3572520000	-6.3576090000	-1.8765590000
9	O	2.0935660000	-10.0329620000	2.0329860000
10	O	0.8770590000	-10.9586210000	0.0990970000
11	O	3.2560480000	-10.1426250000	-0.2557070000
12	O	-3.2992720000	-3.0123890000	1.2996210000
13	O	-7.5656370000	10.1304640000	-1.3817540000
14	O	1.5315310000	10.9767380000	-1.2340660000
15	O	-4.6524460000	14.3774200000	1.4247910000
16	O	2.4089130000	-2.7068580000	4.5635420000
17	N	-4.1149150000	-13.3614690000	-0.2280550000
18	N	9.9817920000	-1.9494990000	-0.1709690000
19	N	-2.5344360000	-5.0312230000	1.8089370000
20	N	-4.1462390000	11.2012220000	-0.4073080000
21	N	-1.3446010000	8.6466050000	-1.0657760000
22	N	-0.5603780000	4.9298520000	-0.9152180000
23	N	0.2175190000	2.2624280000	-0.0453320000
24	N	0.4574020000	-1.0074220000	1.9031360000
25	C	-21.3918250000	-1.0535430000	-3.4481010000
26	C	-20.1587430000	-0.2248170000	-3.0674800000
27	C	-19.7144380000	-0.4775050000	-1.6178200000
28	C	-18.4946200000	0.3798430000	-1.2470520000
29	C	-18.0146740000	0.1376010000	0.1924380000
30	C	-16.8611500000	1.0819220000	0.5673200000

1	C	-16.3049120000	0.7951060000	1.9706010000
2	C	-15.0922210000	1.6810790000	2.2919590000
3	C	-14.6074030000	1.4389570000	3.6986930000
4	C	-13.6568270000	0.5729860000	4.0852550000
5	C	-12.8568800000	-0.3623350000	3.2106390000
6	C	-13.1334460000	-1.8254760000	3.5932210000
7	C	-12.2176930000	-2.8184500000	2.8571250000
8	C	-12.4716520000	-4.2633200000	3.3190280000
9	C	-11.4865870000	-5.2719440000	2.7045710000
10	C	-11.7020160000	-6.6853860000	3.2737000000
11	C	-22.2755260000	3.7188130000	-6.6501360000
12	C	-21.0407980000	2.8099850000	-6.7095090000
13	C	-20.0177130000	3.1644900000	-5.6188900000
14	C	-18.7801360000	2.2548340000	-5.6757470000
15	C	-17.7776950000	2.5788140000	-4.5562540000
16	C	-16.5870890000	1.6070410000	-4.5529080000
17	C	-15.5935450000	1.9151740000	-3.4209930000
18	C	-14.4532040000	0.8870860000	-3.3671910000
19	C	-13.5474520000	1.1455090000	-2.1888230000
20	C	-13.4030420000	0.3669120000	-1.1041070000
21	C	-14.1166670000	-0.9337780000	-0.8282920000
22	C	-13.2312870000	-2.1249610000	-1.2154630000
23	C	-13.9108530000	-3.4695350000	-0.9109950000
24	C	-12.9884170000	-4.6478370000	-1.2572760000
25	C	-13.6283760000	-6.0080840000	-0.9373420000
26	C	-12.6448260000	-7.1572820000	-1.2056430000
27	C	-13.2474420000	-8.5366040000	-0.8965750000
28	C	-12.1861790000	-9.6232370000	-0.9588390000
29	C	-10.7106170000	-7.7154730000	2.7015410000
30	C	-10.9266350000	-9.1277920000	3.2400930000

1	C	-10.2917030000	-10.4002630000	0.2407860000
2	C	-9.4650420000	-10.0231210000	1.4726040000
3	C	-8.2729470000	-10.9730230000	1.6284080000
4	C	-3.3675270000	-13.1798170000	1.0878720000
5	C	-3.8142410000	-11.8439850000	1.7199890000
6	C	9.7700040000	-8.8110720000	1.1778360000
7	C	9.7929660000	-6.5117250000	0.7482890000
8	C	9.7006030000	-5.1495090000	1.4199310000
9	C	18.3989400000	-4.1591170000	16.4635060000
10	C	17.7322550000	-2.9167170000	15.8582200000
11	C	17.8210530000	-2.9100110000	14.3237730000
12	C	17.1528210000	-1.6667110000	13.7146960000
13	C	20.3690330000	-4.9361290000	-16.0190240000
14	C	19.6758760000	-3.7090010000	-15.4120660000
15	C	19.6642750000	-3.7595560000	-13.8758900000
16	C	18.9721810000	-2.5299300000	-13.2655780000
17	C	18.9632290000	-2.5760390000	-11.7287520000
18	C	17.2417310000	-1.6575020000	12.1797470000
19	C	10.9571020000	0.0859060000	-1.0444040000
20	C	10.0424010000	-0.5002500000	0.0298070000
21	C	13.4773580000	4.2198560000	-1.0270060000
22	C	14.9134820000	4.4173350000	-0.5288560000
23	C	15.0628110000	4.0028790000	0.9365330000
24	C	15.7958600000	3.7141800000	-2.6344470000
25	C	16.3954170000	2.4457860000	-3.2209500000
26	C	14.2310600000	4.7445060000	3.0953830000
27	C	14.8846340000	3.4977000000	3.6844570000
28	C	14.7956910000	3.4274310000	5.2197680000
29	C	15.4770450000	2.1710510000	5.7880650000
30	C	15.3897260000	2.1245770000	7.3224850000

1	C	16.0694040000	0.8759630000	7.9075650000
2	C	15.9821700000	0.8472810000	9.4425020000
3	C	16.6575600000	-0.3979950000	10.0395380000
4	C	16.5699160000	-0.4141030000	11.5745900000
5	C	16.3491690000	2.4040570000	-4.7564260000
6	C	16.9815370000	1.1229890000	-5.3248030000
7	C	16.9542520000	1.1122080000	-6.8621150000
8	C	17.6108450000	-0.1480860000	-7.4486200000
9	C	17.6005370000	-0.1316410000	-8.9861630000
10	C	18.2752980000	-1.3762590000	-9.5851460000
11	C	18.2769590000	-1.3411940000	-11.1223750000
12	C	9.7539820000	-2.8526760000	0.8242470000
13	C	-1.4963040000	-5.9934270000	2.1931540000
14	C	-0.8113360000	-6.5780790000	0.9481010000
15	C	0.1609430000	-7.7157050000	1.3105760000
16	C	0.9611230000	-8.2773200000	0.1284520000
17	C	-0.8705560000	-1.6012200000	1.7405790000
18	C	-0.9965540000	-3.1105210000	2.0189250000
19	C	-2.3698070000	-3.6804130000	1.6743920000
20	C	5.1481420000	-7.1776710000	-7.8582160000
21	C	5.6603700000	-6.7815340000	-6.4670060000
22	C	4.7002820000	-5.8123500000	-5.7591120000
23	C	5.2059750000	-5.4192140000	-4.3615830000
24	C	4.2364220000	-4.4622640000	-3.6494800000
25	C	4.7188450000	-4.1035280000	-2.2345700000
26	C	3.6970600000	-3.2354100000	-1.4829270000
27	C	-4.5789860000	8.4161590000	-13.3055360000
28	C	-3.3261280000	8.3235700000	-12.4242640000
29	C	-3.6153180000	8.7422140000	-10.9738780000
30	C	-2.3615690000	8.6560170000	-10.0882570000

1	C	-2.6512060000	9.0824550000	-8.6399480000
2	C	-1.3953370000	9.0122530000	-7.7558590000
3	C	-1.6847580000	9.4591310000	-6.3138060000
4	C	-4.2490930000	9.4779690000	13.4787920000
5	C	-3.5600880000	9.4002360000	12.1098360000
6	C	-4.2853940000	10.2516520000	11.0555690000
7	C	-3.6002590000	10.1735670000	9.6814790000
8	C	-4.3295890000	11.0217820000	8.6267480000
9	C	-3.6522340000	10.9351470000	7.2492700000
10	C	-4.3920540000	11.7708300000	6.1919810000
11	C	-1.4623230000	0.6649910000	5.3056540000
12	C	-2.4302070000	1.8448100000	5.4587900000
13	C	-2.6598880000	2.5747740000	4.1258080000
14	C	-3.6122920000	3.7706860000	4.2871400000
15	C	-3.8821900000	4.4851020000	2.9530500000
16	C	-4.7696860000	5.7274590000	3.1402480000
17	C	-5.0807300000	6.4185450000	1.8034560000
18	C	4.0917730000	-3.0132250000	-0.0121410000
19	C	-5.8439740000	7.7417520000	1.9923380000
20	C	-3.7308750000	11.6633400000	4.8083830000
21	C	-4.4952430000	12.4655770000	3.7424070000
22	C	-6.1280200000	8.4230250000	0.6430920000
23	C	-6.5938950000	9.8805330000	0.7888360000
24	C	-6.6065940000	10.6919350000	-0.5144230000
25	C	-0.4256750000	9.4190830000	-5.4320550000
26	C	-0.7184300000	9.9008190000	-4.0015570000
27	C	0.5418650000	9.8986550000	-3.1191470000
28	C	0.3117720000	10.4425990000	-1.7040540000
29	C	-3.8629820000	12.3123500000	2.3492820000
30	C	-4.6920160000	12.9799230000	1.2473730000

1	C	-0.1455570000	9.3857080000	-0.6883160000
2	C	-5.2592610000	10.7553780000	-1.2470060000
3	C	-4.1851420000	12.6460700000	-0.1614240000
4	C	-2.8434260000	10.7625150000	-0.9135720000
5	C	-2.5975540000	9.2657570000	-0.6287640000
6	C	-1.3176970000	7.2771270000	-0.5400960000
7	C	-0.3359640000	6.3314670000	-1.2659280000
8	C	-0.5106010000	3.1733740000	0.8246850000
9	C	-0.2316430000	4.6435990000	0.4733140000
10	C	0.2023210000	4.0344870000	-1.7722050000
11	C	-0.1028970000	2.5650860000	-1.4332710000
12	C	2.8961890000	-2.5297910000	0.8259100000
13	C	3.2059230000	-2.4664080000	2.3310370000
14	C	1.9681090000	-2.5311070000	3.2341570000
15	C	1.0733530000	-1.2854670000	3.2002200000
16	C	0.4074240000	0.4394220000	1.6730100000
17	C	-0.0604920000	0.8600680000	0.2629390000
18	H	-21.1672100000	-2.0947080000	-3.3460000000
19	H	-22.2058290000	-0.7992350000	-2.8018620000
20	H	-21.6627230000	-0.8448670000	-4.4619880000
21	H	-19.3609970000	-0.5739030000	-3.6892840000
22	H	-20.4626190000	0.8001540000	-3.1121380000
23	H	-19.3908220000	-1.4964970000	-1.5750650000
24	H	-20.5175800000	-0.1446200000	-0.9940820000
25	H	-17.6987380000	0.0539550000	-1.8836590000
26	H	-18.8254380000	1.3968170000	-1.2820210000
27	H	-17.6114820000	-0.8533190000	0.2127940000
28	H	-18.8341370000	0.3949750000	0.8305120000
29	H	-16.0669910000	0.8686050000	-0.1173010000
30	H	-17.2821790000	2.0647290000	0.6088550000

1	H	-15.9443130000	-0.2121050000	1.9507720000
2	H	-17.0728490000	1.0749270000	2.6611610000
3	H	-14.3042860000	1.3648270000	1.6407740000
4	H	-15.4387500000	2.6929180000	2.2603430000
5	H	-15.0695250000	2.0182810000	4.4705270000
6	H	-13.4416170000	0.5422280000	5.1329380000
7	H	-11.8259910000	-0.1790990000	3.4310770000
8	H	-13.2216990000	-0.2337810000	2.2130020000
9	H	-14.1277410000	-2.0359620000	3.2585990000
10	H	-12.8889560000	-1.9111650000	4.6313840000
11	H	-12.4970740000	-2.7782470000	1.8250250000
12	H	-11.2166630000	-2.5796940000	3.1501010000
13	H	-13.4380090000	-4.5279400000	2.9434860000
14	H	-12.2859700000	-4.2735190000	4.3727440000
15	H	-11.7227190000	-5.3273570000	1.6624230000
16	H	-10.5094190000	-4.9671550000	3.0162540000
17	H	-12.6722280000	-6.9929530000	2.9435630000
18	H	-11.4891460000	-6.6189590000	4.3202060000
19	H	-23.1489370000	3.1234710000	-6.4839370000
20	H	-22.3745740000	4.2468270000	-7.5754960000
21	H	-22.1641550000	4.4199470000	-5.8495700000
22	H	-21.3851110000	1.8214620000	-6.4877650000
23	H	-20.5637750000	3.0129610000	-7.6455380000
24	H	-20.4953630000	2.9621840000	-4.6830360000
25	H	-19.6733700000	4.1528830000	-5.8411650000
26	H	-19.1297180000	1.2614300000	-5.4864290000
27	H	-18.2842220000	2.4811000000	-6.5964920000
28	H	-18.2967490000	2.4195940000	-3.6342280000
29	H	-17.3758390000	3.5439240000	-4.7842300000
30	H	-16.9915430000	0.6394650000	-4.3405080000

1	H	-16.0596800000	1.7772910000	-5.4681970000
2	H	-16.1380930000	1.8072500000	-2.5062690000
3	H	-15.1405790000	2.8529610000	-3.6665080000
4	H	-14.9110130000	-0.0644930000	-3.1945420000
5	H	-13.8614110000	1.0447600000	-4.2445850000
6	H	-12.9606350000	2.0396830000	-2.2204310000
7	H	-12.7117070000	0.7030390000	-0.3598120000
8	H	-14.9651940000	-0.9601180000	-1.4796040000
9	H	-14.2538440000	-0.9875530000	0.2315150000
10	H	-13.1181950000	-2.0807380000	-2.2785500000
11	H	-12.3694750000	-2.0752600000	-0.5832370000
12	H	-14.7512260000	-3.5396450000	-1.5695970000
13	H	-14.0573120000	-3.5013180000	0.1484580000
14	H	-12.8592400000	-4.6223280000	-2.3191430000
15	H	-12.1388820000	-4.5598820000	-0.6127190000
16	H	-14.4357670000	-6.1329330000	-1.6283070000
17	H	-13.8196060000	-6.0126910000	0.1154210000
18	H	-12.4552010000	-7.1456460000	-2.2586420000
19	H	-11.8381760000	-7.0245800000	-0.5152760000
20	H	-13.9421520000	-8.7465260000	-1.6828390000
21	H	-13.5835720000	-8.4995390000	0.1185820000
22	H	-10.9132630000	-7.7669350000	1.6521660000
23	H	-9.7430930000	-7.4146170000	3.0454620000
24	H	-10.6966980000	-11.3734280000	0.4247140000
25	H	-9.6699800000	-10.2322620000	-0.6136940000
26	H	-9.1195870000	-9.0178210000	1.3504030000
27	H	-8.6681990000	-11.9672980000	1.6188000000
28	H	-7.7126680000	-10.6281570000	2.4722420000
29	H	-3.4595520000	-13.3861070000	-0.9829670000
30	H	-4.7472610000	-12.5981670000	-0.3603730000

1	H	-3.6928480000	-13.9606280000	1.7431580000
2	H	-2.3295910000	-13.0808920000	0.8474490000
3	H	-4.3976800000	-12.0813720000	2.5849430000
4	H	-2.9428600000	-11.2302370000	1.8143800000
5	H	-5.9833300000	-13.0738180000	2.2145280000
6	H	10.7032310000	-8.9311930000	0.6683670000
7	H	8.9655850000	-8.9479670000	0.4856880000
8	H	9.6947570000	-9.5370620000	1.9602530000
9	H	10.7726460000	-6.5831210000	0.3239910000
10	H	8.9305220000	-6.6086440000	0.1224320000
11	H	10.5784580000	-5.0378310000	2.0214280000
12	H	8.7320160000	-5.0886220000	1.8705220000
13	H	13.5661990000	1.5235760000	-3.2144060000
14	H	19.4324670000	-4.1795870000	16.1872760000
15	H	17.9139390000	-5.0390890000	16.0956430000
16	H	18.3153200000	-4.1250490000	17.5296900000
17	H	18.3025360000	-2.0763780000	16.1951320000
18	H	16.6918940000	-2.9880170000	16.0979400000
19	H	18.8614710000	-2.8381620000	14.0844670000
20	H	17.2515320000	-3.7510450000	13.9873100000
21	H	17.7218720000	-0.8257870000	14.0522270000
22	H	16.1123370000	-1.7392150000	13.9535160000
23	H	21.2074360000	-4.6194320000	-16.6035610000
24	H	19.6781920000	-5.4637860000	-16.6428970000
25	H	20.7048030000	-5.5808360000	-15.2338420000
26	H	20.2747760000	-2.8636290000	-15.6795830000
27	H	18.6527680000	-3.7592010000	-15.7213050000
28	H	20.6875900000	-3.7111370000	-13.5670500000
29	H	19.0641940000	-4.6041930000	-13.6087000000
30	H	19.5706590000	-1.6851380000	-13.5358540000

1	H	17.9482800000	-2.5802740000	-13.5721610000
2	H	19.9872190000	-2.5307560000	-11.4216780000
3	H	18.3601260000	-3.4173040000	-11.4577540000
4	H	18.2818940000	-1.5825640000	11.9402760000
5	H	16.6741880000	-2.4992170000	11.8416520000
6	H	10.5107300000	-0.1450950000	-1.9890160000
7	H	11.9479250000	-0.2525040000	-0.8238400000
8	H	9.0599210000	-0.1163310000	-0.1497420000
9	H	10.5179610000	-0.3329330000	0.9736010000
10	H	9.1969410000	-2.0687000000	-0.7790800000
11	H	13.3890670000	4.7589360000	-1.9470590000
12	H	12.8283470000	4.4495990000	-0.2079200000
13	H	15.1154870000	5.4617030000	-0.6445720000
14	H	14.5804160000	3.0535130000	1.0409280000
15	H	16.0985740000	4.1067900000	1.1841200000
16	H	17.4325820000	2.4622310000	-2.9584270000
17	H	15.7714560000	1.6436130000	-2.8861490000
18	H	15.9266020000	3.5756020000	3.4539510000
19	H	14.3164640000	2.6678700000	3.3191280000
20	H	15.3543290000	4.2620040000	5.5889720000
21	H	13.7558090000	3.3340030000	5.4538950000
22	H	16.5162440000	2.2581740000	5.5485330000
23	H	14.9135920000	1.3351320000	5.4293740000
24	H	15.9503610000	2.9635400000	7.6784760000
25	H	14.3499870000	2.0365520000	7.5593270000
26	H	17.1087480000	0.9594800000	7.6673830000
27	H	15.5055720000	0.0360180000	7.5590160000
28	H	16.5462750000	1.6878120000	9.7891900000
29	H	14.9422250000	0.7658710000	9.6808020000
30	H	17.6972770000	-0.3196520000	9.7992250000

1	H	16.0913960000	-1.2392050000	9.6978880000
2	H	17.1367420000	0.4271660000	11.9149920000
3	H	15.5297520000	-0.4904420000	11.8136120000
4	H	16.9609750000	3.2139390000	-5.0950770000
5	H	15.3133850000	2.3684360000	-5.0224760000
6	H	18.0133680000	1.1461650000	-5.0425140000
7	H	16.3580650000	0.3134400000	-5.0072950000
8	H	17.5588820000	1.9370640000	-7.1766560000
9	H	15.9223180000	1.0680870000	-7.1415150000
10	H	18.6392030000	-0.1153700000	-7.1548360000
11	H	16.9959460000	-0.9723420000	-7.1529850000
12	H	18.2012560000	0.7037710000	-9.2796310000
13	H	16.5722910000	-0.1800670000	-9.2781630000
14	H	19.3010010000	-1.3359340000	-9.2831420000
15	H	17.6674900000	-2.2110730000	-9.3048700000
16	H	18.8758180000	-0.4994960000	-11.4013240000
17	H	17.2514120000	-1.3914140000	-11.4234250000
18	H	-2.7828950000	-5.3225130000	0.8851300000
19	H	-0.7479300000	-5.4393870000	2.7202920000
20	H	-1.9982220000	-6.8066360000	2.6744750000
21	H	-0.2129720000	-5.7915600000	0.5379200000
22	H	-1.5869520000	-7.0225130000	0.3600540000
23	H	0.8842490000	-7.2788440000	1.9669900000
24	H	-0.4534170000	-8.5223550000	1.6523030000
25	H	0.1776800000	-9.5789070000	-1.1163880000
26	H	3.3176500000	-7.3158460000	-2.3818310000
27	H	1.9081650000	-6.4056970000	-2.6612770000
28	H	1.2293580000	-11.4186720000	-0.6663050000
29	H	3.0512250000	-10.7942470000	-0.9302710000
30	H	-1.4815090000	-1.1279520000	2.4806150000

1	H	-1.1030610000	-1.4874940000	0.7023560000
2	H	-0.8892500000	-3.2235280000	3.0775170000
3	H	-0.3056960000	-3.5895440000	1.3569940000
4	H	4.1960760000	-7.6569500000	-7.7646130000
5	H	5.0483620000	-6.3015740000	-8.4643490000
6	H	5.8428210000	-7.8507100000	-8.3157540000
7	H	5.6658700000	-7.6781020000	-5.8830240000
8	H	6.5698560000	-6.2405080000	-6.6252060000
9	H	3.7889740000	-6.3519640000	-5.6066870000
10	H	4.6993250000	-4.9141260000	-6.3405700000
11	H	5.2175510000	-6.3192400000	-3.7830320000
12	H	6.1112800000	-4.8696430000	-4.5142090000
13	H	3.3204910000	-5.0014020000	-3.5257810000
14	H	4.2493210000	-3.5510400000	-4.2101970000
15	H	4.7812280000	-5.0262780000	-1.6964800000
16	H	5.5916730000	-3.4975270000	-2.3604060000
17	H	2.7853620000	-3.7952420000	-1.4657700000
18	H	3.7247040000	-2.2721400000	-1.9479490000
19	H	-5.3381740000	7.7711230000	-12.9150720000
20	H	-4.9365850000	9.4246130000	-13.3122680000
21	H	-4.3357800000	8.1173030000	-14.3037520000
22	H	-3.0595960000	7.2878670000	-12.3899110000
23	H	-2.6336470000	9.0416580000	-12.8112010000
24	H	-4.3052300000	8.0218740000	-10.5865390000
25	H	-3.8858220000	9.7768450000	-11.0094740000
26	H	-2.0941450000	7.6207650000	-10.0477940000
27	H	-1.6693760000	9.3725210000	-10.4786310000
28	H	-3.3347690000	8.3594540000	-8.2463620000
29	H	-2.9298480000	10.1146730000	-8.6821880000
30	H	-1.1250500000	7.9784240000	-7.7007140000

H	-0.7056920000	9.7248370000	-8.1577450000
H	-2.3583050000	8.7346280000	-5.9059720000
H	-1.9759860000	10.4870730000	-6.3723060000
H	-3.5642610000	9.8708620000	14.2009700000
H	-4.5598640000	8.4987240000	13.7777910000
H	-5.1035810000	10.1185240000	13.4121580000
H	-2.5924960000	9.8393130000	12.2358370000
H	-3.6484920000	8.3839420000	11.7869880000
H	-4.1946900000	11.2679510000	11.3777640000
H	-5.2539410000	9.8139240000	10.9322400000
H	-2.6330500000	10.6145500000	9.8037150000
H	-3.6878870000	9.1565770000	9.3606160000
H	-4.2346240000	12.0394430000	8.9433680000
H	-5.2998690000	10.5858310000	8.5109730000
H	-2.6864490000	11.3817750000	7.3618890000
H	-3.7368770000	9.9150490000	6.9376040000
H	-4.2909090000	12.7924760000	6.4934910000
H	-5.3646820000	11.3358560000	6.0935310000
H	-0.6111350000	0.8178480000	5.9357450000
H	-1.9568400000	-0.2409610000	5.5877970000
H	-1.1437390000	0.5949720000	4.2865860000
H	-1.9466570000	2.5469590000	6.1053710000
H	-3.3739930000	1.4239220000	5.7363160000
H	-1.7126980000	2.9820280000	3.8396690000
H	-3.1586060000	1.8784500000	3.4844700000
H	-3.1004940000	4.4779590000	4.9057900000
H	-4.5504150000	3.3641600000	4.6026740000
H	-2.9348420000	4.8425130000	2.6070880000
H	-4.4466100000	3.8005510000	2.3549530000
H	-4.1920500000	6.4247510000	3.7103540000

1	H	-5.7055310000	5.3724810000	3.5185130000
2	H	-5.7442850000	5.7644660000	1.2773620000
3	H	-4.1351360000	6.6786310000	1.3755460000
4	H	4.3623970000	-3.9732220000	0.3752460000
5	H	4.8041170000	-2.2148160000	-0.0089330000
6	H	-6.7963600000	7.4840310000	2.4063930000
7	H	-5.1869890000	8.3950400000	2.5275800000
8	H	-2.7700540000	12.1254120000	4.8990050000
9	H	-3.8052210000	10.6356010000	4.5200720000
10	H	-4.3879190000	13.4963450000	4.0086780000
11	H	-5.4696250000	12.0279800000	3.6792560000
12	H	-6.9511160000	7.8921390000	0.2123110000
13	H	-5.1899570000	8.4633090000	0.1299490000
14	H	-7.6152350000	9.8256540000	1.1030860000
15	H	-5.8662530000	10.3535360000	1.4147000000
16	H	-7.2131800000	10.1037790000	-2.2743130000
17	H	-6.8405040000	11.6986390000	-0.2374180000
18	H	-0.1481940000	8.3888560000	-5.3512120000
19	H	0.2584540000	10.1259690000	-5.8529820000
20	H	-1.3834910000	9.1798660000	-3.5739820000
21	H	-1.0212720000	10.9235020000	-4.0870420000
22	H	0.8099520000	8.8701100000	-2.9961660000
23	H	1.2251620000	10.5776170000	-3.5849980000
24	H	1.7868840000	11.7221440000	-1.7824850000
25	H	-0.4702930000	11.1686720000	-1.7821130000
26	H	-4.4235430000	14.7991900000	0.5933380000
27	H	-2.9347670000	12.8434310000	2.3849050000
28	H	-3.8718340000	11.2644130000	2.1332970000
29	H	-5.6906610000	12.6044290000	1.3286940000
30	H	-0.4094150000	9.9302910000	0.1941280000

1	H	0.6404420000	8.6604770000	-0.6544440000
2	H	-5.0301540000	9.7474840000	-1.5236970000
3	H	-5.3730810000	11.5047920000	-2.0022040000
4	H	-3.1703880000	12.9830990000	-0.2012480000
5	H	-4.9085250000	13.0452310000	-0.8413410000
6	H	-2.1068090000	11.3000530000	-0.3537970000
7	H	-2.8850930000	10.8631430000	-1.9780140000
8	H	-2.5759540000	9.1942020000	0.4386220000
9	H	-3.3578530000	8.7570420000	-1.1837920000
10	H	-0.9527770000	7.3590870000	0.4624090000
11	H	-2.2932460000	6.8775970000	-0.7233440000
12	H	0.6398120000	6.5777490000	-0.9024620000
13	H	-0.5554300000	6.4170090000	-2.3096790000
14	H	-0.1223370000	3.0216160000	1.8101400000
15	H	-1.5495770000	3.0098860000	0.6279600000
16	H	-0.9018300000	5.2281000000	1.0683850000
17	H	0.8253870000	4.7806060000	0.5672140000
18	H	1.2338520000	4.1930520000	-1.5361960000
19	H	-0.1491600000	4.1957630000	-2.7698780000
20	H	-1.1618470000	2.4468240000	-1.5309350000
21	H	0.5588490000	1.9739990000	-2.0312750000
22	H	2.1353300000	-3.2734120000	0.7118030000
23	H	2.7091650000	-1.5216690000	0.5199470000
24	H	3.0070080000	-3.4565850000	4.6058830000
25	H	3.6368050000	-1.5013600000	2.4981400000
26	H	3.7701420000	-3.3491650000	2.5485150000
27	H	1.3730070000	-3.3410090000	2.8669900000
28	H	0.2620060000	-1.4952060000	3.8655210000
29	H	1.7236960000	-0.4581130000	3.3937150000
30	H	-0.3365180000	0.8119820000	2.3458030000

H	1.4191490000	0.7783690000	1.7531790000
H	-1.1263030000	0.7655520000	0.2664990000
H	0.5252010000	0.2862480000	-0.4245170000

6.4. Branched amine core based bone-targeting ionizable lipids ‘Type 1’

Type1-B1-C12

P	-14.2066400000	1.5548820000	-2.1077440000
P	8.2600900000	0.3900900000	7.9395540000
P	-2.5835490000	0.5388530000	8.8222820000
P	-1.4154860000	3.2197210000	8.1162720000
O	-10.9876400000	1.4536830000	8.1333660000
O	-11.7201270000	-0.6491570000	3.6601880000
O	-14.6006690000	3.1813140000	-1.6371540000
O	-13.5865180000	1.6708010000	-3.4553510000
O	-13.3255080000	0.7653490000	-1.0211440000
O	-15.7582300000	1.0501990000	-2.2102380000
O	-18.3850200000	1.1838870000	-2.6785580000
O	-17.7413110000	2.5517580000	-4.3887070000
O	-17.5583660000	-2.3007440000	-2.7635110000
O	-17.2330160000	-4.5249160000	-2.6160770000
O	-11.4063010000	1.3960130000	4.5565550000
O	11.6138900000	-3.1044910000	3.6659930000
O	11.7367960000	-0.8304610000	3.5816450000
O	10.8130280000	2.6600830000	1.6933430000
O	10.1686210000	1.4862110000	3.4961530000
O	6.5989340000	0.6664860000	7.5150310000
O	9.3940090000	0.3788440000	6.8339120000
O	8.2836880000	1.4617690000	9.1686520000
O	8.0008860000	-0.8729410000	8.6811580000
O	4.8388900000	-5.8637390000	-0.3546820000

1	O	-0.7803250000	3.3025830000	-1.5937280000
2	O	-6.9933590000	-1.2174670000	-4.6441680000
3	O	6.3510650000	-0.2133490000	-6.5695140000
4	O	-3.8974550000	2.4641730000	7.4821010000
5	O	-3.5725990000	-0.6058910000	8.4703950000
6	O	-1.0528940000	-0.2609440000	9.0088160000
7	O	-3.0679040000	1.0377200000	10.4136650000
8	O	-1.4032390000	4.4782170000	7.2066150000
9	O	-1.7870990000	3.8600530000	9.6873520000
10	O	0.2464000000	2.7207990000	8.1897750000
11	O	0.6164030000	-4.6364510000	1.4074200000
12	O	10.7883010000	-1.4805270000	-3.1960430000
13	O	-0.9451780000	4.0158630000	1.0990340000
14	O	-5.8149150000	3.6213670000	1.1515210000
15	N	-11.9039610000	1.1611750000	2.3249800000
16	N	6.4563240000	-0.0153950000	9.8320150000
17	N	7.2547040000	-0.2524880000	-2.9231650000
18	N	4.2055710000	0.2757830000	-0.6620150000
19	N	2.9894810000	-3.3371030000	-0.0439550000
20	N	1.0855560000	2.1440820000	0.5132620000
21	N	-1.9503390000	2.6423970000	2.5695620000
22	N	-5.5926280000	2.4697380000	-0.7748400000
23	N	-6.8571430000	0.3933370000	-2.2407550000
24	C	-10.7696970000	2.4527090000	9.1066240000
25	C	-11.1059920000	1.9903830000	6.8383220000
26	C	-11.3297320000	0.8551770000	5.8501350000
27	C	-22.0685780000	-11.5204400000	11.7214570000
28	C	-21.5352180000	-11.5606250000	10.2832940000
29	C	-21.4109260000	-10.1525880000	9.6796060000
30	C	-20.8756770000	-10.1899600000	8.2389760000

1	C	-19.3765420000	18.6457910000	-0.2096470000
2	C	-18.9824330000	17.5052620000	-1.1575810000
3	C	-19.1752680000	16.1266950000	-0.5057420000
4	C	-18.7830650000	14.9823910000	-1.4545310000
5	C	-18.9793000000	13.6020810000	-0.8062440000
6	C	-20.7518320000	-8.7823790000	7.6328130000
7	C	-13.0664920000	1.3817120000	0.2138480000
8	C	-12.2261870000	0.4503210000	1.0859620000
9	C	-16.2022500000	0.3053750000	-3.3146160000
10	C	-17.7062740000	0.0528350000	-3.1605440000
11	C	-17.9874810000	-1.0951930000	-2.1887450000
12	C	-18.1631220000	2.3952440000	-3.2651310000
13	C	-18.4619140000	3.5147480000	-2.2803920000
14	C	-17.5937020000	-3.4906680000	-2.0934170000
15	C	-18.1112960000	-3.4366140000	-0.6588890000
16	C	-18.2252480000	-4.8217770000	0.0032010000
17	C	-18.7655570000	-4.7399940000	1.4409340000
18	C	-18.8848340000	-6.1329060000	2.0810880000
19	C	-19.4270850000	-6.0695790000	3.5181930000
20	C	-19.5494060000	-7.4695750000	4.1423030000
21	C	-20.0896630000	-7.4191660000	5.5806690000
22	C	-20.2136400000	-8.8240300000	6.1932980000
23	C	-18.1976780000	4.9160520000	-2.8531860000
24	C	-18.5289740000	6.0300660000	-1.8465610000
25	C	-18.2880340000	7.4251150000	-2.4465370000
26	C	-18.6438640000	8.5524650000	-1.4637280000
27	C	-18.4238980000	9.9408120000	-2.0870430000
28	C	-18.7982970000	11.0771120000	-1.1217430000
29	C	-18.5930920000	12.4605160000	-1.7607980000
30	C	-11.6723680000	0.5504700000	3.5214260000

1	C	23.1127020000	-4.3438980000	-2.9810450000
2	C	23.4603290000	-3.2893150000	-1.9231410000
3	C	22.7028130000	-1.9707020000	-2.1471960000
4	C	23.0830590000	-0.9189300000	-1.0937920000
5	C	22.3291090000	0.4069560000	-1.2802000000
6	C	22.8053590000	1.4707060000	-0.2779440000
7	C	22.0012230000	2.7755340000	-0.3858900000
8	C	22.4237800000	3.7923420000	0.6848030000
9	C	21.6780160000	5.0915730000	0.5162620000
10	C	20.5274660000	5.4431490000	1.1125500000
11	C	19.7089430000	4.6249270000	2.0822040000
12	C	18.3165010000	4.3337270000	1.4991560000
13	C	17.3761030000	3.6547890000	2.5096670000
14	C	15.9723340000	3.4467130000	1.9166790000
15	C	14.9654350000	2.8914910000	2.9380100000
16	C	13.5505700000	2.8009630000	2.3395990000
17	C	28.6597410000	-5.7045770000	-1.8909730000
18	C	27.5245560000	-5.6565930000	-0.8599060000
19	C	27.2847850000	-4.2302600000	-0.3403770000
20	C	26.1474010000	-4.1794110000	0.6921320000
21	C	25.8763960000	-2.7461930000	1.1774750000
22	C	24.6762930000	-2.6817940000	2.1353400000
23	C	24.3893440000	-1.2465970000	2.6056350000
24	C	23.1354890000	-1.1761450000	3.4905750000
25	C	22.8114950000	0.2510460000	3.8564670000
26	C	21.7467420000	0.9603330000	3.4479480000
27	C	20.6451000000	0.4840640000	2.5332450000
28	C	19.4299000000	0.0255480000	3.3491920000
29	C	18.2709360000	-0.4299300000	2.4482300000
30	C	17.0466960000	-0.8355670000	3.2824290000

1	C	15.8588550000	-1.2696000000	2.4090310000
2	C	14.6239810000	-1.5760600000	3.2699490000
3	C	13.4121640000	-2.0134700000	2.4324690000
4	C	12.1570550000	-2.0920080000	3.2865520000
5	C	12.5102060000	2.2768460000	3.3467050000
6	C	11.1052120000	2.1600260000	2.7604520000
7	C	10.6198680000	-0.6776270000	4.4128250000
8	C	10.4104590000	0.8113020000	4.6995630000
9	C	9.1869110000	1.0161910000	5.5988030000
10	C	6.0621710000	1.4485840000	9.9890540000
11	C	7.2310090000	2.3230800000	9.4864130000
12	C	8.2409680000	11.9981860000	-4.1813880000
13	C	9.1673520000	10.8713180000	-3.7337770000
14	C	8.5089110000	9.5017280000	-3.9340650000
15	C	9.4439460000	8.3713920000	-3.4880390000
16	C	8.7885410000	7.0022760000	-3.7003770000
17	C	9.7270380000	5.8678750000	-3.2715130000
18	C	9.0759980000	4.5026440000	-3.5153040000
19	C	-3.5603760000	7.3034930000	-3.5341730000
20	C	-2.0359900000	7.2782690000	-3.4621990000
21	C	-1.4725180000	6.0207470000	-4.1328950000
22	C	0.0585730000	5.9839440000	-4.0423940000
23	C	0.6090640000	4.7019150000	-4.6761670000
24	C	2.1314780000	4.6097060000	-4.5308300000
25	C	2.6534630000	3.2689580000	-5.0656900000
26	C	-6.0719610000	-3.4969390000	-10.5936820000
27	C	-6.1656800000	-3.3616670000	-9.0767080000
28	C	-4.8370660000	-3.7258090000	-8.4054030000
29	C	-4.9336220000	-3.5845830000	-6.8809670000
30	C	-3.5938980000	-3.9190570000	-6.2180630000

1	C	-3.6712620000	-3.7677250000	-4.6929760000
2	C	-2.3081620000	-4.0552530000	-4.0612020000
3	C	-3.2043790000	-6.0731040000	-11.4723220000
4	C	-1.9234830000	-6.6238790000	-10.8529780000
5	C	-1.8729870000	-6.3385760000	-9.3485810000
6	C	-0.5740590000	-6.8691290000	-8.7303500000
7	C	-0.5107990000	-6.5383500000	-7.2370770000
8	C	0.8288670000	-6.9685060000	-6.6246510000
9	C	0.8979180000	-6.5347000000	-5.1600050000
10	C	2.2788220000	-6.7953660000	-4.5345270000
11	C	2.3029200000	-6.2041760000	-3.1237230000
12	C	3.6855140000	-6.2850750000	-2.4332960000
13	C	3.6618190000	-5.5695560000	-1.0606210000
14	C	3.5830260000	-5.1676610000	-11.2318460000
15	C	2.4114050000	-4.3844070000	-10.6469880000
16	C	2.6577690000	-4.0417150000	-9.1731400000
17	C	1.4805570000	-3.2549740000	-8.5842940000
18	C	1.7511580000	-2.8682130000	-7.1247340000
19	C	0.6061910000	-2.0184620000	-6.5580400000
20	C	0.9356150000	-1.5225860000	-5.1442840000
21	C	-0.1334270000	-0.5444100000	-4.6319850000
22	C	0.3074160000	0.0953220000	-3.3103990000
23	C	-0.6551190000	1.2163950000	-2.8698500000
24	C	-0.0387560000	2.1208620000	-1.7666440000
25	C	-9.2915010000	-12.6974610000	2.7004540000
26	C	-9.4519380000	-11.9494590000	1.3797800000
27	C	-8.7993540000	-10.5640850000	1.4464450000
28	C	-8.9658740000	-9.8107840000	0.1207980000
29	C	-8.3174600000	-8.4236250000	0.1953090000
30	C	-8.4915950000	-7.6553710000	-1.1210800000

1	C	-7.8525640000	-6.2654330000	-1.0253130000
2	C	-8.0372390000	-5.4692680000	-2.3239960000
3	C	-7.4100130000	-4.0766840000	-2.1920130000
4	C	-7.5985880000	-3.2456890000	-3.4725900000
5	C	-6.9153360000	-1.8600490000	-3.3964800000
6	C	4.1703060000	3.1597610000	-4.8818050000
7	C	10.0183210000	3.3569440000	-3.1233690000
8	C	-2.3307540000	-3.9127330000	-2.5307180000
9	C	4.6968560000	1.7824030000	-5.3097760000
10	C	6.2240280000	1.7189900000	-5.1159170000
11	C	6.8474430000	0.3277960000	-5.3716240000
12	C	9.3649290000	2.0047980000	-3.4256290000
13	C	-2.2636040000	2.3196580000	5.0027950000
14	C	-2.2094540000	1.3099450000	6.1609610000
15	C	-2.6122540000	1.8998710000	7.5365090000
16	C	-0.9130640000	-4.1116180000	-1.9879490000
17	C	-0.8302260000	-4.0886760000	-0.4440920000
18	C	0.6212470000	-4.3282960000	0.0385040000
19	C	3.5458350000	-4.0305140000	-1.2400120000
20	C	1.5200310000	-3.0923490000	-0.2007590000
21	C	10.2988750000	0.8356490000	-3.0990960000
22	C	9.7540850000	-0.5670420000	-3.4646440000
23	C	6.6249400000	-0.6825600000	-4.2201030000
24	C	8.5151780000	-1.0017640000	-2.6440970000
25	C	0.1419990000	1.4068360000	-0.3998270000
26	C	3.4851760000	-1.0083240000	-0.9346580000
27	C	3.7060850000	-2.0497970000	0.1896050000
28	C	3.2195800000	1.3896530000	-0.6432170000
29	C	5.1896710000	0.5463790000	-1.7396480000
30	C	2.3639300000	1.3825670000	0.6483160000

1	C	6.3276110000	-0.4927590000	-1.7844840000
2	C	-7.4983460000	-0.9436170000	-2.2901690000
3	C	0.5229020000	2.3657340000	1.8772350000
4	C	-0.8440660000	3.0502330000	1.8436770000
5	C	-1.8956810000	1.6591550000	3.6593630000
6	C	-3.2709600000	3.1875000000	2.2307270000
7	C	-3.6756990000	2.6731620000	0.8389770000
8	C	-5.0954500000	2.9667910000	0.4089920000
9	C	-4.8005300000	1.6733280000	-1.7030610000
10	C	-5.4374870000	0.2898110000	-1.8198680000
11	C	-7.6165640000	1.2507480000	-1.2949250000
12	C	-6.9824470000	2.6422810000	-1.1663060000
13	H	-11.5915310000	3.1379000000	9.1046520000
14	H	-9.8658650000	2.9791640000	8.8811610000
15	H	-10.6865020000	1.9982990000	10.0717610000
16	H	-11.9928960000	2.5889080000	6.8299280000
17	H	-10.1611240000	2.4306840000	6.5969530000
18	H	-12.2886850000	0.4344350000	6.0698760000
19	H	-10.4555960000	0.2388010000	5.8795800000
20	H	-15.5489610000	3.3136870000	-1.7065710000
21	H	-23.0368260000	-11.0651170000	11.7301820000
22	H	-21.4010300000	-10.9507830000	12.3336420000
23	H	-22.1398100000	-12.5173960000	12.1034320000
24	H	-22.2753720000	-12.0711620000	9.7032730000
25	H	-20.5403480000	-11.9498920000	10.3433400000
26	H	-22.4059980000	-9.7638900000	9.6192270000
27	H	-20.6714630000	-9.6420550000	10.2605120000
28	H	-21.6148110000	-10.7015110000	7.6585470000
29	H	-19.8803670000	-10.5779850000	8.2997540000
30	H	-20.1662620000	19.2183280000	-0.6494700000

1	H	-18.5296850000	19.2774780000	-0.0402220000
2	H	-19.7084890000	18.2368510000	0.7217390000
3	H	-19.6678030000	17.5472600000	-1.9781930000
4	H	-17.9319510000	17.6099980000	-1.3319870000
5	H	-20.2256070000	16.0230250000	-0.3298470000
6	H	-18.4888170000	16.0844960000	0.3139560000
7	H	-19.4675480000	15.0270300000	-2.2757440000
8	H	-17.7321530000	15.0846160000	-1.6278340000
9	H	-20.0295460000	13.5013130000	-0.6281160000
10	H	-18.2909710000	13.5545920000	0.0115870000
11	H	-21.7471330000	-8.3947300000	7.5695230000
12	H	-20.0139650000	-8.2697330000	8.2138860000
13	H	-12.4622950000	2.2407110000	0.0089900000
14	H	-14.0100900000	1.4961770000	0.7051940000
15	H	-11.3037970000	0.2832350000	0.5700370000
16	H	-12.8484500000	-0.3786460000	1.3514840000
17	H	-11.0337380000	1.6084390000	2.1184210000
18	H	-16.0762990000	0.9232970000	-4.1790280000
19	H	-15.7186950000	-0.6477440000	-3.2632500000
20	H	-18.0571650000	-0.1927720000	-4.1410810000
21	H	-17.3765040000	-0.9280990000	-1.3263720000
22	H	-19.0505220000	-1.1615380000	-2.0865500000
23	H	-19.5141920000	3.4676980000	-2.0922490000
24	H	-17.7683740000	3.3907730000	-1.4750770000
25	H	-19.1100820000	-3.0578680000	-0.7211730000
26	H	-17.3709810000	-2.9037820000	-0.0994930000
27	H	-18.9575560000	-5.3617110000	-0.5599130000
28	H	-17.2264810000	-5.1976280000	0.0813020000
29	H	-19.7618850000	-4.3570150000	1.3663940000
30	H	-18.0297790000	-4.2100240000	2.0089680000

1	H	-19.6179780000	-6.6620950000	1.5089350000
2	H	-17.8874590000	-6.5132090000	2.1553380000
3	H	-20.4228810000	-5.6846390000	3.4467050000
4	H	-18.6916870000	-5.5473920000	4.0938750000
5	H	-20.2849090000	-7.9902380000	3.5653760000
6	H	-18.5531050000	-7.8537060000	4.2110640000
7	H	-21.0849650000	-7.0321080000	5.5138930000
8	H	-19.3527350000	-6.9032180000	6.1600090000
9	H	-20.9510520000	-9.3387410000	5.6134740000
10	H	-19.2180480000	-9.2107490000	6.2576900000
11	H	-18.8800620000	5.0383380000	-3.6682310000
12	H	-17.1431490000	4.9737160000	-3.0250690000
13	H	-19.5798660000	5.9615380000	-1.6572700000
14	H	-17.8323230000	5.9246370000	-1.0412890000
15	H	-18.9691710000	7.5185570000	-3.2664270000
16	H	-17.2340400000	7.4988040000	-2.6155570000
17	H	-19.6945000000	8.4692810000	-1.2789480000
18	H	-17.9501770000	8.4740150000	-0.6528370000
19	H	-19.1058410000	10.0104370000	-2.9086300000
20	H	-17.3710670000	10.0282510000	-2.2567540000
21	H	-19.8487120000	10.9831220000	-0.9409230000
22	H	-18.1077020000	11.0178970000	-0.3065920000
23	H	-19.2762450000	12.5142570000	-2.5825720000
24	H	-17.5413560000	12.5573560000	-1.9321860000
25	H	22.0639170000	-4.5528570000	-2.9451810000
26	H	23.3696470000	-3.9737400000	-3.9515410000
27	H	23.6620130000	-5.2408490000	-2.7844830000
28	H	23.1128670000	-3.6780640000	-0.9887720000
29	H	24.4978030000	-3.0638180000	-2.0561780000
30	H	21.6664330000	-2.1888710000	-1.9948160000

1	H	23.0360990000	-1.5879780000	-3.0891840000
2	H	22.7617320000	-1.3127930000	-0.1522390000
3	H	24.1171770000	-0.6972310000	-1.2561210000
4	H	21.3049970000	0.2070000000	-1.0433220000
5	H	22.5925860000	0.7692550000	-2.2519090000
6	H	22.6022390000	1.0751990000	0.6953060000
7	H	23.8088850000	1.7151990000	-0.5573330000
8	H	20.9841920000	2.5201670000	-0.1729690000
9	H	22.2575650000	3.2092090000	-1.3298790000
10	H	22.1175110000	3.3865730000	1.6263180000
11	H	23.4561560000	4.0096640000	0.5062790000
12	H	22.1112310000	5.8092760000	-0.1486650000
13	H	20.1471850000	6.4142240000	0.8731780000
14	H	19.5559190000	5.2382930000	2.9454920000
15	H	20.2032000000	3.6807250000	2.1775660000
16	H	18.4675210000	3.6242600000	0.7125500000
17	H	17.8807270000	5.2868870000	1.2835500000
18	H	17.7768750000	2.6784350000	2.6857760000
19	H	17.2613660000	4.3406840000	3.3228600000
20	H	16.0778540000	2.6919480000	1.1656170000
21	H	15.6168040000	4.4202160000	1.6506140000
22	H	15.2697040000	1.8869370000	3.1458350000
23	H	14.9124860000	3.6091350000	3.7298960000
24	H	13.6044370000	2.0687330000	1.5612430000
25	H	13.2611160000	3.8052190000	2.1102840000
26	H	28.2820180000	-6.0769620000	-2.8202500000
27	H	29.4359890000	-6.3503790000	-1.5370270000
28	H	29.0528820000	-4.7198520000	-2.0347010000
29	H	26.6339330000	-5.9395930000	-1.3810590000
30	H	27.8579040000	-6.2295940000	-0.0199960000

H	26.9522650000	-3.6573860000	-1.1807020000
H	28.1754320000	-3.9477580000	0.1810060000
H	25.2628000000	-4.4940850000	0.1789440000
H	26.4931900000	-4.7242490000	1.5456430000
H	25.5981000000	-2.1840910000	0.3105880000
H	26.7326230000	-2.4571350000	1.7503820000
H	23.8228650000	-2.9861330000	1.5661840000
H	24.9607730000	-3.2311980000	3.0083390000
H	24.1670030000	-0.6811560000	1.7248730000
H	25.2123900000	-0.9649130000	3.2286510000
H	22.3216010000	-1.5276070000	2.8914410000
H	23.3813380000	-1.6749870000	4.4046950000
H	23.4996930000	0.7486280000	4.5073860000
H	21.6719780000	1.9652930000	3.8076210000
H	21.0196630000	-0.3830730000	2.0305760000
H	20.3289310000	1.3355390000	1.9676240000
H	19.7442370000	-0.8405370000	3.8932420000
H	19.0790190000	0.8891550000	3.8745150000
H	18.6050240000	-1.3163330000	1.9506610000
H	17.9775530000	0.4259520000	1.8770100000
H	17.3393180000	-1.7017760000	3.8382660000
H	16.7307960000	0.0482620000	3.7961940000
H	16.1431800000	-2.1923610000	1.9479760000
H	15.6038000000	-0.4252000000	1.8033610000
H	14.8860100000	-2.4186860000	3.8751120000
H	14.3472770000	-0.6512450000	3.7315250000
H	13.6164480000	-3.0104980000	2.1021580000
H	13.2428740000	-1.2367320000	1.7162860000
H	12.8117400000	1.2790290000	3.5882390000
H	12.4468930000	3.0176720000	4.1161650000

1	H	9.7694920000	-1.0088360000	3.8542000000
2	H	10.8612160000	-1.1425500000	5.3458300000
3	H	11.2880420000	1.1869890000	5.1828930000
4	H	8.3695190000	0.5130570000	5.1259120000
5	H	9.1250040000	2.0652410000	5.8002010000
6	H	6.4827060000	-0.4518140000	10.7313710000
7	H	7.3594510000	-0.0736600000	9.4066130000
8	H	5.2357720000	1.6226170000	9.3320330000
9	H	5.9602720000	1.6347020000	11.0378040000
10	H	6.9128460000	2.7853150000	8.5753640000
11	H	7.5522580000	2.9271820000	10.3090670000
12	H	6.4997630000	1.5587210000	7.1749110000
13	H	7.9960030000	11.8912760000	-5.2597200000
14	H	7.3005950000	11.9828000000	-3.5901710000
15	H	8.7428640000	12.9764350000	-4.0263310000
16	H	10.1071770000	10.9240660000	-4.3258160000
17	H	9.4141210000	11.0141100000	-2.6591190000
18	H	8.2615720000	9.3694540000	-5.0107700000
19	H	7.5678700000	9.4554450000	-3.3426090000
20	H	10.3850440000	8.4216500000	-4.0792850000
21	H	9.6905740000	8.4989470000	-2.4108520000
22	H	8.5406260000	6.8822110000	-4.7784720000
23	H	7.8478650000	6.9460400000	-3.1092350000
24	H	10.6681820000	5.9321890000	-3.8614180000
25	H	9.9724970000	5.9748330000	-2.1919700000
26	H	8.8262070000	4.4122550000	-4.5960660000
27	H	8.1353270000	4.4275270000	-2.9257740000
28	H	-3.9861150000	6.4178710000	-3.0152090000
29	H	-3.8973990000	7.3030650000	-4.5926070000
30	H	-3.9415740000	8.2214660000	-3.0393300000

H	-1.7300690000	7.2998110000	-2.3932160000
H	-1.6348830000	8.1838120000	-3.9670010000
H	-1.8898630000	5.1234850000	-3.6239330000
H	-1.7800530000	5.9998970000	-5.2016180000
H	0.3548100000	6.0131810000	-2.9702070000
H	0.4887070000	6.8704160000	-4.5579860000
H	0.1532460000	3.8386480000	-4.1579300000
H	0.3305950000	4.6584090000	-5.7519710000
H	2.6097990000	5.4514950000	-5.0778300000
H	2.3950760000	4.6866120000	-3.4523640000
H	2.3989210000	3.1621470000	-6.1430040000
H	2.1634900000	2.4490190000	-4.5011950000
H	-5.8332020000	-4.5445210000	-10.8733970000
H	-5.2848270000	-2.8228540000	-10.9943660000
H	-7.0441850000	-3.2205530000	-11.0530880000
H	-6.9700760000	-4.0328300000	-8.7046170000
H	-6.4329790000	-2.3115020000	-8.8272360000
H	-4.5687170000	-4.7744680000	-8.6615680000
H	-4.0397280000	-3.0499510000	-8.7871120000
H	-5.7203100000	-4.2694570000	-6.4948950000
H	-5.2132210000	-2.5382230000	-6.6269150000
H	-3.3052690000	-4.9633170000	-6.4711860000
H	-2.8162370000	-3.2291440000	-6.6142030000
H	-4.4320830000	-4.4689060000	-4.2847450000
H	-3.9661360000	-2.7256090000	-4.4450480000
H	-1.9866920000	-5.0871440000	-4.3264950000
H	-1.5694780000	-3.3428970000	-4.4834750000
H	4.6701430000	-5.6282220000	0.5952420000
H	-4.0937310000	-6.5467960000	-11.0051390000
H	-3.2153280000	-6.2902130000	-12.5611870000

1	H	-3.2587360000	-4.9725630000	-11.3292560000
2	H	-1.8785170000	-7.7207520000	-11.0280340000
3	H	-1.0524910000	-6.1461460000	-11.3530520000
4	H	-2.7437970000	-6.8168710000	-8.8485350000
5	H	-1.9290170000	-5.2384520000	-9.1885350000
6	H	-0.5048530000	-7.9697890000	-8.8732630000
7	H	0.2870150000	-6.3897140000	-9.2421370000
8	H	-1.3499660000	-7.0366140000	-6.7035650000
9	H	-0.6193220000	-5.4374250000	-7.1150440000
10	H	0.9617280000	-8.0692900000	-6.7058660000
11	H	1.6485700000	-6.4678100000	-7.1815330000
12	H	0.1075960000	-7.0531320000	-4.5735650000
13	H	0.6996820000	-5.4399130000	-5.1210440000
14	H	2.5036600000	-7.8835060000	-4.5096340000
15	H	3.0512350000	-6.2794340000	-5.1470020000
16	H	1.5449700000	-6.7194660000	-2.4936450000
17	H	1.9871630000	-5.1530640000	-3.2351300000
18	H	3.9251540000	-7.3608260000	-2.2858760000
19	H	4.4767430000	-5.8340660000	-3.0716370000
20	H	2.8179140000	-6.0241350000	-0.5137660000
21	H	-1.7380670000	3.0978550000	-1.7426290000
22	H	3.3806840000	-5.3993730000	-12.2987000000
23	H	4.5172400000	-4.5696360000	-11.1690400000
24	H	3.7225820000	-6.1218450000	-10.6804150000
25	H	1.4882010000	-4.9935340000	-10.7453470000
26	H	2.2800880000	-3.4470700000	-11.2305430000
27	H	2.8050220000	-4.9775030000	-8.5934020000
28	H	3.5828960000	-3.4284400000	-9.0961820000
29	H	0.5522300000	-3.8626470000	-8.6453970000
30	H	1.3378370000	-2.3276100000	-9.1820650000

1	H	1.8874000000	-3.7806940000	-6.5051250000
2	H	2.6901190000	-2.2720550000	-7.0896260000
3	H	-0.3393820000	-2.6022390000	-6.5513310000
4	H	0.4735020000	-1.1322820000	-7.2177400000
5	H	1.0448010000	-2.3801600000	-4.4445680000
6	H	1.9067570000	-0.9804670000	-5.1934070000
7	H	-1.1152540000	-1.0489260000	-4.5139130000
8	H	-0.2423870000	0.2670330000	-5.3855110000
9	H	0.4086300000	-0.6809870000	-2.5250690000
10	H	1.3057510000	0.5463720000	-3.4887600000
11	H	-0.8253490000	1.8673080000	-3.7519110000
12	H	-1.6389990000	0.7988890000	-2.5658940000
13	H	0.9392720000	2.4410360000	-2.1713770000
14	H	-7.9546690000	-1.0862230000	-4.8558780000
15	H	-8.2151510000	-12.8386080000	2.9363730000
16	H	-9.7727840000	-13.6951940000	2.6243520000
17	H	-9.7747470000	-12.1319580000	3.5257100000
18	H	-8.9801080000	-12.5464380000	0.5691130000
19	H	-10.5363440000	-11.8415020000	1.1590140000
20	H	-7.7156340000	-10.6789520000	1.6704470000
21	H	-9.2728970000	-9.9761880000	2.2637610000
22	H	-8.4917090000	-10.3952060000	-0.6983210000
23	H	-10.0499160000	-9.6969490000	-0.1013380000
24	H	-7.2326650000	-8.5361430000	0.4151530000
25	H	-8.7906420000	-7.8453520000	1.0196970000
26	H	-8.0158550000	-8.2250720000	-1.9495400000
27	H	-9.5769210000	-7.5448060000	-1.3387130000
28	H	-6.7660900000	-6.3740440000	-0.8112570000
29	H	-8.3268570000	-5.7071510000	-0.1876820000
30	H	-7.5582220000	-6.0150650000	-3.1665870000

1	H	-9.1238000000	-5.3623200000	-2.5366290000
2	H	-6.3224290000	-4.1859120000	-1.9837820000
3	H	-7.8923700000	-3.5646480000	-1.3345920000
4	H	-7.1400120000	-3.8048880000	-4.3178230000
5	H	-8.6829350000	-3.1211820000	-3.6851340000
6	H	-5.8476410000	-2.0607190000	-3.1886880000
7	H	4.6700940000	3.9577910000	-5.4739850000
8	H	4.4134870000	3.3077900000	-3.8056560000
9	H	10.9595150000	3.4450940000	-3.7100280000
10	H	10.2624450000	3.4232560000	-2.0403800000
11	H	-3.0190780000	-4.6734510000	-2.1020500000
12	H	-2.6879700000	-2.8988670000	-2.2445850000
13	H	4.4528500000	1.5965580000	-6.3784080000
14	H	4.1823600000	1.0039630000	-4.7191790000
15	H	6.6657870000	2.4405920000	-5.8379260000
16	H	6.5029680000	2.0544570000	-4.0965050000
17	H	6.7949980000	0.2713860000	-7.3140080000
18	H	7.9375210000	0.4476820000	-5.4726320000
19	H	9.1323150000	1.9837340000	-4.5074370000
20	H	8.4369220000	1.9253170000	-2.8269870000
21	H	-3.2829120000	2.7518680000	4.9649540000
22	H	-1.5398460000	3.1389610000	5.1783550000
23	H	-1.1859710000	0.8810410000	6.2304160000
24	H	-2.9121960000	0.4820350000	5.9198410000
25	H	-4.5353670000	1.7372720000	7.2582580000
26	H	-0.9639010000	-0.8352500000	8.2069090000
27	H	-4.0209130000	1.2911140000	10.3213580000
28	H	-2.6357120000	4.3563150000	9.5659200000
29	H	0.5344710000	2.6544290000	7.2445000000
30	H	-0.5385000000	-5.0986050000	-2.3395310000

1	H	-0.2798970000	-3.3214350000	-2.4346880000
2	H	-1.4645380000	-4.9256630000	-0.0769400000
3	H	-1.2284210000	-3.1394680000	-0.0237170000
4	H	1.4595760000	-5.1267980000	1.5930630000
5	H	0.9383800000	-5.2105440000	-0.5397150000
6	H	2.9621230000	-3.7354440000	-2.1269430000
7	H	4.5693120000	-3.6778470000	-1.5031590000
8	H	1.3258890000	-2.6913140000	-1.2107250000
9	H	1.1733390000	-2.3079220000	0.5103720000
10	H	10.6199490000	-2.2884180000	-3.7494060000
11	H	11.2381310000	0.9849130000	-3.6769960000
12	H	10.5553960000	0.8608620000	-2.0170710000
13	H	9.5443600000	-0.6024690000	-4.5527320000
14	H	7.0233210000	-1.6700190000	-4.5442680000
15	H	5.5309530000	-0.8475740000	-4.1257700000
16	H	8.3355810000	-2.0905940000	-2.7978010000
17	H	8.8196940000	-0.8922460000	-1.5787960000
18	H	0.4982610000	0.3724340000	-0.5661240000
19	H	-0.8665180000	1.2563580000	0.0421360000
20	H	3.7584390000	-1.4425070000	-1.9196550000
21	H	2.3997470000	-0.8361190000	-1.0364020000
22	H	4.7944790000	-2.2464730000	0.3035410000
23	H	3.3697120000	-1.6208530000	1.1572130000
24	H	2.5952950000	1.2898450000	-1.5474930000
25	H	3.7045500000	2.3890020000	-0.7150870000
26	H	4.6549380000	0.5759850000	-2.6990810000
27	H	5.6616570000	1.5433450000	-1.5901790000
28	H	2.1138550000	0.3432800000	0.9505740000
29	H	3.0043610000	1.7857050000	1.4652800000
30	H	5.9534730000	-1.5371250000	-1.8389610000

H	6.8505010000	-0.3879330000	-0.8076120000
H	-7.4078720000	-1.4300620000	-1.2956500000
H	-8.5834680000	-0.8213930000	-2.5073410000
H	-2.5843470000	0.8166230000	3.4304030000
H	-0.8987370000	1.2288700000	3.8185410000
H	-4.0438250000	2.8042230000	2.9296250000
H	-3.2764130000	4.2978210000	2.2871400000
H	-3.5700650000	1.5659200000	0.8670300000
H	-2.9912100000	3.0909280000	0.0832560000
H	-4.7951070000	2.1697350000	-2.6986030000
H	-3.7442150000	1.5460860000	-1.4022730000
H	-4.8395840000	-0.2691840000	-2.5667810000
H	-5.3596010000	-0.2270760000	-0.8355700000
H	-8.6647730000	1.3751960000	-1.6453740000
H	-7.6386300000	0.7809210000	-0.2840290000
H	-7.0267090000	3.1673020000	-2.1460020000
H	-7.5658020000	3.2412720000	-0.4346510000
H	1.3030800000	3.0936190000	0.1258310000
H	0.5140150000	1.4014150000	2.4093130000
H	1.2108100000	3.0425570000	2.4325790000

6.5. Piperazine amine core based bone-targeting ionizable lipids ‘Type 1’

Type1-P1-C12

P	-21.0396350000	2.4167870000	1.3401910000
P	6.7585160000	-8.2584450000	-0.2499600000
P	-5.2767430000	-6.2618660000	-2.1651550000
P	-3.1107520000	-6.3023490000	-0.0495850000
O	-14.2287420000	-5.4323040000	-1.3535130000
O	-16.6947340000	-1.1704980000	-2.2042120000
O	-21.1413070000	1.4116280000	2.7548680000

1	O	-20.9643030000	3.8127520000	1.8497370000
2	O	-19.8590650000	1.9968840000	0.3350080000
3	O	-22.5411630000	2.0631840000	0.7988770000
4	O	-25.1409360000	1.4585170000	0.9119570000
5	O	-25.1128080000	2.8459400000	2.7242220000
6	O	-24.5934580000	2.7867020000	-2.3697220000
7	O	-24.3557110000	3.3798600000	-4.5298540000
8	O	-15.9593400000	-2.4014840000	-0.4639390000
9	O	11.2339860000	-4.0725830000	-2.2925370000
10	O	11.2799110000	-4.7784630000	-0.1261910000
11	O	10.7247370000	-3.9803560000	3.8307950000
12	O	9.6907950000	-5.0672640000	2.1595810000
13	O	5.2507500000	-7.5413810000	0.2280700000
14	O	8.1365370000	-7.5381890000	0.0510900000
15	O	6.4231420000	-9.7356700000	0.3542440000
16	O	6.3712340000	-8.4493080000	-1.6733350000
17	O	11.7324660000	3.2616200000	1.6789180000
18	O	13.8792470000	1.6987310000	-2.2524440000
19	O	10.4198590000	1.7126490000	-6.2934720000
20	O	-2.8280950000	3.1056380000	-2.5938850000
21	O	-5.6300840000	-5.5356860000	0.3987520000
22	O	-4.2164730000	-6.1900480000	-3.2975100000
23	O	-5.5755510000	-7.9633720000	-1.9977870000
24	O	-6.7054950000	-5.5769320000	-2.8783160000
25	O	-1.9861970000	-6.1782520000	-1.1135700000
26	O	-2.3602660000	-5.7172180000	1.4022540000
27	O	-3.2926390000	-8.0177780000	0.1680640000
28	O	-12.9217870000	0.6866870000	0.1076780000
29	O	-1.7362540000	0.3946850000	2.1321410000
30	O	-6.6049220000	-1.4694780000	0.8436420000

1	N	-17.2685440000	-0.5452850000	-0.1128650000
2	N	4.5498900000	-9.3858120000	-1.1721390000
3	N	12.3279220000	3.7829460000	-1.0306030000
4	N	9.1652920000	2.8415010000	-2.9710010000
5	N	-0.5941430000	0.9379250000	-0.5078100000
6	N	-3.3037090000	-0.8219750000	1.0564340000
7	N	-7.5606790000	0.5129980000	0.3926220000
8	N	-10.0648200000	1.8429650000	0.3099920000
9	N	5.3463380000	2.7874590000	-2.7766240000
10	N	2.5772840000	1.9734120000	-2.4740360000
11	C	-13.6091010000	-6.4842130000	-0.6447710000
12	C	-14.7948320000	-4.4734290000	-0.4936900000
13	C	-15.4326960000	-3.3735900000	-1.3296020000
14	C	-23.8335210000	-9.2524710000	-15.3943100000
15	C	-23.8807510000	-7.7664370000	-15.0150050000
16	C	-23.9167550000	-7.5656590000	-13.4915710000
17	C	-23.9630120000	-6.0774910000	-13.1087920000
18	C	-24.2023270000	-5.8290390000	16.9745620000
19	C	-24.2534920000	-4.5320570000	16.1560720000
20	C	-24.2613220000	-4.8091850000	14.6442300000
21	C	-24.3147730000	-3.5111370000	13.8222390000
22	C	-24.3271740000	-3.7859170000	12.3095000000
23	C	-24.0003810000	-5.8745610000	-11.5851520000
24	C	-19.1237630000	0.8318280000	0.6073530000
25	C	-18.0689040000	0.6252460000	-0.4783640000
26	C	-23.4056330000	3.0778920000	0.3577130000
27	C	-24.7535640000	2.4463230000	-0.0080800000
28	C	-24.7110300000	1.7899430000	-1.3896720000
29	C	-25.0906720000	1.7339120000	2.2469940000
30	C	-24.9386110000	0.4456800000	3.0404750000

1	C	-24.4386000000	2.5013080000	-3.6966600000
2	C	-24.3773990000	1.0206490000	-4.0607980000
3	C	-24.3087240000	0.7657580000	-5.5774860000
4	C	-24.2658670000	-0.7337560000	-5.9170680000
5	C	-24.2107530000	-0.9690900000	-7.4355570000
6	C	-24.1709150000	-2.4637800000	-7.7928140000
7	C	-24.1250060000	-2.6841080000	-9.3139360000
8	C	-24.0835380000	-4.1756730000	-9.6838440000
9	C	-24.0437230000	-4.3851210000	-11.2066280000
10	C	-24.8338630000	0.6732960000	4.5566500000
11	C	-24.7039830000	-0.6450330000	5.3371740000
12	C	-24.6313160000	-0.4003420000	6.8534300000
13	C	-24.5323570000	-1.7103490000	7.6518890000
14	C	-24.4882050000	-1.4510020000	9.1668820000
15	C	-24.4125160000	-2.7548580000	9.9776760000
16	C	-24.3881870000	-2.4857430000	11.4913770000
17	C	-16.6385200000	-1.3542770000	-1.0110210000
18	C	24.0896940000	-0.5461360000	-1.8058440000
19	C	24.1117330000	-1.9445270000	-1.1764260000
20	C	23.3824710000	-1.9838400000	0.1761290000
21	C	23.4372850000	-3.3855090000	0.8028590000
22	C	22.7015580000	-3.4623500000	2.1496930000
23	C	22.8616680000	-4.8459050000	2.8001060000
24	C	22.0583090000	-4.9743410000	4.1035550000
25	C	22.1510320000	-6.3909350000	4.6896440000
26	C	21.4197580000	-6.4772930000	6.0050730000
27	C	20.1415600000	-6.8427870000	6.1936360000
28	C	19.1373250000	-7.2456230000	5.1405790000
29	C	17.9531390000	-6.2652980000	5.1231380000
30	C	16.8149680000	-6.7202890000	4.1936290000

1	C	15.6189290000	-5.7555100000	4.2583740000
2	C	14.4086250000	-6.2442930000	3.4450540000
3	C	13.1983750000	-5.3105980000	3.6229440000
4	C	29.2262790000	-2.4982930000	-3.7068730000
5	C	27.8645460000	-3.1625800000	-3.9486370000
6	C	27.4411590000	-4.0450380000	-2.7638000000
7	C	26.0768140000	-4.7110240000	-3.0032340000
8	C	25.6316960000	-5.5567380000	-1.7991780000
9	C	24.2252560000	-6.1434010000	-1.9983030000
10	C	23.7684940000	-6.9720360000	-0.7866750000
11	C	22.3284920000	-7.4807450000	-0.9535440000
12	C	21.8629260000	-8.2024170000	0.2866820000
13	C	20.9092350000	-7.7946120000	1.1398200000
14	C	20.0982870000	-6.5256590000	1.0436560000
15	C	18.7351770000	-6.8067290000	0.3989480000
16	C	17.8644870000	-5.5425540000	0.3205320000
17	C	16.4857920000	-5.8553850000	-0.2799110000
18	C	15.5792820000	-4.6160030000	-0.3458460000
19	C	14.1793810000	-4.9831820000	-0.8610850000
20	C	13.2406830000	-3.7692330000	-0.9409300000
21	C	11.8135260000	-4.2003510000	-1.2380550000
22	C	11.9580850000	-5.7882170000	2.8452050000
23	C	10.7553590000	-4.8593970000	2.9938350000
24	C	9.9824820000	-5.3003510000	-0.2040370000
25	C	9.6452460000	-5.9996200000	1.1151580000
26	C	8.2252940000	-6.5734480000	1.0689270000
27	C	4.0683330000	-9.9119600000	0.1749020000
28	C	5.2894340000	-10.0413010000	1.1107210000
29	C	7.8930490000	15.0469020000	-4.2211590000
30	C	7.6659370000	13.8172810000	-5.0961350000

1	C	8.2062820000	12.5517710000	-4.4211070000
2	C	7.9855000000	11.3163050000	-5.3031560000
3	C	8.5416420000	10.0583830000	-4.6265610000
4	C	8.3498360000	8.8162560000	-5.5068940000
5	C	8.9441340000	7.5790240000	-4.8251580000
6	C	26.2155240000	7.1130500000	0.6915450000
7	C	25.3067260000	6.2615120000	-0.1895650000
8	C	23.8585870000	6.2920490000	0.3135080000
9	C	22.9538220000	5.4325490000	-0.5760970000
10	C	21.5025210000	5.4554250000	-0.0806550000
11	C	20.6123120000	4.5877770000	-0.9768370000
12	C	19.1559000000	4.5948400000	-0.4971720000
13	C	20.8085050000	13.3733060000	3.7817730000
14	C	20.5584860000	11.8682020000	3.8294550000
15	C	19.2025070000	11.5160600000	3.2092090000
16	C	18.9403700000	10.0054070000	3.2648140000
17	C	17.5747470000	9.6739260000	2.6546980000
18	C	17.2702470000	8.1717920000	2.7258800000
19	C	15.8834200000	7.8881030000	2.1400980000
20	C	-18.3828720000	13.2476050000	-1.1436520000
21	C	-18.5232800000	11.8068770000	-0.6605240000
22	C	-17.2012240000	11.0443860000	-0.8033050000
23	C	-17.3469110000	9.5944330000	-0.3258600000
24	C	-16.0258720000	8.8315260000	-0.4792150000
25	C	-16.1720190000	7.3736670000	-0.0258560000
26	C	-14.8533890000	6.6122860000	-0.2050310000
27	C	-1.4958710000	7.3051110000	10.4374130000
28	C	-2.3403570000	7.2131620000	9.1696810000
29	C	-1.6231850000	6.4005500000	8.0859740000
30	C	-2.4717110000	6.3101590000	6.8117390000

1	C	-1.7490550000	5.4980720000	5.7310220000
2	C	-2.5881460000	5.4058060000	4.4506670000
3	C	-1.8522760000	4.5951680000	3.3781150000
4	C	18.2899210000	3.7147070000	-1.4053880000
5	C	15.5057170000	6.4044010000	2.2373260000
6	C	14.0917850000	6.1945080000	1.6848350000
7	C	13.6214850000	4.7359100000	1.8204600000
8	C	12.1560630000	4.5647990000	1.3695620000
9	C	16.8265950000	3.7014730000	-0.9488620000
10	C	15.9890990000	2.8013150000	-1.8722060000
11	C	14.5096610000	2.6627470000	-1.4464460000
12	C	11.8567980000	4.8643650000	-0.1213690000
13	C	13.7282630000	3.9958490000	-1.4786730000
14	C	8.8083450000	6.3248640000	-5.6998430000
15	C	9.4654230000	5.1258240000	-5.0063590000
16	C	9.4269480000	3.8585880000	-5.8800960000
17	C	10.2312790000	2.6755570000	-5.2860680000
18	C	9.5570200000	1.9479710000	-4.0938640000
19	C	-2.6742440000	4.4983120000	2.0868270000
20	C	-1.9175380000	3.6871120000	1.0276050000
21	C	-2.7217860000	3.5750480000	-0.2706420000
22	C	-1.9946690000	2.9219000000	-1.4751420000
23	C	-1.7222280000	1.3871140000	-1.3858880000
24	C	11.4269490000	3.6337350000	-2.2027730000
25	C	10.1507890000	2.8473740000	-1.8557020000
26	C	7.8202340000	2.5106540000	-2.4330760000
27	C	-3.9515450000	-3.0859320000	0.2868950000
28	C	-4.3912510000	-3.9189980000	-0.9278110000
29	C	-4.6627560000	-5.4103650000	-0.6117200000
30	C	-14.9986390000	5.1413880000	0.2058700000

1	C	-13.6836740000	4.3831900000	-0.0156640000
2	C	-13.8351890000	2.8906920000	0.3287930000
3	C	-12.5844400000	2.0451860000	-0.0150500000
4	C	-11.3367460000	2.3906830000	0.8447040000
5	C	-0.9288820000	-0.3841580000	0.0978270000
6	C	-2.0367040000	-0.2613220000	1.1445270000
7	C	-3.7345410000	-1.6151170000	-0.1019820000
8	C	-4.2701610000	-0.5817110000	2.1521840000
9	C	-5.4136600000	0.3685710000	1.7279280000
10	C	-6.5629710000	-0.2522740000	0.9520990000
11	C	-8.6705370000	-0.0910820000	-0.3324900000
12	C	-9.9910620000	0.3630400000	0.3019100000
13	C	-8.9301820000	2.3969750000	1.0876500000
14	C	-7.5984650000	1.9684110000	0.4692450000
15	C	0.6972740000	0.7852340000	-1.2420970000
16	C	1.2606240000	2.1086150000	-1.8022180000
17	C	6.6770570000	2.8391240000	-3.4224430000
18	C	3.6394880000	1.4982330000	-1.5597950000
19	C	4.9919650000	1.4444920000	-2.2791920000
20	C	4.3102250000	3.2355000000	-3.7263270000
21	C	2.9527570000	3.2961820000	-3.0237370000
22	H	-14.3340090000	-6.9757330000	-0.0301040000
23	H	-12.8288640000	-6.0882640000	-0.0288530000
24	H	-13.1947580000	-7.1859590000	-1.3381470000
25	H	-15.5870000000	-4.9601660000	0.0358910000
26	H	-13.9871650000	-4.0246850000	0.0459420000
27	H	-16.2604590000	-3.8137240000	-1.8453440000
28	H	-14.6454110000	-2.9054720000	-1.8827280000
29	H	-22.0380260000	1.0821980000	2.8495780000
30	H	-24.7022110000	-9.7460920000	-15.0114050000

1	H	-22.9558810000	-9.6995590000	-14.9762650000
2	H	-23.8095350000	-9.3480310000	-16.4597640000
3	H	-24.8103940000	-7.3910710000	-15.3888540000
4	H	-22.9581320000	-7.3417150000	-15.3515830000
5	H	-24.8397170000	-7.9900080000	-13.1554610000
6	H	-22.9874210000	-7.9420690000	-13.1180020000
7	H	-24.8919170000	-5.7011050000	-13.4834510000
8	H	-23.0396440000	-5.6535890000	-13.4443500000
9	H	-25.0675530000	-5.8897400000	17.6011390000
10	H	-23.3212770000	-5.8331110000	17.5817140000
11	H	-24.1824750000	-6.6686430000	16.3115610000
12	H	-25.1930840000	-4.0723730000	16.3813640000
13	H	-23.3408780000	-4.0123080000	16.3607600000
14	H	-25.1731890000	-5.3304900000	14.4401710000
15	H	-23.3210820000	-5.2675990000	14.4190500000
16	H	-25.2536290000	-3.0513030000	14.0502810000
17	H	-23.4014820000	-2.9915260000	14.0242390000
18	H	-25.2379630000	-4.3099520000	12.1076300000
19	H	-23.3861780000	-4.2405400000	12.0798410000
20	H	-24.9247010000	-6.2961300000	-11.2492750000
21	H	-23.0723480000	-6.2522890000	-11.2096830000
22	H	-18.5954210000	1.0072680000	1.5211230000
23	H	-19.8070120000	0.0117050000	0.5333970000
24	H	-17.4173010000	1.4734670000	-0.4494850000
25	H	-18.5896400000	0.3811120000	-1.3806580000
26	H	-16.5165590000	-0.1572510000	0.4200020000
27	H	-23.5799250000	3.7243840000	1.1923210000
28	H	-22.9896350000	3.4714400000	-0.5461490000
29	H	-25.4588050000	3.2509530000	0.0021310000
30	H	-23.8134990000	1.2087650000	-1.4293110000

1	H	-25.6602730000	1.3191390000	-1.5386030000
2	H	-25.8446630000	-0.1025380000	2.8874200000
3	H	-24.0015590000	0.0248120000	2.7409670000
4	H	-25.3050420000	0.5992930000	-3.7339400000
5	H	-23.4541440000	0.6575970000	-3.6599310000
6	H	-25.2263380000	1.1370280000	-5.9837420000
7	H	-23.3750170000	1.1722980000	-5.9058420000
8	H	-25.1956020000	-1.1444690000	-5.5826920000
9	H	-23.3437290000	-1.1053930000	-5.5215330000
10	H	-25.1319540000	-0.5930270000	-7.8290850000
11	H	-23.2800150000	-0.5584520000	-7.7672260000
12	H	-25.0989090000	-2.8775310000	-7.4573490000
13	H	-23.2466780000	-2.8402340000	-7.4068500000
14	H	-25.0497250000	-2.3070020000	-9.6981050000
15	H	-23.1975230000	-2.2679690000	-9.6478580000
16	H	-25.0091970000	-4.5939910000	-9.3475910000
17	H	-23.1568480000	-4.5531090000	-9.3047820000
18	H	-24.9709770000	-4.0077730000	-11.5843950000
19	H	-23.1186720000	-3.9646730000	-11.5418930000
20	H	-25.7643230000	1.1074280000	4.8577700000
21	H	-23.9190690000	1.2050650000	4.7156290000
22	H	-25.6094680000	-1.1879350000	5.1632400000
23	H	-23.7630790000	-1.0697170000	5.0556730000
24	H	-25.5639760000	0.0463620000	7.1282030000
25	H	-23.7150900000	0.1243490000	7.0270090000
26	H	-25.4400770000	-2.2451890000	7.4651010000
27	H	-23.5906520000	-2.1484290000	7.3946390000
28	H	-25.4235170000	-0.9966480000	9.4191780000
29	H	-23.5725810000	-0.9298550000	9.3538010000
30	H	-25.3220910000	-3.2831780000	9.7815850000

1	H	-23.4710510000	-3.2030810000	9.7375940000
2	H	-25.3255370000	-2.0273770000	11.7283910000
3	H	-23.4737040000	-1.9660640000	11.6877270000
4	H	23.0760940000	-0.2429400000	-1.9658070000
5	H	24.5702530000	0.1483340000	-1.1488240000
6	H	24.6073580000	-0.5681830000	-2.7420260000
7	H	23.5537270000	-2.5769940000	-1.8348450000
8	H	25.1385220000	-2.1619860000	-0.9683030000
9	H	22.3514980000	-1.7866610000	-0.0315120000
10	H	23.9289780000	-1.3388510000	0.8320400000
11	H	22.9029040000	-4.0294630000	0.1360310000
12	H	24.4684660000	-3.5742350000	1.0172140000
13	H	21.6601120000	-3.3511120000	1.9307960000
14	H	23.1890630000	-2.7658430000	2.7993990000
15	H	22.4334520000	-5.5481930000	2.1157660000
16	H	23.8922320000	-4.9279810000	3.0759740000
17	H	21.0321170000	-4.8248220000	3.8399770000
18	H	22.5288860000	-4.3247960000	4.8117570000
19	H	21.6317000000	-7.0347520000	4.0109000000
20	H	23.1848460000	-6.5630530000	4.9052930000
21	H	21.9781820000	-6.2202190000	6.8808440000
22	H	19.7950290000	-6.8493090000	7.2059470000
23	H	18.7437180000	-8.1940130000	5.4414620000
24	H	19.6295980000	-7.1460740000	4.1957730000
25	H	18.3292250000	-5.3543480000	4.7064500000
26	H	17.5476790000	-6.2733890000	6.1133080000
27	H	17.1976600000	-6.6597320000	3.1962420000
28	H	16.4681220000	-7.6568460000	4.5776330000
29	H	15.9438690000	-4.8503280000	3.7893760000
30	H	15.3030190000	-5.7448680000	5.2806200000

H	14.6911710000	-6.1787020000	2.4151190000
H	14.1255870000	-7.1876890000	3.8631390000
H	13.4794510000	-4.3749160000	3.1866030000
H	12.9356390000	-5.3603270000	4.6589930000
H	29.1140390000	-1.4345420000	-3.7340270000
H	29.9125730000	-2.8040880000	-4.4687060000
H	29.6018860000	-2.7929800000	-2.7492830000
H	27.1489720000	-2.3688610000	-4.0021470000
H	27.9959460000	-3.8214830000	-4.7813930000
H	27.3106690000	-3.3859270000	-1.9310670000
H	28.1567170000	-4.8388080000	-2.7108360000
H	25.3663400000	-3.9156110000	-3.0895180000
H	26.2155940000	-5.3947570000	-3.8144980000
H	25.5608120000	-4.8837180000	-0.9703720000
H	26.3005820000	-6.3907510000	-1.7554150000
H	23.5587500000	-5.3084610000	-2.0578430000
H	24.3000260000	-6.8277450000	-2.8174380000
H	23.7550650000	-6.3017400000	0.0472440000
H	24.3928440000	-7.8407200000	-0.7648380000
H	21.7097880000	-6.6130450000	-1.0494770000
H	22.3533530000	-8.2093440000	-1.7367580000
H	22.3409360000	-9.1334910000	0.5091850000
H	20.7035240000	-8.4345290000	1.9723390000
H	20.6243810000	-5.8775550000	0.3742630000
H	19.9019200000	-6.2124360000	2.0477640000
H	18.9358860000	-7.0950520000	-0.6117380000
H	18.2263230000	-7.4802800000	1.0564410000
H	18.3509800000	-4.8824260000	-0.3668220000
H	17.6913770000	-5.2314950000	1.3295790000
H	16.6625220000	-6.1488570000	-1.2935870000

1	H	16.0128410000	-6.5340090000	0.3988290000
2	H	16.0098930000	-3.9643960000	-1.0772020000
3	H	15.4524660000	-4.2795860000	0.6619440000
4	H	14.3129900000	-5.3214960000	-1.8673610000
5	H	13.7559140000	-5.6378790000	-0.1283190000
6	H	13.5636850000	-3.1915680000	-1.7816880000
7	H	13.2277290000	-3.3318280000	0.0354970000
8	H	12.2291050000	-5.7547940000	1.8106370000
9	H	11.6697980000	-6.7162510000	3.2930260000
10	H	9.3173520000	-4.4668810000	-0.2925280000
11	H	9.9967680000	-6.0561760000	-0.9612850000
12	H	10.3540320000	-6.7862650000	1.2691440000
13	H	7.5772740000	-5.7694940000	0.7885190000
14	H	8.0705710000	-7.0873400000	1.9946030000
15	H	4.3639280000	-10.0657930000	-1.8813950000
16	H	5.5332960000	-9.2096120000	-1.1289340000
17	H	3.4305200000	-9.1620740000	0.5941460000
18	H	3.6944970000	-10.9007730000	0.0093880000
19	H	5.1954540000	-9.2850060000	1.8617770000
20	H	5.3647240000	-11.0703280000	1.3941530000
21	H	5.2047520000	-7.5017840000	1.1861490000
22	H	8.9787310000	15.1946560000	-4.0369390000
23	H	7.3706220000	14.9304660000	-3.2477290000
24	H	7.4942520000	15.9477470000	-4.7334090000
25	H	8.1814630000	13.9706130000	-6.0693140000
26	H	6.5757680000	13.7049970000	-5.2831240000
27	H	9.2957680000	12.6766970000	-4.2325360000
28	H	7.6896540000	12.4052970000	-3.4467400000
29	H	8.5019650000	11.4662960000	-6.2770120000
30	H	6.8972010000	11.1841840000	-5.4911200000

1	H	9.6282770000	10.2030130000	-4.4352310000
2	H	8.0256500000	9.9035960000	-3.6531860000
3	H	8.8637420000	8.9777370000	-6.4802410000
4	H	7.2659700000	8.6538680000	-5.6956100000
5	H	10.0238760000	7.7650310000	-4.6300970000
6	H	8.4311720000	7.4093410000	-3.8526250000
7	H	26.2098490000	6.7309560000	1.7346020000
8	H	27.2550910000	7.0714180000	0.3033060000
9	H	25.8761380000	8.1707860000	0.6876510000
10	H	25.6820610000	5.2147990000	-0.1851620000
11	H	25.3491160000	6.6507540000	-1.2303680000
12	H	23.8235040000	5.9046990000	1.3557590000
13	H	23.4893420000	7.3414830000	0.3083980000
14	H	23.3261290000	4.3840630000	-0.5705540000
15	H	22.9912220000	5.8192020000	-1.6186280000
16	H	21.4628360000	5.0683660000	0.9615530000
17	H	21.1257490000	6.5020000000	-0.0890890000
18	H	20.9946540000	3.5429150000	-0.9673630000
19	H	20.6557450000	4.9741280000	-2.0192800000
20	H	19.1079960000	4.2080010000	0.5447020000
21	H	18.7666470000	5.6369860000	-0.5105630000
22	H	21.7948570000	13.6021760000	4.2372270000
23	H	20.8098860000	13.7318510000	2.7303450000
24	H	20.0211730000	13.9137520000	4.3497370000
25	H	20.5819980000	11.5351890000	4.8900570000
26	H	21.3698250000	11.3499190000	3.2736720000
27	H	18.3991460000	12.0468310000	3.7671110000
28	H	19.1840290000	11.8541160000	2.1493550000
29	H	18.9583510000	9.6707560000	4.3255480000
30	H	19.7365330000	9.4673890000	2.7046730000

1	H	16.7883050000	10.2281770000	3.2141380000
2	H	17.5555580000	10.0042950000	1.5924400000
3	H	17.2910280000	7.8459760000	3.7893330000
4	H	18.0398610000	7.6022470000	2.1596220000
5	H	15.1317930000	8.4842050000	2.7045850000
6	H	15.8614440000	8.2062910000	1.0740920000
7	H	-17.6145330000	13.7826100000	-0.5456500000
8	H	-19.3533490000	13.7749980000	-1.0295070000
9	H	-18.0910230000	13.2686730000	-2.2154030000
10	H	-18.8374240000	11.8140130000	0.4060330000
11	H	-19.3118250000	11.3035050000	-1.2615720000
12	H	-16.4160620000	11.5531480000	-0.2013570000
13	H	-16.8912440000	11.0482350000	-1.8719120000
14	H	-17.6554160000	9.5856680000	0.7427860000
15	H	-18.1331480000	9.0894340000	-0.9297310000
16	H	-15.2386330000	9.3303480000	0.1282000000
17	H	-15.7180980000	8.8504500000	-1.5482400000
18	H	-16.4744460000	7.3442080000	1.0441770000
19	H	-16.9623080000	6.8820720000	-0.6352590000
20	H	-14.0610010000	7.0933680000	0.4098750000
21	H	-14.5528480000	6.6595450000	-1.2753730000
22	H	-1.2997440000	6.2904970000	10.8453290000
23	H	-0.5274670000	7.8046880000	10.2208940000
24	H	-2.0378920000	7.8978370000	11.2039790000
25	H	-3.3103330000	6.7301150000	9.4186540000
26	H	-2.5389980000	8.2413530000	8.7958340000
27	H	-1.4242890000	5.3748530000	8.4681350000
28	H	-0.6519100000	6.8872350000	7.8459300000
29	H	-3.4428390000	5.8226580000	7.0498510000
30	H	-2.6698730000	7.3357490000	6.4291000000

1	H	-1.5511040000	4.4724460000	6.1143280000
2	H	-0.7769690000	5.9856880000	5.4966090000
3	H	-3.5601070000	4.9158730000	4.6804210000
4	H	-2.7852890000	6.4307130000	4.0657330000
5	H	-1.6556180000	3.5706390000	3.7651660000
6	H	-0.8787920000	5.0852700000	3.1544610000
7	H	18.6869190000	2.6753340000	-1.3896140000
8	H	18.3430590000	4.1009070000	-2.4475940000
9	H	15.5318660000	6.0913280000	3.3043530000
10	H	16.2316640000	5.7879890000	1.6625230000
11	H	13.3903250000	6.8430490000	2.2553550000
12	H	14.0780210000	6.5134430000	0.6231110000
13	H	13.6730690000	4.4634990000	2.8979380000
14	H	14.2954180000	4.0423900000	1.2804330000
15	H	10.7465280000	3.2948170000	1.7926060000
16	H	11.5775210000	5.2934140000	1.9874810000
17	H	16.7692610000	3.3141240000	0.0920920000
18	H	16.4435740000	4.7425980000	-0.9691750000
19	H	16.4364340000	1.7826090000	-1.8552010000
20	H	16.0419670000	3.1859320000	-2.9147010000
21	H	13.9858450000	1.9787190000	-3.1995550000
22	H	14.4936160000	2.2766230000	-0.4041930000
23	H	10.7536270000	4.9487460000	-0.1609290000
24	H	12.2090610000	5.8663460000	-0.4468170000
25	H	14.2468010000	4.7163080000	-0.8258240000
26	H	13.7687290000	4.4498300000	-2.4956670000
27	H	9.3155890000	6.5063600000	-6.6730880000
28	H	7.7339660000	6.1084500000	-5.8885590000
29	H	10.5305380000	5.3806330000	-4.8124340000
30	H	8.9557750000	4.9695220000	-4.0397920000

1	H	8.3839640000	3.5494520000	-6.1083400000
2	H	9.9196230000	4.1342000000	-6.8391310000
3	H	11.1884310000	2.0086820000	-6.8488980000
4	H	11.2254070000	3.0620870000	-4.9852640000
5	H	10.2134980000	1.1219750000	-3.7396540000
6	H	8.6744550000	1.4336990000	-4.5209830000
7	H	-3.6474970000	4.0045470000	2.3043440000
8	H	-2.8699400000	5.5222780000	1.6988500000
9	H	-1.7463240000	2.6904280000	1.4512450000
10	H	-0.9449540000	4.1828870000	0.8146490000
11	H	-3.6840680000	3.0515630000	-0.0802090000
12	H	-2.9602550000	4.6174600000	-0.5801370000
13	H	-2.6232440000	3.9983730000	-2.9787650000
14	H	-1.0508900000	3.4750330000	-1.6443690000
15	H	-2.6936100000	0.9381720000	-1.0849020000
16	H	-1.5383810000	0.9966240000	-2.4128820000
17	H	11.9354610000	3.0632420000	-2.9959580000
18	H	11.1666820000	4.6371950000	-2.6060380000
19	H	10.4055900000	1.8126180000	-1.5345820000
20	H	9.6907310000	3.3341730000	-0.9761600000
21	H	7.7959970000	1.4472790000	-2.1123880000
22	H	7.6144790000	3.1487250000	-1.5445420000
23	H	-2.9929440000	-3.4694090000	0.6848290000
24	H	-4.7230380000	-3.1711450000	1.0768020000
25	H	-3.6173760000	-3.8492130000	-1.7234880000
26	H	-5.3297930000	-3.4705350000	-1.3223260000
27	H	-6.4728840000	-5.1368250000	0.0580660000
28	H	-6.3771980000	-8.0201910000	-1.4193250000
29	H	-6.3968670000	-4.7535920000	-3.3342320000
30	H	-2.9782430000	-5.9849080000	2.1281870000

1	H	-3.1286520000	-8.4152850000	-0.7241220000
2	H	-13.1260480000	0.5117120000	1.0638450000
3	H	-15.2898970000	5.0775270000	1.2774040000
4	H	-15.7972860000	4.6730010000	-0.4109210000
5	H	-12.8978060000	4.8562710000	0.6072130000
6	H	-13.3978810000	4.4729860000	-1.0869580000
7	H	-14.1027440000	2.7655430000	1.4010090000
8	H	-14.6741830000	2.4948540000	-0.2859570000
9	H	-12.3413510000	2.2440490000	-1.0817830000
10	H	-11.4977410000	2.0881060000	1.9039830000
11	H	-11.2308820000	3.4928150000	0.8318830000
12	H	-3.0174480000	-1.6101430000	-0.9300210000
13	H	-4.6389550000	-1.1688850000	-0.5538660000
14	H	-4.6459800000	-1.5222400000	2.6041030000
15	H	-3.7789360000	-0.0528830000	2.9948160000
16	H	-5.8757100000	0.8017660000	2.6418770000
17	H	-4.9369070000	1.1891790000	1.1503990000
18	H	-8.6301040000	-1.2012030000	-0.3458530000
19	H	-8.6422940000	0.2573480000	-1.3888280000
20	H	-10.0763020000	-0.0385090000	1.3372120000
21	H	-10.7859190000	-0.0752100000	-0.3277400000
22	H	-8.9762490000	2.0473540000	2.1448810000
23	H	-8.9605060000	3.5092730000	1.0852250000
24	H	-6.7735820000	2.4088030000	1.0605950000
25	H	-7.5320620000	2.3854550000	-0.5599150000
26	H	1.4383800000	0.3872870000	-0.5206650000
27	H	0.5989710000	0.0489040000	-2.0719000000
28	H	1.3251480000	2.8519470000	-0.9746390000
29	H	0.5462180000	2.4636290000	-2.5695860000
30	H	6.6926740000	2.1804350000	-4.3153710000

H	6.8466940000	3.8821210000	-3.7684280000
H	3.4355340000	0.4641490000	-1.2194180000
H	3.7186610000	2.1672870000	-0.6723680000
H	5.7300450000	1.0873430000	-1.5339530000
H	4.9487400000	0.7129650000	-3.1185270000
H	4.2477860000	2.5431870000	-4.5974530000
H	4.5481030000	4.2544270000	-4.1051890000
H	3.0095350000	4.0554070000	-2.2091430000
H	2.1939400000	3.6334830000	-3.7643560000
H	-0.4170380000	1.6108730000	0.2643160000
H	-0.0549310000	-0.7958930000	0.6523900000
H	-1.0949990000	-1.1175240000	-0.7078720000

6.6. Branched amine core based bone-targeting ionizable lipids ‘Type 2’

Type2-B1-C12

P	-7.6828890000	3.9298120000	3.0715830000
P	15.7686890000	1.4991680000	-1.7988060000
P	0.5243380000	4.2259660000	4.8666420000
P	0.7366620000	4.5424250000	1.8827080000
O	-11.9333860000	2.3874500000	-1.5412020000
O	-12.4720500000	2.0019560000	0.6393180000
O	-13.1093950000	-0.6388830000	3.6762060000
O	-11.5346250000	0.8080530000	2.9907610000
O	-6.4628390000	2.7309630000	3.3720710000
O	-9.1705290000	3.5112670000	2.7250450000
O	-7.3390840000	4.8302900000	4.3871330000
O	-6.9053960000	4.6549660000	2.0313580000
O	6.9715930000	7.0626800000	0.8300660000
O	10.3116100000	5.2600260000	-2.4199370000
O	15.7701300000	1.2944180000	-0.0725190000

1	O	16.0980220000	0.1600200000	-2.3576050000
2	O	14.4191450000	2.1586680000	-2.3682330000
3	O	17.0676330000	2.4912960000	-1.8314930000
4	O	19.4322620000	3.5074230000	-1.1150860000
5	O	19.9459800000	1.3227000000	-0.6990090000
6	O	18.8797370000	4.6085510000	-4.4789320000
7	O	18.5642710000	5.5625110000	-6.4951090000
8	O	9.4864660000	4.7839330000	-0.3761290000
9	O	10.1004030000	-5.1305350000	0.2540800000
10	O	11.8050200000	-4.1644820000	1.3459810000
11	O	-3.9773090000	-2.8740230000	-5.0591000000
12	O	3.0943470000	-4.0774500000	3.1710160000
13	O	-3.8988780000	-9.9833370000	0.5147040000
14	O	0.5753220000	2.1860180000	3.1199620000
15	O	0.8176010000	3.3672490000	6.1275440000
16	O	1.1997040000	5.7814740000	5.2447820000
17	O	-1.2004730000	4.4269290000	4.8892820000
18	O	1.1399580000	3.9600110000	0.5008100000
19	O	-0.9721830000	4.8102610000	1.7280800000
20	O	1.4635460000	6.1192270000	1.9207630000
21	O	0.0461890000	-0.6710710000	-4.4756400000
22	O	-7.0084490000	-8.6876360000	-0.5088710000
23	O	2.6924640000	-1.3391560000	4.2891910000
24	O	7.3711420000	-0.2533710000	4.2536270000
25	N	-5.1834700000	4.7803330000	3.2416400000
26	N	11.2456890000	3.5806190000	-1.2363120000
27	N	-5.0553400000	-6.4504430000	0.3070420000
28	N	-2.2749960000	-3.8201880000	0.6781280000
29	N	-1.7525020000	-2.6118240000	-2.9396390000
30	N	0.7810520000	-2.2654860000	2.3753600000

1	N	3.4340030000	0.2564900000	2.8825300000
2	N	7.7768940000	-2.1942860000	3.1894760000
3	N	9.6135040000	-3.2786240000	1.4306040000
4	C	-24.7828050000	2.2782910000	-5.1220450000
5	C	-24.7969620000	3.1867380000	-3.8864590000
6	C	-24.4603690000	2.4192870000	-2.5978560000
7	C	-24.5046780000	3.3397370000	-1.3685160000
8	C	-24.1556330000	2.6053780000	-0.0646580000
9	C	-24.3091360000	3.5250410000	1.1574520000
10	C	-23.8773160000	2.8382810000	2.4623480000
11	C	-23.9449770000	3.7994240000	3.6583670000
12	C	-23.5905160000	3.0887520000	4.9397240000
13	C	-22.3749370000	3.0030220000	5.5035920000
14	C	-21.0815110000	3.5883380000	4.9896350000
15	C	-20.0700590000	2.4719690000	4.6825170000
16	C	-18.6754860000	3.0122620000	4.3223930000
17	C	-17.6729080000	1.8683780000	4.0946530000
18	C	-16.2365240000	2.3663930000	3.8622380000
19	C	-15.2469540000	1.1934030000	3.7483110000
20	C	-28.9277070000	6.0693850000	-6.6242860000
21	C	-27.4786630000	6.4167860000	-6.2587340000
22	C	-27.2824290000	6.5205450000	-4.7380260000
23	C	-25.8313060000	6.8676860000	-4.3691960000
24	C	-25.6241460000	6.9259440000	-2.8470900000
25	C	-24.1537140000	7.1765720000	-2.4770720000
26	C	-23.9396350000	7.2139330000	-0.9552040000
27	C	-22.4556980000	7.3750030000	-0.5915770000
28	C	-22.2544260000	7.3057580000	0.9018810000
29	C	-21.6254070000	6.3325080000	1.5807010000
30	C	-20.9854930000	5.0968530000	0.9969250000

C	-19.4749010000	5.3027140000	0.8282500000
C	-18.7821340000	4.0484310000	0.2721580000
C	-17.2645860000	4.2571940000	0.1596450000
C	-16.5378570000	3.0096070000	-0.3672020000
C	-15.0161700000	3.2203010000	-0.3765540000
C	-14.2517510000	1.9930100000	-0.8967660000
C	-12.7547620000	2.1510580000	-0.6847060000
C	-13.7929430000	1.6581940000	3.5454820000
C	-12.8020520000	0.5046870000	3.4079910000
C	-11.1465560000	2.1678730000	1.0603730000
C	-11.0919120000	2.0742820000	2.5872120000
C	-9.6514130000	2.2401630000	3.0823150000
C	-5.0204080000	4.4910840000	4.7292540000
C	-6.4220830000	4.4666940000	5.3760040000
C	6.1906440000	7.1741300000	2.0007410000
C	7.8477440000	5.9630430000	0.8785480000
C	8.6443680000	5.9068510000	-0.4166360000
C	13.7480510000	21.5183220000	-6.1773200000
C	14.2036270000	20.2335440000	-6.8817320000
C	14.4531130000	19.0915180000	-5.8837380000
C	14.9086580000	17.8026870000	-6.5870840000
C	18.4583430000	-2.2553790000	15.5533980000
C	18.7410520000	-2.6120430000	14.0879380000
C	18.5160570000	-1.4127670000	13.1532510000
C	18.8008530000	-1.7672760000	11.6846900000
C	18.5807430000	-0.5678850000	10.7481790000
C	15.1600140000	16.6593600000	-5.5902490000
C	13.4515050000	2.6094200000	-1.4557120000
C	12.2672250000	3.2044350000	-2.2157280000
C	18.1039920000	2.2913110000	-2.7575250000

1	C	19.2050590000	3.3232860000	-2.4886790000
2	C	18.8515040000	4.6901110000	-3.0787890000
3	C	19.5968360000	2.4157170000	-0.3139700000
4	C	19.2164680000	2.7569710000	1.1182120000
5	C	18.5161070000	5.6493950000	-5.2855360000
6	C	18.0500590000	6.9167590000	-4.5747030000
7	C	17.7564160000	8.0840140000	-5.5345570000
8	C	17.3060050000	9.3530600000	-4.7913550000
9	C	17.0300770000	10.5112230000	-5.7643650000
10	C	16.5818550000	11.7893660000	-5.0372030000
11	C	16.3182300000	12.9410750000	-6.0213420000
12	C	15.8678450000	14.2249890000	-5.3057760000
13	C	15.6126090000	15.3717080000	-6.2978520000
14	C	19.3363440000	1.5648350000	2.0805050000
15	C	18.9684510000	1.9415310000	3.5250920000
16	C	19.1234570000	0.7455450000	4.4789150000
17	C	18.7893370000	1.1124140000	5.9339810000
18	C	18.9752950000	-0.0857440000	6.8796290000
19	C	18.6664390000	0.2745790000	8.3417960000
20	C	18.8737650000	-0.9245170000	9.2817370000
21	C	10.3410650000	4.5838580000	-1.4186530000
22	C	10.4908710000	-4.2318800000	0.9864930000
23	C	12.6157310000	-6.4415950000	1.3943840000
24	C	13.0599390000	-4.9118670000	-0.5756490000
25	C	14.1266890000	-4.4622090000	1.6425160000
26	C	12.8573160000	-4.9846120000	0.9517000000
27	C	-18.3648550000	-0.9900560000	0.4218670000
28	C	-17.5776880000	-2.1404080000	-0.1986840000
29	C	-16.1418130000	-2.1824290000	0.3374290000
30	C	-15.3588570000	-3.3430890000	-0.2865280000

1	C	-13.9226880000	-3.3946430000	0.2492140000
2	C	-13.1534680000	-4.5659760000	-0.3712170000
3	C	-11.7178670000	-4.6390540000	0.1637330000
4	C	6.7562540000	-5.9190840000	6.2666440000
5	C	5.2451210000	-5.9435530000	6.4791660000
6	C	4.5399580000	-6.6943910000	5.3445660000
7	C	3.0194600000	-6.7003150000	5.5466750000
8	C	2.3207230000	-7.4198180000	4.3880850000
9	C	0.7964630000	-7.3685240000	4.5318430000
10	C	0.1144860000	-7.9976940000	3.3106400000
11	C	8.4083960000	-11.3813350000	-6.4648610000
12	C	8.3462460000	-9.8662910000	-6.2911100000
13	C	6.9271950000	-9.4101600000	-5.9366920000
14	C	6.8605870000	-7.8867330000	-5.7643640000
15	C	5.4382590000	-7.4493040000	-5.4032550000
16	C	5.3319520000	-5.9261780000	-5.2379000000
17	C	3.8913700000	-5.5417860000	-4.8912910000
18	C	5.1328910000	-12.2710460000	-9.2155410000
19	C	3.7109710000	-11.7634630000	-9.4382880000
20	C	3.4901940000	-10.4233610000	-8.7304300000
21	C	2.0531640000	-9.9218170000	-8.9246390000
22	C	1.8423270000	-8.6070260000	-8.1706720000
23	C	0.3880310000	-8.1215090000	-8.2561200000
24	C	0.2270410000	-6.8486820000	-7.4230000000
25	C	-1.2319260000	-6.3734090000	-7.3215580000
26	C	-1.2836640000	-5.1571280000	-6.3930270000
27	C	-2.7120110000	-4.6470020000	-6.0946870000
28	C	-2.6871020000	-3.4197680000	-5.1493590000
29	C	-0.0795360000	-12.9094020000	-6.7842300000
30	C	1.0097350000	-12.2948050000	-5.9095770000

1	C	0.5025280000	-11.0332300000	-5.2028080000
2	C	1.5965750000	-10.4120570000	-4.3273290000
3	C	1.0726070000	-9.1684600000	-3.5999540000
4	C	2.1491430000	-8.5654200000	-2.6904460000
5	C	1.5971620000	-7.3633220000	-1.9160710000
6	C	2.6423050000	-6.7886580000	-0.9483170000
7	C	2.0179250000	-5.6889380000	-0.0831380000
8	C	2.9951550000	-5.1713470000	0.9912490000
9	C	2.2805840000	-4.2836190000	2.0452330000
10	C	-1.4109490000	-7.9199650000	3.4305870000
11	C	-10.9752350000	-5.8294310000	-0.4530110000
12	C	3.6938740000	-4.0254410000	-4.7440680000
13	C	-2.0932940000	-8.4635470000	2.1695230000
14	C	-3.6259050000	-8.3743440000	2.3011830000
15	C	-4.3860280000	-8.7647120000	1.0155130000
16	C	-9.5395700000	-5.9395120000	0.0776080000
17	C	3.5176880000	2.7126030000	2.2443090000
18	C	2.7328770000	3.2598000000	3.4549460000
19	C	1.1989900000	3.4237070000	3.3098750000
20	C	2.2185400000	-3.7464410000	-4.4403530000
21	C	1.8711210000	-2.2428870000	-4.3641520000
22	C	0.3414510000	-2.0283830000	-4.2751020000
23	C	-2.1929310000	-3.7982300000	-3.7263680000
24	C	-0.2608820000	-2.4915060000	-2.9292110000
25	C	-8.8434680000	-7.1642160000	-0.5372050000
26	C	-7.4187510000	-7.4431950000	0.0002870000
27	C	-4.3210820000	-7.6867880000	-0.0981680000
28	C	-6.3944080000	-6.3233460000	-0.3442200000
29	C	1.8040190000	-2.9152670000	1.4887630000
30	C	-1.7081090000	-3.8203910000	-0.7049090000

1	C	-2.2900770000	-2.6647650000	-1.5521040000
2	C	-1.1628560000	-3.7462160000	1.6631250000
3	C	-3.0499450000	-5.0584810000	0.9346520000
4	C	-0.5666710000	-2.3209080000	1.7317830000
5	C	-4.2771770000	-5.2172810000	0.0091590000
6	C	1.0934450000	-0.8376080000	2.6566630000
7	C	2.4633770000	-0.6373570000	3.3129620000
8	C	3.2162220000	1.2525350000	1.8166110000
9	C	4.7712600000	0.1957900000	3.4898160000
10	C	5.4968210000	-1.1030580000	3.0948780000
11	C	6.9409560000	-1.1653010000	3.5590040000
12	C	7.3516630000	-3.3059590000	2.3378700000
13	C	8.1996770000	-3.2932800000	1.0620660000
14	C	10.0412120000	-2.1913460000	2.3063400000
15	C	9.1862080000	-2.2112690000	3.5790220000
16	H	-23.8103050000	1.8469570000	-5.2364830000
17	H	-25.5063550000	1.4994570000	-5.0004220000
18	H	-25.0218760000	2.8546600000	-5.9912650000
19	H	-24.0069260000	3.8944820000	-4.0273190000
20	H	-25.8058590000	3.5259030000	-3.7769390000
21	H	-23.4450480000	2.0961900000	-2.6959930000
22	H	-25.2403330000	1.6992190000	-2.4634740000
23	H	-23.7334790000	4.0669040000	-1.5147340000
24	H	-25.5230750000	3.6525750000	-1.2690140000
25	H	-23.1164300000	2.3582650000	-0.1270440000
26	H	-24.8841000000	1.8298660000	0.0485590000
27	H	-23.6272040000	4.3358690000	1.0076940000
28	H	-25.3569880000	3.7183500000	1.2551210000
29	H	-22.8454650000	2.5828140000	2.3402070000
30	H	-24.5994620000	2.0717550000	2.6516770000

1	H	-23.1849500000	4.5356270000	3.4994200000
2	H	-24.9683320000	4.0981320000	3.7501110000
3	H	-24.3921470000	2.6012270000	5.4541240000
4	H	-22.3161550000	2.4575210000	6.4222180000
5	H	-20.6671170000	4.1632470000	5.7912940000
6	H	-21.3093700000	4.0587440000	4.0559870000
7	H	-20.4317850000	1.9781560000	3.8049040000
8	H	-19.9488530000	1.9236940000	5.5933420000
9	H	-18.7796340000	3.5135580000	3.3828430000
10	H	-18.3296320000	3.5501060000	5.1803030000
11	H	-17.9709780000	1.3911800000	3.1845230000
12	H	-17.6482050000	1.3110900000	5.0077350000
13	H	-16.2369280000	2.8540620000	2.9098310000
14	H	-15.9599100000	2.9098660000	4.7414550000
15	H	-15.5158010000	0.6644560000	2.8578990000
16	H	-15.2691130000	0.6941930000	4.6944610000
17	H	-28.9475290000	5.1468460000	-7.1659780000
18	H	-29.3389400000	6.8484810000	-7.2315730000
19	H	-29.5072040000	5.9705530000	-5.7302420000
20	H	-26.8813020000	5.5901950000	-6.5824750000
21	H	-27.2964560000	7.3950520000	-6.6520490000
22	H	-27.4655260000	5.5423330000	-4.3449910000
23	H	-27.8796220000	7.3474260000	-4.4147180000
24	H	-25.2301930000	6.0560040000	-4.7223760000
25	H	-25.6588020000	7.8590240000	-4.7330520000
26	H	-25.8648440000	5.9521610000	-2.4746520000
27	H	-26.1692200000	7.7800670000	-2.5031890000
28	H	-23.6067750000	6.3295120000	-2.8351890000
29	H	-23.9179030000	8.1565030000	-2.8362750000
30	H	-24.2362180000	6.2540810000	-0.5869350000

1	H	-24.4319050000	8.0969300000	-0.6046500000
2	H	-21.9487210000	6.5283890000	-1.0052430000
3	H	-22.1763360000	8.3640050000	-0.8894570000
4	H	-22.6532020000	8.1138880000	1.4787710000
5	H	-21.5726360000	6.4446730000	2.6434970000
6	H	-21.3891290000	4.9803470000	0.0128500000
7	H	-21.1082240000	4.3187710000	1.7210970000
8	H	-19.3576850000	6.0636360000	0.0851770000
9	H	-19.0813040000	5.4506150000	1.8121740000
10	H	-19.1446410000	3.9203210000	-0.7263800000
11	H	-18.9281270000	3.2755700000	0.9976040000
12	H	-17.1230210000	5.0180610000	-0.5792320000
13	H	-16.9103510000	4.4083520000	1.1579280000
14	H	-16.8340780000	2.8954780000	-1.3890280000
15	H	-16.7309080000	2.2269380000	0.3364030000
16	H	-14.8297720000	4.0055430000	-1.0790860000
17	H	-14.7267580000	3.3381540000	0.6467990000
18	H	-14.4064160000	1.9662780000	-1.9551920000
19	H	-14.5577980000	1.1662980000	-0.2903150000
20	H	-13.7770330000	2.1726500000	2.6074080000
21	H	-13.5212720000	2.1735690000	4.4429690000
22	H	-10.5929420000	1.3357530000	0.6782890000
23	H	-10.8606600000	3.1655640000	0.8000330000
24	H	-11.7107680000	2.8468500000	2.9934820000
25	H	-9.0586770000	1.5258960000	2.5499750000
26	H	-9.6883410000	2.2142140000	4.1513620000
27	H	-4.7289130000	5.6424960000	3.0179160000
28	H	-6.1561940000	4.8490670000	3.0200920000
29	H	-4.6191800000	3.5027840000	4.8139820000
30	H	-4.4940680000	5.3203800000	5.1536770000

1	H	-6.6302870000	3.4520870000	5.6445650000
2	H	-6.4404490000	5.2324330000	6.1231360000
3	H	-6.6821740000	2.2441610000	4.1698910000
4	H	6.8329390000	7.2980240000	2.8475040000
5	H	5.6046110000	6.2874520000	2.1243030000
6	H	5.5424430000	8.0213900000	1.9177570000
7	H	8.5467590000	6.1572040000	1.6650460000
8	H	7.2401580000	5.0827750000	0.9080150000
9	H	9.2760240000	6.7703680000	-0.4323620000
10	H	7.9428810000	5.7426470000	-1.2077450000
11	H	16.5617600000	1.6929990000	0.2963380000
12	H	14.5031330000	21.8341650000	-5.4881210000
13	H	12.8374740000	21.3309500000	-5.6475650000
14	H	13.5858570000	22.2857770000	-6.9050610000
15	H	15.1512380000	20.4553870000	-7.3263880000
16	H	13.3845400000	19.9216470000	-7.4955040000
17	H	15.2725720000	19.4035510000	-5.2705310000
18	H	13.5055100000	18.8706750000	-5.4385680000
19	H	15.8557240000	18.0241600000	-7.0330840000
20	H	14.0887570000	17.4904990000	-7.1996210000
21	H	19.3439870000	-2.4065240000	16.1345090000
22	H	17.6749780000	-2.8806520000	15.9279250000
23	H	18.1589340000	-1.2302590000	15.6196140000
24	H	19.7840590000	-2.8441860000	14.0318380000
25	H	18.0138070000	-3.3470750000	13.8127240000
26	H	19.2422650000	-0.6772500000	13.4299010000
27	H	17.4727550000	-1.1817580000	13.2085300000
28	H	19.8434850000	-2.0015360000	11.6304630000
29	H	18.0726300000	-2.5006010000	11.4075170000
30	H	19.3054720000	0.1678250000	11.0281740000

1	H	17.5371680000	-0.3367680000	10.7975500000
2	H	15.9814460000	16.9699390000	-4.9789460000
3	H	14.2135420000	16.4385170000	-5.1426790000
4	H	13.0854400000	1.7448990000	-0.9423890000
5	H	13.8975260000	3.4103530000	-0.9039240000
6	H	11.8537640000	2.4217220000	-2.8168190000
7	H	12.6101530000	4.1077230000	-2.6754790000
8	H	10.6758920000	2.7618040000	-1.1665000000
9	H	18.5226900000	1.3278190000	-2.5543620000
10	H	17.7034080000	2.5071700000	-3.7259450000
11	H	20.0848520000	2.9310630000	-2.9545390000
12	H	17.8389190000	4.8954100000	-2.8005380000
13	H	19.6322020000	5.3631250000	-2.7916240000
14	H	19.9359190000	3.4761180000	1.4500520000
15	H	18.1775100000	3.0116610000	1.0937110000
16	H	18.8737660000	7.2340180000	-3.9699280000
17	H	17.1145820000	6.6699770000	-4.1176690000
18	H	18.6879220000	8.3271420000	-6.0015540000
19	H	16.9230310000	7.7766180000	-6.1311110000
20	H	18.1351820000	9.6583830000	-4.1879130000
21	H	16.3688930000	9.1161380000	-4.3324550000
22	H	17.9676050000	10.7434600000	-6.2248090000
23	H	16.2002090000	10.2039010000	-6.3658400000
24	H	17.4087200000	12.0956590000	-4.4310850000
25	H	15.6404900000	11.5616410000	-4.5823700000
26	H	17.2604160000	13.1672750000	-6.4752370000
27	H	15.4926170000	12.6322100000	-6.6278610000
28	H	16.6914140000	14.5333750000	-4.6962400000
29	H	14.9231270000	14.0019040000	-4.8556190000
30	H	16.5579880000	15.5941420000	-6.7469420000

1	H	14.7902610000	15.0613250000	-6.9080210000
2	H	20.3724260000	1.2980570000	2.0967840000
3	H	18.6050360000	0.8510940000	1.7632340000
4	H	19.6864150000	2.6706360000	3.8378760000
5	H	17.9267870000	2.1859810000	3.5161310000
6	H	20.1619250000	0.4879530000	4.4674990000
7	H	18.3905940000	0.0267360000	4.1770450000
8	H	19.5100630000	1.8449410000	6.2320720000
9	H	17.7462020000	1.3500050000	5.9518340000
10	H	20.0157820000	-0.3331360000	6.8467040000
11	H	18.2435750000	-0.8107490000	6.5900560000
12	H	19.3895610000	1.0091620000	8.6288180000
13	H	17.6228740000	0.5079850000	8.3791420000
14	H	19.9155540000	-1.1640790000	9.2350000000
15	H	18.1437990000	-1.6544510000	9.0002270000
16	H	11.7196710000	-6.8751950000	0.9065450000
17	H	13.4848870000	-7.0794590000	1.1238360000
18	H	12.4712930000	-6.4910350000	2.4949060000
19	H	12.1741930000	-5.2928260000	-1.1233460000
20	H	13.9349520000	-5.5241770000	-0.8832770000
21	H	13.2374360000	-3.8612480000	-0.8914780000
22	H	15.0117750000	-5.0753440000	1.3655460000
23	H	14.3226440000	-3.4085430000	1.3475780000
24	H	14.0076890000	-4.4937900000	2.7470860000
25	H	-18.4167350000	-1.1090490000	1.5251400000
26	H	-17.8831380000	-0.0184430000	0.1811960000
27	H	-19.3984060000	-0.9850630000	0.0160190000
28	H	-18.0936290000	-3.0963590000	0.0390980000
29	H	-17.5608170000	-2.0094320000	-1.3027580000
30	H	-16.1666380000	-2.3091510000	1.4423910000

1	H	-15.6312500000	-1.2234350000	0.0980800000
2	H	-15.8733100000	-4.3000640000	-0.0468390000
3	H	-15.3342350000	-3.2177600000	-1.3916610000
4	H	-13.9479620000	-3.5180510000	1.3545070000
5	H	-13.4028290000	-2.4416330000	0.0066070000
6	H	-13.6812370000	-5.5149770000	-0.1285810000
7	H	-13.1276980000	-4.4447250000	-1.4768440000
8	H	-11.7439150000	-4.7576650000	1.2694730000
9	H	-11.1805540000	-3.6971430000	-0.0838810000
10	H	7.0038040000	-5.4060500000	5.3125770000
11	H	7.1595260000	-6.9537920000	6.2404630000
12	H	7.2406480000	-5.3692450000	7.1008400000
13	H	4.8744950000	-4.8959090000	6.5202590000
14	H	5.0240740000	-6.4392200000	7.4495870000
15	H	4.7777440000	-6.1948500000	4.3788720000
16	H	4.9116150000	-7.7421660000	5.3056130000
17	H	2.6594480000	-5.6483970000	5.5900650000
18	H	2.7673520000	-7.2049190000	6.5051240000
19	H	2.6020470000	-6.9177220000	3.4436340000
20	H	2.6585940000	-8.4783980000	4.3397930000
21	H	0.4756270000	-6.3056990000	4.6087620000
22	H	0.4909260000	-7.9037560000	5.4575020000
23	H	0.4334450000	-7.4435820000	2.4006980000
24	H	0.4257650000	-9.0605630000	3.2086960000
25	H	7.7332750000	-11.7038180000	-7.2857290000
26	H	8.1083100000	-11.8909130000	-5.5243450000
27	H	9.4458660000	-11.6851540000	-6.7180970000
28	H	8.6691690000	-9.3829010000	-7.2387350000
29	H	9.0471380000	-9.5686570000	-5.4808920000
30	H	6.2299960000	-9.7186260000	-6.7475250000

H	6.6117690000	-9.9011300000	-4.9893970000
H	7.1678290000	-7.3955110000	-6.7138260000
H	7.5573130000	-7.5735770000	-4.9556930000
H	4.7434510000	-7.7776950000	-6.2088070000
H	5.1398180000	-7.9427690000	-4.4526990000
H	5.6261640000	-5.4316260000	-6.1895430000
H	6.0135530000	-5.5856720000	-4.4279280000
H	3.2231180000	-5.9143000000	-5.7003850000
H	3.6038520000	-6.0359230000	-3.9401710000
H	-3.8745650000	-1.9457890000	-4.7209090000
H	5.8709810000	-11.5448980000	-9.6178780000
H	5.2677530000	-13.2425500000	-9.7359540000
H	5.3228680000	-12.4202290000	-8.1309350000
H	3.5339990000	-11.6441250000	-10.5292230000
H	2.9971670000	-12.5180120000	-9.0405340000
H	4.2036160000	-9.6692950000	-9.1303010000
H	3.6858660000	-10.5549490000	-7.6426640000
H	1.8434880000	-9.7715310000	-10.0061430000
H	1.3487380000	-10.6835780000	-8.5287150000
H	2.5247700000	-7.8291990000	-8.5790850000
H	2.0966790000	-8.7723370000	-7.0999030000
H	0.1012230000	-7.9257630000	-9.3121280000
H	-0.2790340000	-8.9100250000	-7.8458570000
H	0.8610040000	-6.0413300000	-7.8524710000
H	0.5893330000	-7.0665500000	-6.3936800000
H	-1.6340770000	-6.1146700000	-8.3248620000
H	-1.8466010000	-7.1900340000	-6.8828040000
H	-0.6851350000	-4.3348440000	-6.8436410000
H	-0.7902670000	-5.4719660000	-5.4576550000
H	-3.1727240000	-4.3435470000	-7.0603400000

H	-3.3313880000	-5.4555710000	-5.6488890000
H	-2.0379960000	-2.6813860000	-5.6554810000
H	4.0102620000	-3.8824880000	2.8479420000
H	0.3138670000	-13.8189820000	-7.2849820000
H	-0.9564610000	-13.1951610000	-6.1647910000
H	-0.4056770000	-12.1875650000	-7.5628040000
H	1.8845250000	-12.0429780000	-6.5456130000
H	1.3272560000	-13.0447980000	-5.1527100000
H	0.1751390000	-10.2903640000	-5.9601370000
H	-0.3701960000	-11.3003060000	-4.5664820000
H	2.4682970000	-10.1335240000	-4.9597270000
H	1.9259110000	-11.1618800000	-3.5743330000
H	0.7460590000	-8.4087950000	-4.3427760000
H	0.1961580000	-9.4595260000	-2.9792470000
H	3.0233640000	-8.2562870000	-3.2995430000
H	2.4787390000	-9.3396840000	-1.9627970000
H	1.2631420000	-6.5722580000	-2.6231480000
H	0.7168220000	-7.7071170000	-1.3279450000
H	3.5152080000	-6.3906980000	-1.5090930000
H	2.9900640000	-7.6065950000	-0.2790370000
H	1.6640490000	-4.8630070000	-0.7332710000
H	1.1406810000	-6.1370100000	0.4321250000
H	3.8565970000	-4.6425740000	0.5283210000
H	3.3930740000	-6.0578980000	1.5268720000
H	1.4198680000	-4.8832030000	2.3984620000
H	-1.7430170000	-8.4983170000	4.3206360000
H	-1.7089880000	-6.8557790000	3.5622700000
H	-11.5268380000	-6.7636750000	-0.2057190000
H	-10.9493340000	-5.7133400000	-1.5593400000
H	3.9758400000	-3.5267680000	-5.6971930000

1	H	4.3324160000	-3.6300260000	-3.9242450000
2	H	-1.7991260000	-9.5234210000	2.0070120000
3	H	-1.7304710000	-7.8891560000	1.2997910000
4	H	-3.9202600000	-9.0736480000	3.1144890000
5	H	-3.9465010000	-7.3561220000	2.6070590000
6	H	-4.2848640000	-10.7039650000	1.0785070000
7	H	-5.4535880000	-8.8842640000	1.2916810000
8	H	-9.5660910000	-6.0532790000	1.1835180000
9	H	-8.9952630000	-5.0075980000	-0.1788900000
10	H	3.3702050000	3.3766070000	1.3703460000
11	H	4.5909710000	2.8203610000	2.4883260000
12	H	3.1513310000	4.2677620000	3.6684870000
13	H	2.9526600000	2.6189880000	4.3363820000
14	H	0.6648210000	1.6867440000	3.9719630000
15	H	2.0926770000	5.5890840000	5.6274930000
16	H	-1.5632070000	3.5057910000	4.9223600000
17	H	-1.3475240000	3.9252360000	1.4897980000
18	H	2.4210950000	5.9633510000	1.7218380000
19	H	1.6190270000	-4.1985740000	-5.2620290000
20	H	1.9622500000	-4.2596460000	-3.4926930000
21	H	2.2142330000	-1.7783200000	-5.3151720000
22	H	2.3997870000	-1.7456200000	-3.5222490000
23	H	-0.8913520000	-0.6244070000	-4.7983100000
24	H	-0.0411800000	-2.6243840000	-5.1205240000
25	H	-1.3774440000	-4.5386010000	-3.7339720000
26	H	-3.0330130000	-4.3476390000	-3.2442670000
27	H	0.1728350000	-3.4705710000	-2.6598910000
28	H	0.0759080000	-1.7718380000	-2.1489200000
29	H	-6.9913410000	-8.6209470000	-1.5000610000
30	H	-9.4629920000	-8.0562840000	-0.2937020000

1	H	-8.8115770000	-7.0590090000	-1.6442970000
2	H	-7.4900030000	-7.5271630000	1.1063530000
3	H	-4.7326220000	-8.0883940000	-1.0471590000
4	H	-3.2597430000	-7.4994490000	-0.3546530000
5	H	-6.2860370000	-6.2449210000	-1.4500590000
6	H	-6.8422120000	-5.3744250000	0.0130090000
7	H	1.3968110000	-3.0412270000	0.4664300000
8	H	2.7054550000	-2.2831790000	1.3305950000
9	H	-1.8496320000	-4.7955840000	-1.2167710000
10	H	-0.6084180000	-3.7060730000	-0.6797580000
11	H	-3.3977950000	-2.7533170000	-1.5890190000
12	H	-2.0742380000	-1.6997630000	-1.0458170000
13	H	-0.4059920000	-4.4953180000	1.3690130000
14	H	-1.4883710000	-4.0188930000	2.6922220000
15	H	-2.3723020000	-5.9175480000	0.8282030000
16	H	-3.4303730000	-5.0453530000	1.9800530000
17	H	-0.4829040000	-1.8816330000	0.7142590000
18	H	-1.3084150000	-1.6895350000	2.2719850000
19	H	-3.9977160000	-5.2162340000	-1.0652200000
20	H	-4.9006810000	-4.3128240000	0.1884520000
21	H	3.8874790000	1.0065760000	0.9656200000
22	H	2.1995760000	1.2633910000	1.4117620000
23	H	5.4191910000	1.0045190000	3.1057550000
24	H	4.7047340000	0.3150610000	4.5935560000
25	H	5.4941930000	-1.1439120000	1.9835470000
26	H	4.9608050000	-1.9778900000	3.5018590000
27	H	6.2829440000	-3.2720810000	2.0539510000
28	H	7.5149440000	-4.2619550000	2.8819920000
29	H	7.9254900000	-4.1601340000	0.4235470000
30	H	7.9686630000	-2.3669780000	0.4913750000

H	9.8772750000	-1.2241620000	1.7820320000
H	11.1159950000	-2.2486310000	2.5797720000
H	9.3943740000	-3.1403380000	4.1539980000
H	9.4727410000	-1.3494230000	4.2185840000
H	0.7303430000	-2.7513960000	3.3029130000
H	0.9491510000	-0.2612400000	1.7307780000
H	0.3403500000	-0.4709380000	3.3875440000

6.7. Piperazine amine core based bone-targeting ionizable lipids 'Type 2'

Type2-P1-C12

P	23.1347640000	-3.4042230000	-2.2324780000
P	-3.3001430000	6.5423680000	3.6685300000
P	4.9027690000	4.5619280000	2.2373930000
P	3.5505000000	2.6866800000	4.2008980000
O	15.9341830000	3.8927000000	0.9548550000
O	18.7211430000	1.5724140000	-2.4827190000
O	23.1032060000	-3.7180160000	-0.5229380000
O	23.1316800000	-4.7429920000	-2.8818790000
O	21.9752650000	-2.4080850000	-2.7261670000
O	24.6317560000	-2.7472240000	-2.2349890000
O	27.1764120000	-2.3686710000	-1.5148130000
O	27.1605290000	-4.6356330000	-1.2489690000
O	26.8706200000	-0.9466530000	-4.7885850000
O	26.7723810000	0.1869320000	-6.7327730000
O	17.8251290000	1.1697580000	-0.4530130000
O	-8.1629370000	6.2775610000	-0.5748780000
O	-8.1933850000	4.8955550000	1.2369060000
O	-7.8545290000	1.1420120000	2.7857860000
O	-6.6520060000	3.0336480000	2.6479010000
O	-1.9001120000	5.6105630000	3.2352780000

1	O	-4.7603790000	6.0148310000	3.3558700000
2	O	-2.8156890000	6.8440630000	5.1962790000
3	O	-2.8502820000	7.7855800000	2.9870150000
4	O	10.2124150000	-2.3803970000	2.1416890000
5	O	10.8565550000	-4.3082410000	1.1878130000
6	O	-12.6863960000	-5.8605410000	-1.3806010000
7	O	-15.5163950000	-2.1691940000	0.7522080000
8	O	-13.0279000000	2.0842930000	-0.6732630000
9	O	-0.0103740000	0.0907420000	-4.4636350000
10	O	5.6829140000	2.0370900000	2.7314780000
11	O	3.6071210000	5.4052640000	2.0852920000
12	O	5.8130570000	5.3855850000	3.4691960000
13	O	5.7345200000	4.8398190000	0.7393240000
14	O	2.2047560000	3.4548910000	4.0927380000
15	O	3.0730540000	1.1087500000	4.7443170000
16	O	4.3553670000	3.4305860000	5.5514770000
17	O	0.6567640000	-2.8495700000	0.4484610000
18	O	4.7273570000	-4.4487950000	-0.7451040000
19	N	19.2405110000	-0.3551460000	-1.4297950000
20	N	-0.9494540000	7.7344400000	3.8979290000
21	N	-14.3107560000	-3.5321980000	-1.5776040000
22	N	-11.3582340000	-1.1023660000	-1.6249280000
23	N	-1.0662150000	-0.9873710000	-1.0455990000
24	N	2.1929730000	-1.1990750000	0.3764090000
25	N	6.5441620000	-3.1317880000	-0.8048140000
26	N	9.1110700000	-2.9917770000	0.2857970000
27	N	-7.6082940000	-0.6954800000	-2.2166940000
28	N	-4.7540650000	-0.2615170000	-1.9420330000
29	C	15.2126660000	4.1073120000	2.1490650000
30	C	16.5246290000	2.6164530000	0.9128910000

1	C	17.2733600000	2.4599960000	-0.4024750000
2	C	25.8849280000	16.7713260000	-5.2991900000
3	C	26.0160610000	15.4651290000	-6.0935790000
4	C	25.9963300000	14.2326360000	-5.1754130000
5	C	26.1264790000	12.9224460000	-5.9689910000
6	C	25.0133880000	-8.8350690000	14.7794730000
7	C	25.1900420000	-9.1500240000	13.2880640000
8	C	25.2477280000	-7.8720360000	12.4360400000
9	C	25.4268930000	-8.1851880000	10.9416190000
10	C	25.4893350000	-6.9081220000	10.0876620000
11	C	26.1082460000	11.6883270000	-5.0521080000
12	C	21.1506140000	-1.8032470000	-1.7637120000
13	C	20.1346110000	-0.8959800000	-2.4556570000
14	C	25.5825690000	-3.1243080000	-3.1969300000
15	C	26.8995450000	-2.4020050000	-2.8912290000
16	C	26.8752530000	-0.9541660000	-3.3858120000
17	C	27.0843600000	-3.5192080000	-0.7878430000
18	C	26.8088830000	-3.1938990000	0.6718020000
19	C	26.7571900000	0.2019820000	-5.5193150000
20	C	26.6118090000	1.4933450000	-4.7193520000
21	C	26.5949840000	2.7581890000	-5.5967990000
22	C	26.4654840000	4.0453310000	-4.7648200000
23	C	26.4635880000	5.2980530000	-5.6563990000
24	C	26.3382300000	6.5945650000	-4.8399070000
25	C	26.3466520000	7.8388630000	-5.7432960000
26	C	26.2204580000	9.1422530000	-4.9379600000
27	C	26.2357710000	10.3802610000	-5.8497880000
28	C	26.6510340000	-4.4419640000	1.5544020000
29	C	26.3962010000	-4.0860720000	3.0283350000
30	C	26.2714840000	-5.3456060000	3.9013790000

1	C	26.0472200000	-5.0079190000	5.3843210000
2	C	25.9519790000	-6.2763280000	6.2483130000
3	C	25.7507650000	-5.9514280000	7.7373090000
4	C	25.6760050000	-7.2254170000	8.5950210000
5	C	18.5983090000	0.8432960000	-1.5267180000
6	C	-21.3477070000	5.0582870000	-2.1926230000
7	C	-21.2279630000	5.3272520000	-0.6874750000
8	C	-20.5405810000	4.1707150000	0.0559620000
9	C	-20.4529200000	4.4466890000	1.5648110000
10	C	-19.7546280000	3.3153350000	2.3352600000
11	C	-19.7743200000	3.5711620000	3.8508650000
12	C	-19.0016210000	2.4983560000	4.6337340000
13	C	-18.9486450000	2.8195640000	6.1346860000
14	C	-18.2527230000	1.7197920000	6.8954530000
15	C	-16.9473200000	1.6544190000	7.2029880000
16	C	-15.8717970000	2.6562430000	6.8575970000
17	C	-14.8082230000	2.0135300000	5.9524070000
18	C	-13.5974840000	2.9308900000	5.7095100000
19	C	-12.5228590000	2.2282160000	4.8627950000
20	C	-11.2401540000	3.0624390000	4.7079550000
21	C	-10.1512930000	2.2828400000	3.9498400000
22	C	-26.1653870000	8.1815150000	-1.2735140000
23	C	-24.7295650000	8.6303350000	-0.9718140000
24	C	-24.2438150000	8.1125840000	0.3912010000
25	C	-22.8052770000	8.5602040000	0.6954130000
26	C	-22.3027620000	8.0040590000	2.0375370000
27	C	-20.8327770000	8.3701990000	2.2963510000
28	C	-20.3208960000	7.7971890000	3.6278710000
29	C	-18.8275350000	8.0900100000	3.8384990000
30	C	-18.3200840000	7.4326470000	5.0978990000

1	C	-17.4460630000	6.4158020000	5.1739900000
2	C	-16.7838670000	5.7095570000	4.0164740000
3	C	-15.3784670000	6.2746930000	3.7752510000
4	C	-14.6569460000	5.5511360000	2.6270950000
5	C	-13.2337680000	6.0962730000	2.4358220000
6	C	-12.4742110000	5.3731300000	1.3122950000
7	C	-11.0263250000	5.8767530000	1.2122870000
8	C	-10.2314590000	5.1764220000	0.0992160000
9	C	-8.7558040000	5.5340230000	0.1733090000
10	C	-8.8411830000	3.0788170000	3.8037220000
11	C	-7.7583390000	2.3267060000	3.0335760000
12	C	-6.8427820000	5.1354000000	1.5199180000
13	C	-6.4677320000	4.4120750000	2.8157260000
14	C	-4.9904670000	4.6440010000	3.1497590000
15	C	-0.4518650000	6.8780200000	5.0564620000
16	C	-1.6779410000	6.2908960000	5.7883060000
17	C	10.0669530000	-3.1973230000	1.2422560000
18	C	13.0101630000	-3.6468530000	2.0584550000
19	C	11.3334790000	-4.9085670000	3.4781420000
20	C	12.4380640000	-6.0249210000	1.5299250000
21	C	11.8769990000	-4.6927720000	2.0513250000
22	C	-9.8857550000	-0.9791320000	-13.7416610000
23	C	-9.9011780000	0.0467140000	-12.6120420000
24	C	-10.4765710000	-0.5563080000	-11.3257180000
25	C	-10.4893490000	0.4747590000	-10.1905820000
26	C	-11.0619840000	-0.1341200000	-8.9054950000
27	C	-11.0715540000	0.8883100000	-7.7624100000
28	C	-11.6364830000	0.2642990000	-6.4816540000
29	C	-27.1687720000	-9.2988470000	-1.2450870000
30	C	-26.5108090000	-8.0393110000	-0.6885660000

1	C	-24.9945860000	-8.0688880000	-0.9053370000
2	C	-24.3291810000	-6.8037380000	-0.3526740000
3	C	-22.8144850000	-6.8470660000	-0.5791330000
4	C	-22.1321480000	-5.5846160000	-0.0408210000
5	C	-20.6230840000	-5.6435810000	-0.2948830000
6	C	-25.0615610000	-11.3156000000	-4.0509250000
7	C	-23.8894660000	-11.1199460000	-3.0942420000
8	C	-22.8299210000	-10.1927220000	-3.7009620000
9	C	-21.6581200000	-9.9860720000	-2.7341790000
10	C	-20.5889450000	-9.0776740000	-3.3540610000
11	C	-19.4209290000	-8.8570290000	-2.3856040000
12	C	-18.3331500000	-7.9842630000	-3.0249980000
13	C	3.4243880000	-12.8353110000	-1.2210970000
14	C	3.6574060000	-11.5747150000	-2.0487080000
15	C	2.6770720000	-10.4645640000	-1.6535610000
16	C	2.9122790000	-9.2003090000	-2.4885890000
17	C	1.9290930000	-8.0916410000	-2.0954340000
18	C	2.1589300000	-6.8287850000	-2.9336630000
19	C	1.1710640000	-5.7242550000	-2.5420760000
20	C	-19.9135590000	-4.3836690000	0.2157200000
21	C	-17.1754700000	-7.7422980000	-2.0492610000
22	C	-16.0637520000	-6.9085410000	-2.7010690000
23	C	-14.9227750000	-6.6436240000	-1.7009160000
24	C	-13.7374950000	-5.8711630000	-2.3129340000
25	C	-18.4152100000	-4.4617490000	-0.0933030000
26	C	-17.6663420000	-3.2061500000	0.3855100000
27	C	-16.1373150000	-3.2666870000	0.1335690000
28	C	-14.0386240000	-4.4180190000	-2.7480430000
29	C	-15.7690720000	-3.3417800000	-1.3698310000
30	C	-11.6386330000	1.2690240000	-5.3224270000

1	C	-12.1854260000	0.6179810000	-4.0469060000
2	C	-12.1873780000	1.6093490000	-2.8679900000
3	C	-12.7576870000	1.0225200000	-1.5541010000
4	C	-11.7992420000	0.0567200000	-0.8064660000
5	C	1.3957750000	-4.4612960000	-3.3813100000
6	C	0.4087320000	-3.3576230000	-2.9820650000
7	C	0.6529570000	-2.0825860000	-3.7882120000
8	C	-0.3482630000	-0.9249870000	-3.5509970000
9	C	-0.3183730000	-0.2752120000	-2.1324440000
10	C	-13.6171410000	-2.2275920000	-1.7197750000
11	C	-12.1266170000	-2.3445150000	-1.3523400000
12	C	-9.9059180000	-1.3769010000	-1.4696480000
13	C	3.3172000000	0.7130100000	1.5131150000
14	C	3.6564950000	2.2085650000	1.3993010000
15	C	4.4909050000	2.7645940000	2.5796550000
16	C	-0.3640720000	-0.7746790000	0.2542830000
17	C	0.8817090000	-1.6490820000	0.3657300000
18	C	2.5509230000	0.2257360000	0.2729810000
19	C	3.2857020000	-2.1874490000	0.4583080000
20	C	4.1470980000	-2.1905980000	-0.8168810000
21	C	5.1761300000	-3.3093490000	-0.7786920000
22	C	7.2133290000	-1.8245420000	-0.7921010000
23	C	8.2476720000	-1.8102430000	0.3462130000
24	C	8.8917450000	-3.9523350000	-0.7911950000
25	C	7.4025520000	-4.3263240000	-0.8055240000
26	C	-2.4688790000	-0.4969310000	-0.8874410000
27	C	-3.3526070000	-0.7056020000	-2.1341070000
28	C	-9.0334660000	-0.3049380000	-2.1611320000
29	C	-5.5015950000	-1.1457890000	-1.0188060000
30	C	-6.9600560000	-0.6842610000	-0.8903830000

1	C	-6.8779850000	0.2149160000	-3.1191020000
2	C	-5.4274300000	-0.2449490000	-3.2588520000
3	H	15.8736620000	4.0192460000	2.9858620000
4	H	14.4340310000	3.3780220000	2.2313020000
5	H	14.7834160000	5.0873390000	2.1352110000
6	H	17.2568550000	2.5871140000	1.6925600000
7	H	15.7257260000	1.9047370000	0.9021780000
8	H	18.0917920000	3.1488720000	-0.3796920000
9	H	16.5458030000	2.5192660000	-1.1848100000
10	H	23.9701400000	-3.5432620000	-0.1494580000
11	H	26.6996220000	16.8531760000	-4.6103690000
12	H	24.9607420000	16.7695780000	-4.7599530000
13	H	25.9029780000	17.6020860000	-5.9732910000
14	H	26.9851750000	15.4858740000	-6.5466650000
15	H	25.1408150000	15.3972060000	-6.7053230000
16	H	26.8719640000	14.3006410000	-4.5642340000
17	H	25.0274650000	14.2128230000	-4.7217520000
18	H	27.0949640000	12.9430520000	-6.4234270000
19	H	25.2503850000	12.8543510000	-6.5795000000
20	H	25.8430610000	-9.2295690000	15.3280290000
21	H	24.1074040000	-9.2810390000	15.1333140000
22	H	24.9665200000	-7.7749830000	14.9170260000
23	H	26.1475650000	-9.6177890000	13.1919120000
24	H	24.3066230000	-9.6723820000	12.9853860000
25	H	26.1302680000	-7.3490580000	12.7402060000
26	H	24.2896410000	-7.4052450000	12.5313090000
27	H	26.3835670000	-8.6550410000	10.8472130000
28	H	24.5429120000	-8.7055300000	10.6371180000
29	H	26.3705140000	-6.3848300000	10.3952140000
30	H	24.5309560000	-6.4407680000	10.1770500000

1	H	26.9854340000	11.7545830000	-4.4429680000
2	H	25.1405010000	11.6680880000	-4.5960800000
3	H	20.5944430000	-2.5889940000	-1.2966190000
4	H	21.7790130000	-1.1689700000	-1.1740450000
5	H	19.5417180000	-1.5170040000	-3.0941760000
6	H	20.6779970000	-0.0707520000	-2.8663040000
7	H	18.4932710000	-1.0189070000	-1.3973420000
8	H	25.7623840000	-4.1707810000	-3.0647930000
9	H	25.2359200000	-2.7559000000	-4.1398040000
10	H	27.6568080000	-2.9590940000	-3.4022120000
11	H	25.9429220000	-0.5344060000	-3.0704400000
12	H	27.7959790000	-0.5053540000	-3.0764050000
13	H	27.6814730000	-2.6888680000	1.0301750000
14	H	25.8599150000	-2.6998650000	0.6888460000
15	H	27.4927350000	1.5659740000	-4.1163630000
16	H	25.6486720000	1.4447310000	-4.2557740000
17	H	27.5533040000	2.8048620000	-6.0704480000
18	H	25.7070730000	2.6968610000	-6.1907310000
19	H	27.3488770000	4.1051860000	-4.1640420000
20	H	25.5031170000	4.0065390000	-4.2987240000
21	H	27.4252320000	5.3323080000	-6.1243390000
22	H	25.5790680000	5.2362920000	-6.2553220000
23	H	27.2196320000	6.6557280000	-4.2363430000
24	H	25.3738790000	6.5659630000	-4.3771890000
25	H	27.3114470000	7.8657300000	-6.2051940000
26	H	25.4658520000	7.7749380000	-6.3474530000
27	H	27.0991850000	9.2059960000	-4.3307740000
28	H	25.2539080000	9.1192530000	-4.4795360000
29	H	27.2028180000	10.4024020000	-6.3072050000
30	H	25.3577490000	10.3143350000	-6.4577610000

1	H	27.5942640000	-4.9464880000	1.5284700000
2	H	25.7683410000	-4.9399230000	1.2111950000
3	H	27.2695370000	-3.5697680000	3.3683550000
4	H	25.4423140000	-3.6023600000	3.0603100000
5	H	27.2189620000	-5.8399450000	3.8483310000
6	H	25.3862180000	-5.8491350000	3.5732590000
7	H	26.9239180000	-4.4879710000	5.7098280000
8	H	25.0905180000	-4.5322930000	5.4426570000
9	H	26.9036440000	-6.7598240000	6.1743910000
10	H	25.0664640000	-6.7869530000	5.9320450000
11	H	26.6301790000	-5.4293170000	8.0518400000
12	H	24.7928290000	-5.4810580000	7.8148420000
13	H	26.6306700000	-7.7007100000	8.5077770000
14	H	24.7910970000	-7.7417700000	8.2864480000
15	H	-20.3712980000	4.9282510000	-2.6104910000
16	H	-21.9247390000	4.1713570000	-2.3516500000
17	H	-21.8306280000	5.8876040000	-2.6658250000
18	H	-20.5811220000	6.1735460000	-0.5860640000
19	H	-22.2287960000	5.3707400000	-0.3115210000
20	H	-19.5332760000	4.1378350000	-0.3034200000
21	H	-21.1773680000	3.3184190000	-0.0580270000
22	H	-19.8279590000	5.3084800000	1.6726960000
23	H	-21.4616080000	4.4693200000	1.9210930000
24	H	-18.7266990000	3.3390030000	2.0391160000
25	H	-20.3385670000	2.4339060000	2.1709490000
26	H	-19.2475280000	4.4896820000	4.0048500000
27	H	-20.7965340000	3.4832750000	4.1546010000
28	H	-17.9918780000	2.5386770000	4.2820350000
29	H	-19.5651350000	1.5936290000	4.5398070000
30	H	-18.3382790000	3.6924400000	6.2368600000

1	H	-19.9618970000	2.8320470000	6.4782930000
2	H	-18.8634350000	0.9053950000	7.2251330000
3	H	-16.6333510000	0.7939490000	7.7560840000
4	H	-15.3810940000	2.9054360000	7.7752100000
5	H	-16.3432280000	3.4221190000	6.2778660000
6	H	-15.2736950000	1.8790430000	4.9983900000
7	H	-14.4348900000	1.1668370000	6.4896540000
8	H	-13.9540250000	3.7520200000	5.1234000000
9	H	-13.1571050000	3.1092770000	6.6682300000
10	H	-12.9352900000	2.1353880000	3.8798490000
11	H	-12.2415710000	1.3527020000	5.4098340000
12	H	-11.4968470000	3.8984830000	4.0914820000
13	H	-10.8633820000	3.2223830000	5.6965710000
14	H	-10.5246740000	2.1420670000	2.9570310000
15	H	-9.9162790000	1.4346510000	4.5583170000
16	H	-26.1754430000	7.5967190000	-2.1695120000
17	H	-26.7884240000	9.0418560000	-1.4021140000
18	H	-26.5336220000	7.5929660000	-0.4593190000
19	H	-24.1087170000	8.1643180000	-1.7082080000
20	H	-24.7588810000	9.6971170000	-0.8942590000
21	H	-24.2154270000	7.0457840000	0.3135340000
22	H	-24.8646260000	8.5790760000	1.1273260000
23	H	-22.1886880000	8.1206630000	-0.0605770000
24	H	-22.8397190000	9.6239080000	0.8060910000
25	H	-22.3350350000	6.9380640000	1.9508590000
26	H	-22.8723230000	8.4964940000	2.7978060000
27	H	-20.2652040000	7.8904380000	1.5265520000
28	H	-20.8033550000	9.4349430000	2.3981010000
29	H	-20.4100590000	6.7337010000	3.5507810000
30	H	-20.8415810000	8.3231010000	4.4006600000

1	H	-18.3100830000	7.6261210000	3.0248960000
2	H	-18.7445630000	9.1455350000	3.9930420000
3	H	-18.6944100000	7.8152740000	6.0243850000
4	H	-17.1935970000	6.0714390000	6.1550990000
5	H	-17.3603530000	5.9440330000	3.1460810000
6	H	-16.6555160000	4.6899400000	4.3144780000
7	H	-15.5137920000	7.2861400000	3.4534400000
8	H	-14.8158100000	6.0658450000	4.6610830000
9	H	-15.1949480000	5.7900670000	1.7335820000
10	H	-14.5515890000	4.5307680000	2.9314780000
11	H	-13.3446010000	7.1109060000	2.1146760000
12	H	-12.7068230000	5.8760320000	3.3406550000
13	H	-12.9544070000	5.6480000000	0.3964580000
14	H	-12.4176960000	4.3437420000	1.5987690000
15	H	-11.0894120000	6.9065730000	0.9287550000
16	H	-10.5529090000	5.6031010000	2.1320110000
17	H	-10.5929730000	5.5730870000	-0.8264540000
18	H	-10.2986550000	4.1264370000	0.2939400000
19	H	-9.0825920000	3.9356870000	3.2100920000
20	H	-8.4618720000	3.2052020000	4.7962180000
21	H	-6.2755250000	4.6804070000	0.7349970000
22	H	-6.7469710000	6.1838860000	1.7106960000
23	H	-7.0857160000	4.7908440000	3.6028280000
24	H	-4.4309550000	4.3650140000	2.2814200000
25	H	-4.8059600000	4.1568690000	4.0844030000
26	H	-0.6650010000	8.6828030000	4.0382640000
27	H	-1.9474550000	7.6887620000	3.8542540000
28	H	0.0824070000	6.0546760000	4.6303670000
29	H	0.0380700000	7.5354010000	5.7440040000
30	H	-1.6944890000	5.2400470000	5.5874520000

H	-1.6430000000	6.6420220000	6.7984500000
H	-1.8881940000	4.7959510000	3.7430830000
H	12.6605130000	-2.6680150000	2.4441210000
H	13.8473980000	-3.9836370000	2.7072390000
H	13.4012150000	-3.4953040000	1.0293960000
H	10.9311050000	-3.9694370000	3.9087240000
H	12.1406140000	-5.2681360000	4.1524240000
H	10.5187710000	-5.6642410000	3.4700170000
H	13.2631440000	-6.3908540000	2.1789970000
H	11.6401030000	-6.7983180000	1.5050570000
H	12.8304910000	-5.9024510000	0.4971720000
H	-9.2568220000	-1.8513690000	-13.4626420000
H	-10.9174070000	-1.3283540000	-13.9604850000
H	-9.4648290000	-0.5166420000	-14.6591960000
H	-8.8609630000	0.3941550000	-12.4291550000
H	-10.5183350000	0.9168310000	-12.9255810000
H	-9.8567710000	-1.4287780000	-11.0215160000
H	-11.5155870000	-0.9051610000	-11.5167710000
H	-9.4500980000	0.8228660000	-9.9998870000
H	-11.1098870000	1.3472820000	-10.4922510000
H	-10.4412750000	-1.0083460000	-8.6084310000
H	-12.1014680000	-0.4821120000	-9.0956040000
H	-10.0317960000	1.2355820000	-7.5728530000
H	-11.6945070000	1.7628940000	-8.0527830000
H	-11.0123700000	-0.6133380000	-6.2011070000
H	-12.6767250000	-0.0828660000	-6.6689300000
H	-26.7659770000	-10.2018100000	-0.7386420000
H	-28.2647900000	-9.2534150000	-1.0737610000
H	-26.9832570000	-9.3804370000	-2.3370460000
H	-26.7314280000	-7.9684150000	0.3988330000

1	H	-26.9446800000	-7.1522800000	-1.1997560000
2	H	-24.5688940000	-8.9587780000	-0.3912100000
3	H	-24.7830550000	-8.1465330000	-1.9948000000
4	H	-24.5367740000	-6.7225110000	0.7372120000
5	H	-24.7523760000	-5.9122610000	-0.8661000000
6	H	-22.3915620000	-7.7380880000	-0.0645720000
7	H	-22.6128090000	-6.9339330000	-1.6700000000
8	H	-22.3217040000	-5.4956950000	1.0515900000
9	H	-22.5553710000	-4.6923770000	-0.5529210000
10	H	-20.1974910000	-6.5351940000	0.2164320000
11	H	-20.4471990000	-5.7417080000	-1.3895590000
12	H	-25.8136540000	-11.9872190000	-3.5857830000
13	H	-25.5447890000	-10.3404630000	-4.2733800000
14	H	-24.7125670000	-11.7745290000	-5.0004040000
15	H	-23.4363330000	-12.1107520000	-2.8725000000
16	H	-24.2713420000	-10.6860720000	-2.1463190000
17	H	-22.4524400000	-10.6413570000	-4.6464260000
18	H	-23.2907520000	-9.2067620000	-3.9316440000
19	H	-21.2026000000	-10.9716020000	-2.4919590000
20	H	-22.0360830000	-9.5284470000	-1.7946360000
21	H	-20.2089270000	-9.5507370000	-4.2864570000
22	H	-21.0408450000	-8.0938120000	-3.6100630000
23	H	-18.9798410000	-9.8402330000	-2.1094670000
24	H	-19.8005520000	-8.3656800000	-1.4638170000
25	H	-17.9478290000	-8.4950330000	-3.9350090000
26	H	-18.7695420000	-7.0046460000	-3.3213240000
27	H	3.5700690000	-12.6206450000	-0.1409410000
28	H	2.3937660000	-13.2176020000	-1.3816100000
29	H	4.1465400000	-13.6210490000	-1.5279760000
30	H	4.7010620000	-11.2268500000	-1.8868710000

1	H	3.5268600000	-11.8232130000	-3.1245790000
2	H	2.8105390000	-10.2230510000	-0.5758550000
3	H	1.6355590000	-10.8214680000	-1.8139830000
4	H	3.9532220000	-8.8420200000	-2.3284090000
5	H	2.7790670000	-9.4430320000	-3.5660920000
6	H	2.0625550000	-7.8470260000	-1.0183550000
7	H	0.8885350000	-8.4519600000	-2.2542580000
8	H	3.1985280000	-6.4655930000	-2.7742470000
9	H	2.0262010000	-7.0740520000	-4.0105770000
10	H	1.3058890000	-5.4778510000	-1.4653170000
11	H	0.1314610000	-6.0878350000	-2.6988710000
12	H	-20.0671680000	-4.2862150000	1.3128880000
13	H	-20.3437000000	-3.4903370000	-0.2886720000
14	H	-16.7529360000	-8.7190340000	-1.7254110000
15	H	-17.5645000000	-7.2095370000	-1.1551290000
16	H	-15.6619250000	-7.4635330000	-3.5771360000
17	H	-16.4952660000	-5.9579100000	-3.0689300000
18	H	-14.5390360000	-7.6331700000	-1.3666000000
19	H	-15.3016840000	-6.1139490000	-0.8026000000
20	H	-11.8398020000	-5.7937460000	-1.8953220000
21	H	-13.4244910000	-6.4530420000	-3.2129120000
22	H	-17.9831300000	-5.3542440000	0.4098350000
23	H	-18.3139200000	-4.5777610000	-1.1906320000
24	H	-17.8271910000	-3.1101630000	1.4820760000
25	H	-18.0912680000	-2.3050960000	-0.1089330000
26	H	-15.9253100000	-1.3437370000	0.3802540000
27	H	-15.7551520000	-4.1838460000	0.6317130000
28	H	-13.1330760000	-4.0869250000	-3.2997950000
29	H	-14.8470990000	-4.3694970000	-3.5105130000
30	H	-16.3091180000	-4.2048370000	-1.7779370000

1	H	-16.1474560000	-2.4524980000	-1.9238610000
2	H	-10.5973760000	1.6144620000	-5.1369200000
3	H	-12.2658520000	2.1469850000	-5.5921420000
4	H	-11.5651590000	-0.2657780000	-3.8339990000
5	H	-13.2262550000	0.2708920000	-4.2289440000
6	H	-11.1729300000	2.0250670000	-2.6840670000
7	H	-12.8487690000	2.4491040000	-3.1772920000
8	H	-13.9088150000	2.4655860000	-0.9290880000
9	H	-13.7136930000	0.5121250000	-1.7940470000
10	H	-12.2713010000	-0.2850840000	0.1421050000
11	H	-10.9353750000	0.6722330000	-0.4815200000
12	H	2.4360670000	-4.1003990000	-3.2233890000
13	H	1.2617060000	-4.7045500000	-4.4584370000
14	H	0.5671840000	-3.1446380000	-1.9182930000
15	H	-0.6319950000	-3.7120500000	-3.1496160000
16	H	1.6873970000	-1.7186680000	-3.6004370000
17	H	0.5788570000	-2.3571660000	-4.8642510000
18	H	-0.4647370000	-0.1151980000	-5.3227380000
19	H	-1.3677220000	-1.2891140000	-3.7868990000
20	H	0.7576630000	-0.1398030000	-1.8896970000
21	H	-0.7209700000	0.7612570000	-2.2033920000
22	H	-14.0558710000	-1.4867030000	-1.0315190000
23	H	-13.7355940000	-1.8418500000	-2.7576570000
24	H	-12.0256930000	-2.6466290000	-0.2854400000
25	H	-11.6936910000	-3.1612260000	-1.9636850000
26	H	-9.6543250000	-1.4875580000	-0.3933530000
27	H	-9.6505100000	-2.3325860000	-1.9802020000
28	H	2.6802430000	0.5319530000	2.3996440000
29	H	4.2659070000	0.1494350000	1.6185520000
30	H	4.2441590000	2.3521770000	0.4653440000

1	H	2.7159200000	2.7945290000	1.3045430000
2	H	6.2070290000	2.1398500000	1.8947090000
3	H	5.1420960000	5.8484430000	4.0314340000
4	H	6.6660360000	4.5519840000	0.9125700000
5	H	3.9200310000	0.6691020000	5.0090730000
6	H	3.9810120000	4.3450050000	5.6155760000
7	H	3.1574420000	0.4005270000	-0.6376760000
8	H	1.6839640000	0.8855030000	0.1521480000
9	H	2.8989900000	-3.2195920000	0.5897480000
10	H	3.9173370000	-1.9991680000	1.3497530000
11	H	3.4902300000	-2.3682570000	-1.6954220000
12	H	4.5823480000	-1.1979360000	-0.9625850000
13	H	6.5584420000	-0.9508440000	-0.6676650000
14	H	7.7447210000	-1.6874710000	-1.7583390000
15	H	7.6965730000	-1.8215380000	1.3127770000
16	H	8.8543200000	-0.8803280000	0.2884410000
17	H	9.5187700000	-4.8652680000	-0.7073360000
18	H	9.1507390000	-3.4717970000	-1.7592470000
19	H	7.1896400000	-4.9176130000	0.1127610000
20	H	7.1925220000	-4.9578710000	-1.6962820000
21	H	-2.9281260000	-1.0565220000	-0.0478420000
22	H	-2.4800860000	0.5840600000	-0.6188070000
23	H	-3.3249580000	-1.7757150000	-2.4418460000
24	H	-2.9132910000	-0.0794720000	-2.9366870000
25	H	-9.1403550000	0.6950790000	-1.6908560000
26	H	-9.4071010000	-0.2259670000	-3.2035080000
27	H	-5.0674610000	-1.1156070000	-0.0003350000
28	H	-5.4825070000	-2.1979330000	-1.3849160000
29	H	-7.4652560000	-1.3863990000	-0.1970620000
30	H	-6.9927430000	0.3370590000	-0.4462780000

H	-6.9029210000	1.2594170000	-2.7309930000
H	-7.3379940000	0.2059410000	-4.1323700000
H	-5.4122850000	-1.2601670000	-3.7193240000
H	-4.9100470000	0.4604090000	-3.9464560000
H	-1.1267490000	-2.0116540000	-1.2310270000
H	-1.0035060000	-1.0838170000	1.1126530000
H	-0.2223540000	0.3069110000	0.4197900000

6.8. Branched amine core based bone-targeting ionizable lipids 'Type 3'

Type3-B1-C12

P	-15.4233610000	-6.9436640000	-0.3619820000
P	12.2947420000	-8.3360360000	-0.2825490000
P	4.1670880000	-8.0715370000	1.1369720000
P	2.6714730000	-9.4568990000	-1.0928720000
O	-5.0868890000	-9.8423750000	-0.4477030000
O	-9.2274720000	-8.0012650000	-2.5533320000
O	-15.2492410000	-7.5390340000	1.2619670000
O	-16.2135930000	-5.6886060000	-0.2420040000
O	-14.0295670000	-6.7953460000	-1.1467080000
O	-16.3554360000	-8.1846250000	-0.8756880000
O	-18.1832060000	-10.1294750000	-0.7571960000
O	-19.2678540000	-8.6629340000	0.6149690000
O	-17.7925460000	-9.3730370000	-4.2369100000
O	-17.4848150000	-9.1698170000	-6.4591910000
O	-8.3112290000	-8.2385250000	-0.5066550000
O	16.1661740000	-3.3958210000	-1.8088810000
O	16.3457340000	-4.3520150000	0.2519150000
O	15.7781180000	-4.1252920000	4.2804810000
O	14.8537350000	-5.1201430000	2.4918650000
O	10.7193450000	-7.8808050000	0.2892400000

1	O	13.5776010000	-7.4892730000	0.0990370000
2	O	12.1599940000	-9.9070660000	0.1339120000
3	O	11.9046330000	-8.3974450000	-1.7165840000
4	O	-17.9323150000	1.6922540000	0.4643760000
5	O	-12.8453410000	-2.6723490000	-0.0549080000
6	O	-10.3783660000	6.2785380000	0.3513050000
7	O	-10.8743500000	7.2992050000	-4.5200830000
8	O	-6.8462000000	5.8057110000	-2.0187310000
9	O	4.1223860000	6.6175700000	1.0210370000
10	O	4.4441340000	4.8044530000	-3.5080460000
11	O	11.8696000000	0.5621310000	-4.4543730000
12	O	12.1356550000	-1.8857190000	1.5445820000
13	O	1.7030820000	0.9755980000	-3.4365530000
14	O	-4.8297910000	1.5931250000	-3.0433280000
15	O	2.6882930000	-1.0772980000	1.1177930000
16	O	5.6379020000	-7.9236620000	0.2212630000
17	O	4.2733120000	-6.9394160000	2.4506970000
18	O	4.3139250000	-9.4429470000	1.8507110000
19	O	3.9629020000	-9.3634820000	-2.2511070000
20	O	2.7824190000	-11.0300660000	-0.3658790000
21	O	1.3877570000	-9.5370380000	-1.9639700000
22	O	1.5029790000	-8.2193260000	0.9916280000
23	O	-1.9511420000	-2.4428870000	1.7003960000
24	N	-10.4515050000	-7.4349170000	-0.7436710000
25	N	10.2260130000	-9.6123380000	-1.3269690000
26	N	-11.9641530000	5.6729380000	-2.0976110000
27	N	5.9373130000	5.1646500000	-0.9674310000
28	N	6.6943830000	1.4634550000	-1.6430280000
29	N	10.2604220000	0.0710970000	-1.0802470000
30	N	-14.3249830000	0.7281980000	0.2083900000

1	N	-10.9841840000	2.1107360000	-1.1895710000
2	N	-7.4096190000	2.1034970000	-2.4619770000
3	N	4.3310390000	-0.6350580000	-1.2710600000
4	N	1.9625500000	-3.0057970000	0.2168430000
5	N	-2.4847900000	-0.7863710000	0.2686910000
6	N	-4.4032490000	-0.0641870000	-1.5964200000
7	C	-4.1555770000	-10.1966480000	0.5522630000
8	C	-6.2377870000	-9.2366700000	0.0887170000
9	C	-7.1843730000	-8.8784050000	-1.0474560000
10	C	-8.0978090000	-21.0176770000	-13.4875050000
11	C	-9.0108330000	-19.7850180000	-13.5241060000
12	C	-9.4526590000	-19.3582320000	-12.1151700000
13	C	-10.3667260000	-18.1226570000	-12.1489470000
14	C	-16.7080510000	-12.3973540000	16.6978150000
15	C	-17.2833480000	-11.5435820000	15.5599160000
16	C	-16.8387270000	-12.0542330000	14.1800340000
17	C	-17.4157710000	-11.2015710000	13.0383490000
18	C	-16.9759780000	-11.7131210000	11.6567060000
19	C	-10.8108680000	-17.6948910000	-10.7404690000
20	C	-12.8554800000	-7.2562770000	-0.5292430000
21	C	-11.6632210000	-7.0242470000	-1.4559920000
22	C	-17.5208280000	-7.9493530000	-1.6227340000
23	C	-18.2127630000	-9.2919290000	-1.8841060000
24	C	-17.5491130000	-10.0525760000	-3.0340730000
25	C	-18.5567970000	-9.6292940000	0.4556280000
26	C	-17.8985350000	-10.4232360000	1.5730480000
27	C	-17.2471370000	-9.7627070000	-5.4272380000
28	C	-16.3285850000	-10.9802450000	-5.3766740000
29	C	-15.8322610000	-11.4303960000	-6.7626950000
30	C	-14.9235150000	-12.6688500000	-6.6845370000

1	C	-14.4484510000	-13.1114370000	-8.0783300000
2	C	-13.5412380000	-14.3510820000	-8.0186400000
3	C	-13.0812540000	-14.7874160000	-9.4195280000
4	C	-12.1718570000	-16.0260140000	-9.3727450000
5	C	-11.7223800000	-16.4574380000	-10.7785080000
6	C	-18.2380090000	-9.8960850000	2.9760580000
7	C	-17.5791590000	-10.7291800000	4.0877890000
8	C	-17.9541080000	-10.2066580000	5.4844180000
9	C	-17.3286500000	-11.0473570000	6.6093500000
10	C	-17.7345860000	-10.5296160000	7.9991600000
11	C	-17.1339640000	-11.3763470000	9.1329400000
12	C	-17.5611320000	-10.8623440000	10.5177270000
13	C	-9.3211020000	-7.8915340000	-1.3534020000
14	C	28.4856560000	1.6477750000	-0.9519000000
15	C	28.6960670000	0.1966200000	-0.5024250000
16	C	28.0060620000	-0.1015940000	0.8383720000
17	C	28.2493370000	-1.5514790000	1.2846000000
18	C	27.5575050000	-1.8867430000	2.6150620000
19	C	27.9034470000	-3.3083490000	3.0865040000
20	C	27.1500890000	-3.6976300000	4.3678210000
21	C	27.4319830000	-5.1523750000	4.7716650000
22	C	26.7451010000	-5.4925640000	6.0697540000
23	C	25.5270600000	-6.0379060000	6.2177770000
24	C	24.5593600000	-6.4319980000	5.1278490000
25	C	23.2615030000	-5.6154300000	5.2393030000
26	C	22.1700880000	-6.0931080000	4.2661260000
27	C	20.8641500000	-5.3034880000	4.4572370000
28	C	19.7078640000	-5.8379240000	3.5954850000
29	C	18.3941050000	-5.0946610000	3.8950390000
30	C	33.7853940000	0.6134880000	-3.1088360000

1	C	32.5129180000	-0.1834370000	-3.4246870000
2	C	32.2284960000	-1.2519240000	-2.3573220000
3	C	30.9536930000	-2.0511400000	-2.6710600000
4	C	30.6435130000	-3.0885950000	-1.5799180000
5	C	29.3178570000	-3.8200620000	-1.8435250000
6	C	28.9941460000	-4.8431220000	-0.7426760000
7	C	27.6261030000	-5.5061090000	-0.9643570000
8	C	27.2808500000	-6.4285440000	0.1782880000
9	C	26.3016870000	-6.2535900000	1.0806780000
10	C	25.3361970000	-5.0958330000	1.1481810000
11	C	24.0058460000	-5.4660530000	0.4804860000
12	C	22.9820710000	-4.3227770000	0.5653100000
13	C	21.6411720000	-4.7316980000	-0.0623580000
14	C	20.5852390000	-3.6189130000	0.0319340000
15	C	19.2319100000	-4.0945290000	-0.5178870000
16	C	18.1469470000	-3.0091480000	-0.4404070000
17	C	16.7791810000	-3.5780690000	-0.7816840000
18	C	17.2074240000	-5.6263080000	3.0701400000
19	C	15.9011240000	-4.8832780000	3.3400140000
20	C	15.1226310000	-5.0208500000	0.1161670000
21	C	14.9035150000	-5.9146780000	1.3392940000
22	C	13.5660590000	-6.6540950000	1.2290550000
23	C	9.8426270000	-10.3575490000	-0.0538130000
24	C	11.0896950000	-10.4452110000	0.8521170000
25	C	-13.2691080000	11.7666030000	7.0713710000
26	C	-13.6633010000	10.2988480000	7.2121090000
27	C	-13.9803840000	9.6813600000	5.8463770000
28	C	-14.3814230000	8.2072570000	5.9834920000
29	C	-14.6918490000	7.6030350000	4.6102460000
30	C	-15.0892450000	6.1301170000	4.7162050000

1	C	-15.4177120000	5.5521470000	3.3320920000
2	C	-15.7443560000	4.0612320000	3.4398710000
3	C	-16.0926430000	3.4478740000	2.0754700000
4	C	-20.0332850000	1.0012020000	11.0687340000
5	C	-19.3066310000	-0.1041960000	10.3075360000
6	C	-18.5488430000	0.4614330000	9.1011740000
7	C	-17.8269720000	-0.6511000000	8.3302850000
8	C	-17.0862050000	-0.0812350000	7.1150800000
9	C	-16.3867250000	-1.1891200000	6.3163270000
10	C	-15.6923810000	-0.6054260000	5.0810530000
11	C	-15.0415320000	-1.6990610000	4.2235440000
12	C	-14.4223950000	-1.0862750000	2.9611430000
13	C	-18.9818890000	16.5565390000	3.7574240000
14	C	-17.9814610000	15.4687900000	4.1342170000
15	C	-17.4342200000	14.7577440000	2.8904920000
16	C	-16.4331280000	13.6676030000	3.2881900000
17	C	-15.8818600000	12.9346970000	2.0589830000
18	C	-14.8967410000	11.8409980000	2.4870790000
19	C	-14.3518640000	11.0662900000	1.2821450000
20	C	-19.8966470000	16.4538290000	0.0096380000
21	C	-19.3067910000	15.8444170000	-1.2582880000
22	C	-18.5078680000	14.5774700000	-0.9375470000
23	C	-17.8912540000	13.9744610000	-2.2055750000
24	C	-17.0917610000	12.7143690000	-1.8642310000
25	C	-16.4105320000	12.1187320000	-3.1038420000
26	C	-15.5958540000	10.8869230000	-2.7031900000
27	C	-5.6435730000	10.6752060000	-13.7387730000
28	C	-5.3807890000	9.4178180000	-12.9153830000
29	C	-6.0467550000	9.5082100000	-11.5372480000
30	C	-5.7762890000	8.2435030000	-10.7144110000

1	C	-6.4336940000	8.3307320000	-9.3314340000
2	C	-6.1504860000	7.0657890000	-8.5134170000
3	C	-6.7895030000	7.1456410000	-7.1211310000
4	C	3.3463170000	7.6456580000	8.4439200000
5	C	2.4477550000	7.1954380000	7.2955380000
6	C	3.1888090000	6.2378810000	6.3553540000
7	C	2.2837180000	5.7890360000	5.2017940000
8	C	3.0266050000	4.8342040000	4.2599260000
9	C	2.1263820000	4.3909420000	3.1002820000
10	C	2.8792490000	3.4503270000	2.1530650000
11	C	12.6487150000	8.7347190000	8.5443110000
12	C	13.2846210000	8.0314980000	7.3490820000
13	C	12.4171820000	6.8626800000	6.8677820000
14	C	13.0647670000	6.1546000000	5.6724320000
15	C	12.2108930000	4.9704530000	5.2029900000
16	C	12.8724710000	4.2526210000	4.0212640000
17	C	12.0479410000	3.0381150000	3.5765540000
18	C	10.9009370000	10.4798030000	5.0937870000
19	C	11.6750380000	9.8673780000	3.9306820000
20	C	10.9169270000	8.6811780000	3.3256880000
21	C	11.7105480000	8.0540470000	2.1740440000
22	C	10.9625940000	6.8548240000	1.5834960000
23	C	11.7760040000	6.1954740000	0.4631990000
24	C	11.0350050000	4.9781540000	-0.0949670000
25	C	11.8562170000	4.2670470000	-1.1798360000
26	C	11.1012670000	3.0330510000	-1.6802240000
27	C	7.4929370000	17.9860750000	-6.3065030000
28	C	7.0846150000	16.6251830000	-6.8637390000
29	C	6.9393450000	15.5908650000	-5.7422540000
30	C	6.5332080000	14.2213870000	-6.3019480000

1	C	6.3937880000	13.1939800000	-5.1736760000
2	C	6.0003950000	11.8126620000	-5.7150630000
3	C	5.8803320000	10.8073060000	-4.5657570000
4	C	10.4719600000	18.1390610000	-2.3822780000
5	C	9.8368380000	17.3623220000	-1.2319260000
6	C	9.6712960000	15.8818790000	-1.5914380000
7	C	9.0145700000	15.1016390000	-0.4447760000
8	C	8.8294870000	13.6297340000	-0.8289620000
9	C	8.1154740000	12.8390720000	0.2771280000
10	C	7.8783250000	11.3947860000	-0.1763990000
11	C	-14.7950300000	10.2973250000	-3.8745250000
12	C	-13.3972880000	9.9588110000	1.7472090000
13	C	-6.4832080000	5.8787930000	-6.3146890000
14	C	7.0545180000	10.5887330000	0.8406990000
15	C	-16.3118050000	1.9362170000	2.2370080000
16	C	-16.7185080000	1.1760560000	0.9482690000
17	C	-13.8937520000	-2.1433870000	2.0058320000
18	C	-13.2635480000	-1.5724200000	0.7147700000
19	C	-12.8994690000	9.1144880000	0.5715140000
20	C	-11.9597040000	7.9842600000	1.0468510000
21	C	-11.3964180000	7.1260570000	-0.1158110000
22	C	-13.9485430000	9.1322270000	-3.3554930000
23	C	-12.9632010000	8.5629110000	-4.3979090000
24	C	-11.9988370000	7.5621700000	-3.7193730000
25	C	-7.0945910000	5.9425460000	-4.9091130000
26	C	-6.7611410000	4.6656890000	-4.1232770000
27	C	-7.3473050000	4.6813120000	-2.6969620000
28	C	6.7314760000	9.2084930000	0.2564190000
29	C	5.7447190000	8.3960130000	1.1233970000
30	C	5.2327300000	7.1602900000	0.3550090000

1	C	5.5114850000	9.4006670000	-5.0613430000
2	C	5.4290700000	8.4414540000	-3.8693770000
3	C	5.0830620000	6.9965510000	-4.2855830000
4	C	4.9022480000	6.0562280000	-3.0682940000
5	C	11.8836240000	2.2692550000	-2.7574380000
6	C	11.1662900000	1.0079180000	-3.3194820000
7	C	12.7314710000	2.3143060000	2.4110200000
8	C	11.9580040000	1.0544180000	2.0002280000
9	C	12.6728240000	0.3424490000	0.8411340000
10	C	12.0501800000	-1.0108680000	0.4477860000
11	C	1.9970850000	3.0282100000	0.9705970000
12	C	2.7846100000	2.1376770000	0.0017000000
13	C	1.9331800000	1.7422730000	-1.2145210000
14	C	2.6462100000	1.0243060000	-2.3938320000
15	C	-12.4862300000	6.3017890000	-0.8450850000
16	C	-12.6601690000	6.2317170000	-3.2870550000
17	C	-14.2131240000	-0.7142430000	-0.1807980000
18	C	-15.6722660000	1.2625720000	-0.1748000000
19	C	11.0411410000	-0.1832490000	-2.3265360000
20	C	10.5721760000	-0.9305670000	-0.0195300000
21	C	6.2062960000	5.8557230000	-2.2645930000
22	C	6.3021510000	6.0584500000	0.1648270000
23	C	-12.0475890000	4.1947450000	-2.0913830000
24	C	-6.9551240000	3.4102560000	-1.8880630000
25	C	3.1297750000	-0.4385820000	-2.1538710000
26	C	8.1832320000	1.4285030000	-1.5814850000
27	C	8.7823590000	0.0352970000	-1.3169180000
28	C	6.1514310000	0.5423460000	-2.6828320000
29	C	6.2284320000	2.8479460000	-1.9153620000
30	C	5.5952930000	-0.7597720000	-2.0667690000

1	C	6.6493840000	3.8729650000	-0.8357100000
2	C	-13.3370000000	1.5751440000	-0.5286390000
3	C	-11.1478740000	3.5800090000	-1.0033150000
4	C	-11.8415600000	1.2686760000	-0.3185720000
5	C	-8.8896600000	1.9694890000	-2.4628790000
6	C	-9.5510780000	1.7476660000	-1.0823420000
7	C	-6.7317290000	0.9698650000	-1.7756880000
8	C	-5.2573720000	0.8473740000	-2.1713970000
9	C	4.2019780000	-1.8941830000	-0.4698160000
10	C	2.8963530000	-1.9866630000	0.3280790000
11	C	2.5911840000	-8.0328620000	0.1232310000
12	C	2.4490480000	-6.7067610000	-0.6611710000
13	C	2.2900790000	-5.4618960000	0.2271650000
14	C	2.1302230000	-4.1943620000	-0.6291880000
15	C	0.6853160000	-2.8956540000	0.9337820000
16	C	-0.1628860000	-1.7733400000	0.3062400000
17	C	-1.5910780000	-1.6824210000	0.8120040000
18	C	-2.1200660000	0.1414780000	-0.8008700000
19	C	-3.0037190000	-0.1419970000	-2.0121020000
20	C	-4.7913730000	-0.9782100000	-0.5233360000
21	C	-3.8840940000	-0.7286790000	0.6954960000
22	H	-4.6082340000	-10.8873630000	1.2326410000
23	H	-3.8521460000	-9.3191410000	1.0840620000
24	H	-3.3004410000	-10.6519700000	0.0980330000
25	H	-6.7311210000	-9.9779360000	0.6820530000
26	H	-5.9250080000	-8.3156660000	0.5346090000
27	H	-7.5230190000	-9.7994890000	-1.4738630000
28	H	-6.6803310000	-8.1592440000	-1.6587240000
29	H	-15.8258630000	-8.2960120000	1.3888200000
30	H	-8.6208290000	-21.8356990000	-13.0378490000

1	H	-7.2220210000	-20.7959380000	-12.9141570000
2	H	-7.8138910000	-21.2809750000	-14.4849840000
3	H	-9.8995220000	-20.0854420000	-14.0387690000
4	H	-8.4158620000	-18.9826080000	-13.9075740000
5	H	-10.0480180000	-20.1606270000	-11.7322730000
6	H	-8.5636070000	-19.0588710000	-11.6005160000
7	H	-11.2551960000	-18.4219980000	-12.6646190000
8	H	-9.7709050000	-17.3202850000	-12.5311710000
9	H	-17.5095590000	-12.8213800000	17.2658710000
10	H	-16.1053160000	-11.7838470000	17.3343830000
11	H	-16.1081290000	-13.1821640000	16.2866400000
12	H	-18.3452270000	-11.6694750000	15.5981760000
13	H	-16.8558020000	-10.5690050000	15.6708440000
14	H	-17.2649510000	-13.0295090000	14.0701730000
15	H	-15.7770230000	-11.9269340000	14.1415800000
16	H	-18.4776760000	-11.3264070000	13.0792560000
17	H	-16.9870590000	-10.2272250000	13.1467690000
18	H	-17.4002880000	-12.6895530000	11.5497460000
19	H	-15.9147950000	-11.5832020000	11.6129730000
20	H	-11.4087870000	-18.4958810000	-10.3586210000
21	H	-9.9225530000	-17.3969680000	-10.2237100000
22	H	-12.6994680000	-6.6426690000	0.3333370000
23	H	-12.9598160000	-8.3156060000	-0.4204520000
24	H	-11.5909050000	-5.9689850000	-1.6175190000
25	H	-11.7783820000	-7.6862830000	-2.2886670000
26	H	-10.1503720000	-6.5954840000	-0.2912640000
27	H	-18.1778120000	-7.3747220000	-1.0038060000
28	H	-17.2108350000	-7.5675870000	-2.5730280000
29	H	-19.2250070000	-9.0488490000	-2.1314400000
30	H	-16.4935120000	-10.0128650000	-2.8636930000

1	H	-18.0441130000	-10.9984120000	-3.1066600000
2	H	-18.3157250000	-11.4071140000	1.5197890000
3	H	-16.8447650000	-10.2817260000	1.4528640000
4	H	-16.9275390000	-11.7846590000	-5.0037500000
5	H	-15.4571470000	-10.6685320000	-4.8397090000
6	H	-16.7024160000	-11.7333360000	-7.3067150000
7	H	-15.2200780000	-10.6346530000	-7.1327300000
8	H	-15.5314040000	-13.4645310000	-6.3073610000
9	H	-14.0473140000	-12.3656420000	-6.1504630000
10	H	-15.3266650000	-13.4101940000	-8.6116070000
11	H	-13.8402410000	-12.3147340000	-8.4528240000
12	H	-14.1462860000	-15.1479950000	-7.6395000000
13	H	-12.6588160000	-14.0523300000	-7.4923520000
14	H	-13.9647810000	-15.0855830000	-9.9442920000
15	H	-12.4782170000	-13.9885950000	-9.7978550000
16	H	-12.7726950000	-16.8250980000	-8.9914860000
17	H	-11.2855180000	-15.7279480000	-8.8526880000
18	H	-12.6093940000	-16.7556520000	-11.2973300000
19	H	-11.1233820000	-15.6568110000	-11.1594240000
20	H	-19.2938280000	-10.0229260000	3.0946200000
21	H	-17.8060010000	-8.9192590000	3.0399360000
22	H	-17.9945680000	-11.7124150000	4.0130620000
23	H	-16.5237800000	-10.5851350000	3.9861700000
24	H	-19.0132210000	-10.3296610000	5.5741450000
25	H	-17.5200430000	-9.2319250000	5.5643170000
26	H	-17.7476540000	-12.0278400000	6.5199700000
27	H	-16.2702620000	-10.9090020000	6.5346900000
28	H	-18.7954900000	-10.6522460000	8.0650670000
29	H	-17.3017740000	-9.5555500000	8.0928040000
30	H	-17.5561310000	-12.3544250000	9.0327570000

1	H	-16.0737530000	-11.2430060000	9.0774950000
2	H	-18.6228080000	-10.9858050000	10.5677430000
3	H	-17.1303510000	-9.8883260000	10.6207650000
4	H	27.4389060000	1.8371690000	-1.0674150000
5	H	28.8890450000	2.3112400000	-0.2157000000
6	H	28.9822190000	1.8073440000	-1.8861710000
7	H	28.2080570000	-0.4154870000	-1.2318560000
8	H	29.7461620000	0.0873410000	-0.3284750000
9	H	26.9543700000	-0.0128650000	0.6623870000
10	H	28.4810450000	0.5216870000	1.5669410000
11	H	27.7860240000	-2.1708540000	0.5452660000
12	H	29.3003170000	-1.6326910000	1.4683030000
13	H	26.5059630000	-1.8825380000	2.4172160000
14	H	27.9673740000	-1.2194880000	3.3442240000
15	H	27.5523830000	-3.9694900000	2.3219480000
16	H	28.9417350000	-3.2922650000	3.3445760000
17	H	26.1079400000	-3.6482990000	4.1303540000
18	H	27.5502850000	-3.0859620000	5.1492340000
19	H	26.9833290000	-5.7682320000	4.0204460000
20	H	28.4835190000	-5.2170660000	4.9587140000
21	H	27.2851590000	-5.2766830000	6.9678810000
22	H	25.2054220000	-6.2132500000	7.2231150000
23	H	24.2941750000	-7.4525210000	5.3098120000
24	H	25.0153110000	-6.1549140000	4.2003620000
25	H	23.5115700000	-4.6196910000	4.9378560000
26	H	22.8811460000	-5.7970710000	6.2227850000
27	H	22.5211310000	-5.8618320000	3.2821650000
28	H	21.9515470000	-7.1063020000	4.5317920000
29	H	21.0631110000	-4.3134680000	4.1034360000
30	H	20.5709420000	-5.4592750000	5.4744190000

1	H	19.9582600000	-5.6103970000	2.5803820000
2	H	19.5541880000	-6.8539480000	3.8937770000
3	H	18.5464200000	-4.0842240000	3.5776790000
4	H	18.1614980000	-5.3048140000	4.9180880000
5	H	33.5401210000	1.6495200000	-3.0021630000
6	H	34.4884570000	0.4937010000	-3.9064910000
7	H	34.2150150000	0.2530110000	-2.1975840000
8	H	31.7025100000	0.5134480000	-3.3747930000
9	H	32.7083730000	-0.7124830000	-4.3339780000
10	H	32.0339190000	-0.7225550000	-1.4480320000
11	H	33.0388840000	-1.9487930000	-2.4077630000
12	H	30.1474420000	-1.3478960000	-2.6534970000
13	H	31.1600310000	-2.6063380000	-3.5621710000
14	H	30.5062310000	-2.5374810000	-0.6730960000
15	H	31.4124590000	-3.8298640000	-1.6442780000
16	H	28.5508240000	-3.0756310000	-1.7947140000
17	H	29.4606170000	-4.3830210000	-2.7421890000
18	H	28.9143020000	-4.2880210000	0.1685810000
19	H	29.7228030000	-5.6214660000	-0.8328940000
20	H	26.9016130000	-4.7188740000	-0.9478050000
21	H	27.7256620000	-6.1233950000	-1.8326580000
22	H	27.8763930000	-7.3116400000	0.2801170000
23	H	26.1953650000	-7.0122470000	1.8276950000
24	H	25.7627030000	-4.3085520000	0.5623640000
25	H	25.1232440000	-4.9364000000	2.1845840000
26	H	24.2198450000	-5.5996170000	-0.5593530000
27	H	23.5993810000	-6.2747220000	1.0512240000
28	H	23.3674120000	-3.5264760000	-0.0366160000
29	H	22.7925610000	-4.1631290000	1.6062220000
30	H	21.8319700000	-4.8730070000	-1.1056840000

1	H	21.2714020000	-5.5434210000	0.5286380000
2	H	20.9152960000	-2.8326660000	-0.6144240000
3	H	20.4384410000	-3.4282720000	1.0745300000
4	H	19.3857150000	-4.2863620000	-1.5592530000
5	H	18.9093560000	-4.8830850000	0.1294460000
6	H	18.3772400000	-2.2958450000	-1.2039940000
7	H	18.0997670000	-2.7007820000	0.5831080000
8	H	17.4503230000	-5.4313340000	2.0464770000
9	H	17.0472400000	-6.6315500000	3.3998820000
10	H	14.3565390000	-4.2741190000	0.1360640000
11	H	15.2156590000	-5.6694780000	-0.7297210000
12	H	15.7084140000	-6.6178610000	1.3900060000
13	H	12.8166070000	-5.9105600000	1.0547620000
14	H	13.4964680000	-7.2937370000	2.0839900000
15	H	10.1119170000	-10.2176790000	-2.1147150000
16	H	11.1802560000	-9.3193410000	-1.2671850000
17	H	9.1247750000	-9.7522600000	0.4592310000
18	H	9.5923240000	-11.3577960000	-0.3397380000
19	H	10.9174300000	-9.8054790000	1.6923360000
20	H	11.2993460000	-11.4833850000	1.0042430000
21	H	10.6888810000	-7.9658930000	1.2449770000
22	H	-12.3644310000	11.8639670000	6.4340500000
23	H	-14.0996240000	12.3477370000	6.6164190000
24	H	-13.0455540000	12.1897830000	8.0732810000
25	H	-12.8244300000	9.7450590000	7.6875410000
26	H	-14.5564230000	10.2286340000	7.8706630000
27	H	-13.0843590000	9.7597210000	5.1912380000
28	H	-14.8163230000	10.2450630000	5.3778600000
29	H	-13.5487790000	7.6401570000	6.4553830000
30	H	-15.2815970000	8.1293400000	6.6322070000

1	H	-13.7836380000	7.6729420000	3.9754020000
2	H	-15.5211290000	8.1750590000	4.1377870000
3	H	-14.2442920000	5.5628720000	5.1660560000
4	H	-15.9795380000	6.0291650000	5.3753280000
5	H	-14.5417460000	5.6809420000	2.6578460000
6	H	-16.2854640000	6.0973850000	2.8999350000
7	H	-14.8553120000	3.5393850000	3.8595070000
8	H	-16.6018950000	3.9159950000	4.1337800000
9	H	-15.2492930000	3.6295280000	1.3763090000
10	H	-17.0095540000	3.9303390000	1.6731360000
11	H	-20.7706640000	1.5048900000	10.4075840000
12	H	-19.3073210000	1.7531870000	11.4448290000
13	H	-20.5737020000	0.5642020000	11.9346590000
14	H	-20.0545200000	-0.8511830000	9.9621480000
15	H	-18.5924510000	-0.6060800000	10.9961410000
16	H	-19.2711650000	0.9670670000	8.4225650000
17	H	-17.8039050000	1.2097490000	9.4516150000
18	H	-18.5756330000	-1.3977060000	7.9841040000
19	H	-17.0999520000	-1.1579290000	9.0023210000
20	H	-17.8209740000	0.4318440000	6.4552140000
21	H	-16.3322560000	0.6626790000	7.4548470000
22	H	-17.1475970000	-1.9312120000	5.9872300000
23	H	-15.6386840000	-1.7030640000	6.9587930000
24	H	-16.4565980000	-0.0816960000	4.4669370000
25	H	-14.9212430000	0.1315800000	5.3966700000
26	H	-15.8258330000	-2.4292640000	3.9240990000
27	H	-14.2603550000	-2.2313770000	4.8088720000
28	H	-15.2199820000	-0.5715140000	2.4239710000
29	H	-13.6153610000	-0.3727000000	3.2373390000
30	H	-18.4945790000	17.3239710000	3.1195440000

1	H	-19.8429190000	16.1162540000	3.2110210000
2	H	-19.3599050000	17.0487680000	4.6781950000
3	H	-17.1433730000	15.9330710000	4.6985850000
4	H	-18.4884630000	14.7302630000	4.7931120000
5	H	-16.9297350000	15.4992120000	2.2325050000
6	H	-18.2778060000	14.2965100000	2.3307470000
7	H	-15.5900280000	14.1296600000	3.8481730000
8	H	-16.9413470000	12.9330810000	3.9517350000
9	H	-15.3656100000	13.6595300000	1.3917720000
10	H	-16.7254570000	12.4715800000	1.5005610000
11	H	-14.0505900000	12.3012480000	3.0439490000
12	H	-15.4235940000	11.1301480000	3.1609740000
13	H	-13.8162010000	11.7592230000	0.5967350000
14	H	-15.2049760000	10.6086370000	0.7335570000
15	H	-20.4715600000	17.3680620000	-0.2477140000
16	H	-20.5784400000	15.7311330000	0.5065890000
17	H	-19.0838320000	16.7322330000	0.7127440000
18	H	-18.6416840000	16.5948430000	-1.7386520000
19	H	-20.1330140000	15.5973570000	-1.9598860000
20	H	-17.6928520000	14.8317460000	-0.2243630000
21	H	-19.1794430000	13.8299110000	-0.4601190000
22	H	-17.2138290000	14.7241940000	-2.6709550000
23	H	-18.6948280000	13.7173700000	-2.9302020000
24	H	-16.3086250000	12.9796150000	-1.1191050000
25	H	-17.7704580000	11.9576540000	-1.4118550000
26	H	-15.7287090000	12.8813800000	-3.5408320000
27	H	-17.1708740000	11.8363150000	-3.8643880000
28	H	-14.8780930000	11.1929350000	-1.9090320000
29	H	-16.2754790000	10.1117360000	-2.2846340000
30	H	-5.1495680000	10.5816260000	-14.7288810000

1	H	-5.2346050000	11.5686330000	-13.2204630000
2	H	-6.7345650000	10.8120750000	-13.8969850000
3	H	-5.7792840000	8.5382060000	-13.4665170000
4	H	-4.2825790000	9.2938250000	-12.7922320000
5	H	-7.1444280000	9.6349620000	-11.6664620000
6	H	-5.6456030000	10.3921430000	-10.9937920000
7	H	-6.1780270000	7.3593620000	-11.2572090000
8	H	-4.6779470000	8.1173660000	-10.5897080000
9	H	-7.5327930000	8.4536620000	-9.4514600000
10	H	-6.0311030000	9.2151130000	-8.7898660000
11	H	-6.5547990000	6.1811780000	-9.0534610000
12	H	-5.0501600000	6.9439680000	-8.4022630000
13	H	-7.8910850000	7.2616780000	-7.2238240000
14	H	-6.3841250000	8.0302440000	-6.5821630000
15	H	4.2388940000	8.1773110000	8.0504280000
16	H	3.6771650000	6.7696510000	9.0416520000
17	H	2.7847750000	8.3367870000	9.1071970000
18	H	2.1143590000	8.0921960000	6.7289030000
19	H	1.5534670000	6.6873890000	7.7178100000
20	H	4.0840980000	6.7518830000	5.9404160000
21	H	3.5225790000	5.3451540000	6.9290990000
22	H	1.9506630000	6.6828340000	4.6291770000
23	H	1.3882300000	5.2744800000	5.6152110000
24	H	3.9228030000	5.3509980000	3.8499010000
25	H	3.3595970000	3.9389050000	4.8301590000
26	H	1.7946310000	5.2897090000	2.5345610000
27	H	1.2298530000	3.8699060000	3.5029280000
28	H	3.7802770000	3.9746100000	1.7652500000
29	H	3.2091330000	2.5460630000	2.7105840000
30	H	11.6527870000	9.1417520000	8.2689180000

1	H	13.2982230000	9.5740780000	8.8707470000
2	H	12.5316300000	8.0256450000	9.3914810000
3	H	13.4132450000	8.7673180000	6.5267840000
4	H	14.2868830000	7.6541950000	7.6483010000
5	H	11.4147770000	7.2427300000	6.5719320000
6	H	12.2926480000	6.1350820000	7.7001350000
7	H	13.1871990000	6.8777030000	4.8374850000
8	H	14.0696610000	5.7822650000	5.9713740000
9	H	11.2043710000	5.3334120000	4.8989940000
10	H	12.0935240000	4.2530620000	6.0451640000
11	H	12.9816220000	4.9614990000	3.1725760000
12	H	13.8848440000	3.9076910000	4.3281520000
13	H	11.0320620000	3.3665100000	3.2648070000
14	H	11.9507700000	2.3360310000	4.4340150000
15	H	11.4748470000	11.3315180000	5.5156850000
16	H	10.7464850000	9.7253250000	5.8939940000
17	H	9.9130730000	10.8511970000	4.7471460000
18	H	11.8346930000	10.6456520000	3.1529200000
19	H	12.6659640000	9.5265310000	4.3007130000
20	H	9.9282240000	9.0262440000	2.9497630000
21	H	10.7517740000	7.9140580000	4.1141480000
22	H	11.8773820000	8.8138510000	1.3788710000
23	H	12.6983330000	7.7145790000	2.5558600000
24	H	9.9796890000	7.1902010000	1.1840230000
25	H	10.7834850000	6.1078290000	2.3881420000
26	H	11.9553650000	6.9291320000	-0.3531450000
27	H	12.7574520000	5.8672280000	0.8705280000
28	H	10.0587920000	5.3031020000	-0.5185210000
29	H	10.8388150000	4.2648230000	0.7356020000
30	H	12.0429060000	4.9641850000	-2.0260520000

1	H	12.8335440000	3.9492460000	-0.7552620000
2	H	10.1375680000	3.3750830000	-2.1079230000
3	H	10.9172980000	2.3824700000	-0.8072370000
4	H	8.4694210000	17.9115360000	-5.7820030000
5	H	7.5908260000	18.7148490000	-7.1384570000
6	H	6.7248120000	18.3587470000	-5.5957780000
7	H	7.8589050000	16.2868490000	-7.5864280000
8	H	6.1170990000	16.7323200000	-7.4011910000
9	H	7.9092240000	15.4948010000	-5.2053920000
10	H	6.1656320000	15.9375600000	-5.0218740000
11	H	7.3081140000	13.8744660000	-7.0206980000
12	H	5.5627840000	14.3135590000	-6.8377760000
13	H	7.3646000000	13.1117190000	-4.6359310000
14	H	5.6179630000	13.5418450000	-4.4560370000
15	H	6.7777230000	11.4628740000	-6.4296930000
16	H	5.0270840000	11.8836180000	-6.2486580000
17	H	6.8551940000	10.7580770000	-4.0307260000
18	H	5.1026110000	11.1603210000	-3.8524600000
19	H	10.5787880000	19.2067510000	-2.0968080000
20	H	9.8334690000	18.0763400000	-3.2895180000
21	H	11.4782540000	17.7304880000	-2.6156090000
22	H	10.4811110000	17.4588950000	-0.3311590000
23	H	8.8418600000	17.8060820000	-1.0090480000
24	H	10.6685610000	15.4419100000	-1.8138780000
25	H	9.0340520000	15.7996600000	-2.5002910000
26	H	9.6455910000	15.1711250000	0.4683630000
27	H	8.0192660000	15.5492740000	-0.2281620000
28	H	9.8208620000	13.1695340000	-1.0359420000
29	H	8.2149300000	13.5826730000	-1.7559880000
30	H	8.7180370000	12.8503870000	1.2115330000

1	H	7.1319460000	13.3187700000	0.4775300000
2	H	8.8520360000	10.8892890000	-0.3605860000
3	H	7.3114460000	11.4294680000	-1.1342920000
4	H	-14.1208210000	11.0851570000	-4.2768100000
5	H	-15.4729750000	9.9521950000	-4.6849060000
6	H	-12.5324740000	10.4096480000	2.2819830000
7	H	-13.9470480000	9.2984590000	2.4437640000
8	H	-6.8907010000	4.9948050000	-6.8535990000
9	H	-5.3801280000	5.7641210000	-6.2245730000
10	H	7.6009680000	10.4883190000	1.8035860000
11	H	6.0991000000	11.1286530000	1.0227560000
12	H	-18.6546040000	1.3229110000	1.0371860000
13	H	-15.3695170000	1.5252710000	2.6432730000
14	H	-17.1148110000	1.7724540000	2.9886670000
15	H	-16.8618300000	0.0975820000	1.1786530000
16	H	-11.9475600000	-2.9413800000	0.2743830000
17	H	-14.7288150000	-2.8241450000	1.7265560000
18	H	-13.1213210000	-2.7441060000	2.5348850000
19	H	-12.3760620000	-0.9708070000	0.9964330000
20	H	-12.3538810000	9.7686780000	-0.1433750000
21	H	-13.7928400000	8.6982230000	0.0665750000
22	H	-10.7416370000	5.7647820000	1.1193480000
23	H	-11.0989840000	8.4614770000	1.5653340000
24	H	-12.4845600000	7.3285010000	1.7756120000
25	H	-10.9215390000	7.8377560000	-0.8174560000
26	H	-13.3465350000	9.5230050000	-2.5052570000
27	H	-14.6238740000	8.3421490000	-2.9687110000
28	H	-12.3499140000	9.4145490000	-4.7672060000
29	H	-13.4885960000	8.1057170000	-5.2638740000
30	H	-11.2037250000	6.9691730000	-5.3968460000

1	H	-11.6293000000	8.1055760000	-2.8330590000
2	H	-8.1988410000	6.0501350000	-4.9887030000
3	H	-6.6853290000	6.8281780000	-4.3758560000
4	H	-7.1743700000	3.8104850000	-4.6951910000
5	H	-5.6558590000	4.5556430000	-4.0595570000
6	H	-7.4763700000	6.0019380000	-1.2763460000
7	H	-8.4498920000	4.7876410000	-2.7854210000
8	H	7.6751610000	8.6499920000	0.0880880000
9	H	6.2470260000	9.3823100000	-0.7307480000
10	H	6.1854680000	8.1198760000	2.1056280000
11	H	4.8613600000	9.0470840000	1.3080580000
12	H	3.5866570000	6.1305720000	0.3411830000
13	H	4.8996660000	7.5732680000	-0.6201730000
14	H	6.2904030000	9.0421620000	-5.7696300000
15	H	4.5320370000	9.4313750000	-5.5869920000
16	H	6.4126970000	8.4746010000	-3.3628550000
17	H	4.6499110000	8.8107330000	-3.1662350000
18	H	3.4595680000	4.8732600000	-3.6177020000
19	H	5.8620770000	6.5933610000	-4.9687140000
20	H	4.1173330000	7.0266510000	-4.8364490000
21	H	4.1382610000	6.5131160000	-2.3998840000
22	H	12.7991920000	0.3458330000	-4.1783560000
23	H	12.0412130000	2.9742680000	-3.6035790000
24	H	12.8824130000	1.9774840000	-2.3656070000
25	H	10.1739710000	1.2958820000	-3.6983260000
26	H	12.8128550000	3.0049520000	1.5449860000
27	H	13.7572620000	2.0167410000	2.7228120000
28	H	10.9269120000	1.3335690000	1.6939930000
29	H	11.9032640000	0.3711290000	2.8755950000
30	H	12.0828190000	-2.8105240000	1.1862340000

1	H	13.7255510000	0.1429890000	1.1413320000
2	H	12.7010180000	1.0131480000	-0.0417480000
3	H	12.6943720000	-1.4208550000	-0.3597870000
4	H	1.6582020000	3.9402990000	0.4308330000
5	H	1.1042320000	2.4800470000	1.3447330000
6	H	3.6652350000	2.7114410000	-0.3375590000
7	H	3.1125160000	1.2501430000	0.5624330000
8	H	1.5400870000	2.6940890000	-1.6382940000
9	H	1.0630750000	1.1416030000	-0.8785750000
10	H	2.2064400000	0.9683340000	-4.2932310000
11	H	3.4708020000	1.6775710000	-2.7182360000
12	H	-13.3291140000	6.9773050000	-1.0757250000
13	H	-12.9240540000	5.5564020000	-0.1455890000
14	H	-12.6478390000	5.5464500000	-4.1643740000
15	H	-13.7416640000	6.3394120000	-3.0633780000
16	H	-13.8466450000	-0.7647230000	-1.2319990000
17	H	-15.2003640000	-1.2289530000	-0.1990320000
18	H	-15.6361070000	2.3402860000	-0.4540390000
19	H	-16.0546710000	0.7534650000	-1.0897730000
20	H	10.6306160000	-1.0709340000	-2.8578750000
21	H	12.0893070000	-0.4257240000	-2.0877480000
22	H	9.9830940000	-0.6747050000	0.8893970000
23	H	10.2514960000	-1.9462960000	-0.3460950000
24	H	6.6660040000	6.8427780000	-2.0780980000
25	H	6.9645300000	5.3357390000	-2.8880410000
26	H	7.3126750000	6.4791200000	-0.0256070000
27	H	6.3927220000	5.5099880000	1.1298070000
28	H	-11.6518910000	3.8222980000	-3.0626350000
29	H	-13.1023860000	3.8631530000	-2.0114190000
30	H	-5.8477850000	3.4318170000	-1.8064350000

1	H	-7.3286630000	3.5233320000	-0.8496050000
2	H	3.3247870000	-0.9111120000	-3.1423450000
3	H	2.2295060000	-0.9699190000	-1.7851740000
4	H	8.5213120000	2.0410890000	-0.7228370000
5	H	8.5778460000	1.8577450000	-2.5120630000
6	H	8.2474020000	-0.3061330000	-0.4012900000
7	H	8.5334470000	-0.6907030000	-2.1187420000
8	H	6.9484340000	0.2774000000	-3.4127300000
9	H	5.3779550000	0.9999210000	-3.3258660000
10	H	6.5658570000	3.1717040000	-2.9232070000
11	H	5.1290080000	2.8466780000	-1.9240460000
12	H	5.4430980000	-1.4930440000	-2.8911620000
13	H	6.3996200000	-1.1916420000	-1.4387890000
14	H	7.7461870000	4.0504180000	-0.8583000000
15	H	6.3783560000	3.4250820000	0.1465220000
16	H	-13.5416020000	1.5188860000	-1.6237310000
17	H	-13.4811600000	2.6221810000	-0.1898380000
18	H	-10.1727600000	4.1027300000	-1.1291400000
19	H	-11.4887480000	3.8141110000	0.0259190000
20	H	-11.6628220000	0.2210250000	-0.6297040000
21	H	-11.5688470000	1.3957460000	0.7526820000
22	H	-9.2035550000	1.1276570000	-3.1209180000
23	H	-9.3234410000	2.8603700000	-2.9516470000
24	H	-9.0815890000	2.3467980000	-0.2736540000
25	H	-9.3964950000	0.6827090000	-0.7986360000
26	H	5.8090320000	-8.8274670000	-0.1458150000
27	H	4.9621480000	-6.2848860000	2.1716240000
28	H	3.6870160000	-8.6563740000	-2.8872700000
29	H	1.9079330000	-11.1671150000	0.0785120000
30	H	0.6816260000	-8.2713060000	0.4367340000

H	1.5455490000	-6.7783070000	-1.3062700000
H	3.3338850000	-6.5791460000	-1.3229110000
H	1.4103250000	-5.6030260000	0.8869360000
H	3.1877390000	-5.3276300000	0.8599940000
H	1.2518730000	-4.2826500000	-1.3059200000
H	2.9976880000	-4.1572440000	-1.2950290000
H	0.0901140000	-3.8258460000	0.8082880000
H	0.8486880000	-2.7454030000	2.0232080000
H	-0.2168050000	-1.9908490000	-0.7832560000
H	0.3332920000	-0.7945520000	0.4611310000
H	-1.0644520000	0.0764570000	-1.1192620000
H	-2.2914420000	1.1840660000	-0.4533700000
H	-2.7420930000	0.5744680000	-2.8194520000
H	-2.7912070000	-1.1647480000	-2.3943230000
H	-4.6424520000	-2.0231200000	-0.8737390000
H	-5.8517570000	-0.8808840000	-0.2194770000
H	-4.0945400000	0.2858020000	1.0997580000
H	-4.1253940000	-1.4654910000	1.4910590000
H	-7.1375520000	2.0534140000	-3.4686610000
H	-7.2183470000	0.0066960000	-2.0476850000
H	-6.8154550000	1.1099520000	-0.6750980000
H	4.4466560000	0.1473860000	-0.5984190000
H	4.3791860000	-2.7501010000	-1.1332920000
H	5.0286240000	-1.9348440000	0.2756210000

6.9. Piperazine amine core based bone-targeting ionizable lipids 'Type 3'

Type3-P1-C12

P	-5.5601300000	7.8608920000	2.0453350000
P	18.7722730000	4.0665820000	1.2418330000
P	2.0702150000	4.5793800000	-1.0419950000

1	P	3.1329070000	4.8282170000	1.7949680000
2	O	-9.9445720000	7.5307610000	-2.6865870000
3	O	-10.3396880000	6.4348880000	-0.7279200000
4	O	-10.6428610000	2.9281250000	1.3247950000
5	O	-9.2029670000	4.6353780000	1.0895190000
6	O	-4.2514980000	6.7272190000	1.9099750000
7	O	-7.0343820000	7.4590810000	1.6288350000
8	O	-5.2045680000	8.3230260000	3.5684050000
9	O	-4.8875730000	8.9359030000	1.2678840000
10	O	9.0358400000	8.4934060000	0.3157470000
11	O	13.2114090000	6.4589500000	-1.5263710000
12	O	18.4028410000	4.3687130000	2.9134270000
13	O	19.3698160000	2.7043100000	1.2073470000
14	O	17.5225840000	4.2843550000	0.2562820000
15	O	19.9235510000	5.2221800000	1.1332430000
16	O	21.9316720000	6.8044840000	1.9084780000
17	O	22.5807060000	4.9582760000	3.0833450000
18	O	22.0405080000	6.7790400000	-1.6721270000
19	O	22.0876550000	7.0433440000	-3.9087430000
20	O	12.0009020000	6.4496470000	0.3760650000
21	O	16.1203570000	-3.8024290000	-2.7996230000
22	O	-23.6045770000	-4.4962420000	-3.9066450000
23	O	-19.2097780000	-5.3355310000	-7.7165570000
24	O	-19.7018660000	1.3864760000	-1.8859730000
25	O	19.4169830000	-2.2501720000	-0.0436740000
26	O	14.3331220000	-5.7466690000	5.0725400000
27	O	-11.2073650000	-3.4682780000	2.6049150000
28	O	1.9062860000	-6.9387270000	1.7167650000
29	O	-6.3888830000	-1.7257260000	2.5023960000
30	O	1.8162870000	-3.3459590000	-1.3523820000

1	O	1.4257170000	6.1915120000	-1.0436690000
2	O	3.6027700000	4.6658040000	-1.8552850000
3	O	1.1451330000	3.8353140000	-2.0420820000
4	O	4.8062300000	4.9582050000	1.3516830000
5	O	2.5568000000	6.4468560000	2.0544000000
6	O	3.1844460000	4.2164480000	3.2212590000
7	O	0.7856430000	3.6836110000	1.1600040000
8	O	-1.9257820000	-0.7865370000	-2.6320700000
9	N	-3.1147480000	8.8056020000	2.4017460000
10	N	14.0345750000	5.3831260000	0.2788660000
11	N	-20.0666000000	-3.4893990000	-4.5786590000
12	N	-18.5291390000	-1.2136720000	-1.9703620000
13	N	-14.7512830000	-1.0576540000	-1.6155730000
14	N	17.5784080000	-4.5500470000	-0.1899450000
15	N	14.1937080000	-5.4414190000	1.3507030000
16	N	10.4285600000	-5.9009940000	0.8432420000
17	N	7.5521000000	-5.5202610000	0.8700460000
18	N	-12.0726570000	-0.9053190000	-0.5339650000
19	N	-8.9453270000	-1.6142980000	1.5835280000
20	N	3.9970190000	-4.2085960000	0.1292820000
21	N	1.8670340000	-1.1559260000	-0.8282760000
22	N	-3.0963750000	-1.5983260000	-0.8877780000
23	N	-5.2879990000	-0.9874160000	0.6936750000
24	C	-22.9342470000	7.5608720000	-5.7212770000
25	C	-22.9401970000	8.0279960000	-4.2604860000
26	C	-22.4865260000	6.9210680000	-3.2952570000
27	C	-22.5236260000	7.3991710000	-1.8356300000
28	C	-22.0588460000	6.3196760000	-0.8457660000
29	C	-22.2050960000	6.7908330000	0.6100910000
30	C	-21.6608210000	5.7624490000	1.6136110000

1	C	-21.7255830000	6.2877490000	3.0553820000
2	C	-21.2581290000	5.2381850000	4.0313310000
3	C	-20.0110560000	5.0733320000	4.5009710000
4	C	-18.7871820000	5.8898250000	4.1616040000
5	C	-17.7242980000	5.0100530000	3.4834330000
6	C	-16.3884960000	5.7431970000	3.2723140000
7	C	-15.3284360000	4.8116200000	2.6608130000
8	C	-13.9414110000	5.4676570000	2.5558830000
9	C	-12.8863430000	4.4715590000	2.0432860000
10	C	-27.3878870000	11.2981190000	-5.8073370000
11	C	-25.9465240000	11.6236510000	-5.3943650000
12	C	-25.6760230000	11.2561420000	-3.9268530000
13	C	-24.2323950000	11.5805530000	-3.5109240000
14	C	-23.9480180000	11.1704310000	-2.0569480000
15	C	-22.4789090000	11.4047130000	-1.6712960000
16	C	-22.1863140000	10.9754470000	-0.2244280000
17	C	-20.6983580000	11.1281250000	0.1261630000
18	C	-20.4132270000	10.6060490000	1.5124750000
19	C	-19.6876990000	9.5200960000	1.8255120000
20	C	-19.0022660000	8.5861370000	0.8585430000
21	C	-17.5191760000	8.9515570000	0.7184430000
22	C	-16.7783960000	7.9953410000	-0.2298640000
23	C	-15.2855460000	8.3462870000	-0.3160550000
24	C	-14.5097830000	7.3897940000	-1.2355590000
25	C	-13.0073960000	7.7102670000	-1.2235490000
26	C	-12.1950180000	6.7737950000	-2.1314380000
27	C	-10.7019950000	6.9725580000	-1.9257690000
28	C	-11.4780390000	5.0881990000	1.9552330000
29	C	-10.4241670000	4.1180760000	1.4264970000
30	C	-9.0068520000	6.5616700000	-0.3165540000

1	C	-8.8644870000	5.9946210000	1.0980720000
2	C	-7.4130710000	6.1068960000	1.5760550000
3	C	-2.8539280000	8.0743920000	3.7134450000
4	C	-4.2144440000	7.7378450000	4.3609810000
5	C	8.0008580000	8.7949130000	1.2271680000
6	C	9.9964570000	7.6278420000	0.8700090000
7	C	11.0687590000	7.3454680000	-0.1719600000
8	C	15.5522130000	21.2993720000	-9.5601610000
9	C	16.2989150000	19.9690080000	-9.7243770000
10	C	16.4416300000	19.2241830000	-8.3874680000
11	C	17.1885180000	17.8903030000	-8.5494110000
12	C	17.8377270000	5.9820700000	19.0712280000
13	C	18.4859360000	5.2766620000	17.8725070000
14	C	18.3459760000	6.0978770000	16.5807870000
15	C	18.9966660000	5.3939810000	15.3788760000
16	C	18.8618350000	6.2156500000	14.0863130000
17	C	17.3334530000	17.1442550000	-7.2128250000
18	C	16.3275940000	4.7981280000	0.7856520000
19	C	15.2885130000	4.9299670000	-0.3266040000
20	C	21.1596990000	4.9528330000	0.5241800000
21	C	22.0463000000	6.1971610000	0.6474050000
22	C	21.6844190000	7.2519860000	-0.4003670000
23	C	22.0315150000	6.0355240000	3.0307190000
24	C	21.2982090000	6.6995250000	4.1855540000
25	C	21.7551840000	7.4631550000	-2.8196340000
26	C	20.9988600000	8.7771020000	-2.6458230000
27	C	20.8018860000	9.5487550000	-3.9633120000
28	C	20.0531040000	10.8763410000	-3.7570630000
29	C	19.8772920000	11.6388150000	-5.0806760000
30	C	19.1332590000	12.9707580000	-4.8918410000

1	C	18.9726140000	13.7261560000	-6.2215150000
2	C	18.2284830000	15.0598250000	-6.0456980000
3	C	18.0783260000	15.8097410000	-7.3796420000
4	C	21.3294080000	5.8742520000	5.4815160000
5	C	20.6013230000	6.5773930000	6.6389110000
6	C	20.6688630000	5.7523140000	7.9346230000
7	C	19.9721570000	6.4552100000	9.1110520000
8	C	20.0717390000	5.6312470000	10.4054950000
9	C	19.3985830000	6.3345070000	11.5952200000
10	C	19.5202360000	5.5118250000	12.8885490000
11	C	13.0878840000	6.1145210000	-0.3745240000
12	C	-10.0811110000	-8.9314250000	0.7836840000
13	C	-11.5242480000	-9.1884010000	0.3610490000
14	C	-11.9543230000	-8.2307530000	-0.7565490000
15	C	-13.4031080000	-8.4995590000	-1.1808520000
16	C	-13.8438930000	-7.5350920000	-2.2888710000
17	C	-15.2973430000	-7.8006200000	-2.6996560000
18	C	-15.7563180000	-6.8139700000	-3.7799270000
19	C	-21.3577390000	-14.4582690000	3.9051240000
20	C	-20.9224820000	-13.0148340000	3.6683190000
21	C	-21.6286790000	-12.4183130000	2.4458040000
22	C	-21.1832160000	-10.9710310000	2.2029970000
23	C	-21.8795080000	-10.3845060000	0.9697040000
24	C	-21.4170770000	-8.9469720000	0.7011850000
25	C	-22.0882310000	-8.3881200000	-0.5582820000
26	C	-29.8222970000	-6.3734030000	3.3746720000
27	C	-29.3638450000	-5.5171710000	2.1971480000
28	C	-27.9150270000	-5.0545580000	2.3837120000
29	C	-27.4523360000	-4.1863680000	1.2064220000
30	C	-26.0071160000	-3.7216690000	1.4146830000

1	C	-25.5255720000	-2.8313380000	0.2612890000
2	C	-24.0895070000	-2.3654910000	0.5200090000
3	C	-11.4380580000	-13.2895600000	10.8664250000
4	C	-10.7736870000	-12.8261470000	9.5732620000
5	C	-11.2102320000	-11.4034190000	9.2060020000
6	C	-10.5353570000	-10.9388180000	7.9100300000
7	C	-10.9619140000	-9.5110750000	7.5495430000
8	C	-10.2706230000	-9.0376740000	6.2654910000
9	C	-10.6735980000	-7.5984680000	5.9244760000
10	C	7.8839040000	-11.1440180000	-9.8806460000
11	C	6.6068680000	-11.0147270000	-9.0547890000
12	C	6.8469910000	-10.1751850000	-7.7952420000
13	C	5.5653570000	-10.0479240000	-6.9619410000
14	C	5.8170950000	-9.2124350000	-5.7017420000
15	C	4.5475100000	-9.0856460000	-4.8496890000
16	C	4.8284150000	-8.2609010000	-3.5889140000
17	C	28.0241520000	-8.9272360000	-7.5173790000
18	C	26.5709590000	-8.6529930000	-7.8956460000
19	C	25.8442580000	-7.9035220000	-6.7745100000
20	C	24.3777720000	-7.6362080000	-7.1403860000
21	C	23.6705750000	-6.8987280000	-5.9997770000
22	C	22.1874800000	-6.6422820000	-6.3079110000
23	C	21.5323000000	-5.9413220000	-5.1151170000
24	C	30.3698500000	-9.5184860000	-3.7787650000
25	C	29.8140920000	-8.3921770000	-2.9123080000
26	C	28.2822190000	-8.4278740000	-2.8734560000
27	C	27.7282650000	-7.2894680000	-2.0080010000
28	C	26.1962060000	-7.3184890000	-1.9788330000
29	C	25.6352380000	-6.1588710000	-1.1452940000
30	C	24.1031540000	-6.1703360000	-1.1649920000

1	C	18.8254210000	-15.7234440000	-3.3361940000
2	C	19.0260630000	-14.8794510000	-2.0808280000
3	C	17.8814610000	-13.8757840000	-1.9001020000
4	C	18.0874480000	-13.0268840000	-0.6397560000
5	C	16.9455450000	-12.0188600000	-0.4639760000
6	C	17.1542090000	-11.1605160000	0.7895630000
7	C	16.0192530000	-10.1426340000	0.9507530000
8	C	-21.5918780000	-6.9722260000	-0.8762250000
9	C	-9.9507990000	-7.1080120000	4.6647040000
10	C	23.5183980000	-4.9656180000	-0.4133220000
11	C	16.2339020000	-9.2638310000	2.1894170000
12	C	20.0241350000	-5.7006640000	-5.3031390000
13	C	19.4694150000	-5.0707430000	-4.0214800000
14	C	17.9418740000	-4.8320930000	-4.0348360000
15	C	-22.2169500000	-6.4644800000	-2.1802440000
16	C	-21.6629660000	-5.0881540000	-2.5713540000
17	C	-22.2070120000	-4.6464840000	-3.9479340000
18	C	-17.2240070000	-7.0549860000	-4.1540510000
19	C	-17.7103130000	-6.0228520000	-5.1790710000
20	C	-19.2064820000	-6.2171740000	-5.4795160000
21	C	-19.8057130000	-5.1768570000	-6.4544030000
22	C	-19.6180970000	-3.7189250000	-5.9801010000
23	C	-21.5470980000	-3.3504710000	-4.4629250000
24	C	-23.5696110000	-1.4437000000	-0.5912230000
25	C	-22.1435370000	-0.9928670000	-0.2611450000
26	C	-21.5619430000	-0.0480200000	-1.3282480000
27	C	-20.1564170000	0.4577230000	-0.9350650000
28	C	-19.0545190000	-0.6119700000	-0.7153700000
29	C	17.4594630000	-4.2118890000	-2.6953290000
30	C	21.9918620000	-4.9484430000	-0.5491370000

1	C	21.3680750000	-3.6682680000	0.0411850000
2	C	19.8788210000	-3.5444530000	-0.3370210000
3	C	17.5671480000	-5.2223610000	-1.5242950000
4	C	18.9605190000	-4.5570180000	0.3780880000
5	C	15.1144380000	-8.2243590000	2.3219630000
6	C	15.3406930000	-7.3219090000	3.5494850000
7	C	14.2477040000	-6.2428870000	3.7591370000
8	C	14.3034340000	-5.0379070000	2.7777290000
9	C	-19.3536490000	-2.3216990000	-4.0071150000
10	C	-19.3576610000	-2.3386570000	-2.4694110000
11	C	-17.1351590000	-1.6858770000	-1.7632940000
12	C	-16.1090860000	-0.5460290000	-1.9023100000
13	C	-13.1308310000	-1.7105180000	0.1175140000
14	C	-14.5039640000	-1.1049610000	-0.1586690000
15	C	-13.7106560000	-0.2358010000	-2.2608890000
16	C	-12.3258980000	-0.8448040000	-1.9880600000
17	C	16.6438500000	-5.2209720000	0.7439240000
18	C	15.1908680000	-4.7572580000	0.4845670000
19	C	12.8339260000	-5.2047540000	0.8007720000
20	C	11.7425010000	-6.0253280000	1.5134960000
21	C	8.4665160000	-4.4778650000	0.3540570000
22	C	9.8247280000	-4.5679960000	1.0583050000
23	C	9.5127870000	-6.9396420000	1.3549440000
24	C	8.1596820000	-6.8488400000	0.6459070000
25	C	-10.3149950000	-5.6510290000	4.3571800000
26	C	-9.5425220000	-5.1355190000	3.1352390000
27	C	-9.8378970000	-3.6461260000	2.8623620000
28	C	3.5827390000	-8.1345030000	-2.7006000000
29	C	3.9137440000	-7.3275000000	-1.4385860000
30	C	2.7084140000	-7.2250590000	-0.4897270000

1	C	2.9655230000	-6.5380290000	0.8803460000
2	C	-8.9507330000	-3.1048880000	1.7090000000
3	C	2.9593710000	-4.9777370000	0.8912900000
4	C	6.2189540000	-5.4649970000	0.2244730000
5	C	5.3568300000	-4.3003780000	0.7549950000
6	C	-10.2467050000	-1.0354540000	1.1508650000
7	C	-10.7346310000	-1.4742860000	-0.2513940000
8	C	-7.8299990000	-1.1590100000	0.7062380000
9	C	-6.4513630000	-1.3107150000	1.3526530000
10	C	3.6016630000	-2.7655770000	0.1076480000
11	C	2.3624710000	-2.4415370000	-0.7333170000
12	C	2.0923430000	3.7681360000	0.6529730000
13	C	2.7166980000	2.3554300000	0.6126330000
14	C	1.9305880000	1.3333390000	-0.2274740000
15	C	2.5698470000	-0.0646380000	-0.1291870000
16	C	0.6360800000	-0.9318830000	-1.6021160000
17	C	-0.5755650000	-1.4570820000	-0.8122640000
18	C	-1.9122960000	-1.2623010000	-1.5045440000
19	C	-3.1579690000	-2.1354220000	0.4707990000
20	C	-3.9853020000	-1.1783240000	1.3305270000
21	C	-5.2499680000	-0.4680160000	-0.6730680000
22	C	-4.3911510000	-1.3997730000	-1.5398560000
23	H	-21.9436810000	7.2648410000	-5.9970400000
24	H	-23.5989020000	6.7297740000	-5.8326810000
25	H	-23.2560060000	8.3621000000	-6.3532610000
26	H	-22.2057710000	8.8031300000	-4.1920270000
27	H	-23.9613980000	8.2356050000	-4.0176980000
28	H	-21.4593400000	6.7255500000	-3.5223290000
29	H	-23.2104740000	6.1371130000	-3.3740940000
30	H	-21.8095020000	8.1928990000	-1.7654700000

1	H	-23.5531470000	7.5843190000	-1.6104470000
2	H	-21.0108060000	6.1865530000	-1.0154440000
3	H	-22.7290640000	5.4939960000	-0.9639220000
4	H	-21.5854710000	7.6578410000	0.7063310000
5	H	-23.2564180000	6.8612550000	0.7962720000
6	H	-20.6227780000	5.6397510000	1.3848920000
7	H	-22.3212310000	4.9216890000	1.5702220000
8	H	-21.0238530000	7.0931770000	3.1167110000
9	H	-22.7595570000	6.4616040000	3.2688560000
10	H	-21.9981610000	4.5522480000	4.3873420000
11	H	-19.8682810000	4.2716510000	5.1951050000
12	H	-18.3680150000	6.2121210000	5.0918320000
13	H	-19.0944440000	6.6121100000	3.4344210000
14	H	-18.1002470000	4.7925450000	2.5055510000
15	H	-17.5195590000	4.2130690000	4.1673900000
16	H	-16.5747720000	6.5061720000	2.5456310000
17	H	-16.0322390000	6.0073330000	4.2460770000
18	H	-15.6437590000	4.6250520000	1.6554950000
19	H	-15.2191640000	3.9978970000	3.3469720000
20	H	-14.0241230000	6.2298790000	1.8095070000
21	H	-13.6533690000	5.7249940000	3.5537360000
22	H	-13.1681760000	4.2321000000	1.0392300000
23	H	-12.8257120000	3.6986490000	2.7807380000
24	H	-27.3776650000	10.5956110000	-6.6143550000
25	H	-27.8801260000	12.1948130000	-6.1212500000
26	H	-27.9110650000	10.8769110000	-4.9744090000
27	H	-25.3157210000	10.9909260000	-5.9831330000
28	H	-25.8486620000	12.6871360000	-5.4601170000
29	H	-25.7747900000	10.1927270000	-3.8613230000
30	H	-26.3067000000	11.8892920000	-3.3384070000

H	-23.6003870000	10.9715250000	-4.1229290000
H	-24.1434580000	12.6463280000	-3.5443020000
H	-24.1055740000	10.1132380000	-2.0077560000
H	-24.5273970000	11.8268770000	-1.4418880000
H	-21.8988870000	10.7595850000	-2.2976210000
H	-22.3259070000	12.4631540000	-1.7058400000
H	-22.4006750000	9.9284000000	-0.1730340000
H	-22.7145630000	11.6611960000	0.4045400000
H	-20.1611680000	10.4978760000	-0.5514150000
H	-20.4992470000	12.1791350000	0.1512710000
H	-20.8313740000	11.1567100000	2.3290690000
H	-19.5852670000	9.2941790000	2.8663620000
H	-19.4498360000	8.7557780000	-0.0984330000
H	-19.0359190000	7.6119280000	1.2997730000
H	-17.4909340000	9.9150900000	0.2540000000
H	-17.0836260000	8.8108680000	1.6856050000
H	-17.1850140000	8.1617810000	-1.2054970000
H	-16.8354960000	7.0238570000	0.2149490000
H	-15.2328130000	9.3103340000	-0.7772830000
H	-14.8886600000	8.2010760000	0.6669480000
H	-14.8524910000	7.5819700000	-2.2308080000
H	-14.6143200000	6.4122840000	-0.8131310000
H	-12.9095960000	8.6893520000	-1.6439370000
H	-12.6716300000	7.5205980000	-0.2254570000
H	-12.4042210000	7.0711850000	-3.1377650000
H	-12.4145510000	5.7765800000	-1.8116460000
H	-11.5454400000	5.8724840000	1.2304880000
H	-11.1921440000	5.3128070000	2.9615700000
H	-8.4224200000	5.9387650000	-0.9610140000
H	-8.7997710000	7.6095340000	-0.2533150000

1	H	-9.5088240000	6.5485000000	1.7484160000
2	H	-6.8052570000	5.6459740000	0.8257130000
3	H	-7.3908500000	5.7414420000	2.5814660000
4	H	-2.7289580000	9.7268590000	2.4512520000
5	H	-4.1003600000	8.8650210000	2.2435100000
6	H	-2.3863080000	7.1443590000	3.4659160000
7	H	-2.3597260000	8.7652000000	4.3641730000
8	H	-4.3425810000	6.6774280000	4.2977580000
9	H	-4.2416630000	8.2241670000	5.3136880000
10	H	-4.3961070000	5.9976160000	2.5169080000
11	H	8.4146830000	9.2683700000	2.0928970000
12	H	7.5062960000	7.8913780000	1.5168830000
13	H	7.2971040000	9.4535240000	0.7625580000
14	H	10.4709330000	8.1602680000	1.6676900000
15	H	9.5018030000	6.6995490000	1.0662010000
16	H	11.5854480000	8.2662080000	-0.3456540000
17	H	10.5895540000	6.8394840000	-0.9838980000
18	H	19.0390640000	4.9903850000	3.2744590000
19	H	16.0893430000	21.9284410000	-8.8814400000
20	H	14.5719730000	21.1128680000	-9.1738360000
21	H	15.4746170000	21.7845190000	-10.5106930000
22	H	17.2916440000	20.2189300000	-10.0357120000
23	H	15.6822290000	19.3538850000	-10.3458450000
24	H	17.0587890000	19.8393380000	-7.7665010000
25	H	15.4486790000	18.9753500000	-8.0759710000
26	H	18.1810670000	18.1393960000	-8.8619780000
27	H	16.5707960000	17.2751160000	-9.1697850000
28	H	18.5786280000	6.1637460000	19.8215360000
29	H	17.0654840000	5.3614540000	19.4754260000
30	H	17.4170790000	6.9128870000	18.7525830000

1	H	19.5336050000	5.2274830000	18.0843350000
2	H	17.9286740000	4.3765240000	17.7172310000
3	H	18.9018520000	6.9987000000	16.7370560000
4	H	17.2983370000	6.1457010000	16.3685020000
5	H	20.0437860000	5.3432790000	15.5930500000
6	H	18.4387130000	4.4947140000	15.2210560000
7	H	19.4154960000	7.1173500000	14.2453630000
8	H	17.8152690000	6.2618350000	13.8684460000
9	H	17.9531190000	17.7578460000	-6.5928080000
10	H	16.3410480000	16.8963110000	-6.8988880000
11	H	15.9542800000	4.0662410000	1.4711290000
12	H	16.5423280000	5.7895550000	1.1260380000
13	H	15.1144540000	3.9473560000	-0.7127120000
14	H	15.6228390000	5.7095880000	-0.9787680000
15	H	13.5603030000	4.5309650000	0.4999710000
16	H	21.6272460000	4.1784280000	1.0956670000
17	H	20.9689280000	4.8073460000	-0.5185760000
18	H	23.0477760000	5.8508510000	0.4990290000
19	H	20.6185040000	7.3449550000	-0.3914090000
20	H	22.2976600000	8.1066670000	-0.2045190000
21	H	21.8368070000	7.5995490000	4.3971430000
22	H	20.2702990000	6.7475560000	3.8923040000
23	H	21.6214600000	9.3955160000	-2.0335870000
24	H	20.0178620000	8.5094040000	-2.3128340000
25	H	21.7817300000	9.8094520000	-4.3051350000
26	H	20.1669340000	8.9402610000	-4.5727970000
27	H	20.6826490000	11.4841090000	-3.1412790000
28	H	19.0689840000	10.6181700000	-3.4257690000
29	H	20.8626970000	11.8922070000	-5.4118430000
30	H	19.2468570000	11.0296060000	-5.6941210000

1	H	19.7598480000	13.5797950000	-4.2742970000
2	H	18.1449140000	12.7193240000	-4.5680290000
3	H	19.9617120000	13.9765680000	-6.5438200000
4	H	18.3476190000	13.1148050000	-6.8383870000
5	H	18.8508670000	15.6712250000	-5.4262400000
6	H	17.2374380000	14.8108230000	-5.7283230000
7	H	19.0698410000	16.0585530000	-7.6956960000
8	H	17.4574930000	15.1964960000	-7.9988320000
9	H	22.3577810000	5.8119750000	5.7704300000
10	H	20.7754070000	4.9813840000	5.2796020000
11	H	21.1417060000	7.4810660000	6.8293350000
12	H	19.5684090000	6.6197550000	6.3628800000
13	H	21.7048250000	5.6911730000	8.1952850000
14	H	20.1104160000	4.8588110000	7.7483660000
15	H	20.5181770000	7.3583860000	9.2872210000
16	H	18.9324760000	6.4985370000	8.8618820000
17	H	21.1134080000	5.5739040000	10.6432710000
18	H	19.5122930000	4.7356380000	10.2328570000
19	H	19.9492140000	7.2368740000	11.7608730000
20	H	18.3545120000	6.3794030000	11.3654390000
21	H	20.5654310000	5.4581280000	13.1112230000
22	H	18.9612040000	4.6141990000	12.7252990000
23	H	-9.7995910000	-9.6342180000	1.5959830000
24	H	-9.3938690000	-9.0849740000	-0.0752870000
25	H	-9.9686430000	-7.8931470000	1.1579060000
26	H	-12.1859470000	-9.0521520000	1.2443600000
27	H	-11.6118450000	-10.2391340000	0.0081330000
28	H	-11.8652110000	-7.1825150000	-0.3938820000
29	H	-11.2845300000	-8.3638760000	-1.6347410000
30	H	-14.0703720000	-8.3719500000	-0.2997660000

1	H	-13.4912260000	-9.5460570000	-1.5474420000
2	H	-13.7541680000	-6.4904030000	-1.9166660000
3	H	-13.1818900000	-7.6576840000	-3.1742750000
4	H	-15.9506550000	-7.6874710000	-1.8060420000
5	H	-15.3922570000	-8.8407320000	-3.0824650000
6	H	-15.6488110000	-5.7784570000	-3.3867270000
7	H	-15.1179670000	-6.9212470000	-4.6844420000
8	H	-20.8311160000	-14.8650580000	4.7939610000
9	H	-22.4526180000	-14.5068300000	4.0867820000
10	H	-21.1057380000	-15.0869370000	3.0244350000
11	H	-19.8218800000	-12.9947080000	3.5107870000
12	H	-21.1649530000	-12.4128630000	4.5710780000
13	H	-21.3850590000	-13.0311540000	1.5495860000
14	H	-22.7291210000	-12.4427330000	2.6072220000
15	H	-20.0822340000	-10.9513240000	2.0443790000
16	H	-21.4278960000	-10.3524510000	3.0944750000
17	H	-21.6361530000	-11.0146480000	0.0853850000
18	H	-22.9810530000	-10.3962560000	1.1241600000
19	H	-20.3141000000	-8.9445300000	0.5553890000
20	H	-21.6648930000	-8.3029570000	1.5734430000
21	H	-21.8432570000	-9.0540270000	-1.4156790000
22	H	-23.1920090000	-8.3747460000	-0.4210450000
23	H	-29.1840620000	-7.2780140000	3.4665440000
24	H	-29.7676450000	-5.7915740000	4.3197180000
25	H	-30.8733180000	-6.6941330000	3.2157330000
26	H	-29.4484810000	-6.1142760000	1.2631930000
27	H	-30.0314680000	-4.6315960000	2.1172450000
28	H	-27.2529440000	-5.9448560000	2.4667620000
29	H	-27.8399180000	-4.4649810000	3.3244170000
30	H	-27.5199580000	-4.7720680000	0.2633010000

1	H	-28.1150240000	-3.2965860000	1.1252100000
2	H	-25.3431650000	-4.6108950000	1.4947310000
3	H	-25.9479310000	-3.1460970000	2.3652930000
4	H	-25.5669510000	-3.3995920000	-0.6940730000
5	H	-26.1899860000	-1.9429060000	0.1813110000
6	H	-23.4262410000	-3.2557700000	0.5984480000
7	H	-24.0608720000	-1.8138290000	1.4861250000
8	H	-11.1036050000	-14.3204000000	11.1080970000
9	H	-12.5432160000	-13.2910300000	10.7538610000
10	H	-11.1575610000	-12.6188630000	11.7064650000
11	H	-9.6702550000	-12.8553160000	9.7077960000
12	H	-11.0520040000	-13.5256850000	8.7551540000
13	H	-10.9324360000	-10.7115660000	10.0319670000
14	H	-12.3147040000	-11.3789680000	9.0750250000
15	H	-9.4312910000	-10.9671590000	8.0441620000
16	H	-10.8130220000	-11.6268510000	7.0811530000
17	H	-10.6864330000	-8.8287820000	8.3840890000
18	H	-12.0649540000	-9.4774640000	7.4094900000
19	H	-9.1685230000	-9.0809340000	6.4112600000
20	H	-10.5469470000	-9.7098400000	5.4233060000
21	H	-10.4021450000	-6.9372980000	6.7772770000
22	H	-11.7734850000	-7.5455740000	5.7670330000
23	H	8.6800150000	-11.6404430000	-9.2857750000
24	H	8.2397650000	-10.1416980000	-10.2013850000
25	H	7.6825510000	-11.7559050000	-10.7850310000
26	H	6.2625280000	-12.0317280000	-8.7659740000
27	H	5.8218720000	-10.5355960000	-9.6795010000
28	H	7.6376560000	-10.6585440000	-7.1795280000
29	H	7.1948910000	-9.1607210000	-8.0913060000
30	H	5.2185090000	-11.0625660000	-6.6659290000

1	H	4.7733090000	-9.5624420000	-7.5736470000
2	H	6.6140780000	-9.6995060000	-5.0971440000
3	H	6.1637420000	-8.1975400000	-5.9974990000
4	H	4.2018090000	-10.1007540000	-4.5540720000
5	H	3.7472750000	-8.5927650000	-5.4444840000
6	H	5.6376380000	-8.7568340000	-3.0078970000
7	H	5.1740370000	-7.2453820000	-3.8842570000
8	H	28.0721360000	-9.5523800000	-6.6002500000
9	H	28.5638390000	-7.9733240000	-7.3362740000
10	H	28.5285660000	-9.4702710000	-8.3440470000
11	H	26.0622240000	-9.6228320000	-8.0879950000
12	H	26.5481760000	-8.0463090000	-8.8270570000
13	H	25.8833270000	-8.5140350000	-5.8449440000
14	H	26.3595950000	-6.9355500000	-6.5861490000
15	H	23.8641800000	-8.6055780000	-7.3242550000
16	H	24.3280170000	-7.0216490000	-8.0660240000
17	H	23.7449720000	-7.5168940000	-5.0769790000
18	H	24.1824940000	-5.9277440000	-5.8173830000
19	H	21.6775810000	-7.6145510000	-6.4864170000
20	H	22.0894980000	-6.0103530000	-7.2175400000
21	H	21.6784180000	-6.5839060000	-4.2174290000
22	H	22.0410460000	-4.9677660000	-4.9366470000
23	H	30.0614690000	-10.5056900000	-3.3736000000
24	H	31.4790410000	-9.4681750000	-3.7877650000
25	H	30.0016240000	-9.4203530000	-4.8221130000
26	H	30.2164970000	-8.4995700000	-1.8813440000
27	H	30.1549020000	-7.4192170000	-3.3289860000
28	H	27.9466840000	-9.4034780000	-2.4573920000
29	H	27.8882910000	-8.3259960000	-3.9093340000
30	H	28.1184580000	-7.3864180000	-0.9708380000

1	H	28.0671540000	-6.3168220000	-2.4286260000
2	H	25.8499430000	-8.2850040000	-1.5507600000
3	H	25.8156700000	-7.2327540000	-3.0214470000
4	H	25.9969730000	-6.2342660000	-0.0962840000
5	H	25.9948840000	-5.2001540000	-1.5805280000
6	H	23.7255810000	-7.1149040000	-0.7152110000
7	H	23.7687660000	-6.1232630000	-2.2259520000
8	H	19.6674860000	-16.4394660000	-3.4420580000
9	H	18.7954990000	-15.0743310000	-4.2372770000
10	H	17.8767900000	-16.2975450000	-3.2682520000
11	H	19.0745110000	-15.5542770000	-1.1984270000
12	H	19.9912710000	-14.3339940000	-2.1664680000
13	H	16.9186000000	-14.4271760000	-1.8184670000
14	H	17.8369990000	-13.2079010000	-2.7887580000
15	H	18.1305220000	-13.6929470000	0.2501890000
16	H	19.0512330000	-12.4774500000	-0.7227510000
17	H	15.9806530000	-12.5660030000	-0.3790760000
18	H	16.9038100000	-11.3567640000	-1.3571500000
19	H	17.1916570000	-11.8173080000	1.6865840000
20	H	18.1213220000	-10.6176400000	0.7024280000
21	H	15.0494560000	-10.6797660000	1.0424840000
22	H	15.9849100000	-9.4944500000	0.0469110000
23	H	-20.4858130000	-7.0005120000	-0.9956570000
24	H	-21.8451130000	-6.2823420000	-0.0413470000
25	H	-8.8528200000	-7.1779280000	4.8307860000
26	H	-10.2239100000	-7.7524780000	3.8004920000
27	H	23.8088430000	-4.9943240000	0.6593740000
28	H	23.9268010000	-4.0374080000	-0.8710730000
29	H	16.2615530000	-9.9021110000	3.0998840000
30	H	17.2079720000	-8.7346480000	2.0926190000

1	H	16.1115970000	-2.9624450000	-3.3293280000
2	H	19.5167540000	-6.6743040000	-5.4802530000
3	H	19.8425620000	-5.0326590000	-6.1726750000
4	H	19.7466230000	-5.7577410000	-3.2034010000
5	H	19.9829150000	-4.0992310000	-3.8483560000
6	H	17.3927020000	-5.7782810000	-4.2332200000
7	H	17.7236410000	-4.1201390000	-4.8604400000
8	H	-21.9686770000	-7.1860010000	-2.9903620000
9	H	-23.3227330000	-6.4137710000	-2.0740660000
10	H	-23.8137510000	-3.7999010000	-3.2308730000
11	H	-20.5576690000	-5.1690450000	-2.6263890000
12	H	-21.9143020000	-4.3337680000	-1.7938200000
13	H	-21.9917820000	-5.4531630000	-4.6704090000
14	H	-17.8438780000	-6.9631280000	-3.2340890000
15	H	-17.3458570000	-8.0809960000	-4.5657820000
16	H	-17.5495120000	-5.0166050000	-4.7386350000
17	H	-17.1189530000	-6.1076560000	-6.1164680000
18	H	-19.7572770000	-6.1669030000	-4.5218300000
19	H	-19.3692000000	-7.2334200000	-5.9008630000
20	H	-19.8255730000	-4.9297410000	-8.3816460000
21	H	-20.8893880000	-5.4061950000	-6.5439370000
22	H	-18.5299140000	-3.5171320000	-6.0789280000
23	H	-20.1103270000	-3.0094820000	-6.6822090000
24	H	-21.8313670000	-2.5379200000	-3.7614430000
25	H	-22.0034190000	-3.0679470000	-5.4377500000
26	H	-23.5740600000	-1.9862620000	-1.5611010000
27	H	-24.2292440000	-0.5521080000	-0.6733920000
28	H	-21.5125490000	-1.8987580000	-0.1614530000
29	H	-22.1526720000	-0.4627410000	0.7169270000
30	H	-21.5530880000	-0.5188060000	-2.3302320000

1	H	-22.2310960000	0.8371540000	-1.4044530000
2	H	-20.1176610000	2.2605100000	-1.6643490000
3	H	-20.2745360000	0.9659600000	0.0530200000
4	H	-18.2396270000	-0.0457530000	-0.2163590000
5	H	-19.3482370000	-1.3909620000	0.0200320000
6	H	18.0848870000	-3.3164510000	-2.5023340000
7	H	21.5641440000	-5.8563530000	-0.0755480000
8	H	21.7579170000	-4.9736100000	-1.6371520000
9	H	21.5177730000	-3.6040430000	1.1405710000
10	H	21.8904580000	-2.8066580000	-0.4312190000
11	H	19.6401010000	-1.6746060000	-0.8215990000
12	H	19.8452980000	-3.7214320000	-1.4273460000
13	H	18.4557790000	-5.8689910000	-1.6088870000
14	H	16.7380710000	-5.9560850000	-1.6445660000
15	H	18.9578300000	-4.2664600000	1.4525590000
16	H	19.3745150000	-5.5873810000	0.3404990000
17	H	14.1356840000	-8.7424290000	2.4245520000
18	H	15.0919510000	-7.6411070000	1.3895130000
19	H	15.2410780000	-5.3626400000	5.1959030000
20	H	15.3222640000	-7.9957250000	4.4349090000
21	H	16.3394680000	-6.8350540000	3.5110880000
22	H	13.2712710000	-6.7593190000	3.6874960000
23	H	15.2441380000	-4.4918900000	2.9835250000
24	H	13.5092720000	-4.3056390000	3.0472940000
25	H	-19.7807620000	-1.3684850000	-4.3927620000
26	H	-18.2833860000	-2.3469250000	-4.3034610000
27	H	-20.3900970000	-2.2907580000	-2.1045140000
28	H	-18.9764240000	-3.3168650000	-2.0951030000
29	H	-17.0637000000	-2.1972210000	-0.7796450000
30	H	-16.8432910000	-2.4424030000	-2.5267510000

1	H	-16.3382490000	0.3215400000	-1.2472910000
2	H	-16.1815780000	-0.1850700000	-2.9530270000
3	H	-12.9971400000	-1.7341240000	1.2170240000
4	H	-13.1161450000	-2.7594950000	-0.2590570000
5	H	-15.2565280000	-1.7361380000	0.3554820000
6	H	-14.5492040000	-0.0823610000	0.2820640000
7	H	-13.7437020000	0.8100860000	-1.8782900000
8	H	-13.8693760000	-0.2138080000	-3.3614140000
9	H	-12.2752290000	-1.8641980000	-2.4355340000
10	H	-11.5617080000	-0.2050570000	-2.4817290000
11	H	16.9090750000	-4.9660750000	1.7869580000
12	H	16.7416650000	-6.3239250000	0.6538210000
13	H	14.9657940000	-4.9861350000	-0.5785670000
14	H	15.1200040000	-3.6531310000	0.6099700000
15	H	12.6047060000	-4.1188350000	0.8361690000
16	H	12.8119510000	-5.5255670000	-0.2647970000
17	H	12.0766390000	-7.0867630000	1.4718220000
18	H	11.6479550000	-5.7261630000	2.5788960000
19	H	8.6127410000	-4.5934930000	-0.7443880000
20	H	8.0709380000	-3.4625340000	0.5511940000
21	H	9.6878370000	-4.3701860000	2.1463600000
22	H	10.4650090000	-3.7699650000	0.6323920000
23	H	9.9348590000	-7.9509390000	1.1630570000
24	H	9.3614530000	-6.8237020000	2.4532420000
25	H	7.5045330000	-7.6468080000	1.0609300000
26	H	8.2976660000	-7.0386400000	-0.4437640000
27	H	-10.0519170000	-5.0229310000	5.2370030000
28	H	-11.4091570000	-5.5713670000	4.1755230000
29	H	-8.4548730000	-5.2523510000	3.3384080000
30	H	-9.7994340000	-5.7409120000	2.2397830000

1	H	-11.4219540000	-3.9661950000	1.7729540000
2	H	-9.5713550000	-3.0842360000	3.7843310000
3	H	3.2389250000	-9.1514740000	-2.4091280000
4	H	2.7690460000	-7.6281760000	-3.2649880000
5	H	4.7347590000	-7.8546890000	-0.9079940000
6	H	4.2623610000	-6.3381190000	-1.7646760000
7	H	2.4339430000	-8.2807550000	-0.2655290000
8	H	1.8366210000	-6.7528290000	-0.9927220000
9	H	2.2273900000	-6.8843640000	2.6556520000
10	H	3.8925460000	-6.9587520000	1.3167180000
11	H	-7.9235430000	-3.4465500000	1.9470520000
12	H	-9.1981660000	-3.6040120000	0.7498100000
13	H	2.9956250000	-4.6540370000	1.9567110000
14	H	1.9423630000	-4.7079590000	0.5457290000
15	H	5.6931950000	-6.3949770000	0.4556110000
16	H	6.3245170000	-5.3977470000	-0.8818210000
17	H	5.2832760000	-4.3433020000	1.8644710000
18	H	5.9393850000	-3.3890290000	0.5208380000
19	H	-11.0057490000	-1.2524750000	1.9196720000
20	H	-10.1769790000	0.0760780000	1.1507890000
21	H	-10.7713280000	-2.5784730000	-0.3562490000
22	H	-9.9936450000	-1.0965250000	-0.9890710000
23	H	2.1817030000	6.7653410000	-0.7615700000
24	H	3.8026260000	3.7329510000	-2.1213320000
25	H	4.8199460000	5.6220850000	0.6171470000
26	H	1.7697940000	6.3478430000	2.6475660000
27	H	0.4222050000	4.6061640000	1.1964960000
28	H	2.7557480000	1.9747840000	1.6574450000
29	H	3.7593340000	2.4201830000	0.2306990000
30	H	0.8897010000	1.2825110000	0.1597480000

H	1.9353890000	1.6519890000	-1.2873500000
H	2.6078430000	-0.3166460000	0.9526640000
H	3.6084680000	0.0093860000	-0.5186080000
H	0.7237660000	-1.4085140000	-2.6032700000
H	0.4422490000	0.1365360000	-1.8002370000
H	-0.4504670000	-2.5389300000	-0.5988920000
H	-0.5942160000	-0.8953000000	0.1468920000
H	-2.1703240000	-2.2753220000	0.9486180000
H	-3.6509700000	-3.1321690000	0.4470220000
H	-4.0550340000	-1.5900090000	2.3596910000
H	-3.4632150000	-0.1983290000	1.3975110000
H	-4.7783810000	0.5390490000	-0.6573960000
H	-6.2495540000	-0.3559050000	-1.1360070000
H	-4.9027680000	-2.3831040000	-1.6299060000
H	-4.3026080000	-0.9723440000	-2.5614480000
H	-8.7734440000	-1.2066520000	2.5339030000
H	-7.8532650000	-1.7330670000	-0.2464650000
H	-7.9469110000	-0.0769750000	0.4733120000
H	4.0441950000	-4.4913250000	-0.8696750000
H	3.4448160000	-2.4170760000	1.1528350000
H	4.4155900000	-2.1567410000	-0.3443470000

6.10. Branched amine core based bone-targeting ionizable lipids 'Type 4'

Type4-B1-C12

P	14.1786130000	1.1761400000	2.6402690000
P	-4.1183420000	-1.6368070000	-6.6869990000
P	4.0449410000	0.8707540000	-8.3604310000
P	4.6328950000	-1.7415100000	-6.9627290000
O	10.8414200000	-0.4077940000	-7.4397530000
O	11.6992110000	-1.8639580000	-2.7374580000

1	O	14.5112190000	2.7361430000	1.9490700000
2	O	13.5712280000	1.4525990000	3.9701880000
3	O	13.3118600000	0.2194360000	1.6842810000
4	O	15.7476400000	0.7400580000	2.7838920000
5	O	18.3738090000	1.0200850000	3.1864420000
6	O	17.7045280000	2.5845200000	4.7078380000
7	O	17.6675870000	-2.4460850000	3.7522490000
8	O	17.4165360000	-4.6791420000	3.9102390000
9	O	11.3049960000	0.0305910000	-3.8950250000
10	O	-8.2948080000	-2.3689160000	-1.8141610000
11	O	-8.4902140000	-0.5563460000	-3.1815810000
12	O	-8.1223570000	3.4623110000	-3.7455070000
13	O	-7.0437080000	1.5688230000	-4.2882580000
14	O	-2.6107360000	-0.8923980000	-6.2522200000
15	O	-5.4796170000	-1.1702690000	-6.0254740000
16	O	-3.8790220000	-1.5367690000	-8.2971080000
17	O	-3.6610730000	-3.0281310000	-6.4271250000
18	O	-4.5046390000	4.6448490000	-1.5433400000
19	O	-0.5400830000	6.4693220000	3.9325730000
20	O	-4.7610600000	-1.2911550000	8.1403290000
21	O	-7.8910650000	-3.0486180000	3.7213240000
22	O	2.6145440000	0.2687330000	-9.1398630000
23	O	3.9503900000	2.6046330000	-8.3820260000
24	O	5.1703530000	0.5625530000	-9.3836820000
25	O	3.1768230000	-2.5573690000	-7.4407320000
26	O	5.8476070000	-2.1913820000	-8.1203250000
27	O	5.0709270000	-2.4855170000	-5.6728520000
28	O	5.6869810000	0.6072230000	-6.2448210000
29	O	1.8395120000	-1.3269590000	3.7614900000
30	O	0.8038910000	-1.4800680000	-2.1510080000

1	O	5.5866960000	1.6402980000	0.4666170000
2	N	11.8373080000	0.1147680000	-1.6606790000
3	N	-1.9110560000	-2.7908210000	-7.5789280000
4	N	-2.6030130000	4.3493160000	1.6430480000
5	N	-4.7164520000	-1.1505690000	4.4013340000
6	N	-2.7355230000	0.4965920000	1.5509160000
7	N	0.1243490000	-0.9824680000	0.6889870000
8	N	2.5496010000	-0.0576380000	-1.9666870000
9	N	3.8375560000	2.3435200000	1.6977860000
10	N	1.9938020000	0.7375220000	2.8974500000
11	C	10.5777820000	0.4434240000	-8.5346080000
12	C	10.9570370000	0.3020640000	-6.2306510000
13	C	11.2312940000	-0.6818220000	-5.1028130000
14	C	22.3142990000	-13.3838090000	-9.4361300000
15	C	21.8000380000	-13.2469540000	-7.9969620000
16	C	21.6350930000	-11.7751390000	-7.5858490000
17	C	21.1188780000	-11.6352260000	-6.1445820000
18	C	18.7392090000	18.0127510000	-1.6211090000
19	C	18.3957170000	16.9982900000	-0.5222060000
20	C	18.6275720000	15.5513780000	-0.9861190000
21	C	18.2861250000	14.5333550000	0.1141080000
22	C	18.5214810000	13.0853060000	-0.3461100000
23	C	20.9544250000	-10.1635160000	-5.7309630000
24	C	13.0170650000	0.6550870000	0.3821660000
25	C	12.1985420000	-0.4118870000	-0.3429340000
26	C	16.2301220000	0.1655350000	3.9707800000
27	C	17.7399130000	-0.0569410000	3.8271250000
28	C	18.0483530000	-1.3158130000	3.0137700000
29	C	18.1178250000	2.2916690000	3.6086260000
30	C	18.3663340000	3.2772810000	2.4776570000

1	C	17.7353920000	-3.7137010000	3.2475240000
2	C	18.2334680000	-3.8368380000	1.8103560000
3	C	18.3865940000	-5.2942020000	1.3384770000
4	C	18.9063850000	-5.3895800000	-0.1059660000
5	C	19.0653620000	-6.8514900000	-0.5551580000
6	C	19.5877320000	-6.9649980000	-1.9965330000
7	C	19.7501850000	-8.4316630000	-2.4289280000
8	C	20.2709880000	-8.5582000000	-3.8697850000
9	C	20.4354020000	-10.0280860000	-4.2901770000
10	C	18.0613930000	4.7337600000	2.8613830000
11	C	18.3423080000	5.7120310000	1.7089280000
12	C	18.0611900000	7.1667290000	2.1200400000
13	C	18.3664560000	8.1622060000	0.9889860000
14	C	18.1068080000	9.6140860000	1.4237740000
15	C	18.4305380000	10.6213850000	0.3085580000
16	C	18.1860000000	12.0709650000	0.7593610000
17	C	11.6122400000	-0.6587380000	-2.7602770000
18	C	-20.9990570000	-1.2535410000	2.0973500000
19	C	-21.1143890000	-1.1177500000	0.5740610000
20	C	-20.4635050000	0.1746520000	0.0555800000
21	C	-20.6115210000	0.3055630000	-1.4680410000
22	C	-19.9554380000	1.5805330000	-2.0204630000
23	C	-20.2090520000	1.7356520000	-3.5286120000
24	C	-19.4852520000	2.9553970000	-4.1195430000
25	C	-19.6694570000	3.0415840000	-5.6418690000
26	C	-19.0164740000	4.2836150000	-6.1928160000
27	C	-17.7670920000	4.3915690000	-6.6727340000
28	C	-16.7237470000	3.3044170000	-6.7688000000
29	C	-15.5004710000	3.6546430000	-5.9059120000
30	C	-14.3325420000	2.6722610000	-6.0990600000

1	C	-13.1026470000	3.0954530000	-5.2782270000
2	C	-11.8706130000	2.2145080000	-5.5451740000
3	C	-10.6341130000	2.7349050000	-4.7912570000
4	C	-26.0994770000	-3.8862580000	1.1679130000
5	C	-24.7553980000	-4.2791370000	0.5409030000
6	C	-24.4355990000	-3.4328530000	-0.7014170000
7	C	-23.0891150000	-3.8239870000	-1.3309380000
8	C	-22.7471670000	-2.9466900000	-2.5460670000
9	C	-21.3576070000	-3.2721770000	-3.1162240000
10	C	-21.0037490000	-2.3816920000	-4.3183600000
11	C	-19.5792370000	-2.6499140000	-4.8273380000
12	C	-19.2137100000	-1.6970840000	-5.9382970000
13	C	-18.2925640000	-0.7211920000	-5.8853910000
14	C	-17.4251950000	-0.3653030000	-4.7029600000
15	C	-16.0413870000	-1.0131600000	-4.8373970000
16	C	-15.1156050000	-0.6397640000	-3.6686830000
17	C	-13.7200310000	-1.2556180000	-3.8483800000
18	C	-12.7602430000	-0.8771650000	-2.7091890000
19	C	-11.3505090000	-1.4295360000	-2.9690880000
20	C	-10.3589040000	-1.0712900000	-1.8512420000
21	C	-8.9367420000	-1.4376000000	-2.2439240000
22	C	-9.3736700000	1.8923680000	-5.0609490000
23	C	-8.1432580000	2.3832510000	-4.3016960000
24	C	-7.2134170000	-0.7503550000	-3.7237060000
25	C	-6.9793500000	0.2793920000	-4.8317740000
26	C	-5.5842500000	0.1029830000	-5.4402590000
27	C	-1.5273230000	-1.6725380000	-8.5409270000
28	C	-2.8027580000	-0.8786320000	-8.8966680000
29	C	-8.2634130000	12.4676940000	9.1175320000
30	C	-8.2673550000	12.1432860000	7.6268050000

1	C	-7.5620230000	10.8108520000	7.3465890000
2	C	-7.5652910000	10.4973710000	5.8463990000
3	C	-6.8479010000	9.1739690000	5.5521290000
4	C	-6.8411650000	8.8873290000	4.0467590000
5	C	-6.0958330000	7.5864770000	3.7257440000
6	C	-11.2735150000	-0.1145680000	9.4417510000
7	C	-9.8198540000	0.3404440000	9.5339000000
8	C	-9.3753730000	1.0403760000	8.2442700000
9	C	-7.9131400000	1.4919050000	8.3401460000
10	C	-7.4642930000	2.1858770000	7.0487730000
11	C	-5.9954000000	2.6185380000	7.1380910000
12	C	-5.5426540000	3.2949770000	5.8382510000
13	C	-4.0620890000	3.6940130000	5.9049420000
14	C	-3.6384490000	4.4399290000	4.6326870000
15	C	3.3409220000	-10.3731830000	11.6172600000
16	C	3.2184460000	-9.2421120000	10.6000490000
17	C	1.8103750000	-8.6373900000	10.6144900000
18	C	1.6864000000	-7.5004600000	9.5925360000
19	C	0.2767400000	-6.9001220000	9.6163120000
20	C	0.1364140000	-5.7596270000	8.6001190000
21	C	-1.2775810000	-5.1725760000	8.6479040000
22	C	-3.5922540000	-14.4657940000	8.1544410000
23	C	-3.1918570000	-13.2402820000	7.3385410000
24	C	-4.3082310000	-12.1891400000	7.3324040000
25	C	-3.8978100000	-10.9596720000	6.5139770000
26	C	-5.0056300000	-9.8988040000	6.5129420000
27	C	-4.5792400000	-8.6688590000	5.7040250000
28	C	-5.6649590000	-7.5852800000	5.7177000000
29	C	-6.0659450000	7.3474230000	2.2122160000
30	C	-5.2029450000	-6.3534500000	4.9322970000

1	C	-5.2800920000	6.0806210000	1.8532130000
2	C	-5.1941210000	5.9201820000	0.3370180000
3	C	-2.1731890000	4.9048640000	4.7154140000
4	C	-1.7377270000	5.8341130000	3.5567730000
5	C	-1.4528220000	-4.0264740000	7.6433790000
6	C	-2.8759250000	-3.4677000000	7.7313160000
7	C	-4.4118320000	4.6804280000	-0.1407000000
8	C	-2.8995030000	4.6924280000	0.2155810000
9	C	-1.4688880000	5.1657350000	2.1841860000
10	C	-3.1137700000	-2.3217250000	6.7360130000
11	C	-4.5617020000	-1.8101290000	6.8493100000
12	C	-6.2433870000	-5.2264290000	4.9785550000
13	C	-5.7125700000	-3.9886530000	4.2458070000
14	C	-6.6671480000	-2.7773590000	4.3591580000
15	C	-6.0221500000	-1.5032540000	3.7690460000
16	C	-4.8772470000	-0.7069000000	5.8182860000
17	C	-2.2012680000	2.9130460000	1.7616670000
18	C	-3.2774850000	1.8832260000	1.3982230000
19	C	-2.2469220000	-0.0583380000	0.2519110000
20	C	-3.7027240000	-0.4385830000	2.1724550000
21	C	-0.7158330000	0.0736400000	0.0195260000
22	C	-3.9992800000	-0.0861020000	3.6496820000
23	C	4.4565630000	0.1048940000	-6.6993900000
24	C	3.3227210000	0.4420870000	-5.7019900000
25	C	3.5543030000	-0.0556370000	-4.2633420000
26	C	2.4025850000	0.4007770000	-3.3536810000
27	C	2.4876360000	2.8924440000	1.8046770000
28	C	1.5141030000	1.7291660000	1.9481300000
29	C	3.1374290000	1.1209000000	3.7444090000
30	C	4.3246220000	1.6001840000	2.8701480000

1	C	0.1133490000	-0.9092400000	2.1979630000
2	C	1.3881340000	-0.4985130000	2.9825180000
3	C	1.4844870000	-1.2464410000	0.0955730000
4	C	1.6131200000	-0.9295200000	-1.4156290000
5	C	3.7020670000	0.4262600000	-1.2006420000
6	C	3.6403850000	1.9265360000	-0.8115420000
7	C	4.4262790000	2.0344780000	0.4885440000
8	H	11.3757290000	1.1487130000	-8.6382800000
9	H	9.6593090000	0.9661050000	-8.3669430000
10	H	10.4984790000	-0.1393460000	-9.4284700000
11	H	11.8230450000	0.9244830000	-6.3174000000
12	H	10.0006860000	0.7402460000	-6.0349770000
13	H	12.2013150000	-1.0972650000	-5.2799180000
14	H	10.3784000000	-1.3243460000	-5.0347360000
15	H	15.4552140000	2.9071050000	1.9843640000
16	H	23.2662770000	-12.9029010000	-9.5219580000
17	H	21.6203590000	-12.9236260000	-10.1081270000
18	H	22.4148760000	-14.4203340000	-9.6818930000
19	H	22.5641310000	-13.6505850000	-7.3659760000
20	H	20.8183720000	-13.6725680000	-7.9877020000
21	H	22.6169840000	-11.3500380000	-7.5947050000
22	H	20.8716810000	-11.3716080000	-8.2177220000
23	H	21.8819910000	-12.0398410000	-5.5130390000
24	H	20.1367210000	-12.0597230000	-6.1362080000
25	H	19.5141960000	18.6644470000	-1.2752960000
26	H	17.8693120000	18.5882650000	-1.8597760000
27	H	19.0736320000	17.4929170000	-2.4945120000
28	H	19.0891210000	17.1725230000	0.2738680000
29	H	17.3444580000	17.0917210000	-0.3460670000
30	H	19.6786350000	15.4587980000	-1.1638740000

1	H	17.9331060000	15.3770340000	-1.7812410000
2	H	18.9785600000	14.7102570000	0.9104360000
3	H	17.2345080000	14.6241370000	0.2894960000
4	H	19.5723230000	12.9952950000	-0.5264650000
5	H	17.8253400000	12.9059130000	-1.1386410000
6	H	21.9366160000	-9.7390540000	-5.7367980000
7	H	20.1925330000	-9.7578630000	-6.3633120000
8	H	12.3864380000	1.5139690000	0.4797960000
9	H	13.9502030000	0.7325920000	-0.1356620000
10	H	11.2886900000	-0.5375100000	0.2059630000
11	H	12.8454660000	-1.2486210000	-0.5050010000
12	H	10.9548940000	0.5575560000	-1.5016670000
13	H	16.0936210000	0.8899370000	4.7463500000
14	H	15.7787740000	-0.8008710000	4.0558880000
15	H	18.1108010000	-0.1567480000	4.8258140000
16	H	17.4216550000	-1.2862070000	2.1470100000
17	H	19.1117250000	-1.3610880000	2.9038160000
18	H	19.4172540000	3.2391860000	2.2801320000
19	H	17.6677450000	3.0236590000	1.7078840000
20	H	19.2194320000	-3.4212170000	1.8046630000
21	H	17.4687030000	-3.4083520000	1.1968140000
22	H	19.1436440000	-5.7294790000	1.9567890000
23	H	17.4003570000	-5.7090880000	1.3281110000
24	H	19.8898980000	-4.9681930000	-0.1000440000
25	H	18.1461470000	-4.9649450000	-0.7277570000
26	H	19.8229910000	-7.2748800000	0.0706520000
27	H	18.0807150000	-7.2702210000	-0.5611340000
28	H	20.5706090000	-6.5420970000	-1.9938900000
29	H	18.8280460000	-6.5490900000	-2.6248630000
30	H	20.5099390000	-8.8458920000	-1.7995710000

1	H	18.7668090000	-8.8534120000	-2.4289700000
2	H	21.2532420000	-8.1338520000	-3.8720860000
3	H	19.5099420000	-8.1490130000	-4.5008760000
4	H	21.1968950000	-10.4359670000	-3.6587820000
5	H	19.4528750000	-10.4517850000	-4.2855550000
6	H	18.7490080000	4.9866620000	3.6412070000
7	H	17.0076650000	4.7801030000	3.0414090000
8	H	19.3925610000	5.6524660000	1.5131740000
9	H	17.6399790000	5.4766820000	0.9367590000
10	H	18.7486010000	7.3916770000	2.9085600000
11	H	17.0074150000	7.2285730000	2.2950690000
12	H	19.4170090000	8.0887260000	0.7996720000
13	H	17.6661010000	7.9529140000	0.2075770000
14	H	18.7958580000	9.8157010000	2.2171610000
15	H	17.0537350000	9.6896790000	1.5976220000
16	H	19.4812850000	10.5377290000	0.1246230000
17	H	17.7325640000	10.4306670000	-0.4797050000
18	H	18.8768030000	12.2569140000	1.5550460000
19	H	17.1337210000	12.1561290000	0.9335900000
20	H	-19.9663620000	-1.2660750000	2.3771420000
21	H	-21.4858450000	-0.4245800000	2.5672000000
22	H	-21.4645000000	-2.1647070000	2.4104630000
23	H	-20.5476720000	-1.9247410000	0.1587340000
24	H	-22.1593280000	-1.0322000000	0.3603220000
25	H	-19.4152660000	0.0830830000	0.2497770000
26	H	-21.0180880000	0.9838190000	0.4828800000
27	H	-20.0683860000	-0.5140430000	-1.8901200000
28	H	-21.6599850000	0.4062290000	-1.6564280000
29	H	-18.8997210000	1.4506190000	-1.9043420000
30	H	-20.4486640000	2.4029870000	-1.5459130000

1	H	-19.7732930000	0.8763090000	-3.9939540000
2	H	-21.2558520000	1.9293190000	-3.6363370000
3	H	-18.4406750000	2.7952340000	-3.9518930000
4	H	-19.9667970000	3.8183670000	-3.7093030000
5	H	-19.1406690000	2.2101890000	-6.0590800000
6	H	-20.7199250000	3.1488040000	-5.8148450000
7	H	-19.6113490000	5.1729580000	-6.2024470000
8	H	-17.4777830000	5.3586310000	-7.0276880000
9	H	-16.3874130000	3.2933910000	-7.7845060000
10	H	-17.1582990000	2.4258860000	-6.3395590000
11	H	-15.8144710000	3.5447600000	-4.8889410000
12	H	-15.1512140000	4.6025700000	-6.2585490000
13	H	-14.6564930000	1.7355710000	-5.6958620000
14	H	-14.0469700000	2.7417730000	-7.1279020000
15	H	-13.3629670000	2.9362760000	-4.2526560000
16	H	-12.8437190000	4.0763260000	-5.6184410000
17	H	-12.0926450000	1.2526160000	-5.1324190000
18	H	-11.6508700000	2.3114870000	-6.5878670000
19	H	-10.8512100000	2.6182400000	-3.7500280000
20	H	-10.4315190000	3.7069920000	-5.1898820000
21	H	-25.9415350000	-3.5594400000	2.1744630000
22	H	-26.7546700000	-4.7322020000	1.1662440000
23	H	-26.5395770000	-3.0931430000	0.6003030000
24	H	-24.0054050000	-4.0421900000	1.2663450000
25	H	-24.8678650000	-5.2860930000	0.1969660000
26	H	-24.3240310000	-2.4258850000	-0.3572250000
27	H	-25.1855490000	-3.6703300000	-1.4267310000
28	H	-22.3423210000	-3.6176270000	-0.5929580000
29	H	-23.2111720000	-4.8190630000	-1.7048710000
30	H	-22.6942610000	-1.9411890000	-2.1840330000

1	H	-23.4517280000	-3.2042160000	-3.3090690000
2	H	-20.6545050000	-3.0293350000	-2.3470840000
3	H	-21.4153990000	-4.2724040000	-3.4918640000
4	H	-21.0085570000	-1.3744800000	-3.9572490000
5	H	-21.6641160000	-2.6695400000	-5.1095360000
6	H	-18.9204360000	-2.4325650000	-4.0126970000
7	H	-19.5910810000	-3.6287750000	-5.2592990000
8	H	-19.7418220000	-1.8102200000	-6.8619840000
9	H	-18.1602470000	-0.1366110000	-6.7717650000
10	H	-17.8858220000	-0.8056050000	-3.8433920000
11	H	-17.2718670000	0.6928290000	-4.7447560000
12	H	-16.1976410000	-2.0695240000	-4.7697090000
13	H	-15.5983640000	-0.5930900000	-5.7161300000
14	H	-15.5352280000	-1.0915540000	-2.7942110000
15	H	-14.9858420000	0.4214220000	-3.7127850000
16	H	-13.8524130000	-2.3155610000	-3.7859630000
17	H	-13.3142130000	-0.8185390000	-4.7367340000
18	H	-13.1220360000	-1.3711210000	-1.8316850000
19	H	-12.6755330000	0.1893400000	-2.7262770000
20	H	-11.4421590000	-2.4954960000	-2.9538920000
21	H	-10.9960500000	-0.9355360000	-3.8495550000
22	H	-10.6103730000	-1.6885660000	-1.0142040000
23	H	-10.3818200000	-0.0057620000	-1.7562500000
24	H	-9.5852920000	0.9160210000	-4.6777210000
25	H	-9.1496970000	2.0226530000	-6.0991020000
26	H	-6.5101450000	-0.5353820000	-2.9464710000
27	H	-7.2172890000	-1.7132000000	-4.1904130000
28	H	-7.7278460000	0.1398870000	-5.5835680000
29	H	-4.8886420000	0.1264360000	-4.6275600000
30	H	-5.5026950000	0.8147820000	-6.2349860000

1	H	-1.7146520000	-3.6757750000	-8.0011620000
2	H	-2.8871180000	-2.7304740000	-7.3699750000
3	H	-0.8829410000	-1.0066000000	-8.0059520000
4	H	-1.1865500000	-2.1379040000	-9.4421520000
5	H	-2.7190220000	0.0816090000	-8.4320980000
6	H	-2.9365940000	-0.9506910000	-9.9558160000
7	H	-2.6163020000	0.0269080000	-6.5287070000
8	H	-8.7958830000	11.6759360000	9.6862470000
9	H	-7.2206630000	12.5486210000	9.4920110000
10	H	-8.7784510000	13.4364390000	9.2884390000
11	H	-9.3202800000	12.0908950000	7.2731850000
12	H	-7.7487970000	12.9621840000	7.0816910000
13	H	-8.0824230000	9.9946000000	7.8945270000
14	H	-6.5109210000	10.8708240000	7.7061920000
15	H	-8.6155850000	10.4351550000	5.4850920000
16	H	-7.0489130000	11.3198630000	5.3032730000
17	H	-7.3611810000	8.3438550000	6.0855830000
18	H	-5.7986630000	9.2396750000	5.9161570000
19	H	-7.8884660000	8.8142180000	3.6786680000
20	H	-6.3370180000	9.7301030000	3.5233670000
21	H	-6.5948070000	6.7304690000	4.2311520000
22	H	-5.0518110000	7.6678040000	4.1010740000
23	H	-11.5671040000	-0.6170040000	10.3875420000
24	H	-11.9421090000	0.7577130000	9.2802890000
25	H	-11.4011310000	-0.8325930000	8.6036480000
26	H	-9.1774650000	-0.5484880000	9.7172680000
27	H	-9.7162990000	1.0387360000	10.3930160000
28	H	-9.4862070000	0.3368210000	7.3894970000
29	H	-10.0230360000	1.9268730000	8.0648660000
30	H	-7.2696930000	0.6034940000	8.5203340000

1	H	-7.7990770000	2.1951970000	9.1944250000
2	H	-7.5876080000	1.4828780000	6.1956630000
3	H	-8.1021380000	3.0791310000	6.8682860000
4	H	-5.3629530000	1.7281100000	7.3330670000
5	H	-5.8692600000	3.3277580000	7.9856450000
6	H	-5.6941280000	2.6016500000	4.9828650000
7	H	-6.1644550000	4.2027700000	5.6736010000
8	H	-3.4350750000	2.7843290000	6.0334320000
9	H	-3.9098180000	4.3616500000	6.7816190000
10	H	-3.8086980000	3.7868820000	3.7600670000
11	H	-4.2855130000	5.3361760000	4.5357700000
12	H	3.1423010000	-9.9939420000	12.6424170000
13	H	2.6187490000	-11.1839750000	11.3821370000
14	H	4.3685980000	-10.7924330000	11.5863310000
15	H	3.9652780000	-8.4559000000	10.8459460000
16	H	3.4423970000	-9.6437550000	9.5877400000
17	H	1.5924440000	-8.2420550000	11.6314140000
18	H	1.0683600000	-9.4303480000	10.3728620000
19	H	2.4274130000	-6.7066080000	9.8336310000
20	H	1.9012310000	-7.8946310000	8.5748660000
21	H	0.0645800000	-6.5092010000	10.6363190000
22	H	-0.4637730000	-7.6957220000	9.3781160000
23	H	0.8741840000	-4.9614140000	8.8363380000
24	H	0.3410670000	-6.1474330000	7.5780010000
25	H	-1.4771890000	-4.7912040000	9.6740270000
26	H	-2.0130620000	-5.9753370000	8.4168740000
27	H	-3.7957310000	-14.1784380000	9.2081510000
28	H	-2.7659360000	-15.2073660000	8.1416930000
29	H	-4.5004510000	-14.9373950000	7.7224440000
30	H	-2.2693460000	-12.8027900000	7.7791860000

1	H	-2.9724910000	-13.5590070000	6.2960870000
2	H	-4.5235260000	-11.8780500000	8.3786860000
3	H	-5.2293490000	-12.6321580000	6.8935650000
4	H	-2.9738580000	-10.5220540000	6.9532660000
5	H	-3.6837040000	-11.2692560000	5.4671530000
6	H	-5.2170680000	-9.5918640000	7.5611160000
7	H	-5.9316410000	-10.3286400000	6.0714290000
8	H	-3.6468210000	-8.2523390000	6.1463500000
9	H	-4.3719100000	-8.9709300000	4.6535700000
10	H	-5.8679220000	-7.2901290000	6.7710840000
11	H	-6.6014740000	-7.9839470000	5.2695680000
12	H	-7.1060030000	7.2609910000	1.8271450000
13	H	-5.5770750000	8.2210540000	1.7259800000
14	H	-4.2547300000	-5.9829220000	5.3828670000
15	H	-5.0077000000	-6.6365230000	3.8743970000
16	H	-5.7640500000	5.1878370000	2.3063010000
17	H	-4.2621120000	6.1839000000	2.2478410000
18	H	-5.3620150000	4.1992910000	-1.7730160000
19	H	-6.2325380000	5.8427210000	-0.0551710000
20	H	-4.7271450000	6.8278970000	-0.1060900000
21	H	-0.7800680000	7.2465690000	4.5028380000
22	H	-1.4726970000	4.0480500000	4.8169320000
23	H	-2.1033070000	5.5094490000	5.6473100000
24	H	-2.5165930000	6.6160650000	3.4251900000
25	H	-0.7237040000	-3.2175980000	7.8692690000
26	H	-1.2654430000	-4.4039360000	6.6140340000
27	H	-3.0542600000	-3.1013560000	8.7662800000
28	H	-3.5971170000	-4.2875820000	7.5152520000
29	H	-4.8959960000	3.7735540000	0.2756000000
30	H	-2.5242250000	5.6942370000	-0.0857770000

1	H	-2.3706880000	3.9802990000	-0.4592620000
2	H	-0.5317570000	4.5660480000	2.2754350000
3	H	-1.1848490000	5.9974270000	1.5040010000
4	H	-5.7338910000	-1.3507280000	8.3300830000
5	H	-2.4044320000	-1.4887320000	6.9359120000
6	H	-2.9258020000	-2.6989160000	5.7084340000
7	H	-5.2230750000	-2.6855950000	6.7099310000
8	H	-6.4327830000	-4.9602050000	6.0419430000
9	H	-7.1965530000	-5.5647540000	4.5172800000
10	H	-5.5255040000	-4.2276940000	3.1757720000
11	H	-4.7386000000	-3.7256090000	4.6995140000
12	H	-7.7013740000	-3.2377040000	2.7650430000
13	H	-6.9066150000	-2.5807460000	5.4205430000
14	H	-6.7310750000	-0.6476580000	3.8376470000
15	H	-5.8812760000	-1.7359790000	2.6976860000
16	H	-4.1857090000	0.1311720000	6.0662180000
17	H	-5.9035180000	-0.3164410000	5.9943830000
18	H	-1.8962340000	2.6917420000	2.8086120000
19	H	-1.3251480000	2.7428840000	1.1135660000
20	H	-4.1424890000	2.0790350000	2.0569180000
21	H	-3.6545550000	2.0455270000	0.3732580000
22	H	-2.5165680000	-1.1293380000	0.1109150000
23	H	-2.7643010000	0.4363400000	-0.5991810000
24	H	-3.2480680000	-1.4548240000	2.1974940000
25	H	-4.6255540000	-0.4697700000	1.5558560000
26	H	-0.3736110000	1.0978830000	0.1754830000
27	H	-0.6602410000	-0.0591310000	-1.0724840000
28	H	-3.0056950000	0.0586420000	4.1318320000
29	H	-4.5612050000	0.8653990000	3.7313670000
30	H	1.8640110000	0.6394550000	-8.6108870000

H	3.0850520000	2.8159950000	-7.9496040000
H	3.1216820000	-2.4218890000	-8.4200320000
H	6.7045500000	-1.9071340000	-7.7130920000
H	5.6082200000	1.5957680000	-6.1985550000
H	2.3643880000	0.0276880000	-6.0854850000
H	3.2238650000	1.5497520000	-5.6626340000
H	3.5954040000	-1.1617260000	-4.2379910000
H	4.5161120000	0.3585600000	-3.9003310000
H	1.4560320000	0.0320450000	-3.8044410000
H	2.3391160000	1.5106260000	-3.3593110000
H	2.4397260000	3.5386120000	2.7080970000
H	2.2200670000	3.5608900000	0.9594340000
H	0.4959780000	2.0685620000	2.2091230000
H	1.5264010000	1.2744110000	0.9604450000
H	2.8058550000	1.9784440000	4.3694370000
H	3.4663550000	0.3289190000	4.4510470000
H	5.0632430000	2.1855010000	3.4576460000
H	4.8177280000	0.6593400000	2.5344700000
H	3.8411190000	-0.1907920000	-0.3040440000
H	4.6595680000	0.2051920000	-1.7138430000
H	2.5839060000	2.2016110000	-0.6746760000
H	4.0360650000	2.5701860000	-1.6253050000
H	-0.6916750000	-0.2480220000	2.5589420000
H	-0.1605070000	-1.9221560000	2.5761640000
H	-0.4023760000	-1.8565910000	0.4473300000
H	2.2262600000	-0.7165980000	0.6822210000
H	1.7369330000	-2.3202680000	0.2462750000

6.11. Piperazine amine core based bone-targeting ionizable lipids 'Type 4'

Type4-P1-C12

1	P	-2.2847430000	-8.7323400000	-3.8890150000
2	P	16.6630210000	4.5957790000	0.6858110000
3	P	7.9456060000	-6.9712560000	-1.7375530000
4	P	5.5865480000	-8.8608560000	-1.7486130000
5	O	-6.0926660000	-6.4788340000	0.8168150000
6	O	-6.3079450000	-5.6314320000	-1.2877020000
7	O	-5.7921680000	-2.5368990000	-3.8878510000
8	O	-4.8526400000	-4.5183210000	-3.4034830000
9	O	-0.7132390000	-7.9978830000	-3.9726950000
10	O	-3.5652160000	-7.8794920000	-3.5130080000
11	O	-2.1691350000	-9.5034070000	-5.3214340000
12	O	-1.8760890000	-9.8184250000	-2.9585150000
13	O	13.4497390000	-5.5239760000	-0.9009890000
14	O	13.4734880000	-1.1798740000	1.5680200000
15	O	17.5919320000	4.1875270000	-0.7257300000
16	O	16.1435530000	5.9675620000	0.4360200000
17	O	15.5448900000	3.5044820000	1.0590560000
18	O	17.9426900000	4.6221980000	1.7028610000
19	O	20.4582900000	5.0067700000	2.5159340000
20	O	20.3589430000	6.7989850000	1.1059220000
21	O	18.4699930000	4.9590800000	5.4954690000
22	O	17.3872900000	4.7204490000	7.4564230000
23	O	13.8783440000	-1.9698900000	-0.5048150000
24	O	-8.0187620000	4.4605940000	4.2120790000
25	O	-9.9540530000	8.3284590000	2.8536140000
26	O	-6.5663340000	4.1654260000	-3.2100180000
27	O	8.9997300000	-8.1214340000	-0.9732720000
28	O	8.3037490000	-6.9991240000	-3.4371060000
29	O	8.5309470000	-5.6142420000	-1.2647590000
30	O	5.6292260000	-9.2091650000	-3.4490830000

1	O	6.4222690000	-10.1568220000	-0.9484970000
2	O	4.0985230000	-9.0927790000	-1.3704980000
3	O	6.0296840000	-6.9818170000	0.1219210000
4	O	6.0611060000	1.8660220000	1.4000060000
5	O	2.8868900000	-2.1772780000	-0.6961310000
6	O	7.8755310000	0.3131420000	-3.3614830000
7	N	-0.2133700000	-10.3594370000	-4.1367330000
8	N	14.2980050000	0.2303200000	0.0106490000
9	N	-9.3185510000	5.6837320000	1.7032780000
10	N	-6.3290740000	4.4389940000	-0.3428670000
11	N	-2.8902330000	2.8304190000	-0.4494990000
12	N	-0.4911080000	1.2547550000	-0.1150860000
13	N	3.2349540000	0.4530690000	0.2935940000
14	N	4.7810870000	-2.2992320000	-1.9284130000
15	N	6.2906070000	1.8940610000	-3.1038270000
16	N	5.5973150000	2.6258210000	-0.6629310000
17	C	-18.3784630000	-2.4491330000	4.0974550000
18	C	-18.6073740000	-3.1154030000	2.7351650000
19	C	-17.9379140000	-2.3393050000	1.5896220000
20	C	-18.1996310000	-3.0071990000	0.2309850000
21	C	-17.5285240000	-2.2639880000	-0.9345250000
22	C	-17.8929880000	-2.8945350000	-2.2882140000
23	C	-17.1601750000	-2.2238890000	-3.4604260000
24	C	-17.4599830000	-2.9261580000	-4.7930060000
25	C	-16.7934370000	-2.2099390000	-5.9398790000
26	C	-15.5822340000	-2.4706060000	-6.4575380000
27	C	-14.6035240000	-3.5312930000	-6.0139570000
28	C	-13.3002650000	-2.8862210000	-5.5149210000
29	C	-12.2001340000	-3.9185210000	-5.2140770000
30	C	-10.8900900000	-3.2337830000	-4.7893210000

1	C	-9.7270740000	-4.2234420000	-4.6071030000
2	C	-8.4110560000	-3.4913380000	-4.2904700000
3	C	-23.6576980000	-4.7498190000	4.9011100000
4	C	-22.3880540000	-5.5206700000	4.5166160000
5	C	-22.1271300000	-5.4726540000	3.0028650000
6	C	-20.8552060000	-6.2435240000	2.6152190000
7	C	-20.5685780000	-6.1568370000	1.1074240000
8	C	-19.2458900000	-6.8445950000	0.7340820000
9	C	-18.9457370000	-6.7409260000	-0.7700690000
10	C	-17.5806360000	-7.3510870000	-1.1228290000
11	C	-17.2586320000	-7.1479950000	-2.5825510000
12	C	-16.2901160000	-6.3665960000	-3.0875950000
13	C	-15.3156500000	-5.5213240000	-2.3046140000
14	C	-13.9797590000	-6.2564440000	-2.1381500000
15	C	-12.9473710000	-5.4082320000	-1.3784770000
16	C	-11.6018020000	-6.1406150000	-1.2677800000
17	C	-10.5376490000	-5.3062460000	-0.5373120000
18	C	-9.1812660000	-6.0274690000	-0.5287010000
19	C	-8.0881000000	-5.2240030000	0.1928460000
20	C	-6.7208680000	-5.8569820000	-0.0094930000
21	C	-7.2181160000	-4.4521020000	-4.1319050000
22	C	-5.9091620000	-3.7418250000	-3.7950170000
23	C	-5.0889620000	-6.1834940000	-1.7012910000
24	C	-4.8939050000	-5.9026250000	-3.1932850000
25	C	-3.5615330000	-6.4839610000	-3.6775530000
26	C	0.1465530000	-9.9397520000	-5.5571450000
27	C	-1.1131960000	-9.3439970000	-6.2217350000
28	C	13.5784860000	-6.4422120000	-1.9654360000
29	C	13.7681900000	-4.2103410000	-1.2910500000
30	C	13.5976300000	-3.2836710000	-0.0962870000

1	C	19.2536900000	-10.0800060000	14.8960420000
2	C	18.7625280000	-8.6279520000	14.8275020000
3	C	19.1465000000	-7.9561600000	13.4994510000
4	C	18.6545870000	-6.5014490000	13.4277570000
5	C	27.4368810000	3.3128180000	-13.6218020000
6	C	26.6848350000	4.2200220000	-12.6388910000
7	C	26.3746120000	3.4991900000	-11.3172680000
8	C	25.6230510000	4.4070100000	-10.3301130000
9	C	25.3154630000	3.6895280000	-9.0054820000
10	C	19.0388570000	-5.8272220000	12.1004250000
11	C	15.4975990000	2.3087030000	0.3241590000
12	C	14.3716470000	1.4236200000	0.8561780000
13	C	18.1165030000	5.6753800000	2.6150910000
14	C	19.4354660000	5.4588860000	3.3654680000
15	C	19.2833960000	4.4262400000	4.4843780000
16	C	20.6819480000	5.6557250000	1.3371520000
17	C	21.3367590000	4.7140410000	0.3387880000
18	C	18.0776620000	4.2340590000	6.5847870000
19	C	18.5589420000	2.7868080000	6.6350680000
20	C	18.1723360000	2.0581140000	7.9349020000
21	C	18.6845200000	0.6081700000	7.9669390000
22	C	18.3005480000	-0.0991620000	9.2770820000
23	C	18.8081280000	-1.5493950000	9.3271060000
24	C	18.4247910000	-2.2411380000	10.6458200000
25	C	18.9259780000	-3.6931590000	10.7071750000
26	C	18.5426760000	-4.3737210000	12.0316530000
27	C	21.5867520000	5.3594290000	-1.0332710000
28	C	22.2697640000	4.3945390000	-2.0164080000
29	C	22.5398360000	5.0659860000	-3.3730240000
30	C	23.2508650000	4.1246550000	-4.3588140000

1	C	23.5386230000	4.8183820000	-5.7005860000
2	C	24.2704500000	3.8943140000	-6.6873400000
3	C	24.5705640000	4.6030260000	-8.0184910000
4	C	13.8491860000	-0.9844450000	0.4360760000
5	C	-21.4332710000	3.6370390000	7.3618760000
6	C	-20.0578900000	3.0542490000	7.6755280000
7	C	-18.9601680000	3.7896460000	6.9003040000
8	C	-17.5751980000	3.1998840000	7.1998770000
9	C	-16.4964280000	3.9416260000	6.4055480000
10	C	-15.0967210000	3.3582670000	6.6506930000
11	C	-14.0708860000	4.1162200000	5.8045460000
12	C	-23.3212000000	5.5998550000	3.7123230000
13	C	-22.2510720000	6.6521000000	3.4368890000
14	C	-20.8759510000	6.0039970000	3.2428720000
15	C	-19.8008680000	7.0639450000	2.9741530000
16	C	-18.4260510000	6.4122350000	2.7921010000
17	C	-17.3361290000	7.4665940000	2.5601450000
18	C	-15.9636520000	6.7989890000	2.4272920000
19	C	-11.5607340000	16.9902770000	-4.0886140000
20	C	-10.8482910000	15.8398960000	-4.7942370000
21	C	-10.6136170000	14.6670030000	-3.8365260000
22	C	-9.8956010000	13.5105780000	-4.5428320000
23	C	-9.6699560000	12.3429310000	-3.5768490000
24	C	-8.9494170000	11.1768510000	-4.2646260000
25	C	-8.7447530000	10.0221070000	-3.2792470000
26	C	-14.8395060000	7.8342870000	2.2833850000
27	C	-8.0236870000	8.8361800000	-3.9325630000
28	C	-12.6432060000	3.5594620000	5.9400480000
29	C	-11.7223730000	4.3496540000	5.0055710000
30	C	-10.2555970000	3.8625880000	4.9923240000

1	C	-9.3871800000	4.7228470000	4.0324930000
2	C	-13.4785280000	7.1304920000	2.2593710000
3	C	-12.3050610000	8.1283790000	2.2804880000
4	C	-10.9585880000	7.4074130000	2.5090770000
5	C	-7.8557980000	7.7022090000	-2.9163110000
6	C	-7.1479540000	6.4800440000	-3.5264090000
7	C	-7.0865920000	5.2889510000	-2.5465710000
8	C	-9.7670830000	4.5329500000	2.5390320000
9	C	-10.4822010000	6.5388650000	1.3224950000
10	C	-6.2096400000	5.5675100000	-1.3081000000
11	C	-8.5929840000	5.2345470000	0.4939790000
12	C	-7.1474130000	4.8169080000	0.8438630000
13	C	-5.0014740000	3.9693060000	0.1211210000
14	C	-4.2147800000	3.2221420000	-0.9748610000
15	C	-0.4945390000	2.5292860000	-0.8696520000
16	C	-1.8657470000	2.7795070000	-1.5057520000
17	C	-2.9292820000	1.5343610000	0.2535900000
18	C	-1.5675100000	1.2672450000	0.9004420000
19	C	0.8062050000	1.0397520000	0.5662530000
20	C	1.9498620000	0.6985420000	-0.4186730000
21	C	6.1372490000	-7.1344200000	-1.2705480000
22	C	5.2470200000	-6.1021320000	-2.0075580000
23	C	5.5387060000	-4.6370170000	-1.6446250000
24	C	4.5301330000	-3.6887860000	-2.3141940000
25	C	4.8971930000	2.3545100000	-3.0247640000
26	C	4.6562650000	3.0845350000	-1.6862260000
27	C	7.0315580000	2.7078650000	-0.9939340000
28	C	7.2476670000	2.8304750000	-2.5248460000
29	C	3.7628270000	1.7364610000	0.9058470000
30	C	5.2092350000	2.1097100000	0.5552330000

1	C	4.2366770000	-0.2266140000	-0.5738150000
2	C	3.9053570000	-1.6267310000	-1.0899570000
3	C	6.0056100000	-1.6450330000	-2.4095970000
4	C	5.7224040000	-0.5877130000	-3.5145510000
5	C	6.6826050000	0.5878840000	-3.3510550000
6	H	-17.3286690000	-2.3997400000	4.2984330000
7	H	-18.7858900000	-1.4598340000	4.0836070000
8	H	-18.8611260000	-3.0229860000	4.8607560000
9	H	-18.1149840000	-4.0642940000	2.7805190000
10	H	-19.6606000000	-3.0673730000	2.5526600000
11	H	-16.8832410000	-2.4028780000	1.7585120000
12	H	-18.4171700000	-1.3833460000	1.5528030000
13	H	-17.7318650000	-3.9683780000	0.2781980000
14	H	-19.2536290000	-2.9328520000	0.0622830000
15	H	-16.4744410000	-2.4013480000	-0.8122960000
16	H	-17.9423040000	-1.2772510000	-0.9406950000
17	H	-17.5374810000	-3.9031160000	-2.2524600000
18	H	-18.9344470000	-2.6993280000	-2.4370680000
19	H	-16.1145640000	-2.3592880000	-3.2780400000
20	H	-17.5654590000	-1.2370640000	-3.5430250000
21	H	-17.0066850000	-3.8936510000	-4.7348700000
22	H	-18.5144390000	-2.8380670000	-4.9519450000
23	H	-17.3435400000	-1.4094300000	-6.3887360000
24	H	-15.2755590000	-1.8564020000	-7.2782720000
25	H	-14.3496130000	-4.0960640000	-6.8865750000
26	H	-15.0446550000	-4.0196180000	-5.1702500000
27	H	-13.5376810000	-2.4254690000	-4.5788430000
28	H	-12.9346410000	-2.2920370000	-6.3261950000
29	H	-12.5360140000	-4.4789720000	-4.3667400000
30	H	-11.9939150000	-4.4174260000	-6.1379100000

1	H	-11.0757580000	-2.8149230000	-3.8223750000
2	H	-10.6118300000	-2.5976670000	-5.6034620000
3	H	-9.9619350000	-4.8087800000	-3.7427420000
4	H	-9.5861700000	-4.7007450000	-5.5543240000
5	H	-8.5503930000	-3.0317560000	-3.3342950000
6	H	-8.1939380000	-2.8883830000	-5.1473270000
7	H	-23.4049320000	-3.9620440000	5.5796530000
8	H	-24.3510700000	-5.4160460000	5.3704470000
9	H	-24.1025320000	-4.3337810000	4.0213740000
10	H	-21.5723950000	-5.0042510000	4.9780390000
11	H	-22.5759260000	-6.5464530000	4.7561430000
12	H	-21.9401320000	-4.4466530000	2.7635870000
13	H	-22.9427620000	-5.9894610000	2.5418270000
14	H	-20.0432180000	-5.7463420000	3.1034660000
15	H	-21.0544290000	-7.2739940000	2.8234080000
16	H	-20.4386470000	-5.1179710000	0.8865730000
17	H	-21.3429700000	-6.7140210000	0.6229020000
18	H	-18.4731750000	-6.2965910000	1.2315790000
19	H	-19.3815090000	-7.8856400000	0.9408010000
20	H	-18.8732610000	-5.6963180000	-0.9901620000
21	H	-19.6798210000	-7.3421840000	-1.2645480000
22	H	-16.8496590000	-6.7974700000	-0.5713950000
23	H	-17.6739340000	-8.4070120000	-0.9771610000
24	H	-17.8630840000	-7.6804180000	-3.2868680000
25	H	-16.2003220000	-6.3407740000	-4.1535080000
26	H	-15.7274900000	-5.4112470000	-1.3232010000
27	H	-15.1151680000	-4.6557620000	-2.9008590000
28	H	-14.1810170000	-7.1068100000	-1.5206780000
29	H	-13.5879410000	-6.3922290000	-3.1245280000
30	H	-13.3177590000	-5.3035700000	-0.3800990000

1	H	-12.7703880000	-4.5393240000	-1.9772890000
2	H	-11.7798820000	-6.9987360000	-0.6539260000
3	H	-11.2469700000	-6.2638600000	-2.2696810000
4	H	-10.8522540000	-5.2406480000	0.4832860000
5	H	-10.4031280000	-4.4150620000	-1.1140220000
6	H	-9.3228210000	-6.9186000000	0.0464070000
7	H	-8.8741960000	-6.0939650000	-1.5515330000
8	H	-8.3020200000	-5.2934190000	1.2389430000
9	H	-8.0519120000	-4.2665380000	-0.2834440000
10	H	-7.4456480000	-5.0659320000	-3.2855350000
11	H	-7.0710310000	-4.8990700000	-5.0928870000
12	H	-4.3167820000	-5.6550000000	-1.1823180000
13	H	-5.1762990000	-7.2445040000	-1.5939120000
14	H	-5.7054510000	-6.3499820000	-3.7282290000
15	H	-2.8034300000	-6.0992990000	-3.0277710000
16	H	-3.5088770000	-6.2981420000	-4.7299780000
17	H	-0.0940100000	-11.3476400000	-4.0407750000
18	H	-1.1657590000	-10.1189590000	-3.9493140000
19	H	0.8627000000	-9.1486960000	-5.4779990000
20	H	0.3919990000	-10.8318370000	-6.0945840000
21	H	-0.9466340000	-8.2929070000	-6.3329430000
22	H	-1.3338160000	-9.9444130000	-7.0794800000
23	H	-0.6962600000	-7.3591820000	-4.6891950000
24	H	14.5875710000	-6.4365630000	-2.3212670000
25	H	12.9186010000	-6.1621910000	-2.7598160000
26	H	13.3268730000	-7.4238820000	-1.6220480000
27	H	14.8081370000	-4.2044820000	-1.5427950000
28	H	13.0400620000	-3.9170170000	-2.0181600000
29	H	14.3461190000	-3.5549800000	0.6185950000
30	H	12.5630920000	-3.3118030000	0.1754460000

1	H	18.5278390000	4.2307480000	-0.5164230000
2	H	20.3196680000	-10.1005960000	14.8056650000
3	H	18.8170900000	-10.6422890000	14.0972180000
4	H	18.9668200000	-10.5097080000	15.8330380000
5	H	19.2872750000	-8.0975230000	15.5944340000
6	H	17.6935500000	-8.6720740000	14.8430130000
7	H	20.2154730000	-7.9117390000	13.4845610000
8	H	18.6223860000	-8.4874970000	12.7327140000
9	H	19.1780300000	-5.9706240000	14.1953070000
10	H	17.5856180000	-6.5462300000	13.4419130000
11	H	28.3834940000	3.7515010000	-13.8592450000
12	H	26.8608540000	3.2015140000	-14.5166230000
13	H	27.5906900000	2.3528520000	-13.1749160000
14	H	27.3546830000	5.0179670000	-12.3949890000
15	H	25.7396790000	4.4346170000	-13.0922440000
16	H	27.3199830000	3.2831630000	-10.8650440000
17	H	25.7036960000	2.7021860000	-11.5613060000
18	H	26.2929980000	5.2057060000	-10.0889590000
19	H	24.6765990000	4.6204020000	-10.7813260000
20	H	26.2617220000	3.4714080000	-8.5561290000
21	H	24.6409710000	2.8942070000	-9.2451170000
22	H	20.1077250000	-5.7799500000	12.0867570000
23	H	18.5169720000	-6.3586830000	11.3322550000
24	H	15.2341280000	2.5733080000	-0.6785710000
25	H	16.4140250000	1.7914150000	0.5177520000
26	H	13.4571370000	1.9623030000	0.7205640000
27	H	14.6615640000	1.0992220000	1.8337340000
28	H	13.6417060000	0.4866800000	-0.6989640000
29	H	18.2275440000	6.5711210000	2.0404450000
30	H	17.3302280000	5.5965930000	3.3365230000

1	H	19.6941130000	6.4145920000	3.7712120000
2	H	18.7555070000	3.5937130000	4.0682870000
3	H	20.2548450000	4.2850820000	4.9101270000
4	H	22.3034940000	4.4883300000	0.7380090000
5	H	20.6309120000	3.9278690000	0.1696310000
6	H	19.6279560000	2.8325470000	6.6308900000
7	H	18.0421440000	2.2780010000	5.8483440000
8	H	18.6792130000	2.5705770000	8.7256970000
9	H	17.1040990000	1.9967170000	7.9355060000
10	H	19.7529000000	0.6664230000	7.9586180000
11	H	18.1708390000	0.0904540000	7.1839940000
12	H	18.8120980000	0.4227910000	10.0586070000
13	H	17.2321240000	-0.1569660000	9.2825500000
14	H	19.8766450000	-1.4940810000	9.3165960000
15	H	18.2919680000	-2.0751180000	8.5511580000
16	H	18.9415990000	-1.7141060000	11.4204470000
17	H	17.3561260000	-2.2936770000	10.6555470000
18	H	19.9946960000	-3.6424340000	10.6942520000
19	H	18.4061520000	-4.2227700000	9.9363350000
20	H	19.0633950000	-3.8435870000	12.8015300000
21	H	17.4738430000	-4.4220400000	12.0442890000
22	H	22.2817080000	6.1556160000	-0.8658720000
23	H	20.6213510000	5.5694730000	-1.4441030000
24	H	23.2276940000	4.1667400000	-1.5976270000
25	H	21.5674700000	3.6093700000	-2.2040050000
26	H	23.2236430000	5.8657240000	-3.1787900000
27	H	21.5804800000	5.2738920000	-3.7988290000
28	H	24.2035170000	3.9006910000	-3.9261530000
29	H	22.5606660000	3.3350190000	-4.5709450000
30	H	24.2149070000	5.6186890000	-5.4836830000

1	H	22.5851610000	5.0275720000	-6.1388210000
2	H	25.2191880000	3.6738130000	-6.2444230000
3	H	23.5897870000	3.1012060000	-6.9166380000
4	H	25.2424480000	5.4027470000	-7.7862890000
5	H	23.6214330000	4.8142860000	-8.4650540000
6	H	-22.2101470000	3.0880590000	7.9343920000
7	H	-21.6551430000	3.5391230000	6.2777040000
8	H	-21.4729040000	4.7101860000	7.6461120000
9	H	-19.8681650000	3.1431790000	8.7674450000
10	H	-20.0538600000	1.9774320000	7.3984830000
11	H	-18.9710000000	4.8662470000	7.1808740000
12	H	-19.1655620000	3.7039630000	5.8098470000
13	H	-17.3589200000	3.2854910000	8.2874930000
14	H	-17.5661070000	2.1246570000	6.9149990000
15	H	-16.5034310000	5.0166100000	6.6928430000
16	H	-16.7349850000	3.8632410000	5.3211350000
17	H	-14.8318630000	3.4416810000	7.7273160000
18	H	-15.0909020000	2.2849910000	6.3590070000
19	H	-14.0766210000	5.1897810000	6.0981740000
20	H	-14.3805030000	4.0420380000	4.7375840000
21	H	-23.0777820000	5.0347820000	4.6371620000
22	H	-24.3049820000	6.0962440000	3.8481390000
23	H	-23.3944750000	4.8911650000	2.8599630000
24	H	-22.2138800000	7.3577940000	4.2953420000
25	H	-22.5299060000	7.2178920000	2.5211980000
26	H	-20.6079730000	5.4349220000	4.1610410000
27	H	-20.9193210000	5.2978840000	2.3843680000
28	H	-19.7610080000	7.7669740000	3.8355240000
29	H	-20.0622030000	7.6358510000	2.0565920000
30	H	-18.1795430000	5.8324460000	3.7098230000

1	H	-18.4571330000	5.7142170000	1.9265690000
2	H	-17.3185990000	8.1605140000	3.4296440000
3	H	-17.5597980000	8.0498210000	1.6399720000
4	H	-15.7777150000	6.1991100000	3.3467240000
5	H	-15.9598530000	6.1151730000	1.5501020000
6	H	-12.5492950000	16.6564140000	-3.7069910000
7	H	-10.9482490000	17.3604370000	-3.2388920000
8	H	-11.7188890000	17.8251170000	-4.8032640000
9	H	-11.4712400000	15.5031980000	-5.6514300000
10	H	-9.8731050000	16.2060010000	-5.1831080000
11	H	-11.5934650000	14.3091790000	-3.4495040000
12	H	-9.9953020000	15.0126910000	-2.9785280000
13	H	-10.5120200000	13.1630470000	-5.4011980000
14	H	-8.9138060000	13.8658870000	-4.9265930000
15	H	-10.6541250000	11.9910310000	-3.1950210000
16	H	-9.0581040000	12.6934300000	-2.7161210000
17	H	-9.5576220000	10.8221100000	-5.1258010000
18	H	-7.9611480000	11.5221340000	-4.6403760000
19	H	-9.7375250000	9.6845360000	-2.9065660000
20	H	-8.1437650000	10.3823930000	-2.4147990000
21	H	-14.8746640000	8.5144420000	3.1632190000
22	H	-14.9772310000	8.4361310000	1.3587730000
23	H	-8.6202930000	8.4717350000	-4.7977280000
24	H	-7.0240650000	9.1615970000	-4.2957170000
25	H	-12.2920080000	3.6475020000	6.9910430000
26	H	-12.6355300000	2.4880780000	5.6423800000
27	H	-11.7421140000	5.4190200000	5.3112120000
28	H	-12.1648690000	4.2670110000	3.9971800000
29	H	-7.8799110000	3.4867730000	4.0762350000
30	H	-9.8563930000	3.9719910000	6.0244410000

1	H	-10.1975530000	2.7906480000	4.7036450000
2	H	-9.5439330000	5.7702080000	4.3503900000
3	H	-13.4131920000	6.5040130000	3.1770860000
4	H	-13.4194660000	6.4655790000	1.3728640000
5	H	-12.4620230000	8.7991710000	3.1545830000
6	H	-12.2822630000	8.7537370000	1.3617990000
7	H	-9.8789370000	8.9822920000	2.1098500000
8	H	-11.1408260000	6.7731140000	3.3906260000
9	H	-8.8633240000	7.3926530000	-2.5595380000
10	H	-7.2764190000	8.0944630000	-2.0567620000
11	H	-7.3117480000	3.7572030000	-3.7237960000
12	H	-7.7362470000	6.1558240000	-4.4132090000
13	H	-6.1251860000	6.7523170000	-3.8671940000
14	H	-8.1248370000	5.0674890000	-2.2121090000
15	H	-10.8526010000	4.3919750000	2.4090830000
16	H	-9.3616640000	3.5544790000	2.1983790000
17	H	-11.2984740000	5.9029530000	0.9191890000
18	H	-10.2213440000	7.2466060000	0.5043060000
19	H	-6.4651920000	6.5251080000	-0.8134790000
20	H	-5.1704410000	5.7375090000	-1.6584990000
21	H	-9.1367430000	4.4103360000	-0.0202700000
22	H	-8.5301010000	6.0854240000	-0.2105490000
23	H	-7.2023010000	3.9538410000	1.5403240000
24	H	-6.6563050000	5.6568080000	1.3872590000
25	H	-5.1318860000	3.2617100000	0.9671340000
26	H	-4.4012300000	4.8304620000	0.4945630000
27	H	-4.7851420000	2.3392990000	-1.3432260000
28	H	-4.0842990000	3.8999790000	-1.8417450000
29	H	-0.2506710000	3.3825120000	-0.1950810000
30	H	0.2471090000	2.5057360000	-1.6924910000

1	H	-1.8166390000	3.7526820000	-2.0427730000
2	H	-2.0992030000	1.9788410000	-2.2445470000
3	H	-3.6955460000	1.5398120000	1.0564270000
4	H	-3.1749080000	0.7104870000	-0.4555800000
5	H	-1.3749050000	2.0564180000	1.6637790000
6	H	-1.6160690000	0.2827620000	1.4164340000
7	H	1.0552960000	1.9247860000	1.1907500000
8	H	0.7190870000	0.1689950000	1.2551480000
9	H	2.0920760000	1.4907120000	-1.1740070000
10	H	1.6153000000	-0.1986280000	-0.9750650000
11	H	8.8703150000	-8.9627960000	-1.4784510000
12	H	8.3632610000	-7.9587810000	-3.6713980000
13	H	6.5221460000	-9.6078430000	-3.6031340000
14	H	6.1409180000	-10.0943110000	-0.0008970000
15	H	5.0826190000	-7.1439970000	0.3707740000
16	H	4.1892850000	-6.3142070000	-1.7356620000
17	H	5.3508020000	-6.2368700000	-3.1066910000
18	H	5.4554000000	-4.5082950000	-0.5435780000
19	H	6.5635900000	-4.3748460000	-1.9589610000
20	H	3.5165700000	-4.0183670000	-2.0000590000
21	H	4.5676080000	-3.7779880000	-3.4218500000
22	H	4.6816290000	3.0692780000	-3.8481810000
23	H	4.1435240000	1.5767600000	-3.1658880000
24	H	4.8951810000	4.1593930000	-1.8467260000
25	H	3.5909410000	3.0740420000	-1.4030320000
26	H	7.5577430000	3.4880980000	-0.4039800000
27	H	7.4830240000	1.7229090000	-0.7357430000
28	H	7.0303920000	3.8591390000	-2.8887990000
29	H	8.3076170000	2.6106470000	-2.7780750000
30	H	6.5346390000	-1.2147090000	-1.5327130000

H	6.7307640000	-2.3604280000	-2.8510600000
H	4.6638220000	-0.3172660000	-3.4784880000
H	5.8313920000	-1.0617420000	-4.5137570000
H	3.1176180000	2.6215680000	0.7266350000
H	3.7234760000	1.6055630000	2.0121210000
H	3.0458570000	-0.2197770000	1.0751800000
H	4.4297380000	0.4105360000	-1.4242250000
H	5.1779710000	-0.3533860000	0.0022970000

6.12. Branched amine core based bone-targeting ionizable lipids 'Type 5'

Type5-B1-C12

P	-11.1677480000	0.2107110000	0.8993720000
P	4.1744840000	-9.0629500000	-1.2238000000
P	-5.2473170000	-14.3926680000	-1.0572550000
P	-2.5326930000	-13.7451050000	0.1681670000
O	-11.2212440000	-10.1324420000	-1.9761900000
O	-10.6406870000	-5.2505990000	-2.8696960000
O	-11.6223130000	-0.5325600000	2.4036690000
O	-10.1632370000	1.2485330000	1.2570260000
O	-10.6902470000	-0.8385800000	-0.2193050000
O	-12.6332090000	0.8769430000	0.6144200000
O	-14.9859420000	2.0213380000	1.1550080000
O	-13.7998250000	3.0632080000	2.8035450000
O	-14.3094470000	2.7664300000	-2.2830760000
O	-14.1315120000	3.1133400000	-4.5017520000
O	-10.5345550000	-6.6964870000	-1.1426110000
O	7.8589170000	-4.0755860000	-3.0333730000
O	8.2208580000	-5.0446650000	-1.0027780000
O	7.9946020000	-4.8519690000	3.0610400000
O	6.9308310000	-5.8427840000	1.3493050000

1	O	2.6492350000	-8.6267280000	-0.5171320000
2	O	5.4775330000	-8.2068160000	-0.9455150000
3	O	4.0893800000	-10.6381060000	-0.8098280000
4	O	3.6648440000	-9.1178530000	-2.6201040000
5	O	-4.1975820000	10.4724720000	-1.3129990000
6	O	-2.2914310000	11.4662220000	3.6123120000
7	O	5.9147730000	9.2078790000	0.1286570000
8	O	3.6214130000	3.9308140000	-0.0754210000
9	O	-6.5481750000	4.7117570000	-1.2731450000
10	O	-4.8686240000	1.0431750000	0.4035040000
11	O	-2.8698440000	-8.1105090000	-2.1176350000
12	O	-5.9983580000	-14.7877070000	0.4600340000
13	O	-6.5310070000	-14.0182130000	-2.1643230000
14	O	-4.7100860000	-15.7517220000	-1.5832000000
15	O	-3.0012850000	-13.9023400000	1.8326210000
16	O	-1.8437130000	-15.2727390000	-0.2928710000
17	O	-1.3041520000	-12.7974820000	0.2181930000
18	O	-3.3509100000	-12.9264000000	-2.2746570000
19	O	-0.4176070000	-4.1332370000	-0.4526420000
20	N	-10.3389760000	-4.4341280000	-0.7873360000
21	N	2.0360600000	-10.3513190000	-2.0993820000
22	N	-2.2041490000	9.3905970000	0.4783670000
23	N	3.1085620000	6.7434860000	0.3104480000
24	N	-0.6255890000	5.9344340000	0.3352100000
25	N	-3.5152480000	3.4169560000	0.5406920000
26	N	-3.3254810000	-8.1926940000	0.0814110000
27	N	-2.0883940000	-2.8029730000	0.2536340000
28	N	-3.1869410000	-0.3051660000	-0.2377730000
29	C	-11.2706440000	-11.3513750000	-1.2658760000
30	C	-10.9191930000	-9.0421150000	-1.1400410000

1	C	-10.8760940000	-7.7725160000	-1.9776130000
2	C	-23.3667160000	-6.9382140000	-14.0705620000
3	C	-22.4233610000	-5.7517400000	-13.8323200000
4	C	-22.0664830000	-5.5940820000	-12.3456330000
5	C	-21.1204700000	-4.4065950000	-12.1043120000
6	C	-15.9978700000	-4.4942760000	17.5415730000
7	C	-15.3791120000	-3.4360800000	16.6184990000
8	C	-15.8147200000	-3.6263320000	15.1567970000
9	C	-15.1976630000	-2.5658270000	14.2305000000
10	C	-15.6354970000	-2.7513830000	12.7683400000
11	C	-20.7632820000	-4.2464070000	-10.6174320000
12	C	-10.7950820000	-2.2118020000	0.0554120000
13	C	-10.2967770000	-3.0147740000	-1.1450510000
14	C	-12.7492040000	2.2154100000	0.2062300000
15	C	-14.2375380000	2.5660240000	0.0988380000
16	C	-14.8455320000	2.0458600000	-1.2054670000
17	C	-14.5490470000	2.1863420000	2.4366430000
18	C	-15.0890880000	1.0725140000	3.3198310000
19	C	-14.5933160000	2.4657470000	-3.5850750000
20	C	-15.5200170000	1.2745580000	-3.8108850000
21	C	-15.8839150000	1.0541270000	-5.2904180000
22	C	-16.8321280000	-0.1407380000	-5.4876130000
23	C	-17.1947290000	-0.3373990000	-6.9688540000
24	C	-18.1453210000	-1.5263570000	-7.1834910000
25	C	-18.5062180000	-1.7055090000	-8.6674460000
26	C	-19.4558000000	-2.8928560000	-8.8950780000
27	C	-19.8149730000	-3.0596780000	-10.3808410000
28	C	-14.6086580000	1.1636160000	4.7766960000
29	C	-15.1865080000	0.0402430000	5.6531660000
30	C	-14.7202330000	0.1646790000	7.1130600000

1	C	-15.3136740000	-0.9331840000	8.0108190000
2	C	-14.8605300000	-0.7795070000	9.4721390000
3	C	-15.4659730000	-1.8582430000	10.3847860000
4	C	-15.0222860000	-1.6845610000	11.8467300000
5	C	-10.5061530000	-5.4464710000	-1.6847170000
6	C	20.1616990000	1.0781720000	-3.1825830000
7	C	20.4222370000	-0.3740850000	-2.7639890000
8	C	19.8509650000	-0.6883410000	-1.3719890000
9	C	20.1439620000	-2.1389940000	-0.9594020000
10	C	19.5703200000	-2.4902420000	0.4221630000
11	C	19.9674980000	-3.9118240000	0.8514040000
12	C	19.3288680000	-4.3173180000	2.1887860000
13	C	19.6567940000	-5.7721640000	2.5558330000
14	C	19.0853860000	-6.1280590000	3.9046860000
15	C	17.8891280000	-6.6859440000	4.1509820000
16	C	16.8360850000	-7.0814200000	3.1438090000
17	C	15.5451780000	-6.2779860000	3.3711640000
18	C	14.3795000000	-6.7590430000	2.4901330000
19	C	13.0875270000	-5.9831980000	2.7973270000
20	C	11.8671550000	-6.5224150000	2.0323620000
21	C	10.5769860000	-5.7937690000	2.4479030000
22	C	25.2685720000	0.1094550000	-5.7884950000
23	C	23.9809990000	-0.6972140000	-6.0017700000
24	C	23.7974710000	-1.7759050000	-4.9226170000
25	C	22.5077780000	-2.5849010000	-5.1336080000
26	C	22.3003270000	-3.6329770000	-4.0283400000
27	C	20.9636140000	-4.3750660000	-4.1845420000
28	C	20.7433760000	-5.4089440000	-3.0683430000
29	C	19.3673780000	-6.0832590000	-3.1786420000
30	C	19.1283280000	-7.0170190000	-2.0181820000

1	C	18.2276300000	-6.8577610000	-1.0347920000
2	C	17.2611460000	-5.7097040000	-0.8766750000
3	C	15.8823310000	-6.0877440000	-1.4322560000
4	C	14.8593690000	-4.9548410000	-1.2520600000
5	C	13.4737770000	-5.3719630000	-1.7671720000
6	C	12.4198460000	-4.2699220000	-1.5750610000
7	C	11.0290540000	-4.7544050000	-2.0120800000
8	C	9.9450150000	-3.6799200000	-1.8344800000
9	C	8.5583290000	-4.2593220000	-2.0632240000
10	C	9.3294270000	-6.3307470000	1.7222520000
11	C	8.0441770000	-5.6020510000	2.1075880000
12	C	6.9966040000	-5.7240640000	-1.0397710000
13	C	6.8898000000	-6.6285990000	0.1904280000
14	C	5.5543930000	-7.3798400000	0.1879740000
15	C	1.7685080000	-11.1091580000	-0.8042580000
16	C	3.0885680000	-11.1914500000	-0.0078640000
17	C	8.6747140000	12.9334970000	-4.5925410000
18	C	7.5596060000	13.5836120000	-3.7783800000
19	C	6.6937580000	12.5263840000	-3.0843530000
20	C	5.5704360000	13.1810110000	-2.2708290000
21	C	4.7037140000	12.1187360000	-1.5862870000
22	C	3.5696050000	12.7592750000	-0.7776740000
23	C	2.6922460000	11.6832860000	-0.1277130000
24	C	-0.4172730000	20.3394140000	-8.5548610000
25	C	-0.6425780000	18.8307840000	-8.6004610000
26	C	-0.9409490000	18.2734780000	-7.2038970000
27	C	-1.1651930000	16.7574350000	-7.2530760000
28	C	-1.4621030000	16.2004140000	-5.8557400000
29	C	-1.6823610000	14.6835970000	-5.9018260000
30	C	-1.9747560000	14.1294320000	-4.5025550000

1	C	-3.5589170000	10.4636940000	10.8760950000
2	C	-3.9119000000	10.6127050000	9.3989920000
3	C	-4.1041800000	9.2443630000	8.7353520000
4	C	-4.4539650000	9.3971630000	7.2502170000
5	C	-4.6379270000	8.0276940000	6.5857600000
6	C	-4.9712610000	8.1780930000	5.0969200000
7	C	-5.1288350000	6.8071850000	4.4279570000
8	C	13.4454530000	1.9582030000	0.3848350000
9	C	12.2710620000	2.8079660000	0.8604290000
10	C	11.7890710000	3.7586450000	-0.2427480000
11	C	10.6098400000	4.6074950000	0.2533460000
12	C	10.1023260000	5.6346500000	-0.7735730000
13	C	9.5466450000	4.9858850000	-2.0623830000
14	C	8.9277590000	5.9844230000	-3.0666060000
15	C	13.2756890000	3.5293810000	9.7619010000
16	C	12.1968460000	2.7966260000	8.9695300000
17	C	11.4843170000	3.7446550000	7.9984570000
18	C	10.4003360000	3.0069500000	7.2031850000
19	C	9.6899770000	3.9586690000	6.2340440000
20	C	8.6040170000	3.2282130000	5.4346140000
21	C	7.9019900000	4.1896190000	4.4693150000
22	C	-5.4226120000	6.9585430000	2.9310250000
23	C	-2.1873630000	12.6111910000	-4.5424350000
24	C	1.5320190000	12.3071940000	0.6568100000
25	C	7.4387860000	6.3431980000	-2.8484270000
26	C	-2.4706080000	12.0614730000	-3.1394200000
27	C	-2.6720650000	10.5362230000	-3.1747830000
28	C	-3.0036640000	9.9153900000	-1.8021420000
29	C	0.6148650000	11.2196010000	1.2302170000
30	C	-0.5739500000	11.8407500000	1.9823040000

1	C	-1.5987200000	10.8304370000	2.5648580000
2	C	7.1141610000	7.0143250000	-1.5150000000
3	C	5.6241330000	7.3607260000	-1.3867990000
4	C	5.3356220000	7.9322400000	0.0163230000
5	C	6.8157670000	3.4732160000	3.6570250000
6	C	6.1355510000	4.4502500000	2.6918140000
7	C	5.0652980000	3.7479720000	1.8381900000
8	C	4.4526790000	4.6805710000	0.7732780000
9	C	-1.8691590000	10.0946720000	-0.7783850000
10	C	-2.6716280000	10.2529260000	1.5854680000
11	C	3.6194020000	5.8304850000	1.3788360000
12	C	3.8258430000	8.0496460000	0.3015280000
13	C	-5.5132680000	5.5928340000	2.2388260000
14	C	-5.7599370000	5.7772340000	0.7263050000
15	C	-5.7948330000	4.4705410000	-0.1090370000
16	C	-1.3680520000	8.2446160000	0.8907890000
17	C	-4.4282720000	3.8536770000	-0.5565030000
18	C	1.6575840000	6.9951290000	0.4885540000
19	C	-1.2863300000	7.1570760000	-0.1963480000
20	C	0.8105030000	5.8119740000	-0.0170290000
21	C	-2.5563500000	4.5019450000	0.9083980000
22	C	-1.3705080000	4.7168230000	-0.0638640000
23	C	-2.7910290000	2.1636240000	0.1931260000
24	C	-3.6817970000	0.9261870000	0.1282050000
25	C	-2.6910960000	-7.6532000000	-0.9977080000
26	C	-1.7582820000	-6.4805140000	-0.8314120000
27	C	-2.4997880000	-5.2384270000	-0.3079050000
28	C	-3.8769710000	-13.1153610000	-0.9864590000
29	C	-4.4947490000	-11.7896790000	-0.4896530000
30	C	-3.5166760000	-10.6035860000	-0.4432420000

1	C	-4.2395080000	-9.3186170000	-0.0076270000
2	C	-1.6031020000	-4.0198810000	-0.1682740000
3	C	-1.2239620000	-1.6276120000	0.3472480000
4	C	-1.7807930000	-0.5353280000	-0.5714910000
5	C	-4.0514100000	-1.4830960000	-0.3140810000
6	C	-3.4909640000	-2.5762670000	0.6005020000
7	H	-12.0255360000	-11.2932460000	-0.5097940000
8	H	-10.3211990000	-11.5363580000	-0.8084540000
9	H	-11.5026420000	-12.1484390000	-1.9409800000
10	H	-11.7374580000	-8.9371370000	-0.4586280000
11	H	-9.9292180000	-9.2017860000	-0.7667380000
12	H	-11.8721360000	-7.5939370000	-2.3253350000
13	H	-10.0770810000	-7.8886800000	-2.6797410000
14	H	-12.4986780000	-0.2331830000	2.6565700000
15	H	-24.2732120000	-6.7888650000	-13.5220530000
16	H	-22.8951610000	-7.8405120000	-13.7413300000
17	H	-23.5896150000	-7.0126390000	-15.1144380000
18	H	-22.9714970000	-4.8737960000	-14.1037260000
19	H	-21.5098530000	-5.9892350000	-14.3363030000
20	H	-22.9801050000	-5.3560750000	-11.8420970000
21	H	-21.5192750000	-6.4726460000	-12.0743600000
22	H	-21.6675490000	-3.5283010000	-12.3767180000
23	H	-20.2067160000	-4.6452130000	-12.6073190000
24	H	-16.5934790000	-4.0119120000	18.2882150000
25	H	-15.2186010000	-5.0548450000	18.0142270000
26	H	-16.6129880000	-5.1539460000	16.9659280000
27	H	-15.7803580000	-2.4945650000	16.9306700000
28	H	-14.3220800000	-3.6007630000	16.6400620000
29	H	-16.8720270000	-3.4633400000	15.1358960000
30	H	-15.4121750000	-4.5672580000	14.8445220000

1	H	-15.5977800000	-1.6246930000	14.5452600000
2	H	-14.1406570000	-2.7310020000	14.2493310000
3	H	-16.6932800000	-2.5912300000	12.7497040000
4	H	-15.2308030000	-3.6897500000	12.4511830000
5	H	-21.6762770000	-4.0053760000	-10.1141980000
6	H	-20.2175610000	-5.1252190000	-10.3439750000
7	H	-10.1241140000	-2.4166820000	0.8633270000
8	H	-11.8381650000	-2.4268080000	0.1586090000
9	H	-9.2684640000	-2.7574320000	-1.2908210000
10	H	-11.0022100000	-2.8685320000	-1.9361750000
11	H	-9.4303640000	-4.6065190000	-0.4069350000
12	H	-12.3423600000	2.8177300000	0.9914610000
13	H	-12.3417950000	2.2771590000	-0.7812440000
14	H	-14.2830270000	3.6345220000	0.1326570000
15	H	-14.5196210000	1.0328360000	-1.3171180000
16	H	-15.8910620000	2.2708430000	-1.1715530000
17	H	-16.1497500000	1.2101010000	3.3509180000
18	H	-14.6786180000	0.1640110000	2.9311980000
19	H	-16.4369830000	1.5179560000	-3.3160810000
20	H	-14.9645870000	0.4098390000	-3.5131300000
21	H	-16.4308710000	1.9218400000	-5.5950730000
22	H	-14.9716620000	0.7953850000	-5.7861450000
23	H	-17.7428640000	0.1121800000	-4.9861230000
24	H	-16.2837550000	-1.0114010000	-5.1941300000
25	H	-17.7393130000	0.5361150000	-7.2609160000
26	H	-16.2828040000	-0.5910390000	-7.4678130000
27	H	-19.0564020000	-1.2769160000	-6.6808870000
28	H	-17.5999380000	-2.4019590000	-6.8992750000
29	H	-19.0512620000	-0.8291230000	-8.9498870000
30	H	-17.5937960000	-1.9527860000	-9.1686860000

1	H	-20.3677600000	-2.6484530000	-8.3915940000
2	H	-18.9103170000	-3.7706550000	-8.6179190000
3	H	-20.3607220000	-2.1816150000	-10.6566370000
4	H	-18.9020000000	-3.3020510000	-10.8834690000
5	H	-15.0056180000	2.0783680000	5.1647050000
6	H	-13.5503820000	1.0076330000	4.7517690000
7	H	-16.2470390000	0.1819340000	5.6630960000
8	H	-14.7723390000	-0.8739030000	5.2820870000
9	H	-15.1156600000	1.0910130000	7.4742270000
10	H	-13.6627110000	0.0020590000	7.1029880000
11	H	-16.3731640000	-0.7836110000	8.0076350000
12	H	-14.9029800000	-1.8586570000	7.6647970000
13	H	-15.2571380000	0.1547860000	9.8108110000
14	H	-13.8033670000	-0.9447160000	9.4756350000
15	H	-16.5244690000	-1.7022810000	10.3720780000
16	H	-15.0587650000	-2.7917470000	10.0566810000
17	H	-15.4206110000	-0.7456420000	12.1702550000
18	H	-13.9652880000	-1.8503440000	11.8598190000
19	H	19.1072940000	1.2584660000	-3.2075780000
20	H	20.6201350000	1.7401750000	-2.4779640000
21	H	20.5758970000	1.2490720000	-4.1542480000
22	H	19.8796080000	-0.9855830000	-3.4542960000
23	H	21.4842170000	-0.4746540000	-2.6804240000
24	H	18.7874020000	-0.6083220000	-1.4576100000
25	H	20.3804430000	-0.0657870000	-0.6813510000
26	H	19.6251640000	-2.7574550000	-1.6617230000
27	H	21.2074020000	-2.2115590000	-0.8659720000
28	H	18.5058020000	-2.4945830000	0.3140810000
29	H	20.0345830000	-1.8243370000	1.1192510000
30	H	19.5587730000	-4.5708070000	0.1141210000

1	H	21.0237330000	-3.8877490000	1.0207760000
2	H	18.2699520000	-4.2761660000	2.0407860000
3	H	19.7884090000	-3.7074600000	2.9383160000
4	H	19.1515650000	-6.3868840000	1.8404580000
5	H	20.7209270000	-5.8282270000	2.6526810000
6	H	19.6976650000	-5.9134660000	4.7555470000
7	H	17.6553790000	-6.8714680000	5.1785230000
8	H	16.5962910000	-8.1056750000	3.3395140000
9	H	17.2093570000	-6.7934460000	2.1832680000
10	H	15.7600060000	-5.2778070000	3.0574910000
11	H	15.2511280000	-6.4702060000	4.3818500000
12	H	14.6438640000	-6.5174660000	1.4818410000
13	H	14.1932070000	-7.7761230000	2.7653480000
14	H	13.2470500000	-4.9888490000	2.4357710000
15	H	12.8829350000	-6.1489800000	3.8344190000
16	H	12.0286320000	-6.2853170000	1.0015320000
17	H	11.7482790000	-7.5419430000	2.3345720000
18	H	10.6929390000	-4.7797050000	2.1267660000
19	H	10.4337530000	-6.0133820000	3.4852820000
20	H	25.0240810000	1.1423340000	-5.6532760000
21	H	25.9025610000	0.0020020000	-6.6437210000
22	H	25.7770160000	-0.2534080000	-4.9197520000
23	H	23.1716100000	-0.0084030000	-5.8779590000
24	H	24.1033790000	-1.2179080000	-6.9284850000
25	H	23.6759620000	-1.2548800000	-3.9959740000
26	H	24.6067930000	-2.4646970000	-5.0469710000
27	H	21.6997360000	-1.8894630000	-5.0423130000
28	H	22.6427730000	-3.1317720000	-6.0433370000
29	H	22.2355040000	-3.0896470000	-3.1088340000
30	H	23.0675700000	-4.3664580000	-4.1634040000

1	H	20.1969300000	-3.6382950000	-4.0651070000
2	H	21.0346910000	-4.9302460000	-5.0964760000
3	H	20.7361220000	-4.8611170000	-2.1492490000
4	H	21.4686140000	-6.1796950000	-3.2260530000
5	H	18.6399720000	-5.3030540000	-3.0946140000
6	H	19.3984630000	-6.6933860000	-4.0570940000
7	H	19.7381310000	-7.8951410000	-1.9740980000
8	H	18.1916800000	-7.6226820000	-0.2874610000
9	H	17.6295200000	-4.9142760000	-1.4902670000
10	H	17.1353630000	-5.5596650000	0.1752600000
11	H	16.0086370000	-6.2118800000	-2.4874980000
12	H	15.5328360000	-6.9042600000	-0.8355530000
13	H	15.1852840000	-4.1506690000	-1.8781480000
14	H	14.7573240000	-4.8043970000	-0.1976160000
15	H	13.5767400000	-5.5040390000	-2.8239860000
16	H	13.1625870000	-6.1913300000	-1.1534140000
17	H	12.6870020000	-3.4760110000	-2.2408180000
18	H	12.3602270000	-4.0880950000	-0.5223100000
19	H	11.0957690000	-4.9373660000	-3.0642090000
20	H	10.7694760000	-5.5505600000	-1.3460050000
21	H	10.1034820000	-2.9590590000	-2.6091720000
22	H	9.9819950000	-3.3792990000	-0.8082440000
23	H	9.4829970000	-6.1262100000	0.6832720000
24	H	9.2066360000	-7.3397780000	2.0564340000
25	H	6.2283890000	-4.9847780000	-0.9492080000
26	H	7.0233580000	-6.3657530000	-1.8955860000
27	H	7.7022780000	-7.3244740000	0.1672760000
28	H	4.7863350000	-6.6422110000	0.0836180000
29	H	5.5631330000	-8.0261740000	1.0406620000
30	H	1.8609850000	-10.9520980000	-2.8793910000

1	H	2.9893120000	-10.0497360000	-2.1182820000
2	H	1.0913860000	-10.5143530000	-0.2275320000
3	H	1.5037230000	-12.1096720000	-1.0758460000
4	H	2.9824640000	-10.5593670000	0.8489390000
5	H	3.3195120000	-12.2286480000	0.1177770000
6	H	2.7006030000	-8.7188930000	0.4370510000
7	H	9.2845180000	13.7206990000	-5.0838400000
8	H	9.3349730000	12.3320980000	-3.9319330000
9	H	8.2457760000	12.2745330000	-5.3774310000
10	H	6.9284770000	14.1948940000	-4.4598170000
11	H	8.0144990000	14.2533970000	-3.0163510000
12	H	6.2472390000	11.8575530000	-3.8532540000
13	H	7.3308990000	11.9160060000	-2.4066060000
14	H	4.9343060000	13.7908330000	-2.9497910000
15	H	6.0135230000	13.8485230000	-1.4990530000
16	H	4.2678490000	11.4506730000	-2.3621370000
17	H	5.3388590000	11.5123880000	-0.9068960000
18	H	2.9424440000	13.3766780000	-1.4583420000
19	H	3.9982220000	13.4169130000	0.0102950000
20	H	2.2778150000	11.0314150000	-0.9288500000
21	H	3.3086200000	11.0623200000	0.5582190000
22	H	-0.2036550000	20.7150120000	-9.5776950000
23	H	0.4465950000	20.5811200000	-7.8995210000
24	H	-1.3232880000	20.8536600000	-8.1691050000
25	H	-1.4968030000	18.6159720000	-9.2790530000
26	H	0.2696670000	18.3444520000	-9.0098520000
27	H	-1.8523800000	18.7666620000	-6.7991290000
28	H	-0.0844090000	18.4959460000	-6.5297470000
29	H	-2.0218290000	16.5341830000	-7.9268720000
30	H	-0.2534550000	16.2650670000	-7.6579870000

1	H	-2.3742780000	16.6918050000	-5.4507690000
2	H	-0.6055800000	16.4256790000	-5.1823170000
3	H	-0.7696660000	14.1932380000	-6.3068530000
4	H	-2.5392790000	14.4561200000	-6.5738160000
5	H	-2.8883160000	14.6179940000	-4.0972520000
6	H	-1.1178270000	14.3611520000	-3.8318640000
7	H	-4.3726850000	9.9344870000	11.4162630000
8	H	-2.6128570000	9.8927960000	10.9913830000
9	H	-3.4251660000	11.4681110000	11.3301660000
10	H	-4.8484880000	11.2056640000	9.3114400000
11	H	-3.0917150000	11.1635800000	8.8887310000
12	H	-4.9237140000	8.6970680000	9.2516950000
13	H	-3.1647830000	8.6560870000	8.8318790000
14	H	-5.3938620000	9.9838180000	7.1507770000
15	H	-3.6334970000	9.9458000000	6.7365370000
16	H	-5.4599670000	7.4768470000	7.0939390000
17	H	-3.6969640000	7.4434360000	6.6913710000
18	H	-5.9143860000	8.7566340000	4.9832130000
19	H	-4.1475100000	8.7346200000	4.5967730000
20	H	-5.9555880000	6.2441790000	4.9142730000
21	H	-4.1825310000	6.2357770000	4.5552700000
22	H	13.7706970000	1.2821150000	1.2035120000
23	H	14.3003340000	2.6070200000	0.0985100000
24	H	13.1463410000	1.3405160000	-0.4886600000
25	H	11.4398120000	2.1334770000	1.1619550000
26	H	12.5916540000	3.3960230000	1.7481610000
27	H	11.4797160000	3.1535000000	-1.1206780000
28	H	12.6239130000	4.4305330000	-0.5401380000
29	H	9.7682100000	3.9433700000	0.5508000000
30	H	10.9391190000	5.1699030000	1.1551720000

1	H	9.3406570000	6.2275640000	-0.2487440000
2	H	10.9218770000	6.3369700000	-1.0408000000
3	H	8.8124780000	4.1802660000	-1.8439570000
4	H	10.4197980000	4.5243290000	-2.5699460000
5	H	8.9455040000	5.4895730000	-4.0628990000
6	H	9.5647860000	6.8922120000	-3.1425280000
7	H	12.8257600000	4.3525550000	10.3569620000
8	H	13.7743750000	2.8205250000	10.4559190000
9	H	14.0398730000	3.9513970000	9.0748130000
10	H	11.4587030000	2.3667540000	9.6813500000
11	H	12.6704140000	1.9663830000	8.4015400000
12	H	11.0169020000	4.5746170000	8.5733680000
13	H	12.2293070000	4.1739860000	7.2923790000
14	H	9.6549310000	2.5779150000	7.9087330000
15	H	10.8673740000	2.1776170000	6.6272780000
16	H	9.2245740000	4.7883690000	6.8110110000
17	H	10.4367390000	4.3881170000	5.5297800000
18	H	7.8548660000	2.7992720000	6.1362170000
19	H	9.0685810000	2.3999160000	4.8553870000
20	H	7.4391870000	5.0184590000	5.0495440000
21	H	8.6553970000	4.6189560000	3.7718340000
22	H	-6.3728350000	7.5187500000	2.7886000000
23	H	-4.5939220000	7.5347880000	2.4641170000
24	H	-3.0448980000	12.3762600000	-5.2108600000
25	H	-1.2729440000	12.1246380000	-4.9484210000
26	H	0.9364550000	12.9471100000	-0.0318880000
27	H	1.9318880000	12.9405130000	1.4787820000
28	H	6.8337350000	5.4131900000	-2.9317730000
29	H	7.1345240000	7.0250230000	-3.6725620000
30	H	-3.3858150000	12.5469790000	-2.7355740000

H	-1.6092680000	12.3155680000	-2.4871630000
H	-1.7545090000	10.0538720000	-3.5784570000
H	-3.5126380000	10.3050110000	-3.8657380000
H	-4.6534580000	9.7683010000	-0.7815080000
H	-3.1417550000	8.8234070000	-1.9867960000
H	0.2825810000	10.6046640000	0.3913700000
H	1.1883500000	10.5649680000	1.9227580000
H	-2.7731900000	12.2476060000	3.2316290000
H	-1.1136160000	12.5874140000	1.3598150000
H	-0.1162390000	12.3862660000	2.8378840000
H	-1.0348190000	9.9995000000	3.0396040000
H	7.3250730000	6.2903460000	-0.7067330000
H	7.7330440000	7.9298750000	-1.3966540000
H	6.8051270000	9.0984850000	0.5543750000
H	5.0394120000	6.4311860000	-1.5445890000
H	5.3110960000	8.0866110000	-2.1689030000
H	5.7668090000	7.2646870000	0.7953150000
H	6.0554240000	3.0448600000	4.3466050000
H	7.2808590000	2.6462070000	3.0762740000
H	5.6817860000	5.2706630000	3.2842930000
H	6.9086990000	4.8796590000	2.0160060000
H	4.2038490000	3.5109620000	-0.7614460000
H	4.2662310000	3.3221820000	2.4837400000
H	5.5623080000	2.9092400000	1.3019030000
H	5.2945470000	5.1012940000	0.1899610000
H	-0.9337040000	9.6987540000	-1.2247620000
H	-1.7105720000	11.1754870000	-0.5865830000
H	-3.2913090000	11.0834190000	1.1814530000
H	-3.3639890000	9.6394090000	2.2010170000
H	2.7966660000	5.3557650000	1.9588410000

1	H	4.1911590000	6.4012780000	2.1383570000
2	H	3.3973160000	8.7129140000	-0.4827730000
3	H	3.6860970000	8.5557340000	1.2845980000
4	H	-6.3432900000	4.9962580000	2.6764640000
5	H	-4.5828860000	5.0522660000	2.4570390000
6	H	-6.0886740000	5.4243970000	-1.7912000000
7	H	-6.7672380000	6.2466600000	0.6616990000
8	H	-5.0399240000	6.4848750000	0.2606060000
9	H	-6.3633430000	3.7131370000	0.4732380000
10	H	-1.8415080000	7.7713460000	1.7799380000
11	H	-0.3685420000	8.5887960000	1.1994720000
12	H	-4.7137950000	2.9829060000	-1.1867600000
13	H	-3.9127090000	4.5474090000	-1.2539460000
14	H	1.4409090000	7.1968970000	1.5625470000
15	H	1.3457490000	7.8845630000	-0.1010630000
16	H	-2.3411600000	6.9505880000	-0.4849670000
17	H	-0.7863590000	7.5038500000	-1.1247860000
18	H	0.9434410000	5.6986690000	-1.1163730000
19	H	1.1943990000	4.8897930000	0.4649390000
20	H	-3.0936760000	5.4600610000	0.9901560000
21	H	-2.1264980000	4.2953800000	1.9144570000
22	H	-1.6970850000	4.8038910000	-1.1230750000
23	H	-0.7226400000	3.8154230000	-0.0006120000
24	H	-0.9410640000	-6.7649070000	-0.1336560000
25	H	-1.3079180000	-6.2326320000	-1.8187170000
26	H	-3.3118890000	-4.9958520000	-1.0278440000
27	H	-2.9422260000	-5.4660900000	0.6848990000
28	H	-5.4505470000	-15.5188410000	0.8419960000
29	H	-7.0974860000	-13.3655530000	-1.6808860000
30	H	-3.3182960000	-14.8359020000	1.9204430000

H	-1.2822560000	-15.0736220000	-1.0840980000
H	-2.9991390000	-13.8013140000	-2.5828980000
H	-5.3196290000	-11.5174350000	-1.1851080000
H	-4.9320760000	-11.9435320000	0.5215280000
H	-3.0696110000	-10.4679470000	-1.4487400000
H	-2.7073210000	-10.8017560000	0.2860630000
H	-5.0660150000	-9.0721000000	-0.7096250000
H	-4.6820690000	-9.4963880000	0.9975600000
H	-3.1691230000	-7.7772030000	1.0120920000
H	-0.1706370000	-1.8369860000	0.0632390000
H	-1.2218080000	-1.2595750000	1.3967630000
H	-1.1431090000	0.3645890000	-0.4821230000
H	-1.7146320000	-0.8848070000	-1.6253050000
H	-4.0552070000	-1.8593520000	-1.3607880000
H	-5.1045700000	-1.2715580000	-0.0309350000
H	-3.5508340000	-2.2348750000	1.6572180000
H	-4.1263920000	-3.4781310000	0.5106770000
H	-4.0897540000	3.2004650000	1.3875060000
H	-2.0149110000	1.9567930000	0.9642300000
H	-2.3021180000	2.2893110000	-0.7986170000

6.13. Piperazine amine core based bone-targeting ionizable lipids 'Type 5'

Type5-P1-C12

P	-0.3388340000	12.5465610000	-3.1277130000
P	-14.3693050000	-4.7748150000	-0.1403300000
P	-11.8609360000	11.2924010000	-1.3407740000
P	-10.6331500000	10.2364170000	-3.9203470000
O	3.7379770000	10.0098460000	1.1929780000
O	4.4047450000	10.2141560000	-0.9766010000
O	5.3191790000	8.4627080000	-4.5402410000

1	O	3.6454500000	9.6339680000	-3.6072890000
2	O	-1.4845200000	11.4263840000	-3.7974500000
3	O	1.1470980000	12.1011020000	-2.8084310000
4	O	-0.6567270000	13.7428670000	-4.1895530000
5	O	-1.2014660000	12.9551760000	-1.9869960000
6	O	-16.9887260000	5.5426600000	1.2529210000
7	O	-14.9494150000	1.2168620000	2.7009320000
8	O	-15.1951220000	-4.4543660000	-1.6360000000
9	O	-13.1641480000	-5.5638520000	-0.5134480000
10	O	-14.0746540000	-3.4561190000	0.7284080000
11	O	-15.5409270000	-5.7359870000	0.4729100000
12	O	-17.5227070000	-7.5275510000	0.4931070000
13	O	-16.3010490000	-8.5028680000	-1.1701350000
14	O	-16.2493350000	-7.4484980000	3.8405530000
15	O	-15.7114690000	-7.3372860000	6.0252440000
16	O	-15.4671550000	2.3099090000	0.7978930000
17	O	11.8119060000	-9.2521060000	4.8730780000
18	O	9.4718500000	-9.7672650000	-2.3032740000
19	O	13.9934820000	-5.4814740000	1.0304020000
20	O	-2.3148060000	-5.3656270000	3.1650840000
21	O	-3.4307880000	-2.7001220000	1.2904970000
22	O	-7.3737400000	5.0449210000	0.2124060000
23	O	-13.2943450000	10.3190920000	-1.4751190000
24	O	-11.7150900000	11.7343630000	0.3316390000
25	O	-12.2443940000	12.6293840000	-2.0311730000
26	O	-11.8490600000	9.0445320000	-4.2658010000
27	O	-11.0672790000	11.6519070000	-4.8274200000
28	O	-9.3710490000	9.7228230000	-4.6638870000
29	O	-9.2481430000	11.4701240000	-1.9519340000
30	O	-3.2200780000	3.4336890000	-1.8175870000

1	N	-2.8633070000	13.3313970000	-3.2288850000
2	N	-14.6859230000	0.1494750000	0.7299060000
3	N	11.4548930000	-8.1949390000	1.3031510000
4	N	8.0640090000	-8.0606320000	-0.5109370000
5	N	4.7784360000	-6.2548340000	-1.1009380000
6	N	2.0342720000	-5.3331390000	-1.0559370000
7	N	-1.2486990000	-4.1732910000	0.6012360000
8	N	-7.1832230000	6.6638110000	-1.3257960000
9	N	-3.3791230000	1.7560220000	-0.3263080000
10	N	-3.2263870000	-1.0080580000	-0.1732090000
11	C	16.3787610000	9.3988270000	5.4093930000
12	C	16.4135590000	10.5938320000	4.4487340000
13	C	16.1794070000	10.1722040000	2.9891560000
14	C	16.2434250000	11.3781760000	2.0393580000
15	C	15.9961780000	10.9920580000	0.5727700000
16	C	16.1687240000	12.1988500000	-0.3635280000
17	C	15.8366010000	11.8563310000	-1.8240620000
18	C	15.9203440000	13.0937810000	-2.7299210000
19	C	15.6653420000	12.7246990000	-4.1691190000
20	C	14.4854530000	12.7473880000	-4.8097180000
21	C	13.1415750000	13.1394060000	-4.2441100000
22	C	12.1690280000	11.9496540000	-4.2935890000
23	C	10.7341490000	12.3343920000	-3.8941150000
24	C	9.7758710000	11.1387380000	-4.0266720000
25	C	8.3077450000	11.5140390000	-3.7637670000
26	C	7.3696030000	10.3199520000	-4.0127950000
27	C	20.2597600000	12.8088710000	8.0775750000
28	C	18.8166690000	13.1916840000	7.7241500000
29	C	18.6937670000	13.6756980000	6.2707710000
30	C	17.2486710000	14.0590460000	5.9141110000

1	C	17.1170230000	14.4991720000	4.4470170000
2	C	15.6572840000	14.7889050000	4.0634210000
3	C	15.5196880000	15.2084910000	2.5909550000
4	C	14.0503710000	15.4096590000	2.1898820000
5	C	13.9290900000	15.7195100000	0.7184760000
6	C	13.3818090000	14.9332600000	-0.2227070000
7	C	12.7713090000	13.5693080000	-0.0126890000
8	C	11.2461290000	13.6763260000	0.1097870000
9	C	10.5852440000	12.2997720000	0.2850740000
10	C	9.0558730000	12.4237030000	0.3537090000
11	C	8.3622980000	11.0600400000	0.5003440000
12	C	6.8338940000	11.2123430000	0.4697050000
13	C	6.1022040000	9.8687080000	0.6128250000
14	C	4.6122050000	10.0276760000	0.3565050000
15	C	5.8870250000	10.6700990000	-3.7880410000
16	C	4.9453810000	9.4887450000	-4.0093840000
17	C	3.0961570000	10.4398980000	-1.4221650000
18	C	3.1240070000	10.7394600000	-2.9229710000
19	C	1.7045180000	10.9803660000	-3.4469820000
20	C	-2.9365120000	13.4283830000	-4.7482120000
21	C	-1.5039720000	13.6158300000	-5.2927150000
22	C	-17.4663710000	6.5607620000	0.3997440000
23	C	-16.4955510000	4.4368920000	0.5363280000
24	C	-15.9939940000	3.3915740000	1.5218320000
25	C	-26.1690660000	1.5171040000	15.5184950000
26	C	-24.9655100000	0.5924490000	15.2925620000
27	C	-24.7628130000	0.2708040000	13.8033540000
28	C	-23.5568700000	-0.6546970000	13.5740960000
29	C	-22.3233730000	-4.5172470000	-16.2368240000
30	C	-21.3233460000	-5.1975790000	-15.2924470000

1	C	-21.6132000000	-4.8698260000	-13.8190360000
2	C	-20.6137510000	-5.5523140000	-12.8709890000
3	C	-20.9043960000	-5.2294020000	-11.3961250000
4	C	-23.3531870000	-0.9787810000	12.0848690000
5	C	-14.5954880000	-2.2283330000	0.2885350000
6	C	-14.2015480000	-1.1235250000	1.2675440000
7	C	-15.2237860000	-6.9641800000	1.0749350000
8	C	-16.5280140000	-7.6632290000	1.4751020000
9	C	-17.0942010000	-7.0947660000	2.7781350000
10	C	-17.2166750000	-7.7990680000	-0.8082230000
11	C	-18.1526990000	-7.0384000000	-1.7342630000
12	C	-16.4454080000	-7.0050220000	5.1176260000
13	C	-17.6358820000	-6.0737420000	5.3280390000
14	C	-17.8639430000	-5.6955510000	6.8028610000
15	C	-19.0793950000	-4.7714910000	6.9883640000
16	C	-19.2992880000	-4.4152510000	8.4678320000
17	C	-20.5132020000	-3.4943330000	8.6716270000
18	C	-20.7261690000	-3.1537650000	10.1558840000
19	C	-21.9370820000	-2.2316830000	10.3723480000
20	C	-22.1447220000	-1.9021240000	11.8598120000
21	C	-17.8475220000	-7.2595930000	-3.2240410000
22	C	-18.8213320000	-6.4984420000	-4.1386650000
23	C	-18.5206150000	-6.7554820000	-5.6243880000
24	C	-19.5047340000	-6.0263700000	-6.5536850000
25	C	-19.2083400000	-6.3148430000	-8.0346580000
26	C	-20.2003150000	-5.6096910000	-8.9738440000
27	C	-19.9073820000	-5.9196800000	-10.4511310000
28	C	-15.0212640000	1.2268800000	1.4945780000
29	C	10.5491340000	-6.7745370000	7.7824170000
30	C	9.0923640000	-6.5061030000	8.1505560000

1	C	8.2412900000	-6.2730080000	6.8976630000
2	C	6.7739180000	-6.0145230000	7.2638040000
3	C	5.9306880000	-5.7990620000	6.0022220000
4	C	4.4519730000	-5.5706040000	6.3436000000
5	C	3.6305560000	-5.3992350000	5.0612920000
6	C	8.8402020000	-20.1547880000	-9.5696720000
7	C	8.3831130000	-19.6105250000	-8.2193760000
8	C	8.6178900000	-18.0985720000	-8.1237330000
9	C	8.1628980000	-17.5570070000	-6.7634640000
10	C	8.4064210000	-16.0465480000	-6.6623100000
11	C	7.9677820000	-15.5096660000	-5.2948020000
12	C	8.2333930000	-14.0036530000	-5.1826610000
13	C	23.3593140000	-12.1546840000	-4.7641730000
14	C	21.8498550000	-11.9328100000	-4.7936350000
15	C	21.4056090000	-10.9984730000	-3.6621920000
16	C	19.8878020000	-10.7824570000	-3.6914340000
17	C	19.4420280000	-9.8559250000	-2.5539460000
18	C	17.9220860000	-9.6541550000	-2.5713710000
19	C	17.4747610000	-8.7481060000	-1.4181990000
20	C	23.3850490000	-14.8405780000	4.5907340000
21	C	22.1068850000	-14.6674930000	3.7754450000
22	C	21.1645230000	-13.6519700000	4.4319960000
23	C	19.8821340000	-13.4819220000	3.6093420000
24	C	18.9372530000	-12.4669650000	4.2637710000
25	C	17.6567770000	-12.2999070000	3.4377490000
26	C	16.7078610000	-11.2845980000	4.0859210000
27	C	2.1333360000	-5.2264720000	5.3546170000
28	C	15.4337920000	-11.1213880000	3.2502170000
29	C	15.9510800000	-8.5757230000	-1.4113720000
30	C	15.5028200000	-7.7092440000	-0.2285420000

1	C	14.4796730000	-10.0997000000	3.8808870000
2	C	13.2230120000	-9.9346480000	3.0182750000
3	C	12.2580530000	-8.8748020000	3.5936220000
4	C	7.8258660000	-13.4758200000	-3.8019630000
5	C	8.1297930000	-11.9785400000	-3.6716950000
6	C	7.7667970000	-11.4659110000	-2.2706160000
7	C	8.0970470000	-9.9727760000	-2.0971470000
8	C	13.9731000000	-7.5927810000	-0.1764040000
9	C	13.5115510000	-6.8016850000	1.0680230000
10	C	7.7174390000	-9.4991330000	-0.6841100000
11	C	11.0523590000	-8.6415150000	2.6604390000
12	C	11.9760770000	-6.8051070000	1.2326870000
13	C	10.4312470000	-8.4861910000	0.2728450000
14	C	9.0556920000	-7.8432090000	0.5739070000
15	C	6.8585830000	-7.2484640000	-0.2239570000
16	C	5.9317450000	-7.1108550000	-1.4476610000
17	C	2.6503740000	-6.0451570000	0.0928970000
18	C	3.7362210000	-7.0088310000	-0.3777730000
19	C	4.1751410000	-5.6203360000	-2.2856580000
20	C	3.0855910000	-4.6371980000	-1.8311650000
21	C	1.3534820000	-5.1314280000	4.0384410000
22	C	-0.1702890000	-5.0536440000	4.2595380000
23	C	-0.9455310000	-5.1567230000	2.9245570000
24	C	-0.7576120000	-3.9255770000	1.9918130000
25	C	-0.2995480000	-5.0310050000	-0.1720690000
26	C	1.0425060000	-4.3475840000	-0.5620940000
27	C	-1.5369520000	-2.9050990000	-0.1188560000
28	C	-2.7898660000	-2.1897760000	0.3810970000
29	C	-6.7401500000	5.5319710000	-0.7155040000
30	C	-5.4636660000	4.8784230000	-1.1953220000

1	C	-5.1125060000	3.5987990000	-0.4135610000
2	C	-10.3090140000	10.5617810000	-2.0965420000
3	C	-9.9778780000	9.2644990000	-1.3267890000
4	C	-8.6802380000	8.5678320000	-1.7702830000
5	C	-8.4056640000	7.3283130000	-0.9098560000
6	C	-3.8403060000	2.9220100000	-0.8942960000
7	C	-4.0678510000	1.0752430000	0.7695350000
8	C	-4.4332290000	-0.3398890000	0.3140260000
9	C	-2.5281610000	-0.3262020000	-1.2630540000
10	C	-2.1681890000	1.0939220000	-0.8109240000
11	H	15.4229820000	8.9215800000	5.3504910000
12	H	17.1432460000	8.7009440000	5.1383270000
13	H	16.5459850000	9.7403380000	6.4095930000
14	H	15.5850970000	11.2159340000	4.7161850000
15	H	17.4097930000	10.9819890000	4.4910820000
16	H	15.1766310000	9.8023210000	2.9394550000
17	H	16.9980870000	9.5361350000	2.7242290000
18	H	15.4325570000	12.0182880000	2.3181140000
19	H	17.2498840000	11.7386030000	2.0853560000
20	H	14.9677650000	10.7039240000	0.5064520000
21	H	16.7649530000	10.2953530000	0.3109960000
22	H	15.4428450000	12.9218220000	-0.0545560000
23	H	17.2099160000	12.4443630000	-0.3443650000
24	H	14.8130250000	11.5450760000	-1.8344460000
25	H	16.6025070000	11.1876560000	-2.1574460000
26	H	15.1195840000	13.7397790000	-2.4359240000
27	H	16.9319350000	13.4388010000	-2.6797730000
28	H	16.5141210000	12.4114020000	-4.7403710000
29	H	14.4992880000	12.4542700000	-5.8386020000
30	H	12.7421850000	13.8871230000	-4.8970530000

1	H	13.2992140000	13.3616130000	-3.2093430000
2	H	12.5081430000	11.2591590000	-3.5498900000
3	H	12.1202990000	11.6498120000	-5.3195170000
4	H	10.7666400000	12.5809540000	-2.8534980000
5	H	10.4078050000	13.0629370000	-4.6066060000
6	H	10.0490260000	10.4538680000	-3.2512650000
7	H	9.8239120000	10.8338780000	-5.0511790000
8	H	8.2367150000	11.7408600000	-2.7205530000
9	H	8.0512470000	12.2556770000	-4.4912000000
10	H	7.6170300000	9.5891880000	-3.2713950000
11	H	7.4633760000	10.0811290000	-5.0516220000
12	H	20.2950500000	11.7793980000	8.3670170000
13	H	20.6026230000	13.4188760000	8.8870470000
14	H	20.8881460000	12.9614070000	7.2250270000
15	H	18.2428090000	12.2909030000	7.7897920000
16	H	18.5690490000	14.0299480000	8.3414190000
17	H	18.9422040000	12.8374080000	5.6538910000
18	H	19.2672540000	14.5766360000	6.2056150000
19	H	16.6689090000	13.1649930000	6.0117350000
20	H	17.0116310000	14.9177780000	6.5067450000
21	H	17.4216890000	13.6617870000	3.8546990000
22	H	17.6383970000	15.4300040000	4.3657240000
23	H	15.1329860000	13.8612100000	4.1602230000
24	H	15.3579000000	15.6357550000	4.6449190000
25	H	15.8793070000	14.3852670000	2.0096180000
26	H	15.9874050000	16.1672340000	2.5072640000
27	H	13.5630550000	14.4694150000	2.3428040000
28	H	13.7101610000	16.2796340000	2.7118100000
29	H	14.3185390000	16.6609430000	0.3916970000
30	H	13.3781610000	15.3125520000	-1.2231810000

1	H	13.1291810000	13.2172690000	0.9321940000
2	H	12.9671010000	13.0073960000	-0.9019720000
3	H	11.0556550000	14.2168990000	1.0133490000
4	H	10.8967910000	14.0589540000	-0.8263790000
5	H	10.9019420000	11.9314770000	1.2385070000
6	H	10.8040670000	11.7439210000	-0.6026450000
7	H	8.8413110000	12.9646410000	1.2516640000
8	H	8.7464160000	12.8144870000	-0.5930830000
9	H	8.6109660000	10.6972470000	1.4757860000
10	H	8.6274000000	10.4907410000	-0.3658500000
11	H	6.5753960000	11.7845950000	1.3360210000
12	H	6.5919840000	11.5793090000	-0.5058090000
13	H	6.2036790000	9.5763800000	1.6370620000
14	H	6.4771040000	9.2354910000	-0.1639500000
15	H	5.7991480000	10.9259100000	-2.7527340000
16	H	5.6376970000	11.3894890000	-4.5398160000
17	H	2.5633130000	9.5202900000	-1.2988970000
18	H	2.7510260000	11.3277040000	-0.9346860000
19	H	3.7260310000	11.6098360000	-3.0807160000
20	H	1.1194210000	10.1347910000	-3.1511530000
21	H	1.7971830000	11.2307470000	-4.4831930000
22	H	-3.3685280000	14.0923380000	-2.8216360000
23	H	-1.9074480000	13.3722170000	-2.9379050000
24	H	-3.2862300000	12.4826090000	-5.1059520000
25	H	-3.4786650000	14.3215080000	-4.9793020000
26	H	-1.2351350000	12.7113300000	-5.7971920000
27	H	-1.4833390000	14.5478070000	-5.8179450000
28	H	-1.2021860000	11.1678090000	-4.6777870000
29	H	-18.2605380000	6.1757840000	-0.2052060000
30	H	-16.6709720000	6.9007800000	-0.2299250000

1	H	-17.8297640000	7.3775460000	0.9877980000
2	H	-17.3277080000	4.0043040000	0.0212520000
3	H	-15.6441690000	4.7783840000	-0.0144490000
4	H	-16.8490180000	3.0260850000	2.0512330000
5	H	-15.1808760000	3.8335810000	2.0587980000
6	H	-15.9753670000	-5.0103320000	-1.6963410000
7	H	-27.0563240000	1.0431190000	15.1539200000
8	H	-26.0151740000	2.4373290000	14.9945870000
9	H	-26.2748190000	1.7157060000	16.5646330000
10	H	-25.2062490000	-0.3347180000	15.7693640000
11	H	-24.1018040000	1.1439120000	15.6003670000
12	H	-25.6264070000	-0.2811820000	13.4960510000
13	H	-24.5232390000	1.1982470000	13.3266800000
14	H	-23.7963600000	-1.5817190000	14.0517270000
15	H	-22.6934080000	-0.1022340000	13.8807310000
16	H	-22.8450970000	-5.2621090000	-16.8005530000
17	H	-21.7977360000	-3.8673850000	-16.9047810000
18	H	-23.0246740000	-3.9477300000	-15.6632960000
19	H	-21.4774420000	-6.2508490000	-15.4011330000
20	H	-20.3666490000	-4.7715690000	-15.5116300000
21	H	-22.5707600000	-5.2944730000	-13.6006270000
22	H	-21.4576400000	-3.8168080000	-13.7104610000
23	H	-20.7672500000	-6.6052560000	-12.9824510000
24	H	-19.6569390000	-5.1252350000	-13.0875890000
25	H	-21.8635150000	-5.6519040000	-11.1802660000
26	H	-20.7454790000	-4.1773850000	-11.2827150000
27	H	-24.2152900000	-1.5333860000	11.7783230000
28	H	-23.1152870000	-0.0517960000	11.6063580000
29	H	-14.1133840000	-2.0038340000	-0.6399650000
30	H	-15.6613500000	-2.3125180000	0.3313140000

1	H	-13.1326000000	-1.0758400000	1.2745870000
2	H	-14.7350070000	-1.3042900000	2.1772880000
3	H	-13.9163480000	0.4806050000	0.1838790000
4	H	-14.7623710000	-7.5703980000	0.3235820000
5	H	-14.6982580000	-6.7417800000	1.9801310000
6	H	-16.2738270000	-8.6954080000	1.5967160000
7	H	-17.0564400000	-6.0288240000	2.6935890000
8	H	-18.0297830000	-7.5850350000	2.9494850000
9	H	-19.1269560000	-7.4490710000	-1.5696220000
10	H	-17.9710910000	-6.0005190000	-1.5479840000
11	H	-18.5000910000	-6.6301520000	5.0305860000
12	H	-17.3878010000	-5.1619570000	4.8260820000
13	H	-18.1023050000	-6.6067840000	7.3105780000
14	H	-17.0084440000	-5.1256160000	7.0998300000
15	H	-19.9357560000	-5.3365400000	6.6846260000
16	H	-18.8391290000	-3.8551650000	6.4909150000
17	H	-19.5352620000	-5.3336110000	8.9636360000
18	H	-18.4422100000	-3.8496860000	8.7687850000
19	H	-21.3710450000	-4.0562790000	8.3663220000
20	H	-20.2760810000	-2.5723580000	8.1829840000
21	H	-20.9630260000	-4.0766370000	10.6427560000
22	H	-19.8666060000	-2.5936810000	10.4598180000
23	H	-22.7973200000	-2.7892890000	10.0657770000
24	H	-21.6995950000	-1.3064690000	9.8903480000
25	H	-22.3825670000	-2.8279150000	12.3405720000
26	H	-21.2830750000	-1.3463040000	12.1656960000
27	H	-18.0144170000	-8.2997650000	-3.4112060000
28	H	-16.8815150000	-6.8331590000	-3.3969040000
29	H	-19.7917270000	-6.9091830000	-3.9528030000
30	H	-18.6400180000	-5.4573740000	-3.9708480000

1	H	-18.6794770000	-7.8015810000	-5.7840000000
2	H	-17.5589780000	-6.3255880000	-5.8125510000
3	H	-20.4702620000	-6.4420310000	-6.3540980000
4	H	-19.3328670000	-4.9799040000	-6.4113320000
5	H	-19.3633800000	-7.3647590000	-8.1709210000
6	H	-18.2494710000	-5.8850300000	-8.2363220000
7	H	-21.1619070000	-6.0294380000	-8.7642320000
8	H	-20.0362930000	-4.5596470000	-8.8495370000
9	H	-20.0608460000	-6.9717770000	-10.5716900000
10	H	-18.9499530000	-5.4910170000	-10.6622140000
11	H	10.6255260000	-7.6803420000	7.1436530000
12	H	10.9762820000	-5.9065590000	7.2364010000
13	H	11.1423230000	-6.9406960000	8.7062940000
14	H	8.6985130000	-7.3811030000	8.7123490000
15	H	9.0453520000	-5.6098330000	8.8066890000
16	H	8.3022570000	-7.1718950000	6.2445310000
17	H	8.6409740000	-5.3980850000	6.3388520000
18	H	6.3780620000	-6.8910910000	7.8227940000
19	H	6.7048110000	-5.1141220000	7.9131610000
20	H	6.0171700000	-6.7002670000	5.3551470000
21	H	6.3207710000	-4.9200990000	5.4427170000
22	H	4.0694100000	-6.4510020000	6.9058460000
23	H	4.3467430000	-4.6644830000	6.9797950000
24	H	3.7679030000	-6.3064260000	4.4311690000
25	H	4.0028570000	-4.5156690000	4.4969340000
26	H	8.2742340000	-19.6697900000	-10.3932450000
27	H	9.9259290000	-19.9685240000	-9.7134450000
28	H	8.6592690000	-21.2496110000	-9.6101980000
29	H	7.3006220000	-19.8296370000	-8.0913870000
30	H	8.9496310000	-20.1282430000	-7.4146780000

1	H	8.0510470000	-17.5859840000	-8.9320430000
2	H	9.7021480000	-17.8879160000	-8.2578380000
3	H	7.0785360000	-17.7650470000	-6.6273220000
4	H	8.7300940000	-18.0734520000	-5.9575870000
5	H	7.8375720000	-15.5251980000	-7.4635930000
6	H	9.4911930000	-15.8428340000	-6.8031180000
7	H	6.8824120000	-15.7056670000	-5.1498690000
8	H	8.5363820000	-16.0392960000	-4.4985240000
9	H	7.6618010000	-13.4646400000	-5.9700310000
10	H	9.3191340000	-13.8157960000	-5.3367750000
11	H	23.6628130000	-12.6172990000	-3.8008190000
12	H	23.6518620000	-12.8342920000	-5.5922570000
13	H	23.8941720000	-11.1893060000	-4.8902060000
14	H	21.3415520000	-12.9160910000	-4.6874810000
15	H	21.5706320000	-11.4908460000	-5.7749310000
16	H	21.6933070000	-11.4456420000	-2.6849300000
17	H	21.9189460000	-10.0176400000	-3.7719620000
18	H	19.3778400000	-11.7654380000	-3.5835230000
19	H	19.5974010000	-10.3339000000	-4.6670630000
20	H	19.7386210000	-10.3066110000	-1.5809020000
21	H	19.9465460000	-8.8702770000	-2.6601910000
22	H	17.4243450000	-10.6440210000	-2.4700820000
23	H	17.6184220000	-9.1983760000	-3.5395560000
24	H	17.7910730000	-9.2079020000	-0.4554680000
25	H	17.9609990000	-7.7525000000	-1.5158940000
26	H	24.0468450000	-15.5800840000	4.0926950000
27	H	23.9269550000	-13.8739990000	4.6688970000
28	H	23.1451990000	-15.2084190000	5.6111630000
29	H	21.5972180000	-15.6523590000	3.6944880000
30	H	22.3772020000	-14.3206950000	2.7540980000

1	H	20.9009900000	-14.0032830000	5.4541670000
2	H	21.6816250000	-12.6703240000	4.5129540000
3	H	19.3658280000	-14.4640960000	3.5280130000
4	H	20.1463430000	-13.1310980000	2.5871060000
5	H	18.6718240000	-12.8173450000	5.2856670000
6	H	19.4526180000	-11.4843720000	4.3446110000
7	H	17.1419670000	-13.2827800000	3.3563140000
8	H	17.9240630000	-11.9505140000	2.4156640000
9	H	16.4364730000	-11.6318780000	5.1073020000
10	H	17.2222680000	-10.3013630000	4.1662710000
11	H	1.7739520000	-6.1106710000	5.9260100000
12	H	1.9647150000	-4.3114980000	5.9633980000
13	H	14.9182030000	-12.1039550000	3.1691550000
14	H	15.7118600000	-10.7782380000	2.2285920000
15	H	15.4780040000	-9.5790320000	-1.3235120000
16	H	15.6210810000	-8.1065580000	-2.3642640000
17	H	15.8558720000	-8.1854870000	0.7133870000
18	H	15.9575670000	-6.6979360000	-0.3104240000
19	H	14.1932540000	-10.4376990000	4.9007310000
20	H	14.9982100000	-9.1185560000	3.9595690000
21	H	12.6964740000	-10.9103930000	2.9292750000
22	H	13.5433720000	-9.6286370000	2.0026910000
23	H	11.3298290000	-10.1158720000	4.7845020000
24	H	12.7957130000	-7.9133460000	3.7121650000
25	H	6.7392380000	-13.6507110000	-3.6412810000
26	H	8.3961440000	-14.0289200000	-3.0228570000
27	H	7.5552630000	-11.4112020000	-4.4364850000
28	H	9.2159390000	-11.8211850000	-3.8499180000
29	H	6.6796770000	-11.6246330000	-2.0959910000
30	H	8.3357990000	-12.0509660000	-1.5141340000

1	H	9.5847960000	-9.3954070000	-3.2168860000
2	H	7.4980020000	-9.3768200000	-2.8272070000
3	H	13.6278330000	-5.0504550000	0.2138230000
4	H	13.5566100000	-8.6214310000	-0.1295530000
5	H	13.5903430000	-7.1091740000	-1.1018280000
6	H	13.9585230000	-7.2867630000	1.9555970000
7	H	6.6313970000	-9.6901390000	-0.5287960000
8	H	8.2182920000	-10.1414920000	0.0722530000
9	H	10.5026560000	-9.6072550000	2.5884080000
10	H	10.3659190000	-7.8977030000	3.1228940000
11	H	11.5494870000	-6.2555030000	0.3635150000
12	H	11.6951560000	-6.2234030000	2.1385770000
13	H	10.3382340000	-9.5875130000	0.1902160000
14	H	10.7953270000	-8.1301680000	-0.7158410000
15	H	8.6480000000	-8.2269070000	1.5352200000
16	H	9.2324090000	-6.7519030000	0.6911140000
17	H	6.3255700000	-7.6756690000	0.6520410000
18	H	7.1542290000	-6.2097430000	0.0480010000
19	H	5.5850060000	-8.0949480000	-1.8316740000
20	H	6.5396570000	-6.6342400000	-2.2492090000
21	H	1.8991970000	-6.6423550000	0.6461390000
22	H	3.1115910000	-5.3217100000	0.8048270000
23	H	4.1509520000	-7.5067800000	0.5218080000
24	H	3.2880840000	-7.7921220000	-1.0312430000
25	H	3.7402250000	-6.3874330000	-2.9664840000
26	H	4.9438850000	-5.0458880000	-2.8476870000
27	H	3.5595160000	-3.8390820000	-1.2138120000
28	H	2.6401200000	-4.1645980000	-2.7341290000
29	H	1.5730950000	-6.0465280000	3.4442800000
30	H	1.7171820000	-4.2487570000	3.4746430000

H	-2.6453240000	-4.6000450000	3.7045560000
H	-0.4613470000	-5.9322740000	4.8770200000
H	-0.4476010000	-4.1305340000	4.8133920000
H	-0.5615050000	-6.0777350000	2.4484580000
H	-1.2882250000	-3.0756740000	2.4713060000
H	0.2931780000	-3.6054420000	1.9664230000
H	-0.1104410000	-5.9534120000	0.3959770000
H	-0.7809410000	-5.3825140000	-1.1122920000
H	1.4979710000	-3.7799230000	0.2724130000
H	0.8081350000	-3.5971640000	-1.3495900000
H	-5.5875360000	4.6228840000	-2.2703080000
H	-4.6336530000	5.6108910000	-1.0927440000
H	-5.9547940000	2.8820230000	-0.5283760000
H	-4.9898370000	3.8629290000	0.6594940000
H	-13.5964810000	10.4323550000	-2.4114700000
H	-11.8260440000	10.8841660000	0.8269650000
H	-11.4344890000	8.1789850000	-4.0211950000
H	-12.0464170000	11.7383990000	-4.7070820000
H	-9.5013140000	12.2993660000	-2.4344840000
H	-9.8689980000	9.5242880000	-0.2503630000
H	-10.8271630000	8.5527670000	-1.4252300000
H	-7.8257370000	9.2721490000	-1.6714890000
H	-8.7620860000	8.2401750000	-2.8248670000
H	-8.3119120000	7.6330180000	0.1558590000
H	-9.2581570000	6.6203640000	-1.0098440000
H	-6.6329900000	7.0536140000	-2.1075730000
H	-4.9871610000	1.5886300000	1.1106530000
H	-3.3828270000	1.0122550000	1.6434130000
H	-4.9225690000	-0.8774150000	1.1538740000
H	-5.1705620000	-0.2737740000	-0.5161120000

H	-3.2090190000	-0.2619990000	-2.1400530000
H	-1.6079590000	-0.8418380000	-1.5984790000
H	-1.4298910000	1.0362610000	0.0188680000
H	-1.6845620000	1.6327930000	-1.6534120000
H	-2.1461110000	-4.7120950000	0.6525830000
H	-0.6648930000	-2.2194000000	-0.0324160000
H	-1.7169060000	-3.1288500000	-1.1944590000

7. References

[1] a) W. P. Aue, E. Bartholdi, R. R. Ernst., *J. Chem. Phys.* **1976**, *64*(5), 2229−2246. b) G. E. Martin, A. S. Zektzer, *Two-Dimensional NMR Methods for Establishing Molecular Connectivity*, Wiley-VCH, **1988**. c) G. D. Mateescu, A. Valeriu, *2D NMR: density matrix and product operator treatment*, Prentice Hall, **1993**.

[2] J. Keeler, *Understanding NMR spectroscopy*, John Wiley & Sons, **2010**.

[3] Gaussian 16, Revision C.01, M. J. Frisch, G. W. Trucks, H. B. Schlegel, G. E. Scuseria, M. A. Robb, J. R. Cheeseman, G. Scalmani, V. Barone, G. A. Petersson, H. Nakatsuji, X. Li, M. Caricato, A. V. Marenich, J. Bloino, B. G. Janesko, R. Gomperts, B. Mennucci, H. P. Hratchian, J. V. Ortiz, A. F. Izmaylov, J. L. Sonnenberg, D. Williams-Young, F. Ding, F. Lipparini, F. Egidi, J. Goings, B. Peng, A. Petrone, T. Henderson, D. Ranasinghe, V. G. Zakrzewski, J. Gao, N. Rega, G. Zheng, W. Liang, M. Hada, M. Ehara, K. Toyota, R. Fukuda, J. Hasegawa, M. Ishida, T. Nakajima, Y. Honda, O. Kitao, H. Nakai, T. Vreven, K. Throssell, J. A. Montgomery, Jr., J. E. Peralta, F. Ogliaro, M. J. Bearpark, J. J. Heyd, E. N. Brothers, K. N. Kudin, V. N. Staroverov, T. A. Keith, R. Kobayashi, J. Normand, K. Raghavachari, A. P. Rendell, J. C. Burant, S. S. Iyengar, J. Tomasi, M. Cossi, J. M. Millam, M. Klene, C. Adamo, R. Cammi, J. W. Ochterski, R. L. Martin, K. Morokuma, O. Farkas, J. B. Foresman, D. J. Fox, Gaussian, Inc., Wallingford CT, **2016**.

[4] A. K. Rappé, C. J. Casewit, K. S. Colwell, W. A. Goddard III, W. M. Skiff, *J. Am. Chem. Soc.* **1992**, *114*(25), 10024−10035.

[5] J. Espinosa-Garcia, J. C. Corchado, *J. Phys. Chem.* **1995**, *99*(21), 8613−8616.

[6] GaussView, Version 6.1, Roy Dennington, Todd A. Keith, John M. Millam, Semichem Inc., Shawnee Mission, KS, **2016**.

[7] a) J. X. Chen, *Guide to graphics software tools*, Springer Science & Business Media, **2007**. b) Jmol: an open-source Java viewer for chemical structures in 3D.

[8] J. B. Miller, S. Zhang, P. Kos, H. Xiong, K. Zhou, S. S. Perelman, H. Zhu, D. J. Siegwart, *Angew. Chem. Int. Ed.* **2016**, *129*(55), 1−6.

[9] C. B. W. Phippen, J. K. Beattie, C. S. P. McErlean, *Chem. Commun.* **2010**, *46*(43), 8234−8236.

[10] R. D. Katsarava, D. P. Kharadze, L. M. Avalishvili, M. M. Zaalishvili, *Polym. Sci. USSR* **1984**, *26*(7), 1668−1678.

[11] L. Xue, N. Gong, S. J. Shepherd, X. Xiong, X. Liao, X. Han, G. Zhao, C. Song, X. Huang, H. Zhang, M. S. Padilla, J. Q., Y. Shi, M.-G. Alameh, D. J. Pochan, K. Wang, F. Long, D. Weissman, M. J. Mitchell, *J. Am. Chem. Soc.* **2022**, *144*(22), 9926−9937.

[12] X. Zhang, K. Su, S. Wu, L. Lin, S. He, X. Yan, L. Shi, S. Liu, *Angew. Chem. Int. Ed.* **2024**, *63*(26), e202405444.
